# Global emergence of unprecedented lifetime exposure to climate extremes

**DOI:** 10.1038/s41586-025-08907-1

**Published:** 2025-05-07

**Authors:** Luke Grant, Inne Vanderkelen, Lukas Gudmundsson, Erich Fischer, Sonia I. Seneviratne, Wim Thiery

**Affiliations:** 1 Department of Water and Climate, Vrije Universiteit Brussel, Brussels, Belgium; 2 Canadian Centre for Climate Modelling and Analysis, Environment and Climate Change Canada, Victoria, British Columbia Canada; 3 Department of Earth and Environmental Sciences, KU Leuven, Leuven, Belgium; 4 Royal Meteorological Institute Belgium, Brussels, Belgium; 5 Institute for Atmospheric and Climate Science, ETH Zurich, Zurich, Switzerland

**Keywords:** Projection and prediction, Climate-change impacts

## Abstract

Climate extremes are escalating under anthropogenic climate change^1^. Yet, how this translates into unprecedented cumulative extreme event exposure in a person’s lifetime remains unclear. Here we use climate models, impact models and demographic data to project the number of people experiencing cumulative lifetime exposure to climate extremes above the 99.99th percentile of exposure expected in a pre-industrial climate. We project that the birth cohort fraction facing this unprecedented lifetime exposure to heatwaves, crop failures, river floods, droughts, wildfires and tropical cyclones will at least double from 1960 to 2020 under current mitigation policies aligned with a global warming pathway reaching 2.7 °C above pre-industrial temperatures by 2100. Under a 1.5 °C pathway, 52% of people born in 2020 will experience unprecedented lifetime exposure to heatwaves. If global warming reaches 3.5 °C by 2100, this fraction rises to 92% for heatwaves, 29% for crop failures and 14% for river floods. The chance of facing unprecedented lifetime exposure to heatwaves is substantially larger among population groups characterized by high socioeconomic vulnerabilities. Our results call for deep and sustained greenhouse gas emissions reductions to lower the burden of climate change on current young generations.

## Abstract (web summary)

Climate models, impact models and demographic data are used to estimate the number of people projected to experience unprecedented lifetime exposure to extreme climate events across multiple dimensions, including birth year, warming scenario and vulnerability.

## Main

Climate extremes have detrimental effects on society and are a foremost concern around climate change^1^. Anthropogenic influences have been identified in heatwaves, river floods, droughts, crop failures and certain aspects of wildfires and tropical cyclones^2,3^. With continued atmospheric warming, the intensity, frequency and duration of some of these events are projected to increase further^4–9^, with varying levels and spread depending on the event considered^3^. Current policies could warm global mean temperature (GMT) to +2.7 °C (+2.2–3.4 °C) above pre-industrial levels by the end of the century^10^. As this warming is expected to increase human exposure to climate extremes^3^, young generations will reap the consequences of the present-day mitigation of greenhouse gas emissions.

The above climate extremes are projected to occur most frequently across the lifetimes of current young generations^11^. As such, the number of climate extremes experienced across a person’s lifetime can far exceed the expected exposure under a pre-industrial climate. Yet, the number of people who will experience this unprecedented lifetime exposure (ULE) to climate extremes remains unclear. Here we cross an extensive portfolio of multi-model projections of climate extremes with demographic data, GMT trajectories and two measures of vulnerability. We evaluate the emergence of ULE to extreme events at the grid scale to estimate the global membership of birth cohorts that will face ULE (Methods). Then, we show how this sub-population is stratified in terms of vulnerability. This is one of the first estimates of the number of people projected to experience ULE across a multidimensional framework, including birth year, warming scenario and vulnerability.

## Unprecedented exposure to heatwaves

**Fig. 1:**
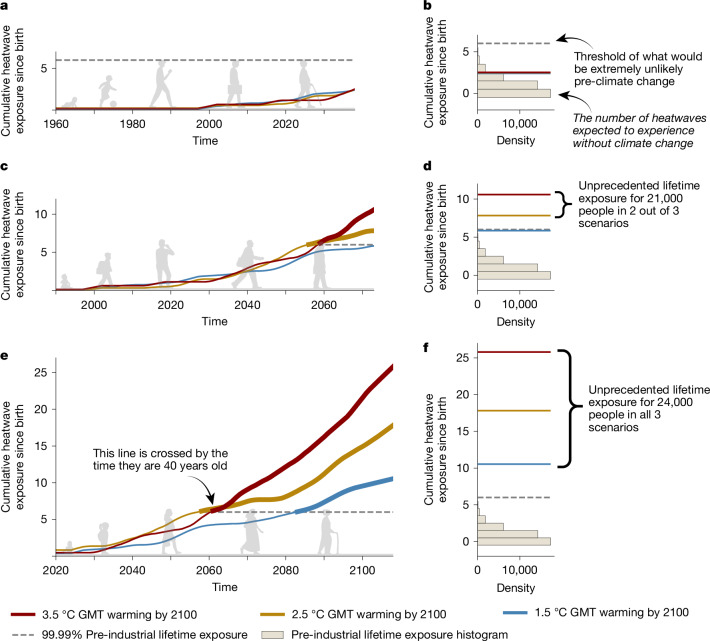
Cumulative heatwave exposure since birth for Brussels, Belgium. **a**,**c**,**e**, Multi-model mean time series of cumulative heatwave exposure for people born in 1960 (**a**), 1990 (**c**) and 2020 (**e**) in 1.5 °C (blue line), 2.5 °C (gold line) and 3.5 °C (red line) pathways. **b**,**d**,**f**, Histograms for 1960 (**b**), 1990 (**d**) and 2020 (**f**) birth cohorts show the pre-industrial sample density of 40,000 bootstrapped lifetime exposures overlaid with final lifetime exposures from the time series of the birth cohort. Dashed lines show the 99.99th percentile of the pre-industrial sample distribution, that is, the threshold of unprecedented lifetime exposure (ULE) for this location, cohort and climate extreme. Counts of people (right of **d**,**f**) show the population of the birth cohort that has emerged beyond the 99.99th percentile of the pre-industrial sample distribution.

We illustrate what ULE means for extreme heatwaves in one grid cell (0.5° × 0.5°) located over Brussels, Belgium, for three GMT pathways in which warming above pre-industrial temperatures reaches 1.5 °C, 2.5 °C and 3.5 °C by the year 2100. People born in 1960 and spending their life in Brussels are projected to experience three heatwaves in their lifetime, showing little sensitivity to the GMT pathway (Fig. 1a). In this location, the 1960 birth cohort does not exceed the threshold of ULE, which we define as the 99.99th percentile of a large sample of lifetime exposures in a pre-industrial control climate and which is six heatwaves here (Fig. 1b, grey histogram and dashed line). By contrast, the 1990 birth cohort emerges into ULE for the two warmest GMT pathways shown (Fig. 1c,d). This implies that, under temperature pathways reaching 2.5 °C or higher warming by 2100, this cohort will face more heatwaves than they would have been expected to experience with a one in ten thousand chance in the absence of climate change. Different GMT pathways cause a further divergence in the lifetime exposure of those born in 2020 in this location (Fig. 1e,f). In the 1.5 °C pathway, the 2020 birth cohort is projected to experience nearly 11 heatwaves, yet this increases to 18 and 26 heatwaves in pathways reaching 2.5 °C and 3.5 °C, respectively, by the end of the century. This by far exceeds the ULE threshold under each GMT pathway, with an age of emergence already around 40 years old for the 2.5 °C and 3.5 °C pathways (Fig. 1e). We then count the number of people per birth cohort that eventually reach ULE, using absolute population estimates at the grid scale and relative cohort sizes at the country level. In this location, a best estimate of 21,000 people from the 1990 birth cohort and 24,000 people from the 2020 birth cohort are projected to experience ULE (except for the 1990 birth cohort under the 1.5 °C pathway). Under a 1.5 °C pathway, all cohorts born in Brussels after 1990 reach ULE, totalling 665,000 people. For a 3.5 °C pathway, ULE begins for people born in 1978, increasing this total to 941,000 people. For cohorts that emerge, it is virtually certain (at least  >99.99% chance) that their lifetime heatwave exposure cannot be explained by internal climate variability.

**Fig. 2:**
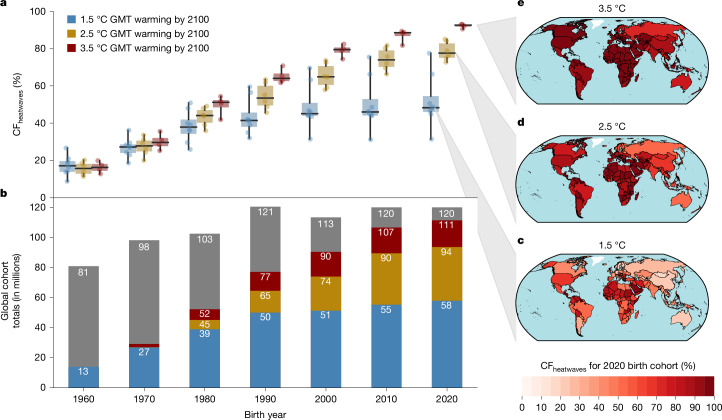
Rising fraction of birth cohorts facing unprecedented lifetime heatwave exposure. **a**, Box plots show the cohort fraction reaching ULE to heatwaves (CF_heatwaves_) for 1.5 °C (blue), 2.5 °C (gold) and 3.5 °C (red) pathways for global birth cohorts between 1960 and 2020 (middle line, median; box limits, upper and lower quartiles; whiskers, extend to the full range of the model ensemble). **b**, Bars show global cohort sizes in millions, with totals in grey and median numbers of people reaching ULE to heatwaves for 1.5 °C (blue), 2.5 °C (gold) and 3.5 °C (red) pathways. **c**–**e**, Maps display country-level CF_heatwaves_ of the 2020 birth cohort for 1.5 °C (**c**), 2.5 °C (**d**) and 3.5 °C (**e**) pathways.

We now repeat this analysis for every land grid cell and project the population fraction of each birth cohort experiencing ULE to heatwaves across the globe (CF_heatwaves_ for cohort fraction reaching ULE to heatwaves). Of the 81 million people born in 1960, on average, around 16% (13 million people) face ULE to heatwaves regardless of the scenario. This fraction rises towards younger generations, and from the 1980 birth cohort onwards, CF_heatwaves_ begins to depend on GMT pathways (Fig. 2a). In a 1.5 °C pathway, CF_heatwaves_ stabilizes for recent birth cohorts, reaching an average of 52% for the 2020 birth cohort (62 million people). Comparatively, CF_heatwaves_ of the 2020 birth cohort is almost doubled in a 3.5 °C pathway, reaching 92%. This implies that 111 million children born in 2020 will live an unprecedented life in terms of heatwave exposure in a world that warms to 3.5 °C compared with 62 million in a 1.5 °C pathway.

At the country level, CF_heatwaves_ for the 2020 birth cohort is the highest in the tropics under low GMT pathways, yet this pattern disappears as heatwaves become widespread under high GMT pathways (Fig. 2c–e and Supplementary Tables 1–3). Under a 1.5 °C pathway, equatorial regions have relatively high CF_heatwaves_; of the 177 countries in this analysis, 104 have most of the population of 2020 birth cohort living with unprecedented exposure to heatwaves (CF_heatwaves_ ≥ 50%; Fig. 2c). This latitudinal pattern is less apparent in a 2.5 °C pathway (Fig. 2d). Here, 157 countries have CF_heatwaves_ ≥ 50%. In a 3.5 °C pathway, 167 countries have CF_heatwaves_ ≥ 50%, 155 countries have CF_heatwaves_ ≥ 90% and in 113 countries the entire birth cohort faces unprecedented heatwave exposure (CF_heatwaves_ = 100%; Fig. 2e).

## Unprecedented multi-hazard exposure

**Fig. 3:**
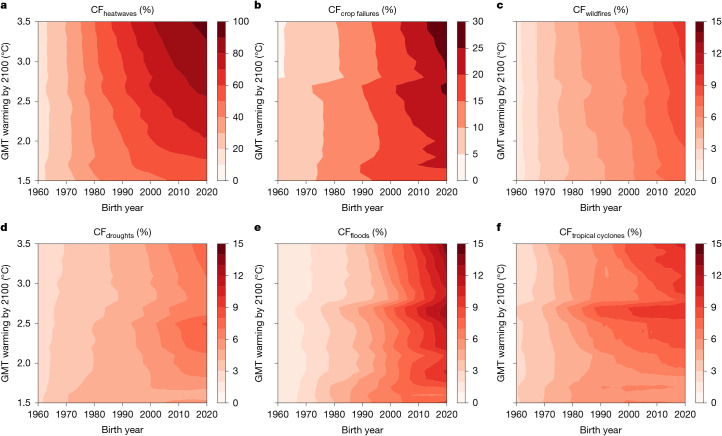
Greater unprecedented exposure to climate extremes for younger generations and higher warming pathways. **a**–**d**, Cohort fraction (CF) across all birth years (1960–2020) and GMT pathways (1.5–3.5 °C) for heatwaves (CF_heatwaves_; **a**), crop failures (CF_crop failures_; **b**), wildfires (CF_wildfires_; **c**), droughts (CF_droughts_; **d**), river floods (CF_floods_; **e**) and tropical cyclones (CF_tropical cyclones_; **f**). Each extreme event panel has its colour bar range.

We then expand the analysis to a total of six climate extremes^12^ and 21 warming pathways (Fig. 3 and Methods). For every combination of birth cohort, climate extreme and warming pathway, we quantify the number of people experiencing ULE at the grid scale and subsequently aggregate to the country or global level. Cohort fraction (CF) for climate extremes other than heatwaves is lower across all birth years and GMT pathways because they are generally less widespread than heatwaves; however, they still affect a large population fraction (Fig. 3 and Supplementary Tables 4–18). In a 3.5 °C pathway, 29% of those born in 2020 will live through unprecedented exposure to crop failures (Fig. 3b). This is followed by river floods, in which 14% will face unprecedented exposure to this extreme (Fig. 3e). As not all climate projections reach high warming levels, the ensemble size shrinks towards higher warming levels. Consequently, crop failures, droughts, river floods and tropical cyclones, which are more dependent on changes in the water cycle than heatwaves, exhibit discontinuities in CF at some GMT intervals (Fig. 3b,d–f). These sampling artefacts disappear when visualizing CFs for a smaller subset of simulations that are available for all GMT trajectories (Supplementary Note 1 and Supplementary Fig. 1). Although model uncertainties are larger for extremes other than heatwaves, differences in CF across birth cohorts are statistically significant for all six climate extremes (Supplementary Note 2 and Supplementary Figs.  2 and 3).

Across all projections available for the 2.7 °C pathway aligned with current policies^10^, ULE to heatwaves occurs in the Americas, Africa, the Middle East and Australia already for the 1960 birth cohort and globally for the 2020 birth cohort (Supplementary Figs. 4m–o and 5e,k). The ULE to crop failures expands around the United States, South America, Sub-Saharan Africa and East Asia between 1960 and 2020 cohorts (Supplementary Figs. 4 and 5b,h). The ULE to river floods occurs in northern latitudes for the 1960 cohort, in line with the observations and model projections for precipitation changes^13–15^ and expands southwards into much of the world for the 2020 cohort (Supplementary Fig. 5d,j).

The lower CF of some extremes, such as tropical cyclones, is expected given the geographical constraints of these events and their distinct meteorological drivers. Tropical cyclones can, therefore, be re-evaluated by limiting the analysis to regions that can experience them. We consider these regions to be any grid cells exposed at least once to the event across our whole ensemble of exposure projections (Supplementary Fig. 6). CF_tropical cyclones_ nearly doubles when constraining total birth cohort size to exposed regions. For the 2020 birth cohort, this estimate changes from 6% to 11% in a 1.5 °C pathway and from 10% to 19% in a 3.5 °C pathway.

## Heatwaves across vulnerability strata

**Fig. 4:**
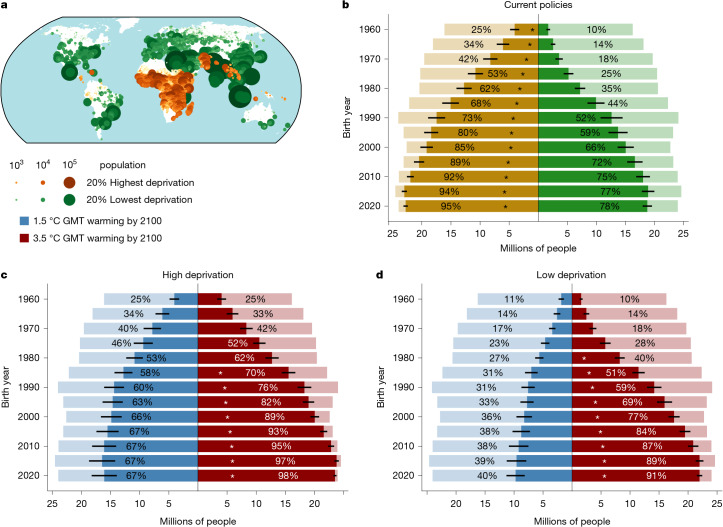
The most deprived face significantly higher chance of ULE to heatwaves. **a**, Geographic distribution of the 20% highest (brown markers) and 20% lowest (green markers) scoring 2020 birth cohort members (with roughly equal population) in the GRDI^16^. Grid cell marker sizes and colours are scaled by their population. **b**, Fraction of these two groups projected to experience ULE to heatwaves under the current policies pathway of 2.7 °C warming by 2100 for every fifth birth year. Light-coloured bars show total cohort sizes per birth year and vulnerability group, whereas dark colours indicate the affected fraction. Error bars show the standard deviation across projections. Asterisks indicate that a low- or high-vulnerability group from a given birth cohort has significantly more members with ULE to heatwaves than the alternative vulnerability group of the same birth cohort (at the 5% level). **c**,**d**, The high deprivation (**c**) and low deprivation (**d**) share of the birth cohort that is projected to experience ULE under the 1.5 °C (blue) and 3.5 °C (red) pathways.

Finally, we cross our grid-scale projections for ULE to heatwaves against two grid-scale indicators of socioeconomic vulnerability (Methods): (1) the Global Gridded Relative Deprivation Index v.1 (GRDI; ref. ^16^), which expresses relative deprivation according to six socioeconomic indicators; and (2) lifetime mean GDP per capita (denoted as GDP; ref. ^17^). Binning our birth cohort members into the top and bottom 20% of GRDI (Fig. 4a) and GDP (Supplementary Fig. 7a) enables a grid-scale comparison of ULE for population groups with high and low socioeconomic vulnerability. Using GRDI, we find that the most vulnerable subset of each birth cohort projected to experience ULE to heatwaves under current policies is substantially larger than the least vulnerable subset. This implies that socioeconomically vulnerable people have a consistently higher chance of facing unprecedented lifetime heatwave exposure compared with the least vulnerable members of their generation (Fig. 4b). For example, of the 2020 birth cohort, 95% or 23 million members of the high deprivation (high socioeconomic vulnerability) group face ULE to heatwaves, whereas this is 78% (19 million) for the low deprivation group. This disparity is similar when using GDP, but with only 1974 and later birth years having significant differences across vulnerability strata (Supplementary Fig. 7). Here, for the 2020 birth year, 92% (22 million) of the low-income group face ULE under current policies, whereas this is 79% (19 million) for the high-income group. Under alternative warming pathways of 1.5 °C and 3.5 °C, although the same direction of disparities remains across vulnerability strata, the lowest vulnerability groups (low deprivation and high GDP) benefit the most from a low warming pathway (Fig. 4c,d and Supplementary Fig. 7c,d). Socioeconomically vulnerable groups have lower adaptive capacity and face more constraints when it comes to implementing effective adaptation measures^18,19^. Our results highlight that precisely these groups with the highest socioeconomic vulnerability and lowest adaptation potential face the highest chance for unprecedented heatwave exposure (Fig. 4). This underlines the disproportionate risk for deprived communities in light of past and future climate extremes.

## Discussion

Our analysis only quantifies local exposure by design; yet in reality, the effects of climate extremes cascade non-locally. For example, in 2023, smoke from an active wildfire season in Canada was transported south along the east coast of the United States, exposing millions of people to hazardous air quality^20^ and causing an increased cardiopulmonary disease burden^21^. Climate extremes also affect society through economic impacts, including the rising cost of living due to supply chain disruptions^22^ and taxation to recover public infrastructure^23^. For instance, climate change endangers staple crop production in the main breadbasket countries that supply most of our caloric intake globally^24^, forcing market instabilities that only the wealthiest can cope with^25^. These missing non-local impacts make our estimates conservative.

By contrast, we do not capture how people adapt to extremes and thereby potentially reduce their exposure or vulnerability. For example, exposure to heatwaves can be reduced for population groups that can afford access to air conditioning^18^. However, maladaptive responses to climate extremes can instead create lock-ins of vulnerability and exposure^1^. Therefore, our lifetime exposure estimates omit beneficial adaptation outcomes as well as detrimental non-local and maladaptation effects. Finally, opting for a threshold below 99.99% would lower the bar and increase ULE estimates, and vice versa. Yet this effect is limited because the reference distribution is typically composed of small integers. By contrast, using thresholds above 99.99% risks redundancy in our bootstrapped data sample (Methods).

Some demographic realities are not accounted for here. Factors such as within-country migration, fertility and mortality respond in reality to the climate extremes considered here^11^. In the United States, where the population faces exposure to all extremes analysed in this study, city centres attract young people^26^ and disparities in life expectancy have been found across race–county combinations^27^ and rural–urban residency^28^. For instance, life expectancy is longer for those living in cities, yet here we apply country-average life expectancy and cohort size distribution uniformly within each country. Furthermore, we do not account for within-grid-cell heterogeneity, that is, we miss some fine-scale variations in socioeconomic vulnerability and exposure in socioeconomically diverse regions such as cities. Finally, we focus on the socioeconomic dimension of vulnerability, thereby neglecting that vulnerability to climate extremes may also vary with, for instance, age, gender or disability status. As demographic and multidimensional vulnerability information becomes available at ever higher spatial resolution and explicitly accounts for climate impact projections, it will become possible to deepen the analysis of the interaction between climate change and population dynamics.

The uncertainties of the extremes other than heatwaves are non-negligible. Hydrological variables have high internal climate variability^29^ and projecting these events requires an additional impact-modelling step relative to heatwaves, which are computed directly from global climate model output (Methods). Furthermore, these events have sensitivities to input data quality and process representation across the modelling chain (Supplementary Note 2). Other uncertainties, such as demographic representation, are not captured in this analysis. Finally, we opt for assessing ULE at the grid scale instead of at the country level. In doing so, we downscale demographic data instead of upscaling climate data, thereby projecting lifetime exposure based on the local climate of individual birth cohort members. This incurs a trade-off for accepting natural variability in locations at which ULE occurs, yet minimizing year-to-year variability in country- and global-scale CF estimates (Supplementary Note 3 and Supplementary Fig. 8).

## Conclusions

In summary, we find that large fractions of global birth cohorts are projected to live unprecedented exposure to heatwaves, river floods, droughts, crop failures, wildfires and tropical cyclones. As the frequency of these six climate extremes increases with warming, so does the fraction of people who will face ULE to these events. More ambitious policies are needed to achieve the goal of the Paris Agreement of limiting global warming to 1.5 °C by 2100 relative to the 2.7 °C warming expected under current policies, especially as the most vulnerable groups have more members projected to face unprecedented exposure to heatwaves. Children would reap the direct benefits of this increased ambition: a total of 613 million children born between 2003 and 2020 would then avoid ULE to heatwaves. For crop failures, this is 98 million, for river floods 64 million, for tropical cyclones 76 million, for droughts 26 million and for wildfires 17 million. This underlines the urgent need for deep and sustained greenhouse gas emission reductions to safeguard the future of current young generations.

## Methods

### ISIMIP and exposure projections

The Inter-Sectoral Impact Model Intercomparison Project (ISIMIP) provides a simulation protocol for projecting the impacts of climate change across sectors such as biomes, agriculture, lakes, water, fisheries, marine ecosystems and permafrost (www.isimip.org). In ISIMIP2b, impact models representing these sectors are run using atmospheric boundary conditions from a consistent set of bias-adjusted global climate models (GCMs) from phase 5 of the Coupled Model Intercomparison Project (CMIP5) that were selected based on their availability of daily data and ability to represent a range of climate sensitivities^17^; the Geophysical Fluid Dynamics Laboratory Earth System Model (GFDL-ESM2M; ref. ^30^), the earth system configuration of the Hadley Centre Global Environmental Model (HadGEM2-ES; ref.^ 31^), the general circulation model from the Institut Pierre-Simon Laplace Coupled Model (IPSL-CM5A-LR; ref.^ 32^) and the Model for Interdisciplinary Research on Climate (MIROC5; ref. ^33^). Impact simulations are run for pre-industrial control (286 ppm CO_2_; 1666–2099), historical (1861–2005) and future (2006–2099) periods. Future simulations are based on Representative Concentration Pathways (RCPs) 2.6, 6.0 and 8.5 of GCM input datasets. Global projections of annual, grid-scale fractions of exposure to each extreme event category are calculated from ISIMIP2b impact simulations and GCM input data. For the full details of these computations, we refer to ref. ^12^, but we summarize extreme event definitions below.

For heatwaves, droughts, crop failures and river floods, we use localized pre-industrial thresholds to determine event occurrences, whereas for tropical cyclones, we use a single absolute threshold, and wildfires are modelled explicitly (Supplementary Table 20). Heatwaves affect an entire grid cell if the Heat Wave Magnitude Index daily (HWMId; refs. ^34,35^) of that year exceeds a threshold in the pre-industrial control HWMId distribution in that grid cell^11^. Although we refer to heatwaves throughout the paper, our definition technically refers to a 3-day extreme heat event that is expected on average once per century under pre-industrial climate conditions. These extreme heat events occur, by definition, everywhere across the world, but with different associated absolute temperature values. Previous analysis highlighted that intergenerational inequalities in lifetime heatwave exposure are robust across a range of heatwave definitions^11^. Crop failures are based on the sum of the area occupied by maize, wheat, soy or rice within a grid cell when their simulated yield falls below a threshold of their pre-industrial reference yield. Droughts, such as heatwaves, affect an entire grid cell if, for 7 months, monthly soil moisture remains below a threshold of pre-industrial soil moisture levels. Floods only correspond to river flooding, and the flooded area is derived from comparing daily discharge simulations from models of the global water sector to pre-industrial discharge. CaMa-Flood, a global river-routing model^36^, is used to convert these discharge values to flooded areas. Tropical cyclones occur if a grid cell sustains hurricane-force winds (≥64 knots) at least once a year^37,38^. Exposure to tropical cyclones does not encompass the flood hazards typically associated with tropical cyclones. Wildfires occur when the burnt area is simulated in a grid cell. Burnt area is either taken directly from annual burnt area calculations or as the annual sum of monthly burnt area in cases in which impact models simulate burnt area sub-annually, capped at 100% of a grid cell. We reiterate that all exposure definitions here neglect potential exposure reduction measures and non-local effects.

We subsequently quantify human exposure to climate extremes in a way that facilitates comparison and aggregation across extreme event categories. We consider all people in a grid cell exposed to a climate extreme in a particular year if the climate extreme occurs in that year. We thereby assume that if such a river flood or wildfire occurs somewhere in a 0.5° × 0.5° grid cell, this is sufficiently close to any person located in that grid cell to be considered affected by this extreme event. Using demographic data (see below), we subsequently convert this annual human exposure to lifetime exposure of birth cohorts by summing annual grid fractions of individual event categories across their lifetimes.

### Demographics

Demographic data for population totals, cohort sizes and life expectancy enable our projection of the CF experiencing ULE to these six extremes. Population totals at the grid scale come from the ISIMIP database (Fig. 2b; ref. ^17^) and originate from population estimates from v.3.2 of the History Database of the Global Environment (HYDE3.2; refs. ^39,40^) for the historical period (1860–2000) and population projections from middle-of-the-road Shared Socioeconomic Pathway (SSP2; refs. ^41,42^) for the future period (2010–2100). We note that these datasets at present do not account for the impact of climate on population dynamics, for example, through changes in migration, fertility and mortality, although these feedbacks may substantially alter the demographic data. Cohort sizes from the Wittgenstein Centre for Demography and Global Human Capital^43^ provide estimates of country-level population totals every 5 years (between 1950 and 2100) for each 5-year age group (0- to 4-year-olds, 5- to 9-year-olds, and so on, until 95- to 99-year-olds and a final age group for those 100 years and older). Life expectancy data come from the United Nations World Population Prospects (UNWPP; ref. ^44^) and describe the life expectancy of 5-year-olds at the country level for 5-year blocks (1950–1955 to 2015–2020). In this dataset, life expectancy is reported for 5-year-olds to exclude biases from infant mortality. Countries that can be spatially resolved at the ISIMIP grid scale and have cohort and life expectancy estimates in these datasets meet the requirements of this study and total 177. We refer to the supplementary material of ref. ^11^ for a broader discussion of these datasets but explain our application of them in this analysis below.

All demographic datasets are modified to represent lifetimes annually, beginning from 1960 to 2020. Life expectancies for each country are first linearly interpolated to annual values by assuming that the values of the original 5-year groups are representative of the middle of that group. Furthermore, we add 5 years to annual life expectancies to capture the life expectancy of each cohort since birth, as the original data begin at age 5. As the maximum UNWPP life expectancy for people born in 2020 prescribes the final year in this analysis (2113), annual population totals must be extrapolated to reach this year. For population totals, we take each year beyond 2100 as the mean of the preceding 10 years of the dataset, such that population numbers for 2101 are the mean of 2091–2100. For cohort sizes in each country, we interpolate annual cohort sizes and age groups from the original 5-year age groups and divide age totals by 5 to maintain original population sizes in this dataset and linearly extrapolate these estimates to 2113. This provides the absolute numbers of 0- to 100-year-olds for each year across 1960–2113.

To downscale this demographic information to the grid scale, we assume spatially homogeneous cohort representation and life expectancy. Birth cohort size is represented as the number of people of age 0 of a given birth year in a given grid cell. This is estimated by multiplying the absolute population of the birth year (using the annual grid-scale population totals from ISIMIP) by the relative size of the age 0 cohort (using the interpolated 0- to 100-year-old population totals from the Wittgenstein Centre cohort data). Spatial variability in age structure and life expectancy within a country is therefore ignored in this study.

### Mapping impacts to GMT trajectories

To project CF across different warming pathways by 2100, we construct a series of incrementally warming GMT pathways between 1960 and 2113 based on GMT trajectories taken from the AR6 Scenario Explorer^45^. The time series from the AR6 scenario explorer were chosen as anchor points for interpolation to produce a range of plausible GMT time series. Furthermore, they were selected to minimize overshooting in the early years of low GMT pathways over higher GMT pathways, which can skew lifetime exposure estimates for early birth cohorts (Supplementary Fig. 9). The upper bound of this subset was limited to 3.5 °C in favour of sampling more simulations for higher GMT projections, which we discuss further below. For the lower bound, 1.5 °C was chosen because it is a more realistic minimal warming scenario than 1.0 °C. Note that the 1.5 °C anchor scenario maximally reaches 1.57 °C before reducing to 1.5 °C by 2100. It is, therefore, referred to as 1.5 °C throughout this analysis. These warming levels are reported relative to pre-industrial temperatures from 1850 to 1900. This yields a total of 21 GMT pathways for which we project CF.

Our dataset of extreme event exposures represents occurrences of these extremes forced by GCM-modelled climates. These climates have unique GMT warming pathways that depend on their radiative forcing scenario (historical or RCP), as prescribed by the ISIMIP2b modelling protocol. To project these exposure maps along even intervals of warming scenarios, which the original simulations do not provide, we use the 21 GMT pathways described above. For each pairing of the 21 target GMT pathways and the concatenated historical and future exposure projections, we sample exposures by matching the GMT warming levels of the exposure series to the years of the target GMT pathways (Supplementary Figs. 9 and 10). The GMT warming levels behind the exposure projections are first smoothed with a 21-year rolling mean before GMT mapping is undertaken. In cases in which our constructed GMT pathways exceed the GMT warming levels of GCM simulations by too much, this mapping erroneously resamples the year of exposures corresponding to the maximum warming level of their forcing GCM. To this end, we implement a constraint in this sampling procedure such that GMT-mapped series are only used if the maximum difference across all GMT pairs is no larger than 0.2 °C. This constraint incrementally reduces ensemble sizes of exposure projections for higher GMT pathways (Supplementary Table 19).

### Lifetime exposure

Estimating lifetime exposure to extreme events requires crossing life expectancy data at the country level with grid-scale exposure projections. For each GMT trajectory (1.5–3.5 °C, 0.1 °C intervals), birth year (1960–2020) and country (177), exposures are summed across lifetimes at the grid scale. This assumes life expectancy to be spatially homogeneous across each country. Exposure during the death years is also included in this sum by multiplying these exposure projections by the fraction of the final year lived. This produces country-wide maps of lifetime exposure at the grid scale for each GMT trajectory and birth year in this analysis.

To generate a baseline distribution of lifetime exposure in a world without climate change, large samples of pre-industrial lifetime exposures are bootstrapped assuming 1960 life expectancy in each country. Here, for each exposure projection originating from a simulation under a pre-industrial climate, 10,000 lifetime exposures are estimated by resampling exposure years with replacement. Depending on ISIMIP2b data availability, pre-industrial exposure projections have a length of 239–639 years per simulation from which to resample from^11^. This process generates 40,000–310,000 country-wide maps of lifetime exposure, depending on the extreme event considered and its underlying data availability, enabling exposure projections in a pre-industrial climate. Using the pre-industrial period as a baseline enables (1) our GMT mapping procedure; (2) bootstrapping a stationary time series to achieve a large reference dataset; and (3) the production of a reference dataset with information that is independent of the projections forming our ULE estimates.

### Emergence of ULE

We define an emergence threshold for ULE to extreme events as the 99.99th percentile of our grid-scale samples of pre-industrial lifetime exposure. When it comes to the selection of this percentile, we went as extreme as possible given the bootstrapping of the pre-industrial control runs. This choice was based on a sensitivity analysis for different percentile values that showed a levelling off of lifetime exposure for percentiles more extreme than 99.99%. This indicated that the 99.99th percentile achieves the limit of reliable information that can be extracted from the empirical distribution. For each extreme event, birth year, GMT pathway and grid cell, we assess if lifetime exposure emerges or passes this threshold of extreme exposure in a pre-industrial climate. If this threshold is passed, we consider the whole birth cohort in this grid cell to have emerged, tallying its size among a global pool of the same birth cohort and GMT trajectory of people projected to live ULE. This means that, in some locations, even if the sum of exposed grid cell fractions across a pre-industrial lifetime does not cover the entire grid cell, we still extract the entire birth cohort size associated with that grid cell. We sum the number of emerged people in each birth cohort globally, although this birth cohort has a different life expectancy in each country. Once the number of people who have emerged globally is tallied, we divide this by the respective total cohort sizes to estimate CF per birth cohort. Note that ULE, therefore, does not refer to unprecedented in terms of the magnitude of assets or people exposed, but rather in terms of the number of events accumulated across an average person’s lifespan in comparison with what they would face in a pre-industrial climate.

### ULE across socioeconomic vulnerability strata

We use two grid-scale indicators of vulnerability to compare with our estimates of ULE to heat waves. The first is an ISIMIP2b GDP input dataset using concatenated historical and SSP2 time series covering 1860–2099 annually^17^. This dataset was disaggregated from the country to grid level using spatial and socioeconomic interactions among cities, land cover and road network information and SSP-prescribed estimates of rural and urban expansion^46^. The second indicator is the Global Gridded Relative Deprivation Index v.1 (GRDI; ref. ^16^), which communicates relative levels of multidimensional deprivation and poverty (0–100, least to most deprived). This deprivation score uses six input components. First is the child dependency ratio, which is the ratio between the population of children and the working-age population (15–64 years). This can indicate vulnerability, for which high ratios indicate a dependency of supposed consumers and non-producers on the working-age (producing) population^47^. Second, infant mortality rates (IMR), taken as the deaths in children younger than 1 year of age per 1,000 live births annually, are a signal of population health and form a long-term Sustainable Development Goal of the United Nations^48^. Third, the Subnational Human Development Index (SHDI), an assessment of human well-being across education, health and standard of living, originates from the Human Development Index, the latter of which is considered one of the most popular indices to assess country-level well-being. The SHDI improves on the HDI in terms of spatial scale and in representing 161 countries across all world regions and development levels^49^. Fourth, as rural populations are generally prone to multidimensional poverty^50^, low values in the ratio of built-up to non-built-up area (BUILT) signal high deprivation. The fifth and sixth components use the mean (of 2020; VNL 2020) and slope (2012–2020; VNL Slope) of nighttime light intensity, a proxy for human activity, economic output and infrastructure development^51^, to indicate deprivation for areas of low nighttime light intensity. These input components range from 30 arc seconds (roughly 1 km) resolution to subnational regions and are harmonized in an ArcGIS Fishnet feature class for aggregation onto a 0–100 range representing low to high deprivation. For the final aggregation, the IMR and SHDI components are given half the weight of the rest of the inputs, given their coarser resolution. The GRDI, therefore, encapsulates multiple dimensions through which generations face deprivation and therewith socioeconomic vulnerability to climate extremes. Although our approach does not explicitly account for actual or potential adaptation to climate change, this multidimensional approach to vulnerability provides relevant information on the current adaptation potential of local populations.

We preprocess GDP and GRDI products to enable their comparison with our ULE estimates across birth years. For GDP, similar to other datasets in our analysis, we extend the series to 2113 to accommodate the longest life expectancy of the 2020 birth cohort by copying the final year of the original dataset. We then use our ISIMIP population totals to compute GDP per capita at the grid scale. Using the GDP per capita metric, we calculate lifetime mean GDP per capita using our life expectancy information for the 1960–2020 birth cohorts. We refer to lifetime mean GDP per capita as simply GDP. For GRDI, we conservatively regrid the original grid cells of  about 1 km to the 0.5° ISIMIP grid. Although GRDI is a map composed of data spanning 2010–2020, we assume this to be representative of 2020, but nonetheless compare it with the 1960–2020 birth cohort range, similar to the rest of the analysis.

We then identify 20% quantile ranges (that is, (0–20], (20–40], … (80–100]) for the lifetime GDP of each birth year and for the singular GRDI map (assumed to align with 2020 population totals). To this end, we rank the vulnerability indicators and apply these ranks to our birth cohort totals on the same grid and for the matching year. For example, the ranks taken from the lifetime mean GDP of the 2020 birth cohort are aligned with the population totals of newborns in 2020. Finally, we bin the ranked vulnerability indicators by their associated population totals into five groups of nearly equal population (as it is not possible to achieve perfect bin sizes given the sums of grid-scale population totals). This groups the richest and poorest and least and most deprived into the aforementioned quantile ranges. The quantile range of each vulnerability indicator is then a map that can be used to mask the existing locations of ULE, such as birth years and all GMT pathways. With GRDI (Supplementary Fig. 11) and GDP (Supplementary Fig. 12), we compare the lowest and the highest 20% of each indicator by population.

## Online content

Any methods, additional references, Nature Portfolio reporting summaries, source data, extended data, supplementary information, acknowledgements, peer review information; details of author contributions and competing interests; and statements of data and code availability are available at 10.1038/s41586-025-08907-1.

## Supplementary information

Supplementary Information

## Data Availability

The data for this analysis originate from multiple sources and are hereby listed. Model inputs, raw impact model simulations and post-processed extremes (the latter as Derived Output Data) from ISIMIP2b, as well as GDP data, are accessible at the ISIMIP repository here (https://data.isimip.org). Cohort sizes are taken from the Wittgenstein Centre for Demography and Global Human Capital (https://dataexplorer.wittgensteincentre.org/wcde-v2). Life expectancy data come from the UN demographics data portal (https://population.un.org/dataportal/home?df=10750103-f8fa-4a7e-bb6a-b0f151970005). Global mean temperatures are extracted from the AR6 scenario explorer^45^ (10.5281/zenodo.7197970). GRDI is hosted on the NASA EARTHDATA platform (10.7927/3xxe-ap97). Maps in this analysis contain base map information made with Natural Earth (naturalearthdata.com).

## Code availability

The code for this analysis can be found at GitHub (https://github.com/VUB-HYDR/2025_Grant_etal_Nature/tree/main).
