# Supplementary material for: Global emergence of unprecedented lifetime exposure to climate extremes
Source: Nature. 2025 May 7;641(8062):374–9. doi: 10.1038/s41586-025-08907-1 (PMC12058528; doi:10.1038/s41586-025-08907-1)
Supplement: Supplementary file 1 — Supplementary Information [file 41586_2025_8907_MOESM1_ESM.pdf]

nature portfolio

https://doi.org/10.1038/s41586-025-08907-1

Supplementary information

Global emergence of unprecedented lifetime exposure to climate extremes

In the format provided by the authors and unedited

Supplementary Information - Global emergence of unprecedented exposure to climate extremes

Grant et al.

Contents

List of Tables **3**

List of Figures **5**

1 Supplementary Notes **110**

1.1 Supplementary Note 1: Sensitivity of results to ensemble sampling 110

1.2 Supplementary Note 2: Uncertainty in projections of hydroclimate extremes . . 110

1.3 Supplementary Note 3: Country scale CF for hydroclimate variables 112

Supplementary References **113**

List of Tables

1 Absolute population (in thousands) of cohorts living unprecedented exposure to heatwaves and $CF_{heatwaves}$ (%) per country and birth year in a 1.5°C pathway . . . 7
2 Absolute population (in thousands) of cohorts living unprecedented exposure to heatwaves and $CF_{heatwaves}$ (%) per country and birth year in a 2.5°C pathway . . . 13
3 Absolute population (in thousands) of cohorts living unprecedented exposure to heatwaves and $CF_{heatwaves}$ (%) per country and birth year in a 3.5°C pathway . . . 19
4 Absolute population (in thousands) of cohorts living unprecedented exposure to floods and CF_{floods} (%) per country and birth year in a 1.5°C pathway . . . 25
5 Absolute population (in thousands) of cohorts living unprecedented exposure to floods and CF_{floods} (%) per country and birth year in a 2.5°C pathway . . . 30
6 Absolute population (in thousands) of cohorts living unprecedented exposure to floods and CF_{floods} (%) per country and birth year in a 3.5°C pathway . . . 35
7 Absolute population (in thousands) of cohorts living unprecedented exposure to droughts and $CF_{droughts}$ (%) per country and birth year in a 1.5°C pathway . . . 40
8 Absolute population (in thousands) of cohorts living unprecedented exposure to droughts and $CF_{droughts}$ (%) per country and birth year in a 2.5°C pathway . . . 46
9 Absolute population (in thousands) of cohorts living unprecedented exposure to droughts and $CF_{droughts}$ (%) per country and birth year in a 3.5°C pathway . . . 52
10 Absolute population (in thousands) of cohorts living unprecedented exposure to crop failures and $CF_{crop\ failures}$ (%) per country and birth year in a 1.5°C pathway . . . 57
11 Absolute population (in thousands) of cohorts living unprecedented exposure to crop failures and $CF_{crop\ failures}$ (%) per country and birth year in a 2.5°C pathway . . . 62
12 Absolute population (in thousands) of cohorts living unprecedented exposure to crop failures and $CF_{crop\ failures}$ (%) per country and birth year in a 3.5°C pathway . . . 67
13 Absolute population (in thousands) of cohorts living unprecedented exposure to wildfires and $CF_{wildfires}$ (%) per country and birth year in a 1.5°C pathway . . . 72

14 **Absolute population (in thousands) of cohorts living unprecedented exposure to wildfires and $CF_{wildfires}$ (%) per country and birth year in a 2.5°C pathway** . . . 78
15 **Absolute population (in thousands) of cohorts living unprecedented exposure to wildfires and $CF_{wildfires}$ (%) per country and birth year in a 3.5°C pathway** . . . 83
16 **Absolute population (in thousands) of cohorts living unprecedented exposure to tropical cyclones and $CF_{tropical\ cyclones}$ (%) per country and birth year in a 1.5°C pathway** . . . 88
17 **Absolute population (in thousands) of cohorts living unprecedented exposure to tropical cyclones and $CF_{tropical\ cyclones}$ (%) per country and birth year in a 2.5°C pathway** . . . 91
18 **Absolute population (in thousands) of cohorts living unprecedented exposure to tropical cyclones and $CF_{tropical\ cyclones}$ (%) per country and birth year in a 3.5°C pathway** . . . 95
19 **Ensemble members per extreme event category and GMT pathway** . . . 98
20 **The thresholds and models used for defining extreme events. In cases where models are not listed as global climate models (for all extremes other than heatwaves), global climate models provide boundary conditions to the listed models.** . . . 99

List of Figures

1 **Heatmaps of multi-model mean CF for all extreme events when ensembles are limited to simulations valid for GMT pathways**. CF shown across all birth years (1960 to 2020) and GMT pathways (1.5 to 3.5 °C pathways) for wild fires ($CF_{wildfires}$; **a**), crop failures ($CF_{cropfailures}$; **b**), droughts ($CF_{droughts}$; **c**), river floods (CF_{floods}; **d**), heatwaves ($CF_{heatwaves}$; **e**) and tropical cyclones ($CF_{tropicalcyclones}$; **f**). Each extreme event panel has its own colorbar range in percents. 100

2 **Birth cohort fractions experiencing unprecedented levels of exposure to extremes in 1.5, 2.5 and 3.5 °C pathways**. Blue, gold and red box plots show CF for 1.5, 2.5 and 3.5 °C pathways and birth cohorts between 1960 and 2020 for wildfires (**a**), crop failures (**b**), droughts (**c**), river floods (**d**), heatwaves (**e**) and tropical cyclones (**f**). 100

3 **Birth cohort fractions experiencing unprecedented levels of exposure to extremes in 2.7 °C pathways**. Multi-model mean time series and range across projections for CF in 2.7 °C pathway and birth cohorts between 1960 and 2020 for wildfires (**a**), crop failures (**b**), droughts (**c**), river floods (**d**), heatwaves (**e**) and tropical cyclones (**f**). 101

4 **Maps of country level CF of the 2020 birth cohort for 1.5, 2.5 and 3.5 °C pathways for all extreme events**. CF is shown for wildfires (**a,g,m**), crop failures (**b,h,n**), droughts (**c,i,o**), river floods (**d,j,p**), heatwaves (**e,k,q**) and tropical cyclones (**f,l,r**) 102

5 **Locations of emergence shown as the fraction of projections reaching unprecedented exposure**. Fractions of projections with emergence for each extreme event for the 1960 birth cohort (**a-f**) and the 2020 birth cohort (**h-m**) in a 2.7 °C pathway, in line with the Climate Action Tracker current pledges estimate. Black hatching marks regions where emergence into unprecedented exposure occurs for $\geq$25% of the event category's ensemble of projections. . . . 103

6 **Locations where exposure to extreme events occurs**. Grid cells (brown) where exposure occurs for each extreme event in any projections in our ensemble at any time for wildfires (**a**), crop failures (**b**), droughts (**c**), river floods (**d**), heatwaves (**e**) and tropical cyclones (**f**). 104

7 **The poorest experience significantly more unprecedented exposure to heatwaves**. Geographic distribution of 2020 birth cohort belonging to the 20% lowest (**a**; brown markers) and 20% highest (**a**; green markers) in lifetime mean gross domestic product (GDP). Grid cell marker sizes and colors are scaled by their population. The birth cohort membership of these two groups projected to experience ULE to heatwaves under the current policies pathway of 2.7 °C warming by 2100 for every 5th birth year (**b**). Error bars show the standard deviation in projections. Asterisks imply that a given birth cohort and low- or high-vulnerability group has significantly more members with ULE to heatwaves than the alternative vulnerability group of the same birth cohort (at the 5% level). The low- (**c**) and high-GDP (**d**) group's membership that is projected to experience ULE under 1.5 and 3.5 °C pathways. Light colored bars show total cohort sizes per birth year and vulnerability class. 105

8 **Heatmaps of multi-model mean CF for all extreme events using country average exposure**. CF shown across all birth years (1960 to 2020) and GMT trajectories (1.5 to 3.5 °C pathways) for wild fires ($CF_{wildfires}$; **a**), crop failures ($CF_{cropfailures}$; **b**), droughts ($CF_{droughts}$; **c**), river floods (CF_{floods}; **d**), heatwaves ($CF_{heatwaves}$; **e**) and tropical cyclones ($CF_{tropicalcyclones}$; **f**). Each extreme event panel has its own colorbar range in percents. 106

9 **GMT pathways of RCP forcings and AR6 time series' used in mapping exposure to climate extremes**. GMT time series of RCPs 2.6, 6.0 and 8.5 smoothed with a 21-year rolling mean (**a**) and pathways generated for mapping exposure projections to specific warming targets (**b**). 106

10 **Mapping extreme event exposures from radiative forcing scenarios to GMT pathways**. Per year (GMT anomaly) of the GMT pathway considered for the lifetime exposure analysis (**b**; 2.5 °C), we find the closest GMT anomaly from the original exposure projections to select an annual map of exposed area (**a**; RCP 8.5). 107

11 **Geographic distribution of GRDI quantile ranges**. The lowest ranking in the GRDI depravity index globally for the 2020 birth cohort (0-20% or least deprived; **a**) to the highest ranking (80-100% or most deprived; **e**), as binned by population. Populations of each grid cell are communicated by color and marker size. 108

12 **Geographic distribution of lifetime mean GDP quantile ranges**. The lowest ranking in lifetime mean GDP globally for the 2020 birth cohort (0-20%; **a**) to the highest ranking (80-100%; **e**), as binned by population. Populations of each grid cell are communicated by color and marker size. 109

Supplementary Tables

Table 1: **Absolute population (in thousands) of cohorts living unprecedented exposure to heatwaves and $CF_{heatwaves}$ (%) per country and birth year in a 1.5°C pathway**

Country	1960	1970	1980	1990	2000	2010	2020
Afghanistan	53 (16)	152 (35)	215 (40)	267 (51)	499 (54)	624 (53)	699 (52)
Angola	21 (12)	52 (24)	97 (34)	145 (39)	237 (52)	327 (52)	342 (51)
Albania	14 (28)	27 (47)	31 (51)	36 (51)	26 (50)	18 (50)	16 (46)
United Arab Emirates	1 (81)	2 (92)	11 (98)	18 (98)	19 (98)	28 (98)	32 (99)
Argentina	85 (19)	116 (24)	209 (31)	219 (32)	226 (32)	240 (33)	238 (33)
Armenia	34 (54)	34 (59)	39 (60)	45 (60)	23 (60)	27 (61)	26 (61)
Australia	19 (9)	39 (17)	44 (21)	44 (19)	50 (21)	58 (22)	65 (22)
Austria	25 (21)	38 (30)	33 (37)	36 (40)	43 (50)	50 (62)	51 (61)
Azerbaijan	22 (20)	48 (39)	45 (42)	61 (42)	44 (42)	41 (42)	50 (47)
Burundi	17 (17)	29 (24)	68 (44)	110 (50)	128 (56)	173 (56)	207 (56)
Belgium	16 (10)	32 (20)	48 (35)	47 (35)	46 (35)	54 (36)	55 (36)
Benin	13 (19)	40 (43)	74 (61)	105 (65)	147 (71)	181 (70)	204 (67)
Burkina Faso	74 (48)	144 (71)	204 (76)	267 (76)	345 (76)	447 (76)	529 (76)
Bangladesh	15 (1)	49 (2)	290 (11)	494 (17)	499 (17)	488 (18)	595 (23)
Bulgaria	54 (41)	71 (55)	86 (64)	75 (68)	44 (68)	49 (68)	41 (64)
Bahamas	0 (60)	0 (60)	0 (67)	0 (71)	0 (71)	0 (69)	0 (73)
Bosnia and Herzegovina	32 (35)	37 (44)	40 (54)	37 (54)	25 (53)	19 (58)	19 (58)
Belarus	28 (14)	40 (27)	39 (26)	45 (28)	26 (27)	29 (28)	28 (28)
Belize	0 (5)	1 (36)	1 (47)	2 (52)	3 (59)	3 (58)	3 (58)
Bolivia	46 (42)	77 (56)	125 (72)	155 (77)	179 (78)	184 (78)	192 (81)
Brazil	591 (26)	920 (34)	1460 (48)	1645 (51)	1658 (53)	1386 (53)	1341 (53)
Brunei Darussalam	1 (49)	2 (61)	3 (62)	4 (74)	4 (74)	5 (74)	5 (62)
Bhutan	0 (0)	0 (0)	1 (4)	4 (20)	5 (26)	6 (33)	7 (36)
Botswana	5 (24)	14 (50)	21 (54)	25 (55)	27 (58)	26 (58)	25 (57)
Central African Republic	5 (13)	19 (36)	41 (59)	57 (63)	67 (64)	72 (62)	75 (61)
Canada	79 (19)	97 (27)	141 (42)	155 (43)	146 (44)	156 (45)	164 (43)
Switzerland	10 (13)	22 (26)	20 (34)	25 (38)	26 (38)	24 (38)	26 (37)
Chile	53 (26)	74 (32)	96 (45)	123 (50)	118 (51)	130 (59)	126 (61)
China	153 (1)	1691 (6)	2243 (11)	4282 (17)	3092 (20)	3530 (22)	3279 (24)

Cote dIvoire	45 (34)	102 (48)	187 (56)	271 (61)	365 (64)	437 (66)	460 (66)
Cameroon	46 (27)	94 (40)	164 (49)	253 (56)	315 (60)	407 (61)	409 (59)
Democratic Republic of the Congo	82 (15)	229 (33)	399 (43)	582 (47)	859 (53)	1204 (53)	1354 (51)
Republic of Congo	9 (20)	25 (29)	70 (53)	103 (57)	166 (60)	212 (70)	212 (70)
Colombia	260 (46)	429 (60)	495 (67)	572 (71)	566 (73)	551 (72)	569 (76)
Comoros	3 (100)	4 (100)	6 (100)	7 (100)	9 (100)	0 (0)	0 (0)
Cape Verde	1 (62)	1 (62)	1 (62)	2 (62)	1 (62)	0 (0)	0 (0)
Costa Rica	19 (45)	42 (84)	51 (92)	65 (92)	65 (93)	62 (93)	61 (93)
Cuba	95 (77)	139 (81)	93 (83)	104 (82)	87 (82)	71 (84)	64 (83)
Cyprus	2 (41)	1 (31)	2 (53)	2 (53)	2 (53)	2 (53)	2 (53)
Czech Republic	13 (10)	14 (12)	25 (16)	32 (29)	25 (32)	40 (37)	38 (37)
Germany	71 (6)	112 (9)	182 (23)	206 (23)	186 (23)	172 (24)	180 (24)
Djibouti	1 (46)	1 (78)	2 (84)	2 (82)	2 (81)	7 (78)	8 (78)
Denmark	2 (3)	2 (3)	5 (11)	11 (24)	12 (24)	12 (28)	12 (28)
Dominican Republic	68 (60)	85 (63)	97 (64)	108 (64)	126 (72)	132 (74)	128 (75)
Algeria	154 (39)	293 (59)	521 (78)	637 (84)	491 (84)	637 (86)	656 (84)
Ecuador	46 (29)	82 (39)	106 (43)	122 (43)	123 (42)	116 (39)	112 (38)
Egypt	215 (25)	436 (41)	613 (48)	808 (50)	692 (47)	817 (47)	921 (47)
Eritrea	22 (47)	48 (78)	65 (81)	98 (95)	102 (99)	158 (99)	167 (95)
Spain	110 (21)	213 (39)	253 (48)	172 (50)	159 (52)	239 (63)	208 (65)
Estonia	3 (18)	3 (19)	3 (16)	6 (37)	4 (39)	5 (34)	3 (23)
Ethiopia	303 (39)	536 (51)	767 (57)	1177 (65)	1618 (66)	1692 (64)	1862 (64)
Finland	11 (15)	8 (13)	9 (16)	15 (27)	15 (29)	14 (26)	13 (23)
Fiji	4 (80)	4 (82)	5 (89)	5 (89)	5 (89)	8 (89)	7 (89)
France	96 (13)	171 (22)	191 (28)	203 (29)	208 (30)	275 (38)	269 (38)
Gabon	4 (37)	8 (67)	11 (67)	15 (68)	16 (71)	23 (72)	22 (69)
United Kingdom	27 (3)	65 (8)	156 (25)	210 (30)	201 (31)	251 (36)	271 (37)
Georgia	25 (29)	42 (48)	43 (51)	47 (51)	28 (50)	29 (50)	26 (49)
Ghana	59 (27)	138 (46)	190 (52)	259 (56)	321 (57)	385 (57)	431 (57)
Guinea	35 (36)	77 (62)	105 (75)	154 (82)	230 (88)	256 (88)	274 (88)

The Gambia	2 (24)	3 (25)	4 (25)	10 (37)	18 (46)	22 (46)	23 (46)
Guinea-Bissau	6 (49)	8 (63)	13 (72)	16 (73)	19 (79)	23 (81)	25 (80)
Equatorial Guinea	2 (36)	5 (64)	5 (74)	8 (76)	12 (80)	10 (77)	11 (72)
Greece	32 (29)	43 (39)	57 (53)	47 (58)	46 (58)	47 (58)	39 (57)
Guatemala	96 (62)	130 (67)	208 (82)	237 (80)	280 (79)	295 (79)	329 (83)
Guyana	11 (58)	20 (98)	18 (100)	15 (100)	16 (100)	13 (100)	13 (100)
Hong Kong	0 (0)	0 (0)	9 (12)	17 (25)	20 (37)	0 (0)	0 (0)
Honduras	16 (23)	40 (42)	70 (56)	97 (63)	117 (66)	122 (70)	123 (75)
Croatia	14 (19)	18 (30)	23 (37)	21 (37)	23 (46)	22 (59)	20 (58)
Haiti	78 (68)	103 (74)	125 (74)	168 (78)	177 (78)	183 (79)	173 (79)
Hungary	44 (26)	60 (41)	77 (42)	60 (46)	60 (58)	58 (58)	60 (66)
Indonesia	394 (15)	742 (23)	1398 (37)	1535 (41)	1537 (43)	1619 (42)	1554 (44)
India	2024 (14)	4235 (25)	7120 (35)	10351 (44)	11689 (47)	11459 (47)	10491 (46)
Ireland	0 (0)	1 (1)	23 (18)	15 (19)	13 (19)	15 (27)	18 (34)
Iran	313 (41)	633 (67)	961 (73)	1428 (81)	997 (81)	993 (80)	964 (76)
Iraq	90 (36)	191 (48)	289 (58)	394 (64)	512 (64)	620 (62)	765 (67)
Iceland	1 (27)	1 (27)	1 (38)	1 (39)	1 (38)	2 (48)	2 (48)
Israel	14 (38)	17 (42)	35 (61)	40 (61)	53 (61)	73 (65)	79 (60)
Italy	201 (30)	251 (34)	262 (45)	208 (46)	194 (46)	237 (52)	205 (51)
Jamaica	39 (91)	49 (100)	43 (100)	44 (100)	43 (100)	37 (100)	38 (100)
Jordan	8 (26)	29 (48)	46 (59)	63 (60)	78 (60)	86 (60)	87 (60)
Japan	221 (17)	386 (26)	471 (33)	381 (34)	349 (36)	338 (39)	279 (36)
Kazakhstan	21 (7)	69 (21)	99 (28)	137 (36)	81 (35)	114 (35)	139 (42)
Kenya	193 (62)	319 (72)	504 (78)	684 (80)	826 (80)	989 (78)	1101 (82)
Kyrgyzstan	5 (7)	13 (15)	22 (22)	28 (22)	25 (22)	23 (17)	29 (20)
Cambodia	3 (2)	1 (0)	35 (19)	135 (36)	112 (34)	115 (33)	116 (34)
Kiribati	0 (0)	0 (0)	0 (0)	0 (0)	0 (0)	0 (0)	0 (0)
Republic of Korea	0 (0)	3 (0)	3 (0)	2 (0)	3 (1)	5 (2)	5 (2)
Kuwait	4 (76)	13 (88)	21 (100)	26 (100)	24 (100)	28 (100)	29 (100)
Lao PDR	11 (16)	19 (22)	41 (38)	67 (49)	69 (48)	58 (42)	58 (43)
Lebanon	3 (6)	16 (25)	17 (24)	31 (52)	33 (52)	24 (52)	29 (50)
Liberia	2 (6)	18 (44)	40 (73)	48 (84)	61 (87)	78 (86)	95 (83)
Libya	7 (17)	24 (33)	42 (42)	56 (50)	52 (51)	55 (50)	57 (48)
Sri Lanka	101 (42)	128 (48)	192 (69)	191 (74)	176 (75)	215 (75)	194 (76)
Lesotho	12 (46)	24 (73)	33 (77)	38 (77)	41 (77)	44 (77)	44 (73)
Lithuania	8 (14)	14 (26)	15 (27)	22 (37)	14 (36)	11 (35)	11 (31)

Luxembourg	0 (12)	0 (12)	1 (25)	1 (25)	1 (25)	1 (25)	1 (25)
Latvia	7 (22)	7 (25)	7 (24)	13 (35)	6 (34)	7 (33)	5 (27)
Morocco	135 (39)	221 (53)	307 (65)	353 (69)	316 (69)	373 (71)	411 (79)
Moldova	23 (29)	42 (65)	49 (69)	62 (76)	34 (77)	29 (77)	22 (64)
Madagascar	76 (44)	113 (50)	153 (51)	224 (59)	302 (59)	358 (58)	428 (61)
Mexico	608 (44)	1045 (57)	1267 (59)	1316 (61)	1340 (60)	1186 (56)	1336 (63)
Macedonia	4 (12)	16 (51)	16 (54)	16 (58)	13 (58)	12 (58)	10 (46)
Mali	86 (50)	169 (80)	222 (84)	280 (86)	366 (85)	508 (85)	589 (85)
Myanmar	45 (7)	162 (20)	276 (28)	358 (37)	354 (37)	315 (35)	278 (35)
Montenegro	2 (20)	3 (37)	3 (38)	3 (38)	2 (31)	3 (37)	2 (37)
Mongolia	0 (0)	5 (10)	9 (15)	10 (15)	8 (16)	9 (16)	11 (18)
Mozambique	49 (20)	111 (38)	178 (46)	208 (48)	279 (48)	344 (47)	367 (48)
Mauritania	24 (75)	35 (84)	48 (88)	60 (89)	74 (89)	91 (88)	97 (87)
Malawi	27 (23)	92 (56)	147 (63)	196 (59)	215 (54)	282 (54)	340 (53)
Malaysia	42 (19)	78 (32)	99 (35)	130 (37)	148 (39)	132 (38)	132 (33)
Namibia	8 (43)	15 (62)	23 (68)	29 (68)	34 (69)	36 (67)	40 (66)
New Caledonia	1 (72)	1 (75)	1 (84)	1 (84)	1 (84)	1 (75)	1 (75)
Niger	66 (48)	126 (69)	181 (76)	246 (78)	363 (81)	517 (80)	643 (77)
Nigeria	426 (29)	1007 (53)	1652 (62)	2351 (71)	3092 (75)	4041 (75)	4558 (72)
Nicaragua	39 (51)	59 (65)	106 (86)	132 (94)	131 (95)	123 (95)	116 (95)
Netherlands	20 (11)	39 (20)	48 (34)	51 (34)	53 (34)	52 (35)	52 (35)
Norway	4 (8)	10 (18)	10 (23)	14 (29)	15 (29)	11 (25)	11 (23)
Nepal	25 (6)	58 (12)	111 (18)	230 (30)	292 (34)	302 (38)	315 (40)
New Zealand	4 (8)	5 (10)	5 (11)	6 (11)	8 (15)	11 (19)	11 (19)
Oman	7 (50)	17 (81)	29 (82)	45 (89)	40 (91)	44 (91)	49 (93)
Pakistan	592 (40)	1018 (53)	1484 (58)	2079 (58)	2286 (59)	2462 (57)	2539 (55)
Panama	15 (51)	27 (74)	30 (75)	32 (77)	33 (73)	34 (72)	39 (82)
Peru	88 (30)	161 (44)	208 (49)	247 (54)	247 (54)	229 (53)	214 (52)
Philippines	171 (24)	268 (32)	428 (41)	632 (50)	723 (50)	708 (49)	827 (55)
Papua New Guinea	7 (12)	21 (32)	35 (40)	50 (49)	64 (50)	93 (59)	84 (53)
Poland	94 (13)	123 (24)	173 (28)	211 (36)	164 (40)	168 (43)	161 (42)
Puerto Rico	61 (100)	55 (100)	58 (100)	52 (100)	49 (100)	41 (100)	35 (100)
Dem. Rep. Korea	0 (0)	1 (0)	1 (0)	47 (15)	48 (15)	44 (17)	39 (17)
Portugal	12 (8)	32 (25)	31 (25)	31 (36)	44 (53)	45 (58)	43 (62)
Paraguay	19 (29)	33 (41)	43 (46)	54 (46)	68 (53)	57 (50)	53 (45)
Qatar	1 (75)	2 (87)	6 (99)	8 (100)	9 (100)	10 (100)	14 (100)

Romania	184 (50)	272 (69)	284 (71)	271 (78)	169 (76)	170 (78)	144 (76)
Russian Federation	249 (9)	263 (14)	381 (18)	481 (21)	287 (23)	401 (26)	407 (26)
Rwanda	23 (18)	35 (22)	94 (40)	148 (48)	158 (53)	207 (53)	266 (60)
Saudi Arabia	114 (87)	179 (93)	311 (96)	471 (97)	445 (97)	567 (97)	591 (96)
Sudan	205 (63)	352 (83)	505 (88)	640 (91)	809 (93)	1003 (93)	1130 (91)
Senegal	17 (15)	51 (33)	107 (51)	185 (69)	229 (70)	294 (70)	337 (72)
Solomon Islands	2 (97)	3 (100)	4 (99)	5 (100)	5 (100)	5 (100)	6 (100)
Sierra Leone	3 (5)	19 (24)	31 (31)	57 (47)	74 (57)	105 (64)	116 (66)
El Salvador	50 (51)	71 (57)	95 (66)	100 (70)	106 (73)	86 (73)	82 (73)
Somalia	42 (59)	79 (86)	148 (92)	183 (95)	227 (97)	276 (97)	303 (96)
Serbia	33 (30)	59 (57)	70 (62)	64 (63)	54 (62)	46 (63)	46 (65)
South Sudan	16 (17)	54 (43)	106 (64)	170 (77)	215 (77)	206 (76)	236 (76)
Sao Tome and Principe	2 (100)	3 (100)	4 (100)	4 (100)	4 (100)	0 (0)	0 (0)
Suriname	10 (85)	12 (99)	9 (99)	10 (100)	10 (100)	10 (100)	10 (100)
Slovakia	22 (22)	27 (33)	35 (35)	36 (41)	34 (56)	34 (56)	38 (61)
Slovenia	5 (20)	6 (27)	10 (38)	8 (38)	8 (51)	15 (61)	15 (61)
Sweden	12 (13)	18 (17)	18 (21)	27 (28)	21 (27)	19 (23)	20 (22)
Swaziland	2 (17)	7 (45)	12 (58)	17 (63)	17 (63)	18 (61)	15 (53)
Syria	16 (10)	66 (30)	131 (41)	211 (55)	237 (55)	265 (55)	245 (55)
Chad	39 (39)	78 (61)	124 (71)	192 (81)	291 (87)	379 (87)	408 (81)
Togo	12 (22)	36 (48)	64 (66)	83 (66)	107 (70)	130 (66)	121 (62)
Thailand	99 (11)	243 (20)	398 (34)	390 (38)	336 (37)	295 (37)	271 (38)
Tajikistan	7 (8)	5 (5)	11 (8)	15 (8)	15 (8)	14 (8)	21 (12)
Turkmenistan	12 (21)	24 (32)	40 (43)	50 (42)	40 (40)	48 (41)	58 (42)
Timor-Leste	5 (33)	8 (45)	8 (58)	17 (69)	24 (69)	23 (69)	27 (69)
Trinidad and Tobago	10 (50)	18 (94)	19 (94)	19 (94)	13 (94)	15 (94)	13 (94)
Tunisia	13 (10)	51 (33)	90 (49)	108 (55)	88 (59)	91 (59)	95 (57)
Turkey	139 (16)	518 (51)	682 (58)	762 (65)	760 (65)	736 (65)	679 (63)
Tanzania	101 (31)	208 (48)	336 (56)	465 (58)	680 (67)	990 (67)	1192 (69)
Uganda	71 (27)	163 (44)	291 (58)	472 (66)	614 (61)	803 (62)	1006 (62)
Ukraine	198 (25)	262 (39)	322 (45)	328 (49)	192 (46)	218 (47)	196 (46)
Uruguay	9 (18)	9 (18)	19 (35)	17 (33)	16 (31)	15 (30)	14 (30)

United States	1432 (36)	1739 (51)	2064 (64)	2437 (65)	2441 (65)	2585 (66)	2635 (66)
Uzbekistan	66 (23)	95 (25)	131 (28)	152 (24)	127 (23)	118 (22)	138 (25)
Saint Vincent and the Grenadines	2 (75)	3 (100)	3 (100)	2 (100)	2 (100)	0 (0)	0 (0)
Venezuela	182 (74)	260 (81)	353 (90)	441 (90)	456 (90)	458 (88)	459 (89)
United States Virgin Islands	1 (100)	1 (100)	1 (100)	1 (100)	1 (100)	0 (0)	0 (0)
Vietnam	129 (12)	243 (18)	521 (36)	670 (40)	529 (41)	531 (41)	515 (40)
Vanuatu	0 (73)	0 (99)	0 (100)	1 (100)	1 (100)	0 (100)	0 (100)
Samoa	1 (88)	1 (100)	1 (100)	1 (100)	1 (100)	0 (0)	0 (0)
Yemen	59 (36)	146 (66)	291 (91)	458 (93)	525 (89)	635 (90)	780 (90)
South Africa	169 (31)	329 (48)	485 (55)	559 (57)	552 (58)	572 (58)	522 (54)
Zambia	44 (38)	94 (59)	147 (66)	188 (66)	302 (81)	389 (83)	477 (82)
Zimbabwe	61 (41)	111 (55)	164 (57)	193 (55)	199 (54)	241 (59)	220 (58)

Table 2: **Absolute population (in thousands) of cohorts living unprecedented exposure to heatwaves and $CF_{heatwaves}$ (%) per country and birth year in a 2.5°C pathway**

Country	1960	1970	1980	1990	2000	2010	2020
Afghanistan	66 (20)	157 (36)	266 (50)	392 (75)	773 (83)	1019 (87)	1241 (93)
Angola	20 (11)	46 (21)	94 (33)	178 (48)	275 (61)	510 (81)	593 (88)
Albania	11 (23)	25 (43)	33 (54)	53 (73)	51 (100)	36 (100)	35 (100)
United Arab Emirates	1 (87)	2 (98)	11 (99)	18 (99)	19 (100)	28 (100)	32 (100)
Argentina	107 (24)	162 (33)	309 (46)	363 (53)	450 (65)	550 (75)	631 (88)
Armenia	27 (43)	43 (76)	51 (79)	60 (80)	32 (84)	38 (86)	42 (100)
Australia	17 (8)	21 (9)	33 (16)	64 (28)	98 (42)	128 (49)	158 (54)
Austria	7 (6)	21 (17)	29 (32)	59 (67)	65 (76)	73 (91)	76 (91)
Azerbaijan	18 (16)	48 (39)	62 (58)	91 (63)	82 (78)	83 (84)	90 (84)
Burundi	23 (22)	44 (35)	55 (36)	105 (47)	148 (65)	251 (82)	343 (93)
Belgium	4 (3)	38 (24)	85 (63)	108 (81)	125 (95)	149 (100)	153 (100)
Benin	11 (17)	41 (45)	81 (67)	121 (76)	164 (80)	217 (84)	270 (89)
Burkina Faso	68 (44)	125 (62)	183 (68)	243 (70)	325 (71)	452 (77)	577 (83)
Bangladesh	44 (3)	70 (3)	291 (11)	649 (22)	972 (32)	1042 (38)	1129 (43)
Bulgaria	36 (27)	59 (46)	76 (56)	77 (69)	58 (91)	67 (93)	61 (95)
Bahamas	0 (46)	0 (62)	0 (97)	0 (97)	0 (100)	0 (100)	0 (100)
Bosnia and Herzegovina	20 (22)	36 (43)	42 (57)	63 (92)	42 (90)	32 (95)	32 (95)
Belarus	24 (12)	49 (33)	25 (17)	54 (33)	34 (36)	44 (43)	49 (48)
Belize	0 (15)	1 (40)	2 (59)	3 (82)	4 (97)	5 (97)	5 (97)
Bolivia	51 (46)	82 (59)	128 (74)	183 (91)	218 (96)	230 (97)	232 (97)
Brazil	463 (21)	966 (36)	1537 (50)	2154 (67)	2226 (72)	1939 (74)	1937 (77)
Brunei Darussalam	2 (65)	2 (65)	3 (65)	4 (66)	4 (66)	6 (83)	6 (83)
Bhutan	0 (0)	0 (0)	2 (10)	4 (19)	5 (24)	7 (34)	6 (34)
Botswana	4 (20)	10 (35)	25 (63)	30 (66)	37 (79)	43 (96)	43 (96)
Central African Republic	6 (14)	17 (32)	41 (59)	62 (69)	85 (80)	103 (89)	114 (92)
Canada	56 (13)	119 (33)	144 (43)	193 (53)	233 (70)	256 (74)	286 (76)
Switzerland	6 (8)	14 (17)	36 (60)	55 (84)	65 (95)	62 (100)	70 (100)
Chile	61 (30)	108 (47)	128 (60)	175 (71)	180 (78)	177 (80)	178 (85)
China	93 (1)	2176 (8)	3339 (17)	7990 (31)	7264 (46)	9608 (60)	8963 (66)
Cote dIvoire	40 (30)	98 (46)	170 (51)	238 (53)	328 (57)	431 (65)	512 (73)

Cameroon	44 (26)	97 (41)	178 (54)	287 (64)	380 (72)	531 (79)	573 (82)
Democratic Republic of the Congo	85 (16)	204 (29)	427 (46)	712 (58)	1157 (71)	1858 (81)	2321 (87)
Republic of Congo	4 (10)	26 (31)	58 (44)	111 (62)	222 (80)	252 (83)	254 (84)
Colombia	273 (48)	476 (67)	594 (80)	678 (84)	654 (84)	698 (92)	712 (95)
Comoros	3 (100)	4 (100)	6 (100)	7 (100)	9 (100)	0 (0)	0 (0)
Cape Verde	1 (67)	1 (67)	1 (67)	2 (67)	2 (67)	0 (0)	0 (0)
Costa Rica	24 (56)	45 (91)	54 (99)	69 (99)	70 (100)	67 (100)	66 (100)
Cuba	85 (69)	140 (82)	101 (90)	119 (94)	103 (98)	83 (98)	77 (100)
Cyprus	2 (42)	2 (58)	2 (71)	3 (70)	2 (70)	3 (74)	4 (87)
Czech Republic	0 (0)	0 (0)	6 (4)	34 (30)	43 (54)	79 (73)	70 (67)
Germany	36 (3)	156 (13)	234 (29)	349 (39)	468 (58)	569 (81)	594 (80)
Djibouti	0 (27)	1 (67)	2 (97)	3 (100)	3 (100)	9 (93)	10 (100)
Denmark	2 (3)	8 (15)	13 (27)	13 (29)	18 (36)	20 (48)	20 (49)
Dominican Republic	73 (64)	98 (72)	112 (74)	136 (80)	167 (96)	175 (99)	170 (99)
Algeria	148 (38)	285 (58)	535 (80)	655 (87)	521 (89)	685 (93)	747 (96)
Ecuador	48 (30)	73 (35)	108 (44)	166 (59)	163 (56)	219 (74)	212 (73)
Egypt	308 (36)	483 (46)	652 (51)	889 (55)	1016 (69)	1346 (77)	1593 (81)
Eritrea	25 (54)	48 (79)	71 (88)	102 (100)	103 (100)	158 (100)	175 (100)
Spain	137 (26)	260 (48)	332 (63)	252 (73)	242 (79)	336 (88)	290 (90)
Estonia	3 (20)	4 (25)	3 (21)	4 (22)	2 (22)	7 (51)	6 (51)
Ethiopia	288 (37)	520 (50)	801 (60)	1301 (71)	2039 (84)	2280 (87)	2579 (89)
Finland	7 (10)	11 (17)	17 (29)	19 (34)	18 (35)	31 (56)	32 (56)
Fiji	3 (68)	4 (85)	6 (100)	6 (100)	6 (100)	9 (100)	8 (100)
France	79 (11)	218 (28)	387 (57)	510 (73)	587 (86)	702 (97)	698 (97)
Gabon	3 (30)	8 (64)	13 (74)	18 (81)	19 (88)	31 (95)	31 (97)
United Kingdom	14 (2)	118 (14)	187 (30)	327 (46)	356 (54)	506 (73)	579 (78)
Georgia	15 (17)	55 (62)	58 (70)	69 (76)	47 (84)	54 (93)	53 (100)
Ghana	46 (20)	136 (45)	212 (58)	291 (63)	366 (66)	453 (67)	542 (71)
Guinea	33 (34)	69 (55)	101 (72)	142 (76)	225 (86)	254 (87)	289 (93)
The Gambia	2 (27)	3 (24)	4 (21)	5 (18)	6 (17)	21 (45)	26 (53)
Guinea-Bissau	5 (43)	7 (61)	14 (78)	18 (82)	21 (87)	25 (90)	31 (97)

Equatorial Guinea	2 (36)	4 (59)	5 (74)	9 (87)	15 (94)	13 (96)	15 (96)
Greece	30 (27)	62 (57)	81 (76)	68 (84)	75 (96)	77 (96)	65 (96)
Guatemala	88 (56)	124 (64)	173 (68)	229 (77)	282 (80)	328 (88)	355 (90)
Guyana	11 (59)	17 (83)	18 (100)	15 (100)	16 (100)	13 (100)	13 (100)
Hong Kong	0 (0)	12 (17)	24 (33)	23 (33)	36 (67)	0 (0)	0 (0)
Honduras	14 (20)	46 (49)	86 (69)	112 (73)	143 (81)	168 (96)	164 (100)
Croatia	7 (10)	13 (22)	25 (40)	47 (83)	43 (87)	37 (96)	34 (96)
Haiti	83 (72)	104 (75)	137 (82)	174 (81)	187 (82)	191 (82)	187 (85)
Hungary	22 (13)	40 (27)	70 (39)	67 (52)	68 (66)	72 (71)	71 (78)
Indonesia	424 (16)	804 (24)	1342 (36)	1865 (49)	1904 (53)	2271 (59)	2270 (64)
India	1665 (12)	3791 (23)	7939 (39)	11774 (50)	13621 (55)	15670 (64)	15419 (68)
Ireland	1 (0)	15 (10)	21 (16)	28 (34)	33 (49)	34 (61)	27 (51)
Iran	318 (42)	653 (69)	1146 (86)	1720 (97)	1217 (99)	1231 (99)	1251 (99)
Iraq	84 (34)	263 (66)	400 (80)	595 (97)	782 (98)	974 (98)	1128 (98)
Iceland	1 (33)	1 (33)	1 (34)	1 (37)	2 (65)	2 (52)	2 (51)
Israel	15 (42)	29 (72)	55 (96)	65 (99)	87 (100)	112 (100)	131 (100)
Italy	143 (21)	314 (43)	410 (71)	370 (81)	380 (89)	430 (95)	386 (95)
Jamaica	42 (97)	49 (100)	43 (100)	44 (100)	43 (100)	37 (100)	38 (100)
Jordan	4 (14)	39 (65)	56 (72)	92 (87)	125 (96)	138 (96)	140 (96)
Japan	211 (16)	365 (24)	515 (36)	487 (44)	597 (61)	590 (67)	562 (73)
Kazakhstan	20 (6)	60 (19)	143 (41)	199 (52)	147 (64)	248 (76)	270 (81)
Kenya	190 (61)	342 (77)	563 (87)	770 (90)	974 (94)	1263 (99)	1344 (100)
Kyrgyzstan	3 (5)	13 (15)	38 (38)	59 (45)	55 (49)	79 (60)	106 (75)
Cambodia	4 (2)	1 (1)	59 (33)	198 (53)	200 (61)	229 (67)	238 (70)
Kiribati	0 (0)	0 (0)	0 (0)	0 (0)	0 (0)	0 (0)	0 (0)
Republic of Korea	0 (0)	1 (0)	11 (2)	58 (11)	139 (27)	98 (30)	93 (30)
Kuwait	5 (100)	14 (100)	21 (100)	26 (100)	24 (100)	28 (100)	29 (100)
Lao PDR	13 (19)	25 (29)	59 (56)	93 (67)	111 (76)	123 (89)	126 (95)
Lebanon	2 (3)	36 (57)	56 (82)	52 (86)	63 (100)	45 (100)	58 (100)
Liberia	1 (4)	15 (37)	37 (67)	40 (70)	53 (74)	68 (75)	87 (76)
Libya	5 (13)	25 (35)	41 (41)	69 (61)	74 (74)	93 (84)	105 (88)
Sri Lanka	89 (37)	130 (49)	185 (67)	216 (84)	214 (91)	266 (93)	236 (93)
Lesotho	12 (47)	17 (52)	28 (67)	33 (67)	36 (67)	39 (67)	42 (69)
Lithuania	6 (11)	12 (23)	7 (14)	10 (17)	16 (41)	15 (46)	16 (47)
Luxembourg	0 (0)	1 (17)	2 (50)	2 (67)	4 (83)	4 (100)	4 (100)
Latvia	8 (29)	9 (32)	4 (13)	6 (18)	6 (32)	11 (51)	10 (51)
Morocco	130 (38)	248 (60)	357 (76)	411 (80)	412 (90)	495 (94)	493 (95)

Moldova	8 (11)	29 (45)	44 (61)	61 (76)	36 (81)	33 (88)	34 (98)
Madagascar	63 (36)	97 (43)	188 (63)	295 (78)	463 (90)	579 (94)	690 (98)
Mexico	565 (41)	955 (52)	1374 (64)	1575 (73)	1699 (76)	1732 (82)	1711 (81)
Macedonia	5 (16)	15 (49)	19 (64)	23 (83)	22 (99)	21 (99)	21 (99)
Mali	81 (47)	151 (72)	212 (80)	282 (86)	383 (89)	550 (92)	664 (95)
Myanmar	37 (5)	162 (20)	392 (40)	495 (51)	552 (58)	581 (65)	566 (72)
Montenegro	2 (17)	3 (36)	5 (68)	6 (72)	7 (100)	7 (100)	6 (100)
Mongolia	0 (1)	2 (4)	6 (11)	18 (26)	18 (36)	33 (57)	46 (71)
Mozambique	56 (23)	100 (34)	211 (54)	285 (65)	451 (77)	649 (89)	733 (96)
Mauritania	22 (69)	34 (81)	49 (90)	64 (96)	80 (96)	101 (98)	109 (98)
Malawi	35 (29)	77 (46)	171 (73)	230 (69)	355 (89)	519 (99)	636 (100)
Malaysia	63 (29)	84 (34)	110 (38)	151 (43)	199 (52)	203 (58)	246 (62)
Namibia	6 (35)	13 (53)	22 (65)	29 (69)	37 (76)	53 (97)	59 (99)
New Caledonia	1 (80)	1 (96)	1 (100)	1 (100)	1 (100)	1 (100)	1 (100)
Niger	59 (44)	100 (55)	164 (69)	250 (79)	392 (87)	566 (88)	779 (94)
Nigeria	351 (24)	857 (45)	1632 (62)	2382 (72)	3390 (83)	4805 (90)	5883 (93)
Nicaragua	30 (40)	66 (72)	117 (94)	138 (98)	135 (98)	130 (100)	122 (100)
Netherlands	8 (4)	38 (20)	65 (46)	95 (63)	117 (74)	139 (91)	137 (91)
Norway	7 (13)	18 (33)	18 (40)	21 (44)	23 (46)	27 (59)	27 (56)
Nepal	7 (2)	32 (7)	120 (20)	309 (41)	351 (41)	481 (61)	497 (63)
New Zealand	6 (11)	9 (17)	13 (28)	27 (53)	33 (62)	37 (65)	37 (65)
Oman	8 (53)	18 (85)	32 (89)	47 (92)	42 (95)	47 (97)	52 (99)
Pakistan	569 (38)	1137 (59)	1827 (71)	2756 (77)	3098 (79)	3431 (80)	3722 (80)
Panama	18 (63)	26 (73)	33 (84)	38 (91)	42 (93)	46 (97)	47 (99)
Peru	77 (26)	157 (43)	220 (52)	277 (61)	290 (64)	326 (75)	324 (78)
Philippines	141 (20)	277 (33)	537 (52)	801 (63)	1045 (73)	1133 (79)	1229 (82)
Papua New Guinea	8 (14)	21 (33)	39 (45)	48 (48)	69 (54)	95 (60)	99 (62)
Poland	39 (5)	85 (17)	174 (28)	230 (40)	240 (60)	303 (77)	299 (79)
Puerto Rico	61 (100)	55 (100)	58 (100)	52 (100)	49 (100)	41 (100)	35 (100)
Dem. Rep. Korea	0 (0)	0 (0)	49 (19)	67 (22)	99 (32)	101 (38)	99 (42)
Portugal	16 (11)	35 (27)	58 (45)	59 (67)	63 (76)	59 (77)	52 (75)
Paraguay	21 (33)	32 (41)	54 (58)	98 (83)	122 (95)	109 (96)	113 (97)
Qatar	1 (83)	3 (99)	6 (100)	9 (100)	9 (100)	10 (100)	14 (100)
Romania	92 (25)	177 (45)	218 (55)	236 (68)	180 (81)	179 (82)	164 (87)
Russian Federation	180 (7)	215 (12)	380 (18)	679 (30)	483 (38)	743 (47)	855 (54)

Rwanda	19 (14)	54 (35)	98 (42)	154 (50)	171 (58)	327 (84)	375 (84)
Saudi Arabia	113 (87)	184 (96)	321 (99)	485 (100)	459 (100)	585 (100)	616 (100)
Sudan	172 (53)	336 (79)	508 (88)	667 (95)	834 (96)	1054 (97)	1221 (98)
Senegal	17 (14)	39 (25)	89 (42)	140 (52)	191 (59)	288 (69)	333 (71)
Solomon Islands	2 (96)	3 (100)	4 (100)	5 (100)	5 (100)	5 (100)	6 (100)
Sierra Leone	4 (5)	21 (26)	33 (34)	47 (39)	83 (64)	118 (72)	133 (75)
El Salvador	49 (51)	68 (55)	95 (66)	95 (67)	121 (83)	98 (83)	101 (90)
Somalia	45 (63)	79 (87)	153 (94)	191 (99)	234 (100)	284 (100)	315 (100)
Serbia	29 (26)	48 (47)	64 (57)	73 (72)	72 (83)	62 (85)	60 (85)
South Sudan	14 (15)	45 (36)	102 (61)	173 (78)	233 (83)	235 (87)	279 (89)
Sao Tome and Principe	2 (100)	3 (100)	4 (100)	4 (100)	4 (100)	0 (0)	0 (0)
Suriname	11 (96)	12 (98)	9 (100)	10 (100)	10 (100)	10 (100)	10 (100)
Slovakia	2 (2)	11 (14)	31 (31)	47 (54)	40 (66)	42 (70)	47 (76)
Slovenia	4 (15)	3 (15)	9 (34)	16 (80)	14 (89)	25 (98)	24 (98)
Sweden	15 (16)	36 (35)	39 (47)	50 (52)	44 (55)	50 (62)	56 (62)
Swaziland	1 (6)	4 (31)	9 (47)	13 (48)	13 (48)	20 (67)	22 (75)
Syria	10 (6)	104 (46)	190 (60)	236 (62)	328 (76)	378 (78)	381 (86)
Chad	42 (41)	66 (52)	133 (76)	211 (89)	313 (94)	422 (97)	486 (97)
Togo	11 (21)	38 (51)	64 (66)	98 (78)	122 (80)	165 (84)	169 (86)
Thailand	110 (12)	428 (35)	559 (47)	569 (55)	545 (60)	502 (63)	461 (65)
Tajikistan	9 (11)	9 (8)	34 (26)	98 (53)	123 (69)	143 (81)	175 (99)
Turkmenistan	11 (19)	42 (57)	66 (72)	104 (87)	98 (98)	117 (99)	139 (100)
Timor-Leste	5 (31)	9 (47)	10 (72)	21 (85)	33 (96)	32 (96)	38 (96)
Trinidad and Tobago	14 (67)	16 (84)	17 (84)	17 (85)	13 (92)	16 (100)	13 (100)
Tunisia	11 (8)	27 (17)	65 (36)	100 (52)	113 (75)	122 (79)	135 (80)
Turkey	197 (22)	542 (53)	854 (73)	990 (85)	1029 (88)	1056 (94)	1040 (96)
Tanzania	112 (35)	214 (49)	394 (65)	602 (76)	834 (82)	1386 (94)	1665 (97)
Uganda	66 (25)	188 (50)	345 (69)	529 (74)	806 (81)	1083 (83)	1361 (84)
Ukraine	82 (10)	206 (31)	267 (37)	386 (57)	270 (64)	327 (71)	339 (80)
Uruguay	11 (22)	12 (22)	13 (24)	25 (47)	38 (72)	38 (77)	31 (67)
United States	1485 (37)	1819 (53)	2067 (64)	2716 (73)	3243 (86)	3566 (91)	3613 (90)
Uzbekistan	70 (24)	168 (45)	302 (64)	464 (74)	453 (81)	459 (84)	491 (89)

Saint Vincent and the Grenadines	2 (83)	3 (100)	3 (100)	2 (100)	2 (100)	0 (0)	0 (0)
Venezuela	184 (75)	279 (87)	367 (93)	457 (94)	481 (95)	508 (98)	513 (99)
United States Virgin Islands	1 (100)	1 (100)	1 (100)	1 (100)	1 (100)	0 (0)	0 (0)
Vietnam	74 (7)	315 (24)	647 (45)	973 (59)	950 (73)	992 (77)	963 (75)
Vanuatu	0 (81)	0 (100)	0 (100)	1 (100)	1 (100)	0 (100)	0 (100)
Yemen	50 (30)	147 (67)	315 (99)	490 (100)	582 (98)	697 (98)	870 (100)
South Africa	131 (24)	254 (37)	463 (53)	585 (60)	644 (68)	772 (78)	810 (83)
Zambia	45 (39)	99 (62)	173 (78)	250 (87)	341 (92)	455 (97)	568 (98)
Zimbabwe	58 (39)	101 (49)	209 (72)	250 (71)	256 (69)	382 (93)	357 (95)

Table 3: **Absolute population (in thousands) of cohorts living unprecedented exposure to heatwaves and $CF_{heatwaves}$ (%) per country and birth year in a 3.5°C pathway**

Country	1960	1970	1980	1990	2000	2010	2020
Afghanistan	60 (18)	99 (23)	285 (53)	358 (69)	861 (93)	1167 (100)	1334 (100)
Angola	22 (12)	47 (22)	102 (36)	227 (61)	387 (85)	593 (94)	648 (97)
Albania	5 (10)	20 (34)	53 (85)	72 (100)	51 (100)	36 (100)	35 (100)
United Arab Emirates	1 (84)	2 (98)	11 (100)	18 (100)	19 (100)	28 (100)	32 (100)
Argentina	108 (24)	176 (36)	398 (60)	531 (78)	565 (81)	600 (82)	617 (86)
Armenia	16 (25)	28 (49)	44 (69)	59 (78)	37 (97)	44 (100)	42 (100)
Australia	20 (10)	41 (18)	54 (26)	83 (36)	131 (55)	181 (70)	220 (75)
Austria	4 (4)	20 (16)	67 (75)	81 (92)	86 (100)	80 (100)	84 (100)
Azerbaijan	25 (22)	55 (45)	64 (59)	113 (78)	93 (88)	91 (92)	107 (100)
Burundi	28 (27)	34 (27)	44 (28)	128 (57)	225 (99)	307 (100)	369 (100)
Belgium	2 (1)	3 (2)	93 (69)	129 (97)	126 (97)	149 (100)	153 (100)
Benin	15 (23)	53 (58)	90 (75)	129 (81)	191 (93)	251 (96)	304 (100)
Burkina Faso	101 (65)	147 (73)	207 (77)	293 (84)	414 (91)	547 (93)	653 (94)
Bangladesh	66 (4)	86 (4)	348 (14)	724 (24)	1243 (41)	1533 (56)	1849 (71)
Bulgaria	38 (29)	48 (38)	86 (64)	101 (91)	64 (100)	72 (100)	64 (100)
Bahamas	0 (40)	0 (79)	0 (95)	0 (100)	0 (100)	0 (100)	0 (100)
Bosnia and Herzegovina	27 (30)	31 (37)	58 (79)	69 (100)	47 (100)	34 (100)	33 (100)
Belarus	38 (19)	49 (33)	39 (25)	49 (31)	44 (47)	55 (53)	76 (74)
Belize	0 (9)	1 (21)	2 (73)	3 (81)	4 (91)	5 (100)	5 (100)
Bolivia	39 (35)	69 (50)	138 (79)	183 (91)	220 (96)	235 (99)	237 (100)
Brazil	659 (29)	1170 (44)	1824 (60)	2352 (73)	2451 (79)	2268 (87)	2303 (91)
Brunei Darussalam	1 (49)	2 (73)	3 (73)	4 (75)	4 (75)	5 (75)	8 (99)
Bhutan	0 (0)	0 (0)	2 (14)	6 (27)	11 (55)	14 (73)	14 (74)
Botswana	4 (21)	11 (41)	27 (70)	34 (76)	43 (93)	44 (100)	45 (100)
Central African Republic	7 (18)	22 (42)	49 (70)	72 (79)	92 (87)	109 (95)	122 (98)
Canada	74 (18)	109 (30)	135 (40)	226 (62)	276 (82)	330 (95)	370 (98)
Switzerland	6 (8)	43 (51)	53 (88)	65 (100)	68 (100)	62 (100)	70 (100)
Chile	49 (24)	100 (43)	142 (66)	188 (76)	206 (89)	210 (95)	198 (95)
China	163 (1)	2295 (9)	4752 (24)	11743 (46)	11033 (70)	12853 (81)	11736 (86)
Cote dIvoire	49 (37)	123 (58)	221 (66)	305 (68)	445 (78)	559 (84)	665 (95)

Cameroon	59 (34)	109 (46)	208 (63)	332 (74)	440 (83)	614 (92)	687 (99)
Democratic Republic of the Congo	78 (14)	179 (26)	403 (44)	895 (73)	1379 (85)	2155 (94)	2599 (97)
Republic of Congo	4 (8)	36 (42)	86 (65)	134 (75)	216 (77)	303 (100)	303 (100)
Colombia	368 (65)	601 (85)	703 (95)	799 (99)	771 (99)	757 (99)	749 (100)
Comoros	3 (100)	4 (100)	6 (100)	7 (100)	9 (100)	0 (0)	0 (0)
Cape Verde	1 (50)	1 (50)	1 (50)	3 (100)	2 (100)	0 (0)	0 (0)
Costa Rica	28 (65)	49 (100)	55 (100)	70 (100)	70 (100)	67 (100)	66 (100)
Cuba	90 (73)	152 (89)	106 (95)	125 (100)	106 (100)	85 (100)	77 (100)
Cyprus	0 (7)	2 (50)	2 (56)	3 (81)	3 (100)	4 (100)	4 (100)
Czech Republic	0 (0)	0 (0)	68 (44)	85 (75)	70 (88)	98 (90)	93 (90)
Germany	0 (0)	27 (2)	381 (47)	726 (82)	736 (92)	675 (96)	707 (96)
Djibouti	0 (11)	1 (81)	2 (95)	3 (100)	3 (100)	9 (100)	10 (100)
Denmark	1 (1)	10 (18)	15 (30)	24 (53)	35 (67)	35 (83)	41 (99)
Dominican Republic	77 (68)	107 (79)	134 (88)	165 (98)	173 (99)	178 (100)	171 (100)
Algeria	136 (35)	262 (53)	537 (81)	718 (95)	582 (99)	738 (100)	780 (100)
Ecuador	62 (39)	111 (54)	158 (64)	207 (73)	217 (74)	223 (75)	219 (75)
Egypt	183 (21)	389 (37)	614 (48)	1021 (63)	1202 (82)	1636 (94)	1909 (97)
Eritrea	23 (50)	44 (72)	75 (93)	102 (100)	103 (100)	159 (100)	175 (100)
Spain	153 (29)	331 (61)	398 (76)	315 (91)	298 (97)	377 (99)	318 (99)
Estonia	2 (13)	2 (10)	2 (11)	8 (49)	4 (40)	10 (75)	9 (75)
Ethiopia	260 (33)	486 (46)	845 (63)	1458 (80)	2167 (89)	2493 (95)	2864 (99)
Finland	15 (19)	16 (25)	23 (40)	38 (68)	38 (72)	42 (76)	44 (77)
Fiji	4 (77)	4 (90)	6 (100)	6 (100)	6 (100)	9 (100)	8 (100)
France	106 (14)	273 (35)	546 (80)	674 (96)	675 (99)	720 (100)	717 (100)
Gabon	3 (32)	7 (60)	14 (83)	20 (90)	21 (95)	32 (98)	32 (100)
United Kingdom	50 (7)	226 (26)	305 (49)	563 (79)	542 (82)	673 (97)	719 (97)
Georgia	10 (11)	38 (43)	54 (64)	79 (87)	51 (90)	58 (100)	53 (100)
Ghana	50 (22)	166 (55)	244 (67)	327 (71)	473 (85)	601 (89)	719 (94)
Guinea	40 (41)	86 (69)	110 (79)	163 (87)	257 (98)	286 (98)	304 (97)
The Gambia	2 (33)	2 (22)	4 (25)	7 (23)	24 (63)	38 (80)	40 (80)
Guinea-Bissau	7 (58)	8 (68)	13 (74)	16 (74)	24 (98)	28 (100)	32 (100)

Equatorial Guinea	3 (47)	5 (67)	5 (71)	10 (95)	15 (96)	13 (94)	15 (100)
Greece	21 (19)	59 (55)	93 (87)	78 (96)	78 (100)	80 (100)	68 (100)
Guatemala	88 (56)	139 (72)	227 (89)	278 (94)	354 (100)	373 (100)	397 (100)
Guyana	14 (72)	17 (84)	18 (100)	15 (100)	16 (100)	13 (100)	13 (100)
Hong Kong	0 (0)	19 (25)	37 (50)	52 (75)	54 (100)	0 (0)	0 (0)
Honduras	17 (24)	62 (65)	101 (81)	142 (92)	164 (93)	175 (100)	164 (100)
Croatia	11 (15)	13 (21)	50 (80)	56 (100)	49 (100)	38 (100)	35 (100)
Haiti	79 (68)	109 (79)	143 (85)	186 (87)	197 (87)	209 (90)	220 (100)
Hungary	20 (12)	45 (30)	126 (69)	100 (77)	84 (82)	99 (98)	91 (100)
Indonesia	421 (16)	989 (30)	1661 (44)	2087 (55)	2964 (82)	3587 (94)	3434 (96)
India	2007 (14)	5032 (30)	8586 (43)	13665 (58)	16967 (69)	19241 (79)	20326 (89)
Ireland	1 (1)	6 (4)	47 (36)	47 (58)	42 (63)	37 (67)	42 (80)
Iran	174 (23)	589 (63)	1024 (77)	1736 (98)	1226 (100)	1238 (100)	1260 (100)
Iraq	50 (20)	229 (58)	363 (72)	593 (97)	799 (100)	992 (100)	1149 (100)
Iceland	2 (50)	2 (50)	2 (54)	3 (73)	3 (75)	3 (75)	3 (75)
Israel	4 (10)	32 (77)	57 (100)	66 (100)	87 (100)	112 (100)	131 (100)
Italy	157 (23)	343 (47)	462 (80)	457 (100)	427 (100)	455 (100)	405 (100)
Jamaica	42 (96)	49 (100)	43 (100)	44 (100)	43 (100)	37 (100)	38 (100)
Jordan	4 (12)	28 (47)	61 (79)	94 (89)	131 (100)	143 (100)	145 (100)
Japan	171 (13)	327 (22)	577 (41)	844 (76)	903 (92)	823 (94)	734 (96)
Kazakhstan	13 (4)	48 (15)	148 (42)	262 (68)	180 (78)	311 (95)	335 (100)
Kenya	168 (54)	335 (76)	578 (90)	819 (96)	989 (96)	1270 (100)	1347 (100)
Kyrgyzstan	1 (2)	10 (11)	29 (29)	61 (46)	85 (76)	116 (88)	136 (96)
Cambodia	4 (2)	2 (1)	81 (45)	257 (68)	248 (76)	265 (77)	262 (77)
Kiribati	0 (0)	0 (0)	0 (0)	0 (0)	0 (0)	0 (0)	0 (0)
Republic of Korea	0 (0)	1 (0)	20 (3)	58 (11)	260 (50)	224 (68)	220 (70)
Kuwait	5 (100)	14 (100)	21 (100)	26 (100)	24 (100)	28 (100)	29 (100)
Lao PDR	15 (23)	32 (38)	64 (60)	111 (81)	126 (87)	127 (92)	127 (95)
Lebanon	0 (0)	22 (35)	54 (80)	59 (98)	63 (100)	45 (100)	58 (100)
Liberia	1 (5)	22 (53)	43 (79)	48 (83)	58 (83)	75 (83)	104 (91)
Libya	4 (9)	16 (22)	35 (35)	76 (68)	89 (89)	108 (98)	119 (100)
Sri Lanka	128 (53)	188 (71)	235 (85)	233 (91)	223 (95)	283 (99)	255 (100)
Lesotho	11 (41)	18 (53)	32 (75)	37 (75)	40 (75)	49 (85)	60 (100)
Lithuania	9 (15)	12 (22)	11 (21)	27 (46)	21 (55)	22 (68)	25 (73)
Luxembourg	0 (0)	0 (0)	3 (100)	4 (100)	4 (100)	4 (100)	4 (100)
Latvia	6 (20)	3 (12)	5 (17)	15 (43)	9 (50)	15 (68)	14 (72)
Morocco	111 (32)	289 (70)	386 (82)	464 (91)	443 (96)	521 (99)	517 (99)

Moldova	13 (16)	25 (38)	47 (65)	60 (75)	41 (91)	37 (100)	35 (100)
Madagascar	51 (29)	87 (39)	203 (68)	340 (90)	507 (99)	614 (100)	702 (100)
Mexico	520 (38)	1080 (59)	1599 (74)	1888 (87)	2032 (91)	1942 (91)	1956 (92)
Macedonia	7 (21)	14 (44)	22 (75)	27 (99)	22 (100)	21 (100)	22 (100)
Mali	99 (58)	168 (80)	226 (86)	298 (91)	425 (99)	600 (100)	697 (100)
Myanmar	51 (8)	283 (34)	541 (56)	644 (66)	730 (76)	734 (82)	704 (90)
Montenegro	1 (14)	3 (39)	8 (100)	8 (100)	7 (100)	7 (100)	6 (100)
Mongolia	0 (0)	0 (0)	3 (6)	7 (10)	23 (46)	44 (76)	54 (83)
Mozambique	45 (19)	83 (28)	160 (41)	312 (72)	525 (90)	724 (100)	764 (100)
Mauritania	23 (71)	37 (88)	51 (95)	66 (98)	82 (98)	103 (100)	112 (100)
Malawi	31 (26)	49 (30)	157 (67)	231 (70)	361 (91)	526 (100)	638 (100)
Malaysia	71 (33)	112 (45)	160 (56)	268 (76)	319 (83)	315 (90)	360 (91)
Namibia	6 (31)	12 (49)	28 (82)	32 (76)	46 (95)	54 (99)	60 (100)
New Caledonia	1 (69)	1 (100)	1 (100)	1 (100)	1 (100)	1 (100)	1 (100)
Niger	83 (61)	118 (65)	192 (80)	298 (95)	424 (94)	620 (96)	826 (99)
Nigeria	439 (30)	1025 (54)	1877 (71)	2853 (86)	3832 (93)	5291 (99)	6256 (99)
Nicaragua	29 (39)	68 (75)	120 (97)	141 (100)	138 (100)	130 (100)	122 (100)
Netherlands	8 (4)	10 (5)	77 (54)	132 (87)	149 (94)	150 (99)	148 (99)
Norway	7 (13)	20 (36)	24 (54)	37 (78)	41 (80)	40 (88)	43 (88)
Nepal	2 (0)	75 (16)	106 (18)	280 (37)	364 (43)	488 (62)	573 (73)
New Zealand	3 (5)	12 (22)	19 (40)	29 (58)	42 (81)	50 (86)	51 (90)
Oman	7 (44)	18 (85)	33 (92)	50 (98)	44 (100)	49 (100)	53 (100)
Pakistan	531 (36)	979 (51)	1692 (66)	2486 (70)	2912 (75)	3200 (75)	3579 (77)
Panama	17 (58)	30 (84)	39 (97)	41 (98)	45 (98)	48 (100)	47 (100)
Peru	75 (26)	153 (42)	247 (58)	317 (69)	359 (79)	376 (87)	371 (90)
Philippines	185 (26)	348 (42)	685 (66)	985 (77)	1284 (89)	1317 (92)	1402 (94)
Papua New Guinea	9 (17)	23 (35)	48 (55)	68 (67)	93 (72)	118 (74)	128 (80)
Poland	56 (8)	132 (26)	357 (57)	468 (81)	352 (87)	362 (92)	360 (95)
Puerto Rico	61 (100)	55 (100)	58 (100)	52 (100)	49 (100)	41 (100)	35 (100)
Dem. Rep. Korea	0 (0)	0 (0)	39 (15)	46 (15)	90 (29)	134 (51)	131 (55)
Portugal	8 (6)	43 (33)	71 (56)	57 (65)	71 (86)	70 (91)	63 (91)
Paraguay	28 (44)	57 (72)	86 (93)	117 (99)	129 (100)	113 (100)	117 (100)
Qatar	1 (75)	3 (100)	6 (100)	9 (100)	9 (100)	10 (100)	14 (100)
Romania	102 (28)	151 (38)	310 (78)	297 (85)	215 (97)	218 (100)	189 (100)
Russian Federation	158 (6)	213 (11)	487 (23)	804 (35)	545 (43)	960 (61)	1165 (73)

Rwanda	25 (20)	55 (35)	89 (38)	175 (57)	245 (83)	379 (98)	443 (100)
Saudi Arabia	106 (81)	182 (95)	322 (100)	486 (100)	460 (100)	585 (100)	617 (100)
Sudan	214 (66)	368 (87)	537 (93)	677 (96)	851 (98)	1074 (99)	1233 (99)
Senegal	18 (16)	48 (31)	90 (42)	168 (63)	230 (71)	336 (80)	437 (93)
Solomon Islands	2 (94)	3 (100)	4 (100)	5 (100)	5 (100)	5 (100)	6 (100)
Sierra Leone	5 (8)	27 (35)	54 (55)	92 (76)	108 (84)	153 (93)	178 (100)
El Salvador	60 (62)	91 (74)	108 (75)	142 (100)	145 (100)	117 (100)	111 (100)
Somalia	39 (55)	78 (86)	156 (97)	192 (99)	235 (100)	284 (100)	315 (100)
Serbia	17 (15)	31 (30)	93 (83)	92 (91)	87 (100)	73 (100)	71 (100)
South Sudan	23 (25)	67 (53)	119 (71)	186 (84)	247 (88)	256 (95)	302 (97)
Sao Tome and Principe	2 (100)	3 (100)	4 (100)	4 (100)	4 (100)	0 (0)	0 (0)
Suriname	11 (95)	12 (98)	9 (100)	10 (100)	10 (100)	10 (100)	10 (100)
Slovakia	3 (3)	23 (28)	71 (70)	73 (83)	59 (97)	60 (100)	61 (100)
Slovenia	4 (18)	5 (22)	22 (86)	20 (100)	15 (100)	25 (100)	24 (100)
Sweden	12 (13)	40 (39)	50 (59)	69 (73)	62 (78)	67 (82)	76 (84)
Swaziland	1 (9)	7 (45)	13 (63)	19 (67)	18 (66)	26 (87)	29 (100)
Syria	4 (3)	62 (28)	134 (42)	273 (71)	368 (86)	463 (96)	444 (100)
Chad	55 (55)	83 (65)	147 (84)	230 (96)	323 (97)	433 (99)	498 (99)
Togo	15 (29)	42 (56)	66 (68)	102 (81)	137 (89)	192 (98)	196 (100)
Thailand	195 (21)	630 (52)	790 (67)	759 (74)	678 (75)	610 (77)	557 (78)
Tajikistan	3 (4)	8 (7)	31 (24)	144 (78)	176 (100)	176 (100)	177 (100)
Turkmenistan	5 (9)	42 (57)	77 (84)	117 (98)	100 (100)	118 (100)	140 (100)
Timor-Leste	4 (26)	7 (37)	10 (71)	23 (97)	34 (100)	34 (100)	39 (100)
Trinidad and Tobago	10 (50)	14 (76)	15 (77)	20 (100)	14 (100)	16 (100)	13 (100)
Tunisia	11 (8)	27 (17)	67 (37)	127 (65)	135 (90)	154 (99)	167 (99)
Turkey	95 (11)	410 (40)	858 (73)	1018 (87)	1088 (93)	1127 (100)	1082 (100)
Tanzania	96 (30)	182 (42)	347 (57)	627 (79)	919 (90)	1458 (99)	1721 (100)
Uganda	92 (35)	231 (62)	356 (71)	586 (82)	854 (85)	1155 (89)	1528 (95)
Ukraine	84 (11)	164 (24)	343 (47)	409 (61)	295 (70)	399 (87)	394 (93)
Uruguay	16 (33)	17 (33)	22 (40)	37 (71)	38 (72)	35 (71)	33 (73)
United States	1421 (35)	1792 (52)	2221 (69)	3200 (86)	3545 (94)	3871 (98)	3976 (99)
Uzbekistan	31 (11)	153 (41)	363 (77)	522 (83)	527 (95)	528 (96)	553 (100)

Saint Vincent and the Grenadines	2 (75)	3 (100)	3 (100)	2 (100)	2 (100)	0 (0)	0 (0)
Venezuela	198 (81)	301 (93)	382 (97)	487 (100)	505 (100)	519 (100)	517 (100)
United States Virgin Islands	1 (100)	1 (100)	1 (100)	1 (100)	1 (100)	0 (0)	0 (0)
Vietnam	102 (9)	460 (34)	914 (63)	1340 (81)	1115 (86)	1150 (89)	1165 (91)
Vanuatu	0 (71)	0 (100)	0 (100)	1 (100)	1 (100)	0 (100)	0 (100)
Yemen	48 (29)	159 (72)	311 (98)	491 (100)	592 (100)	708 (100)	870 (100)
South Africa	134 (24)	278 (40)	592 (68)	761 (78)	782 (82)	883 (89)	920 (94)
Zambia	38 (33)	82 (51)	182 (82)	250 (87)	349 (94)	471 (100)	579 (100)
Zimbabwe	52 (35)	101 (50)	242 (84)	297 (85)	317 (86)	408 (100)	377 (100)

Table 4: **Absolute population (in thousands) of cohorts living unprecedented exposure to floods and $\mathbf{CF_{floods}}$ (%) per country and birth year in a 1.5°C pathway**

Country	1960	1970	1980	1990	2000	2010	2020
Afghanistan	1 (0)	4 (1)	12 (2)	29 (6)	68 (7)	106 (9)	137 (10)
Angola	2 (1)	5 (2)	12 (4)	21 (6)	41 (9)	68 (11)	69 (10)
Albania	0 (1)	0 (1)	0 (0)	2 (2)	1 (3)	1 (3)	1 (3)
United Arab Emirates	0 (0)	0 (0)	0 (0)	0 (0)	0 (0)	0 (0)	0 (0)
Argentina	7 (2)	27 (5)	46 (7)	58 (9)	66 (10)	73 (10)	86 (12)
Armenia	0 (0)	1 (1)	1 (1)	2 (2)	1 (2)	1 (3)	1 (2)
Australia	1 (0)	3 (1)	8 (4)	9 (4)	10 (4)	17 (7)	20 (7)
Austria	0 (0)	1 (0)	2 (2)	4 (4)	3 (4)	4 (5)	5 (6)
Azerbaijan	0 (0)	0 (0)	1 (1)	2 (2)	3 (3)	3 (3)	4 (3)
Burundi	0 (0)	2 (1)	2 (2)	6 (2)	37 (16)	55 (18)	66 (18)
Belgium	10 (6)	3 (2)	3 (2)	6 (5)	6 (4)	6 (4)	6 (4)
Benin	3 (4)	4 (4)	5 (4)	7 (4)	10 (5)	16 (6)	21 (7)
Burkina Faso	3 (2)	4 (2)	8 (3)	12 (3)	16 (3)	21 (4)	24 (3)
Bangladesh	68 (4)	156 (7)	173 (7)	237 (8)	276 (9)	223 (8)	177 (7)
Bulgaria	0 (0)	0 (0)	0 (0)	1 (1)	1 (1)	1 (2)	1 (2)
Bosnia and Herzegovina	2 (2)	2 (2)	1 (2)	1 (1)	0 (1)	0 (0)	0 (0)
Belarus	4 (2)	3 (2)	3 (2)	1 (1)	1 (1)	1 (1)	1 (1)
Belize	0 (0)	0 (0)	0 (0)	0 (1)	0 (1)	0 (1)	0 (1)
Bolivia	1 (1)	3 (2)	8 (5)	16 (8)	29 (13)	31 (13)	33 (14)
Brazil	37 (2)	83 (3)	124 (4)	168 (5)	197 (6)	172 (7)	207 (8)
Bhutan	0 (2)	0 (3)	1 (3)	2 (10)	3 (13)	3 (13)	2 (13)
Botswana	0 (0)	0 (0)	0 (0)	0 (1)	0 (1)	0 (1)	1 (1)
Central African Republic	0 (0)	1 (1)	3 (4)	3 (4)	5 (5)	4 (4)	5 (4)
Canada	8 (2)	9 (3)	14 (4)	18 (5)	18 (6)	20 (6)	25 (7)
Switzerland	1 (1)	1 (1)	1 (2)	5 (8)	5 (8)	5 (8)	7 (10)
Chile	0 (0)	1 (0)	1 (0)	1 (0)	10 (4)	10 (4)	10 (5)
China	68 (0)	356 (1)	507 (3)	983 (4)	737 (5)	931 (6)	1010 (7)
Cote dIvoire	0 (0)	1 (0)	1 (0)	8 (2)	18 (3)	43 (6)	65 (9)
Cameroon	2 (1)	6 (2)	10 (3)	12 (3)	28 (5)	46 (7)	51 (7)

Democratic Republic of the Congo	2 (0)	7 (1)	20 (2)	47 (4)	111 (7)	170 (7)	202 (8)
Republic of Congo	0 (0)	0 (0)	1 (1)	4 (2)	11 (4)	12 (4)	12 (4)
Colombia	21 (4)	36 (5)	52 (7)	90 (11)	91 (12)	95 (12)	98 (13)
Costa Rica	3 (7)	5 (9)	6 (11)	3 (4)	2 (4)	2 (3)	2 (3)
Cuba	0 (0)	1 (1)	1 (1)	4 (3)	4 (4)	3 (4)	3 (4)
Czech Republic	1 (1)	2 (1)	2 (1)	6 (5)	4 (5)	6 (5)	5 (5)
Germany	44 (4)	33 (3)	21 (3)	37 (4)	35 (4)	27 (4)	34 (5)
Denmark	0 (0)	0 (0)	0 (0)	0 (1)	1 (1)	1 (1)	1 (1)
Dominican Republic	0 (0)	1 (1)	2 (1)	3 (2)	3 (2)	4 (2)	7 (4)
Algeria	1 (0)	1 (0)	3 (1)	4 (1)	3 (0)	3 (0)	9 (1)
Ecuador	5 (3)	9 (4)	29 (12)	54 (19)	70 (24)	75 (25)	80 (28)
Egypt	68 (8)	106 (10)	123 (10)	242 (15)	211 (14)	271 (16)	303 (15)
Eritrea	0 (0)	0 (0)	0 (0)	3 (3)	12 (12)	21 (13)	24 (14)
Spain	5 (1)	8 (2)	11 (2)	7 (2)	8 (3)	14 (4)	15 (5)
Estonia	0 (1)	0 (3)	1 (3)	1 (5)	1 (6)	1 (6)	1 (6)
Ethiopia	25 (3)	47 (4)	79 (6)	166 (9)	342 (14)	407 (15)	478 (17)
Finland	2 (2)	2 (2)	2 (3)	2 (3)	2 (3)	2 (4)	2 (4)
Fiji	0 (1)	0 (1)	0 (1)	0 (3)	0 (3)	0 (3)	0 (4)
France	27 (4)	27 (4)	41 (6)	57 (8)	59 (9)	68 (9)	70 (10)
Gabon	0 (1)	0 (2)	0 (3)	1 (3)	1 (5)	2 (5)	2 (7)
United Kingdom	8 (1)	12 (1)	20 (3)	45 (6)	43 (6)	46 (7)	48 (6)
Georgia	1 (1)	1 (1)	2 (2)	3 (3)	2 (3)	2 (4)	2 (4)
Ghana	3 (1)	3 (1)	6 (2)	10 (2)	16 (3)	23 (3)	30 (4)
Guinea	0 (0)	1 (1)	2 (1)	5 (3)	7 (3)	19 (7)	23 (7)
The Gambia	0 (0)	0 (0)	0 (0)	0 (1)	0 (1)	1 (2)	1 (2)
Guinea-Bissau	0 (0)	0 (0)	0 (0)	0 (0)	0 (1)	0 (1)	0 (1)
Equatorial Guinea	0 (0)	0 (1)	0 (3)	0 (3)	1 (4)	1 (8)	1 (8)
Greece	0 (0)	1 (1)	2 (2)	1 (1)	1 (1)	2 (3)	2 (3)
Guatemala	4 (2)	5 (3)	6 (2)	7 (2)	10 (3)	9 (2)	8 (2)
Guyana	1 (4)	1 (4)	1 (5)	0 (2)	0 (1)	0 (2)	0 (2)
Honduras	1 (2)	2 (2)	3 (2)	4 (2)	4 (2)	4 (2)	4 (2)

Croatia	0 (0)	0 (0)	0 (1)	2 (4)	2 (4)	2 (4)	2 (4)
Haiti	0 (0)	0 (0)	0 (0)	1 (0)	8 (3)	12 (5)	12 (5)
Hungary	3 (2)	2 (2)	2 (1)	3 (2)	3 (2)	3 (3)	5 (5)
Indonesia	7 (0)	46 (1)	143 (4)	138 (4)	136 (4)	174 (5)	253 (7)
India	155 (1)	434 (3)	890 (4)	1970 (8)	2309 (9)	2444 (10)	2513 (11)
Ireland	27 (19)	28 (18)	20 (16)	13 (16)	11 (16)	8 (14)	6 (11)
Iran	1 (0)	5 (1)	8 (1)	29 (2)	23 (2)	30 (2)	42 (3)
Iraq	0 (0)	0 (0)	1 (0)	6 (1)	9 (1)	14 (1)	19 (2)
Iceland	0 (0)	0 (0)	0 (0)	0 (0)	0 (0)	0 (0)	0 (2)
Israel	0 (0)	0 (1)	0 (1)	1 (1)	1 (1)	1 (1)	1 (1)
Italy	0 (0)	15 (2)	15 (3)	14 (3)	14 (3)	18 (4)	19 (5)
Japan	5 (0)	15 (1)	18 (1)	17 (2)	19 (2)	18 (2)	14 (2)
Kazakhstan	9 (3)	19 (6)	27 (8)	37 (10)	27 (12)	47 (14)	49 (15)
Kenya	5 (2)	8 (2)	3 (0)	18 (2)	35 (3)	51 (4)	73 (5)
Kyrgyzstan	1 (1)	2 (2)	5 (5)	10 (8)	10 (9)	16 (12)	17 (12)
Cambodia	0 (0)	0 (0)	14 (8)	40 (11)	39 (12)	50 (15)	60 (18)
Republic of Korea	0 (0)	13 (2)	8 (1)	20 (4)	23 (4)	15 (4)	14 (4)
Lao PDR	0 (0)	0 (0)	7 (6)	19 (14)	27 (19)	30 (22)	32 (24)
Lebanon	1 (1)	1 (1)	1 (1)	1 (2)	1 (2)	1 (2)	1 (2)
Liberia	0 (0)	0 (0)	0 (1)	2 (3)	4 (5)	10 (11)	17 (15)
Libya	0 (0)	1 (1)	2 (2)	5 (4)	4 (4)	5 (4)	5 (4)
Sri Lanka	60 (25)	67 (25)	72 (26)	5 (2)	3 (1)	5 (2)	11 (4)
Lesotho	0 (1)	0 (1)	1 (2)	1 (2)	1 (2)	1 (1)	1 (1)
Lithuania	0 (1)	0 (0)	0 (0)	0 (0)	0 (0)	0 (0)	0 (0)
Luxembourg	0 (4)	0 (0)	0 (0)	0 (0)	0 (0)	0 (0)	0 (2)
Latvia	0 (0)	0 (1)	0 (1)	1 (3)	0 (3)	1 (3)	1 (4)
Morocco	0 (0)	1 (0)	2 (0)	9 (2)	9 (2)	10 (2)	11 (2)
Moldova	2 (3)	2 (2)	1 (2)	1 (1)	0 (0)	0 (0)	0 (1)
Madagascar	1 (0)	2 (1)	11 (4)	22 (6)	35 (7)	45 (7)	53 (8)
Mexico	10 (1)	56 (3)	114 (5)	113 (5)	116 (5)	90 (4)	131 (6)
Macedonia	0 (0)	0 (0)	0 (0)	0 (2)	0 (2)	0 (2)	1 (3)
Mali	0 (0)	0 (0)	7 (3)	9 (3)	14 (3)	22 (4)	27 (4)
Myanmar	19 (3)	49 (6)	85 (9)	109 (11)	140 (15)	131 (15)	145 (18)
Montenegro	0 (5)	0 (5)	0 (5)	0 (5)	0 (3)	0 (1)	0 (0)
Mongolia	0 (1)	1 (2)	2 (4)	3 (5)	3 (5)	4 (7)	5 (9)
Mozambique	5 (2)	10 (3)	13 (3)	21 (5)	33 (6)	38 (5)	43 (6)
Mauritania	0 (0)	0 (1)	1 (1)	1 (1)	1 (1)	2 (2)	2 (1)
Malawi	3 (2)	4 (3)	6 (3)	9 (3)	11 (3)	9 (2)	12 (2)
Malaysia	14 (6)	24 (10)	25 (9)	23 (7)	19 (5)	17 (5)	22 (6)

Namibia	0 (0)	0 (0)	0 (1)	1 (1)	1 (1)	1 (2)	1 (2)
New Caledonia	0 (1)	0 (1)	0 (3)	0 (2)	0 (2)	0 (1)	0 (1)
Niger	1 (1)	2 (1)	8 (3)	13 (4)	20 (4)	28 (4)	38 (5)
Nigeria	23 (2)	56 (3)	139 (5)	217 (7)	300 (7)	388 (7)	667 (11)
Nicaragua	9 (12)	12 (13)	16 (13)	4 (3)	3 (2)	3 (2)	2 (2)
Netherlands	14 (7)	10 (5)	8 (6)	10 (7)	9 (6)	9 (6)	8 (5)
Norway	1 (3)	1 (3)	2 (5)	4 (9)	5 (10)	5 (10)	6 (11)
Nepal	1 (0)	7 (1)	14 (2)	51 (7)	77 (9)	80 (10)	87 (11)
New Zealand	0 (1)	1 (2)	2 (3)	2 (4)	2 (5)	3 (5)	3 (6)
Oman	0 (0)	0 (0)	0 (0)	0 (0)	0 (0)	0 (1)	0 (1)
Pakistan	12 (1)	29 (2)	111 (4)	196 (5)	229 (6)	353 (8)	380 (8)
Panama	4 (14)	6 (18)	8 (20)	3 (8)	3 (7)	4 (8)	4 (9)
Peru	16 (5)	30 (8)	65 (15)	84 (18)	97 (21)	100 (23)	102 (25)
Philippines	2 (0)	16 (2)	24 (2)	33 (3)	47 (3)	56 (4)	99 (7)
Papua New Guinea	1 (1)	1 (2)	2 (2)	5 (5)	12 (9)	15 (10)	16 (10)
Poland	22 (3)	15 (3)	20 (3)	18 (3)	12 (3)	10 (3)	19 (5)
Puerto Rico	1 (1)	0 (0)	0 (0)	4 (7)	3 (7)	3 (7)	2 (7)
Dem. Rep. Korea	0 (0)	5 (1)	5 (2)	9 (3)	17 (5)	18 (7)	19 (8)
Portugal	0 (0)	0 (0)	4 (3)	5 (6)	5 (6)	5 (7)	5 (7)
Paraguay	0 (1)	1 (1)	10 (11)	20 (17)	28 (22)	26 (23)	31 (26)
Romania	6 (2)	6 (2)	4 (1)	3 (1)	2 (1)	2 (1)	5 (2)
Russian Federation	126 (5)	117 (6)	123 (6)	124 (5)	72 (6)	99 (6)	124 (8)
Rwanda	0 (0)	2 (1)	1 (0)	2 (1)	32 (11)	51 (13)	58 (13)
Saudi Arabia	0 (0)	0 (0)	0 (0)	1 (0)	1 (0)	1 (0)	1 (0)
Sudan	29 (9)	43 (10)	58 (10)	53 (7)	87 (10)	118 (11)	130 (10)
Senegal	1 (0)	0 (0)	0 (0)	3 (1)	6 (2)	9 (2)	10 (2)
Sierra Leone	0 (0)	2 (2)	4 (4)	6 (5)	8 (6)	18 (11)	20 (11)
El Salvador	1 (1)	2 (1)	2 (2)	4 (3)	5 (4)	4 (4)	3 (3)
Somalia	0 (1)	2 (2)	4 (2)	9 (5)	14 (6)	23 (8)	26 (8)
Serbia	3 (3)	2 (2)	2 (1)	1 (1)	1 (2)	1 (1)	1 (1)
South Sudan	4 (4)	6 (5)	8 (5)	11 (5)	18 (6)	20 (8)	26 (8)

Suriname	0 (0)	0 (0)	1 (10)	1 (10)	1 (10)	1 (11)	1 (11)
Slovakia	4 (4)	3 (3)	3 (3)	6 (6)	4 (7)	4 (7)	6 (10)
Slovenia	0 (1)	0 (1)	0 (0)	0 (2)	0 (2)	1 (2)	1 (3)
Sweden	1 (1)	2 (2)	3 (4)	8 (8)	7 (8)	6 (8)	7 (8)
Swaziland	0 (1)	0 (3)	1 (3)	0 (2)	0 (2)	1 (3)	3 (10)
Syria	0 (0)	0 (0)	0 (0)	1 (0)	1 (0)	1 (0)	0 (0)
Chad	1 (1)	3 (2)	12 (7)	18 (7)	26 (8)	26 (6)	28 (6)
Togo	3 (5)	3 (5)	6 (6)	10 (8)	12 (8)	15 (7)	19 (10)
Thailand	13 (1)	49 (4)	95 (8)	140 (14)	144 (16)	140 (18)	126 (18)
Tajikistan	1 (1)	2 (2)	6 (5)	20 (11)	27 (15)	34 (19)	35 (20)
Turkmenistan	2 (3)	2 (3)	4 (5)	9 (8)	11 (11)	14 (12)	16 (11)
Tunisia	0 (0)	0 (0)	0 (0)	6 (3)	5 (3)	5 (3)	6 (3)
Turkey	3 (0)	5 (0)	15 (1)	28 (2)	31 (3)	34 (3)	40 (4)
Tanzania	6 (2)	17 (4)	16 (3)	24 (3)	42 (4)	92 (6)	105 (6)
Uganda	19 (7)	25 (7)	17 (3)	27 (4)	55 (5)	88 (7)	111 (7)
Ukraine	31 (4)	26 (4)	21 (3)	7 (1)	5 (1)	5 (1)	10 (2)
Uruguay	0 (0)	0 (0)	10 (18)	10 (19)	10 (20)	10 (20)	10 (21)
United States	58 (1)	74 (2)	111 (3)	171 (5)	197 (5)	224 (6)	247 (6)
Uzbekistan	9 (3)	18 (5)	47 (10)	84 (13)	91 (16)	104 (19)	102 (18)
Venezuela	33 (14)	46 (14)	60 (15)	50 (10)	48 (9)	51 (10)	49 (10)
Vietnam	3 (0)	36 (3)	74 (5)	97 (6)	83 (6)	96 (7)	112 (9)
Yemen	0 (0)	0 (0)	0 (0)	1 (0)	2 (0)	2 (0)	5 (1)
South Africa	1 (0)	4 (1)	9 (1)	10 (1)	20 (2)	29 (3)	30 (3)
Zambia	0 (0)	0 (0)	1 (0)	3 (1)	8 (2)	17 (4)	33 (6)
Zimbabwe	1 (1)	2 (1)	6 (2)	8 (2)	11 (3)	12 (3)	13 (3)

Table 5: **Absolute population (in thousands) of cohorts living unprecedented exposure to floods and CF_{floods} (%) per country and birth year in a 2.5°C pathway**

Country	1960	1970	1980	1990	2000	2010	2020
Afghanistan	1 (0)	2 (0)	9 (2)	12 (2)	42 (5)	75 (6)	150 (11)
Angola	2 (1)	2 (1)	5 (2)	7 (2)	18 (4)	53 (8)	69 (10)
Albania	0 (0)	0 (0)	0 (0)	0 (0)	0 (0)	0 (0)	0 (0)
Argentina	6 (1)	10 (2)	27 (4)	53 (8)	104 (15)	121 (17)	119 (17)
Armenia	0 (0)	0 (0)	0 (0)	0 (0)	0 (0)	0 (0)	1 (2)
Australia	1 (1)	4 (2)	4 (2)	5 (2)	4 (2)	6 (2)	8 (3)
Azerbaijan	0 (0)	0 (0)	0 (0)	0 (0)	0 (0)	0 (0)	1 (1)
Burundi	0 (0)	1 (1)	1 (0)	7 (3)	12 (5)	18 (6)	31 (8)
Belgium	8 (5)	2 (1)	4 (3)	3 (2)	3 (3)	13 (9)	13 (8)
Benin	3 (5)	5 (5)	5 (4)	8 (5)	18 (9)	35 (13)	44 (15)
Burkina Faso	3 (2)	3 (2)	5 (2)	6 (2)	8 (2)	12 (2)	17 (2)
Bangladesh	62 (4)	106 (5)	171 (7)	235 (8)	278 (9)	227 (8)	301 (12)
Bosnia and Herzegovina	1 (1)	1 (1)	1 (1)	1 (1)	1 (2)	0 (1)	0 (1)
Belarus	4 (2)	3 (2)	3 (2)	4 (2)	2 (2)	2 (2)	7 (6)
Bolivia	1 (1)	3 (3)	8 (4)	24 (12)	35 (15)	47 (20)	60 (25)
Brazil	30 (1)	38 (1)	48 (2)	113 (4)	176 (6)	227 (9)	267 (11)
Bhutan	0 (2)	0 (3)	1 (4)	3 (13)	3 (17)	3 (15)	4 (20)
Botswana	0 (0)	0 (0)	0 (1)	0 (1)	0 (1)	0 (1)	0 (1)
Central African Republic	0 (0)	1 (1)	2 (2)	4 (4)	5 (5)	9 (8)	22 (18)
Canada	6 (2)	9 (3)	16 (5)	21 (6)	25 (7)	31 (9)	36 (10)
Switzerland	0 (0)	0 (0)	0 (1)	1 (2)	1 (2)	1 (2)	1 (2)
Chile	0 (0)	0 (0)	0 (0)	2 (1)	5 (2)	6 (3)	9 (4)
China	66 (0)	282 (1)	344 (2)	588 (2)	577 (4)	923 (6)	1131 (8)
Cote dIvoire	0 (0)	1 (0)	4 (1)	11 (3)	29 (5)	59 (9)	99 (14)
Cameroon	3 (1)	6 (3)	12 (4)	16 (3)	42 (8)	103 (15)	144 (21)
Democratic Republic of the Congo	2 (0)	5 (1)	20 (2)	56 (5)	103 (6)	179 (8)	280 (10)
Republic of Congo	0 (0)	0 (0)	1 (0)	1 (0)	18 (6)	22 (7)	30 (10)
Colombia	18 (3)	30 (4)	52 (7)	113 (14)	120 (15)	160 (21)	171 (23)

Costa Rica	3 (7)	4 (8)	4 (8)	3 (4)	2 (3)	3 (5)	4 (6)
Cuba	1 (1)	2 (1)	2 (1)	2 (2)	2 (2)	2 (2)	2 (2)
Czech Republic	0 (0)	0 (0)	0 (0)	0 (0)	1 (1)	2 (2)	1 (1)
Germany	44 (4)	26 (2)	25 (3)	24 (3)	27 (3)	27 (4)	22 (3)
Denmark	0 (0)	0 (0)	0 (0)	0 (0)	0 (0)	1 (3)	1 (3)
Dominican Republic	1 (0)	1 (1)	2 (1)	3 (2)	4 (2)	3 (2)	4 (3)
Algeria	0 (0)	1 (0)	1 (0)	0 (0)	0 (0)	0 (0)	1 (0)
Ecuador	2 (1)	8 (4)	31 (13)	63 (22)	80 (27)	85 (29)	99 (34)
Egypt	88 (10)	182 (17)	360 (28)	467 (29)	459 (31)	561 (32)	722 (37)
Eritrea	0 (0)	0 (0)	0 (0)	0 (0)	2 (2)	18 (12)	25 (14)
Spain	4 (1)	5 (1)	3 (0)	1 (0)	2 (1)	3 (1)	5 (2)
Estonia	0 (2)	0 (3)	0 (3)	1 (4)	1 (6)	1 (6)	1 (9)
Ethiopia	26 (3)	37 (4)	86 (6)	344 (19)	674 (28)	776 (29)	931 (32)
Finland	2 (2)	1 (2)	1 (2)	1 (2)	2 (3)	2 (3)	2 (4)
Fiji	0 (1)	0 (3)	0 (3)	0 (5)	0 (5)	0 (5)	0 (5)
France	36 (5)	27 (3)	25 (4)	32 (5)	39 (6)	59 (8)	57 (8)
Gabon	0 (2)	0 (2)	1 (4)	1 (4)	1 (5)	2 (6)	3 (9)
United Kingdom	7 (1)	9 (1)	12 (2)	15 (2)	30 (5)	52 (7)	55 (7)
Georgia	1 (1)	1 (1)	0 (1)	1 (1)	1 (1)	1 (1)	1 (2)
Ghana	3 (1)	1 (0)	2 (1)	7 (1)	15 (3)	64 (9)	105 (14)
Guinea	0 (0)	1 (1)	2 (2)	7 (4)	11 (4)	21 (7)	39 (13)
Guinea-Bissau	0 (0)	0 (0)	0 (0)	0 (0)	0 (0)	0 (1)	1 (2)
Equatorial Guinea	0 (0)	0 (1)	0 (3)	1 (5)	1 (6)	1 (7)	0 (3)
Greece	0 (0)	0 (0)	1 (1)	0 (0)	0 (0)	1 (1)	1 (1)
Guatemala	4 (2)	4 (2)	6 (2)	7 (2)	9 (3)	9 (3)	11 (3)
Guyana	0 (2)	1 (4)	1 (4)	1 (4)	1 (4)	1 (4)	0 (4)
Hong Kong	0 (0)	0 (0)	0 (0)	0 (0)	1 (2)	0 (0)	0 (0)
Honduras	1 (2)	2 (2)	2 (2)	2 (2)	2 (1)	2 (1)	4 (2)
Croatia	0 (0)	0 (0)	0 (0)	0 (1)	0 (1)	0 (0)	0 (0)
Haiti	0 (0)	0 (0)	3 (2)	5 (2)	7 (3)	8 (3)	11 (5)
Hungary	3 (2)	3 (2)	3 (2)	1 (1)	1 (1)	1 (1)	1 (1)
Indonesia	6 (0)	87 (3)	145 (4)	113 (3)	139 (4)	209 (5)	280 (8)
India	157 (1)	386 (2)	703 (3)	1194 (5)	1602 (6)	2308 (9)	2842 (12)
Ireland	25 (17)	24 (15)	23 (17)	16 (19)	15 (22)	14 (26)	14 (26)
Iran	1 (0)	2 (0)	2 (0)	3 (0)	5 (0)	11 (1)	26 (2)
Iraq	0 (0)	0 (0)	0 (0)	0 (0)	1 (0)	12 (1)	14 (1)
Italy	0 (0)	1 (0)	1 (0)	1 (0)	2 (1)	4 (1)	6 (1)

Japan	3 (0)	2 (0)	3 (0)	3 (0)	15 (2)	18 (2)	17 (2)
Kazakhstan	7 (2)	9 (3)	13 (4)	19 (5)	15 (6)	28 (9)	34 (10)
Kenya	11 (4)	13 (3)	12 (2)	27 (3)	60 (6)	134 (10)	229 (17)
Kyrgyzstan	0 (0)	1 (2)	2 (2)	3 (3)	3 (3)	6 (5)	9 (7)
Cambodia	0 (0)	0 (0)	7 (4)	30 (8)	47 (14)	57 (17)	70 (21)
Republic of Korea	3 (0)	3 (0)	0 (0)	0 (0)	5 (1)	11 (3)	22 (7)
Lao PDR	0 (0)	0 (0)	5 (5)	16 (12)	21 (15)	24 (18)	25 (19)
Liberia	0 (0)	0 (0)	2 (3)	6 (10)	18 (25)	30 (33)	48 (42)
Sri Lanka	50 (21)	53 (20)	56 (20)	4 (2)	0 (0)	0 (0)	1 (0)
Lesotho	0 (1)	0 (1)	0 (1)	0 (1)	0 (1)	0 (1)	3 (5)
Lithuania	1 (2)	1 (1)	1 (2)	1 (2)	1 (2)	1 (3)	3 (9)
Luxembourg	0 (5)	0 (0)	0 (0)	0 (0)	0 (0)	0 (2)	0 (2)
Latvia	0 (1)	0 (1)	0 (1)	1 (2)	0 (3)	1 (3)	2 (10)
Morocco	0 (0)	1 (0)	1 (0)	2 (0)	4 (1)	4 (1)	8 (1)
Moldova	3 (3)	1 (2)	2 (2)	0 (1)	0 (0)	0 (0)	0 (1)
Madagascar	1 (0)	1 (0)	2 (1)	3 (1)	4 (1)	9 (1)	9 (1)
Mexico	32 (2)	39 (2)	75 (4)	143 (7)	293 (13)	295 (14)	305 (14)
Mali	0 (0)	0 (0)	2 (1)	4 (1)	7 (2)	15 (3)	23 (3)
Myanmar	31 (5)	71 (9)	123 (13)	169 (17)	221 (23)	252 (28)	231 (29)
Montenegro	0 (0)	0 (0)	0 (0)	0 (0)	0 (2)	0 (2)	0 (0)
Mongolia	0 (0)	0 (0)	1 (1)	1 (1)	1 (2)	4 (7)	5 (7)
Mozambique	2 (1)	3 (1)	5 (1)	6 (1)	12 (2)	28 (4)	65 (9)
Mauritania	0 (0)	0 (0)	0 (1)	1 (1)	1 (1)	2 (2)	2 (1)
Malawi	0 (0)	1 (0)	1 (1)	2 (1)	1 (0)	14 (3)	57 (9)
Malaysia	15 (7)	13 (5)	15 (5)	13 (4)	22 (6)	32 (9)	48 (12)
Namibia	0 (0)	0 (0)	0 (0)	0 (0)	0 (1)	1 (2)	2 (3)
New Caledonia	0 (1)	0 (1)	0 (1)	0 (0)	0 (0)	0 (1)	0 (2)
Niger	0 (0)	0 (0)	5 (2)	10 (3)	24 (5)	42 (7)	63 (8)
Nigeria	23 (2)	40 (2)	82 (3)	156 (5)	260 (6)	639 (12)	1063 (17)
Nicaragua	8 (10)	11 (12)	15 (12)	11 (7)	8 (6)	9 (7)	7 (6)
Netherlands	11 (6)	10 (5)	10 (7)	11 (7)	14 (9)	16 (11)	14 (9)
Norway	1 (2)	1 (1)	1 (2)	1 (2)	2 (3)	2 (4)	3 (5)
Nepal	0 (0)	1 (0)	7 (1)	22 (3)	33 (4)	68 (9)	61 (8)
New Zealand	0 (0)	0 (1)	0 (1)	1 (2)	1 (3)	2 (3)	2 (3)
Oman	0 (0)	0 (0)	0 (0)	0 (0)	0 (1)	1 (1)	1 (2)
Pakistan	9 (1)	22 (1)	120 (5)	236 (7)	406 (10)	577 (13)	629 (14)
Panama	3 (11)	6 (16)	7 (18)	7 (17)	8 (17)	9 (19)	9 (19)

Peru	12 (4)	24 (7)	60 (14)	83 (18)	110 (24)	118 (27)	124 (30)
Philippines	2 (0)	2 (0)	5 (0)	20 (2)	80 (6)	105 (7)	121 (8)
Papua New Guinea	0 (1)	0 (1)	1 (1)	5 (5)	10 (8)	15 (9)	19 (12)
Poland	13 (2)	11 (2)	15 (2)	8 (1)	10 (2)	13 (3)	15 (4)
Puerto Rico	1 (1)	0 (0)	1 (1)	1 (1)	1 (2)	1 (2)	1 (3)
Dem. Rep. Korea	0 (0)	1 (0)	0 (0)	0 (0)	2 (1)	4 (1)	12 (5)
Portugal	0 (0)	0 (0)	0 (0)	0 (0)	1 (1)	1 (2)	1 (2)
Paraguay	0 (0)	1 (1)	2 (2)	7 (6)	12 (9)	13 (11)	12 (10)
Romania	6 (2)	5 (1)	2 (1)	1 (0)	1 (0)	1 (0)	3 (1)
Russian Federation	127 (5)	87 (5)	85 (4)	71 (3)	43 (3)	76 (5)	91 (6)
Rwanda	0 (0)	1 (1)	2 (1)	5 (2)	7 (2)	17 (4)	26 (6)
Saudi Arabia	0 (0)	0 (0)	0 (0)	0 (0)	0 (0)	1 (0)	1 (0)
Sudan	31 (9)	43 (10)	74 (13)	106 (15)	152 (18)	259 (24)	328 (26)
Senegal	0 (0)	0 (0)	1 (0)	4 (2)	5 (2)	7 (2)	9 (2)
Sierra Leone	0 (0)	1 (1)	3 (3)	8 (7)	21 (16)	38 (23)	46 (26)
El Salvador	0 (0)	2 (2)	2 (2)	2 (2)	3 (2)	2 (2)	2 (2)
Somalia	0 (1)	2 (2)	5 (3)	11 (6)	29 (12)	45 (16)	61 (19)
Serbia	2 (2)	2 (2)	1 (1)	0 (0)	0 (0)	0 (0)	0 (1)
South Sudan	4 (5)	7 (5)	13 (8)	22 (10)	31 (11)	35 (13)	48 (16)
Suriname	0 (0)	0 (0)	0 (0)	0 (0)	0 (1)	0 (1)	0 (1)
Slovakia	3 (3)	1 (2)	2 (2)	0 (0)	0 (0)	0 (1)	0 (1)
Slovenia	0 (0)	0 (0)	0 (0)	0 (0)	0 (2)	0 (2)	1 (2)
Sweden	1 (2)	2 (2)	3 (3)	5 (5)	6 (7)	6 (7)	7 (8)
Swaziland	0 (1)	0 (2)	1 (4)	1 (3)	1 (4)	1 (5)	7 (25)
Syria	0 (0)	0 (0)	0 (0)	0 (0)	0 (0)	1 (0)	0 (0)
Chad	1 (1)	5 (4)	8 (5)	22 (9)	39 (12)	53 (12)	82 (16)
Togo	4 (7)	2 (3)	2 (2)	5 (4)	8 (5)	32 (16)	40 (20)
Thailand	8 (1)	16 (1)	79 (7)	141 (14)	150 (17)	167 (21)	192 (27)
Tajikistan	0 (1)	1 (1)	4 (3)	9 (5)	12 (7)	18 (10)	22 (13)
Turkmenistan	2 (3)	2 (3)	5 (5)	7 (6)	7 (6)	8 (7)	12 (9)
Timor-Leste	0 (0)	0 (0)	0 (0)	0 (0)	0 (0)	4 (11)	4 (11)
Tunisia	0 (0)	0 (0)	0 (0)	0 (0)	1 (1)	1 (1)	1 (0)
Turkey	2 (0)	2 (0)	2 (0)	2 (0)	5 (0)	7 (1)	13 (1)

Tanzania	6 (2)	9 (2)	11 (2)	21 (3)	44 (4)	114 (8)	248 (14)
Uganda	25 (9)	34 (9)	32 (6)	61 (9)	127 (13)	255 (20)	357 (22)
Ukraine	32 (4)	27 (4)	22 (3)	5 (1)	2 (0)	4 (1)	6 (1)
Uruguay	0 (0)	0 (0)	1 (1)	1 (2)	3 (6)	3 (7)	3 (7)
United States	38 (1)	41 (1)	57 (2)	107 (3)	136 (4)	176 (4)	220 (5)
Uzbekistan	4 (1)	8 (2)	19 (4)	37 (6)	39 (7)	47 (9)	56 (10)
Venezuela	31 (13)	46 (14)	58 (15)	61 (13)	69 (14)	71 (14)	66 (13)
Vietnam	4 (0)	11 (1)	47 (3)	117 (7)	101 (8)	134 (10)	158 (12)
Yemen	0 (0)	0 (0)	0 (0)	0 (0)	8 (1)	17 (2)	27 (3)
South Africa	2 (0)	3 (0)	8 (1)	12 (1)	16 (2)	20 (2)	43 (4)
Zambia	0 (0)	1 (0)	1 (0)	1 (0)	13 (4)	17 (4)	31 (5)
Zimbabwe	1 (1)	2 (1)	2 (1)	3 (1)	3 (1)	4 (1)	5 (1)

Table 6: **Absolute population (in thousands) of cohorts living unprecedented exposure to floods and CF_{floods} (%) per country and birth year in a 3.5°C pathway**

Country	1960	1970	1980	1990	2000	2010	2020
Afghanistan	1 (0)	2 (0)	6 (1)	17 (3)	39 (4)	77 (7)	118 (9)
Angola	2 (1)	3 (1)	5 (2)	13 (4)	25 (5)	78 (12)	106 (16)
Albania	0 (0)	0 (0)	0 (0)	0 (0)	0 (0)	0 (0)	1 (1)
United Arab Emirates	0 (0)	0 (0)	0 (0)	0 (0)	0 (0)	0 (0)	0 (0)
Argentina	5 (1)	9 (2)	13 (2)	16 (2)	24 (3)	44 (6)	88 (12)
Australia	0 (0)	7 (3)	8 (4)	4 (2)	3 (1)	11 (4)	14 (5)
Azerbaijan	0 (0)	0 (0)	0 (0)	0 (0)	0 (0)	1 (1)	2 (2)
Burundi	0 (0)	0 (0)	1 (1)	4 (2)	4 (2)	13 (4)	18 (5)
Belgium	8 (5)	4 (2)	3 (2)	3 (3)	3 (2)	2 (1)	1 (1)
Benin	3 (4)	3 (4)	4 (3)	5 (3)	14 (7)	20 (8)	26 (9)
Burkina Faso	3 (2)	4 (2)	6 (2)	7 (2)	8 (2)	15 (3)	21 (3)
Bangladesh	44 (3)	59 (3)	91 (4)	238 (8)	459 (15)	558 (20)	607 (23)
Bosnia and Herzegovina	1 (1)	2 (2)	1 (2)	2 (3)	1 (3)	0 (1)	2 (6)
Belarus	7 (3)	5 (3)	4 (3)	2 (1)	1 (1)	1 (1)	1 (1)
Bolivia	1 (1)	3 (2)	5 (3)	23 (12)	43 (19)	67 (28)	91 (38)
Brazil	42 (2)	50 (2)	61 (2)	152 (5)	233 (8)	277 (11)	303 (12)
Brunei Darussalam	0 (0)	0 (0)	0 (0)	0 (0)	0 (0)	0 (4)	0 (4)
Bhutan	0 (0)	0 (0)	1 (3)	1 (6)	4 (18)	4 (19)	5 (25)
Botswana	0 (0)	0 (0)	0 (0)	0 (0)	0 (0)	1 (2)	1 (2)
Central African Republic	0 (0)	1 (1)	3 (4)	4 (5)	8 (8)	8 (7)	18 (15)
Canada	5 (1)	13 (4)	20 (6)	24 (7)	30 (9)	38 (11)	51 (14)
Switzerland	0 (0)	0 (0)	0 (0)	2 (2)	4 (5)	5 (8)	9 (12)
Chile	0 (0)	0 (0)	0 (0)	0 (0)	1 (0)	3 (1)	1 (1)
China	50 (0)	185 (1)	231 (1)	652 (3)	782 (5)	1452 (9)	1782 (13)
Cote dIvoire	0 (0)	1 (0)	2 (1)	4 (1)	13 (2)	46 (7)	58 (8)
Cameroon	1 (1)	4 (2)	7 (2)	34 (7)	33 (6)	82 (12)	115 (16)
Democratic Republic of the Congo	2 (0)	6 (1)	22 (2)	39 (3)	134 (8)	222 (10)	421 (16)

Republic of Congo	0 (0)	0 (0)	1 (1)	1 (1)	2 (1)	35 (12)	89 (29)
Colombia	15 (3)	20 (3)	35 (5)	75 (9)	146 (19)	197 (26)	227 (30)
Costa Rica	2 (5)	4 (8)	4 (8)	2 (3)	1 (2)	5 (7)	3 (4)
Cuba	1 (1)	1 (1)	1 (1)	1 (1)	2 (2)	2 (3)	1 (2)
Germany	47 (4)	40 (3)	31 (4)	30 (3)	48 (6)	47 (7)	45 (6)
Djibouti	0 (0)	0 (0)	0 (0)	0 (0)	0 (0)	0 (0)	0 (1)
Denmark	0 (0)	0 (0)	0 (0)	0 (0)	0 (0)	1 (1)	0 (0)
Dominican Republic	0 (0)	0 (0)	1 (1)	1 (1)	2 (1)	3 (2)	3 (2)
Algeria	0 (0)	0 (0)	1 (0)	0 (0)	0 (0)	3 (0)	9 (1)
Ecuador	3 (2)	12 (6)	28 (11)	69 (24)	89 (31)	91 (31)	115 (39)
Egypt	120 (14)	218 (21)	326 (26)	487 (30)	454 (31)	542 (31)	610 (31)
Eritrea	0 (0)	0 (0)	0 (0)	3 (3)	7 (7)	13 (8)	26 (15)
Spain	3 (1)	4 (1)	4 (1)	0 (0)	1 (0)	1 (0)	2 (0)
Estonia	0 (0)	0 (2)	0 (2)	0 (2)	0 (2)	1 (4)	1 (6)
Ethiopia	19 (2)	26 (3)	113 (8)	321 (18)	580 (24)	703 (27)	1017 (35)
Finland	1 (2)	1 (2)	1 (2)	1 (2)	1 (2)	2 (4)	3 (5)
France	28 (4)	17 (2)	40 (6)	23 (3)	25 (4)	41 (6)	104 (15)
Gabon	0 (1)	0 (1)	1 (5)	1 (3)	1 (3)	3 (8)	6 (18)
United Kingdom	4 (1)	6 (1)	11 (2)	18 (3)	27 (4)	38 (5)	43 (6)
Georgia	1 (1)	0 (1)	1 (1)	1 (1)	1 (1)	1 (2)	1 (2)
Ghana	2 (1)	1 (0)	2 (1)	5 (1)	10 (2)	15 (2)	23 (3)
Guinea	0 (0)	0 (0)	1 (1)	9 (5)	11 (4)	17 (6)	36 (12)
The Gambia	0 (0)	0 (0)	0 (0)	0 (0)	0 (1)	1 (1)	1 (3)
Guinea-Bissau	0 (0)	0 (0)	0 (0)	0 (0)	0 (0)	1 (3)	3 (9)
Equatorial Guinea	0 (0)	0 (0)	0 (2)	0 (1)	1 (7)	2 (17)	6 (43)
Greece	0 (0)	1 (1)	1 (1)	0 (0)	0 (0)	0 (0)	0 (0)
Guatemala	2 (1)	3 (2)	5 (2)	6 (2)	7 (2)	14 (4)	15 (4)
Guyana	0 (2)	0 (2)	1 (5)	0 (1)	0 (1)	1 (5)	1 (5)
Hong Kong	0 (0)	0 (0)	0 (0)	0 (0)	2 (4)	0 (0)	0 (0)
Honduras	1 (1)	1 (1)	2 (1)	1 (1)	2 (1)	2 (1)	1 (0)
Croatia	0 (0)	0 (1)	0 (1)	0 (1)	0 (1)	0 (0)	0 (1)
Haiti	0 (0)	0 (0)	1 (1)	1 (0)	3 (1)	10 (5)	16 (7)
Hungary	5 (3)	3 (2)	4 (2)	1 (0)	0 (0)	0 (0)	0 (0)
Indonesia	6 (0)	17 (1)	46 (1)	70 (2)	173 (5)	273 (7)	363 (10)
India	161 (1)	332 (2)	466 (2)	660 (3)	1804 (7)	2909 (12)	3952 (17)

Ireland	15 (11)	16 (10)	13 (10)	10 (12)	11 (17)	14 (26)	14 (26)
Iran	3 (0)	3 (0)	3 (0)	4 (0)	7 (1)	23 (2)	32 (3)
Iraq	0 (0)	0 (0)	0 (0)	1 (0)	1 (0)	1 (0)	1 (0)
Iceland	0 (0)	0 (0)	0 (0)	0 (0)	0 (0)	0 (0)	0 (3)
Israel	0 (0)	0 (0)	0 (0)	0 (0)	4 (5)	5 (5)	6 (5)
Italy	0 (0)	1 (0)	2 (0)	2 (1)	2 (1)	6 (1)	10 (2)
Japan	4 (0)	3 (0)	3 (0)	3 (0)	10 (1)	13 (1)	26 (3)
Kazakhstan	9 (3)	12 (4)	17 (5)	19 (5)	13 (6)	25 (8)	38 (11)
Kenya	10 (3)	15 (3)	19 (3)	24 (3)	60 (6)	174 (14)	168 (12)
Kyrgyzstan	0 (0)	0 (0)	1 (1)	1 (0)	2 (2)	3 (3)	7 (5)
Cambodia	0 (0)	0 (0)	9 (5)	41 (11)	51 (15)	60 (17)	71 (21)
Republic of Korea	0 (0)	0 (0)	0 (0)	0 (0)	0 (0)	1 (0)	3 (1)
Lao PDR	0 (0)	0 (0)	4 (4)	11 (8)	13 (9)	17 (12)	20 (15)
Liberia	0 (0)	0 (0)	3 (5)	6 (11)	13 (18)	25 (27)	42 (37)
Libya	0 (0)	0 (0)	0 (0)	0 (0)	0 (0)	2 (2)	0 (0)
Sri Lanka	40 (17)	42 (16)	46 (17)	1 (0)	0 (0)	5 (2)	5 (2)
Lesotho	0 (2)	1 (2)	1 (2)	1 (2)	1 (2)	0 (0)	0 (1)
Lithuania	1 (1)	0 (1)	1 (1)	1 (2)	1 (2)	0 (0)	1 (1)
Luxembourg	0 (4)	0 (0)	0 (0)	0 (0)	0 (4)	0 (4)	0 (4)
Latvia	0 (0)	0 (0)	0 (0)	1 (2)	0 (1)	1 (2)	1 (3)
Morocco	0 (0)	0 (0)	0 (0)	0 (0)	0 (0)	0 (0)	1 (0)
Moldova	4 (5)	3 (4)	3 (4)	1 (2)	0 (0)	0 (0)	0 (0)
Madagascar	1 (1)	2 (1)	3 (1)	3 (1)	10 (2)	15 (3)	21 (3)
Mexico	9 (1)	12 (1)	75 (3)	156 (7)	285 (13)	332 (16)	349 (16)
Mali	0 (0)	0 (0)	1 (0)	2 (1)	4 (1)	16 (3)	37 (5)
Myanmar	15 (2)	49 (6)	63 (7)	92 (10)	172 (18)	228 (25)	290 (37)
Montenegro	0 (0)	0 (0)	0 (0)	0 (0)	0 (0)	0 (0)	0 (8)
Mongolia	0 (0)	0 (1)	1 (1)	2 (2)	3 (6)	4 (8)	7 (11)
Mozambique	2 (1)	5 (2)	4 (1)	7 (2)	13 (2)	44 (6)	88 (12)
Mauritania	0 (0)	0 (0)	1 (1)	1 (1)	1 (1)	2 (1)	3 (3)
Malawi	0 (0)	1 (0)	0 (0)	1 (0)	3 (1)	24 (4)	78 (12)
Malaysia	11 (5)	10 (4)	10 (4)	20 (6)	35 (9)	45 (13)	55 (14)
Namibia	0 (0)	0 (0)	0 (0)	0 (0)	0 (0)	2 (3)	2 (4)
New Caledonia	0 (1)	0 (1)	0 (1)	0 (0)	0 (0)	0 (1)	0 (16)
Niger	0 (0)	1 (0)	5 (2)	12 (4)	20 (5)	45 (7)	92 (11)
Nigeria	21 (1)	40 (2)	92 (3)	147 (4)	323 (8)	490 (9)	760 (12)
Nicaragua	6 (8)	8 (9)	12 (10)	8 (6)	7 (5)	7 (5)	2 (2)
Netherlands	13 (7)	14 (7)	12 (8)	15 (10)	18 (11)	20 (13)	17 (11)

Norway	1 (2)	1 (1)	1 (2)	1 (3)	1 (3)	1 (3)	2 (3)
Nepal	0 (0)	0 (0)	1 (0)	1 (0)	28 (3)	111 (14)	157 (20)
New Zealand	0 (0)	0 (1)	0 (1)	0 (1)	1 (1)	2 (3)	3 (5)
Pakistan	2 (0)	13 (1)	50 (2)	78 (2)	151 (4)	292 (7)	721 (16)
Panama	3 (11)	5 (14)	6 (15)	5 (13)	6 (14)	8 (17)	9 (19)
Peru	12 (4)	29 (8)	50 (12)	80 (17)	113 (25)	142 (33)	157 (38)
Philippines	1 (0)	1 (0)	1 (0)	5 (0)	31 (2)	107 (7)	200 (13)
Papua New Guinea	1 (1)	1 (2)	3 (3)	5 (5)	18 (14)	31 (20)	53 (33)
Poland	24 (3)	16 (3)	28 (4)	6 (1)	6 (1)	8 (2)	9 (2)
Puerto Rico	1 (1)	1 (1)	0 (0)	0 (1)	0 (1)	0 (1)	0 (1)
Dem. Rep. Korea	0 (0)	0 (0)	0 (0)	0 (0)	0 (0)	1 (0)	2 (1)
Paraguay	0 (0)	1 (2)	3 (3)	6 (5)	10 (8)	13 (12)	13 (11)
Romania	9 (2)	7 (2)	3 (1)	0 (0)	1 (0)	1 (0)	1 (1)
Russian Federation	141 (5)	103 (6)	85 (4)	63 (3)	41 (3)	71 (5)	84 (5)
Rwanda	0 (0)	1 (1)	1 (1)	2 (1)	3 (1)	11 (3)	41 (9)
Sudan	26 (8)	39 (9)	69 (12)	89 (13)	156 (18)	227 (21)	300 (24)
Senegal	0 (0)	0 (0)	1 (0)	1 (1)	6 (2)	21 (5)	56 (12)
Sierra Leone	0 (0)	1 (1)	2 (2)	6 (5)	12 (9)	28 (17)	43 (24)
El Salvador	0 (0)	0 (0)	0 (0)	0 (0)	1 (1)	1 (1)	3 (3)
Somalia	0 (0)	2 (2)	3 (2)	10 (5)	24 (10)	35 (12)	66 (21)
Serbia	3 (3)	2 (2)	1 (1)	0 (0)	0 (0)	0 (0)	1 (1)
South Sudan	3 (4)	5 (4)	13 (8)	19 (9)	31 (11)	39 (14)	60 (19)
Suriname	0 (0)	0 (0)	0 (0)	0 (0)	0 (0)	0 (1)	0 (1)
Slovakia	5 (5)	2 (3)	3 (3)	0 (0)	0 (0)	0 (1)	0 (0)
Sweden	1 (1)	2 (2)	3 (3)	4 (4)	3 (3)	5 (6)	6 (7)
Swaziland	0 (2)	1 (4)	2 (8)	1 (3)	1 (3)	3 (11)	3 (10)
Chad	1 (1)	6 (4)	17 (9)	27 (11)	47 (14)	66 (15)	78 (16)
Togo	2 (4)	1 (1)	1 (1)	0 (0)	7 (4)	15 (8)	18 (9)
Thailand	3 (0)	15 (1)	69 (6)	129 (12)	133 (15)	167 (21)	171 (24)
Tajikistan	1 (1)	1 (1)	2 (2)	3 (2)	7 (4)	15 (9)	27 (16)
Turkmenistan	2 (4)	3 (4)	4 (5)	6 (5)	6 (6)	8 (7)	10 (7)
Timor-Leste	0 (0)	0 (0)	0 (0)	1 (3)	2 (5)	3 (9)	4 (9)
Tunisia	0 (0)	0 (0)	0 (0)	0 (0)	1 (0)	0 (0)	3 (2)

Turkey	2 (0)	2 (0)	2 (0)	3 (0)	12 (1)	10 (1)	14 (1)
Tanzania	8 (2)	16 (4)	12 (2)	28 (3)	65 (6)	146 (10)	169 (10)
Uganda	17 (6)	27 (7)	28 (6)	48 (7)	112 (11)	234 (18)	405 (25)
Ukraine	47 (6)	38 (6)	30 (4)	3 (0)	1 (0)	3 (1)	4 (1)
Uruguay	0 (0)	0 (0)	0 (1)	1 (2)	2 (4)	4 (8)	5 (12)
United States	43 (1)	47 (1)	47 (1)	83 (2)	115 (3)	186 (5)	188 (5)
Uzbekistan	8 (3)	12 (3)	16 (3)	26 (4)	42 (7)	72 (13)	99 (18)
Venezuela	22 (9)	35 (11)	46 (12)	31 (6)	32 (6)	36 (7)	39 (8)
Vietnam	3 (0)	12 (1)	33 (2)	84 (5)	97 (7)	200 (15)	230 (18)
Yemen	0 (0)	0 (0)	0 (0)	0 (0)	4 (1)	5 (1)	23 (3)
South Africa	2 (0)	4 (1)	13 (1)	12 (1)	15 (2)	30 (3)	36 (4)
Zambia	0 (0)	0 (0)	0 (0)	0 (0)	4 (1)	15 (3)	44 (8)
Zimbabwe	2 (1)	4 (2)	3 (1)	4 (1)	2 (1)	9 (2)	23 (6)

Table 7: **Absolute population (in thousands) of cohorts living unprecedented exposure to droughts and $CF_{droughts}$ (%) per country and birth year in a 1.5°C pathway**

Country	1960	1970	1980	1990	2000	2010	2020
Afghanistan	14 (4)	19 (4)	31 (6)	28 (5)	70 (8)	90 (8)	99 (7)
Angola	2 (1)	4 (2)	5 (2)	8 (2)	12 (3)	18 (3)	18 (3)
Albania	1 (2)	1 (2)	2 (3)	3 (4)	2 (5)	2 (5)	2 (6)
United Arab Emirates	0 (12)	0 (14)	2 (17)	3 (14)	3 (15)	4 (15)	5 (15)
Argentina	5 (1)	7 (1)	11 (2)	13 (2)	15 (2)	15 (2)	15 (2)
Armenia	13 (20)	12 (21)	14 (21)	10 (13)	5 (12)	6 (13)	6 (15)
Australia	4 (2)	13 (6)	13 (6)	15 (6)	16 (7)	17 (7)	19 (6)
Austria	5 (4)	7 (6)	7 (8)	8 (9)	7 (9)	7 (9)	7 (8)
Azerbaijan	9 (8)	10 (8)	8 (8)	10 (7)	7 (7)	8 (8)	10 (9)
Burundi	0 (0)	1 (1)	1 (1)	2 (1)	3 (1)	5 (1)	4 (1)
Belgium	0 (0)	0 (0)	0 (0)	1 (1)	1 (1)	2 (1)	2 (1)
Benin	3 (4)	4 (4)	4 (4)	6 (4)	9 (4)	11 (4)	11 (4)
Burkina Faso	11 (7)	14 (7)	23 (9)	39 (11)	54 (12)	76 (13)	85 (12)
Bangladesh	29 (2)	30 (1)	44 (2)	59 (2)	60 (2)	67 (2)	62 (2)
Bulgaria	6 (5)	7 (6)	15 (11)	17 (16)	10 (16)	11 (16)	10 (16)
Bahamas	0 (8)	0 (8)	0 (2)	0 (1)	0 (1)	0 (0)	0 (0)
Bosnia and Herzegovina	3 (4)	3 (4)	5 (7)	5 (7)	4 (8)	3 (8)	3 (8)
Belarus	1 (1)	1 (1)	1 (1)	3 (2)	2 (2)	3 (3)	3 (3)
Belize	0 (1)	0 (0)	0 (2)	0 (2)	0 (2)	0 (2)	0 (2)
Bolivia	5 (4)	7 (5)	12 (7)	18 (9)	20 (9)	22 (9)	24 (10)
Brazil	18 (1)	41 (2)	83 (3)	109 (3)	121 (4)	98 (4)	101 (4)
Brunei Darussalam	0 (0)	0 (0)	0 (0)	0 (0)	0 (0)	0 (0)	0 (0)
Bhutan	0 (0)	0 (1)	0 (2)	0 (2)	0 (2)	0 (2)	0 (2)
Botswana	1 (5)	2 (7)	3 (9)	4 (9)	4 (9)	5 (11)	5 (11)
Central African Republic	0 (1)	1 (2)	2 (2)	2 (2)	3 (3)	4 (3)	4 (3)
Canada	16 (4)	15 (4)	16 (5)	17 (5)	17 (5)	19 (6)	21 (6)
Switzerland	1 (1)	1 (1)	1 (1)	1 (1)	1 (1)	1 (1)	0 (0)
Chile	26 (13)	44 (19)	48 (23)	57 (23)	52 (23)	49 (22)	51 (24)
China	442 (2)	1121 (4)	969 (5)	1269 (5)	755 (5)	762 (5)	666 (5)
Cote dIvoire	8 (6)	18 (8)	35 (10)	58 (13)	93 (16)	105 (16)	110 (16)
Cameroon	4 (2)	6 (3)	8 (2)	12 (3)	14 (3)	19 (3)	19 (3)

Democratic Republic of the Congo	33 (6)	55 (8)	71 (8)	90 (7)	134 (8)	179 (8)	216 (8)
Republic of Congo	1 (2)	2 (2)	2 (1)	2 (1)	2 (1)	3 (1)	7 (2)
Colombia	4 (1)	5 (1)	7 (1)	16 (2)	15 (2)	15 (2)	29 (4)
Cape Verde	0 (9)	0 (14)	0 (21)	1 (23)	1 (23)	0 (0)	0 (0)
Costa Rica	1 (2)	1 (2)	1 (2)	1 (2)	2 (2)	2 (2)	3 (4)
Cuba	1 (1)	3 (2)	4 (3)	4 (4)	4 (4)	3 (3)	3 (4)
Cyprus	1 (26)	1 (27)	1 (33)	1 (29)	1 (27)	1 (26)	1 (24)
Czech Republic	6 (5)	9 (7)	13 (9)	10 (9)	7 (9)	15 (13)	14 (14)
Germany	34 (3)	39 (3)	21 (3)	32 (4)	28 (3)	35 (5)	35 (5)
Djibouti	0 (13)	0 (10)	0 (7)	0 (3)	0 (3)	0 (3)	0 (4)
Denmark	3 (5)	3 (5)	2 (5)	3 (6)	3 (6)	2 (6)	2 (6)
Dominican Republic	0 (0)	1 (0)	1 (0)	1 (1)	2 (1)	2 (1)	3 (2)
Algeria	23 (6)	37 (8)	61 (9)	85 (11)	63 (11)	79 (11)	82 (11)
Ecuador	1 (0)	1 (0)	1 (1)	1 (0)	1 (1)	1 (0)	1 (1)
Egypt	132 (15)	171 (16)	211 (17)	299 (18)	266 (18)	319 (18)	346 (18)
Eritrea	6 (12)	3 (4)	3 (4)	4 (4)	4 (4)	6 (4)	6 (3)
Spain	38 (7)	46 (9)	51 (10)	31 (9)	25 (8)	32 (8)	26 (8)
Estonia	0 (1)	0 (1)	0 (1)	0 (1)	0 (2)	0 (2)	0 (2)
Ethiopia	9 (1)	12 (1)	17 (1)	34 (2)	52 (2)	57 (2)	69 (2)
Finland	1 (1)	1 (1)	0 (1)	1 (1)	1 (1)	0 (1)	1 (1)
France	12 (2)	16 (2)	13 (2)	14 (2)	10 (2)	13 (2)	18 (3)
United Kingdom	23 (3)	22 (3)	17 (3)	25 (3)	15 (2)	15 (2)	15 (2)
Georgia	9 (11)	11 (12)	11 (13)	11 (12)	6 (11)	7 (12)	6 (12)
Ghana	20 (9)	27 (9)	43 (12)	60 (13)	78 (14)	93 (14)	101 (13)
Guinea	0 (0)	1 (1)	4 (3)	22 (12)	32 (12)	43 (15)	46 (15)
The Gambia	1 (12)	1 (13)	3 (20)	7 (24)	9 (25)	12 (25)	11 (22)
Guinea-Bissau	1 (10)	1 (9)	2 (11)	5 (25)	7 (27)	8 (30)	9 (29)
Equatorial Guinea	0 (0)	0 (1)	0 (1)	0 (1)	0 (2)	0 (1)	0 (1)
Greece	1 (1)	4 (3)	7 (7)	8 (10)	8 (10)	10 (12)	8 (12)
Guatemala	4 (3)	8 (4)	13 (5)	18 (6)	22 (6)	22 (6)	29 (7)

Guyana	2 (11)	4 (21)	6 (32)	5 (32)	5 (32)	4 (30)	4 (29)
Hong Kong	0 (0)	0 (0)	0 (0)	1 (2)	1 (2)	0 (0)	0 (0)
Honduras	6 (8)	7 (8)	13 (11)	17 (11)	17 (10)	17 (10)	17 (11)
Croatia	2 (3)	2 (3)	5 (8)	5 (9)	4 (9)	4 (10)	3 (9)
Haiti	0 (0)	1 (1)	1 (1)	1 (1)	0 (0)	0 (0)	0 (0)
Hungary	10 (6)	10 (7)	20 (11)	15 (12)	12 (12)	11 (11)	10 (11)
Indonesia	19 (1)	28 (1)	93 (2)	78 (2)	82 (2)	88 (2)	79 (2)
India	156 (1)	200 (1)	241 (1)	333 (1)	395 (2)	457 (2)	439 (2)
Ireland	0 (0)	1 (1)	1 (1)	1 (1)	1 (1)	0 (1)	0 (1)
Iran	85 (11)	112 (12)	149 (11)	178 (10)	116 (9)	114 (9)	112 (9)
Iraq	28 (11)	53 (13)	66 (13)	99 (16)	125 (16)	149 (15)	159 (14)
Iceland	0 (0)	0 (0)	0 (0)	0 (1)	0 (1)	0 (1)	0 (1)
Israel	5 (14)	7 (17)	12 (22)	18 (28)	26 (30)	32 (28)	37 (28)
Italy	13 (2)	23 (3)	24 (4)	23 (5)	22 (5)	25 (5)	26 (6)
Jordan	2 (5)	5 (9)	11 (14)	26 (25)	34 (26)	36 (25)	38 (26)
Japan	4 (0)	6 (0)	4 (0)	4 (0)	3 (0)	9 (1)	8 (1)
Kazakhstan	3 (1)	2 (1)	6 (2)	9 (2)	5 (2)	10 (3)	11 (3)
Kenya	1 (0)	2 (1)	8 (1)	12 (1)	16 (2)	25 (2)	34 (2)
Kyrgyzstan	2 (2)	2 (2)	4 (4)	5 (4)	4 (4)	5 (4)	5 (4)
Cambodia	1 (0)	0 (0)	2 (1)	4 (1)	10 (3)	9 (3)	8 (2)
Republic of Korea	1 (0)	5 (1)	6 (1)	4 (1)	4 (1)	3 (1)	2 (1)
Kuwait	1 (16)	3 (18)	4 (20)	6 (21)	5 (21)	7 (23)	7 (23)
Lao PDR	1 (1)	2 (2)	3 (3)	5 (3)	5 (3)	4 (3)	4 (3)
Lebanon	4 (8)	7 (10)	10 (14)	11 (19)	12 (19)	9 (19)	12 (20)
Liberia	0 (0)	0 (0)	1 (1)	3 (4)	4 (5)	5 (6)	6 (5)
Libya	4 (9)	7 (10)	11 (11)	18 (16)	17 (17)	18 (17)	20 (17)
Sri Lanka	1 (0)	1 (0)	1 (0)	0 (0)	0 (0)	0 (0)	0 (0)
Lesotho	2 (7)	2 (7)	4 (8)	4 (8)	4 (8)	7 (12)	7 (12)
Lithuania	1 (2)	1 (2)	0 (1)	1 (2)	1 (3)	1 (2)	1 (2)
Latvia	0 (1)	1 (2)	0 (1)	1 (2)	1 (3)	1 (2)	0 (2)
Morocco	37 (11)	71 (17)	105 (22)	116 (23)	101 (22)	111 (21)	111 (21)
Moldova	1 (1)	1 (1)	2 (3)	3 (4)	2 (4)	1 (4)	1 (4)
Madagascar	2 (1)	4 (2)	6 (2)	14 (4)	22 (4)	25 (4)	40 (6)
Mexico	244 (18)	357 (20)	432 (20)	377 (17)	381 (17)	363 (17)	361 (17)
Macedonia	2 (5)	2 (6)	3 (10)	4 (15)	3 (15)	3 (16)	3 (16)
Mali	11 (6)	14 (7)	21 (8)	34 (10)	46 (11)	67 (11)	76 (11)
Myanmar	6 (1)	6 (1)	12 (1)	15 (2)	12 (1)	10 (1)	9 (1)
Montenegro	0 (1)	0 (1)	0 (1)	0 (1)	0 (1)	0 (1)	0 (1)

Mongolia	3 (9)	5 (11)	8 (14)	10 (15)	7 (15)	9 (15)	10 (15)
Mozambique	6 (2)	10 (3)	14 (4)	18 (4)	25 (4)	32 (4)	40 (5)
Mauritania	3 (10)	5 (11)	7 (13)	10 (15)	13 (15)	15 (15)	16 (14)
Malawi	6 (5)	12 (7)	21 (9)	30 (9)	38 (10)	50 (9)	63 (10)
Malaysia	17 (8)	20 (8)	23 (8)	7 (2)	8 (2)	5 (1)	5 (1)
Namibia	1 (5)	1 (6)	2 (6)	3 (7)	3 (7)	4 (7)	4 (7)
New Caledonia	0 (0)	0 (0)	0 (0)	0 (0)	0 (0)	0 (2)	0 (3)
Niger	6 (4)	9 (5)	16 (7)	22 (7)	34 (8)	47 (7)	51 (6)
Nigeria	35 (2)	48 (3)	63 (2)	73 (2)	97 (2)	123 (2)	140 (2)
Nicaragua	6 (9)	8 (9)	15 (12)	20 (14)	19 (14)	17 (13)	17 (14)
Netherlands	1 (0)	1 (0)	1 (1)	2 (1)	2 (1)	3 (2)	3 (2)
Norway	2 (4)	1 (2)	0 (0)	0 (1)	0 (1)	0 (1)	0 (1)
Nepal	0 (0)	1 (0)	2 (0)	2 (0)	6 (1)	6 (1)	9 (1)
New Zealand	0 (0)	0 (1)	0 (1)	0 (1)	0 (1)	1 (1)	1 (1)
Oman	3 (17)	4 (17)	5 (15)	6 (12)	5 (11)	5 (10)	5 (9)
Pakistan	45 (3)	63 (3)	89 (3)	119 (3)	118 (3)	109 (3)	107 (2)
Panama	1 (3)	1 (4)	2 (5)	1 (3)	1 (3)	1 (3)	4 (9)
Peru	7 (2)	13 (3)	15 (3)	15 (3)	15 (3)	14 (3)	13 (3)
Philippines	45 (6)	55 (7)	50 (5)	45 (4)	84 (6)	84 (6)	88 (6)
Papua New Guinea	1 (2)	1 (2)	2 (2)	2 (2)	2 (1)	2 (1)	2 (1)
Poland	11 (2)	7 (1)	15 (2)	20 (3)	15 (4)	18 (5)	17 (4)
Puerto Rico	2 (3)	2 (3)	0 (0)	0 (0)	0 (0)	0 (1)	0 (1)
Dem. Rep. Korea	14 (5)	21 (6)	15 (6)	19 (6)	15 (5)	8 (3)	7 (3)
Portugal	10 (7)	9 (7)	11 (9)	7 (8)	6 (7)	5 (7)	5 (7)
Paraguay	3 (4)	4 (5)	5 (5)	2 (2)	2 (2)	2 (2)	2 (2)
Qatar	0 (25)	1 (25)	1 (21)	2 (25)	2 (25)	3 (25)	3 (23)
Romania	24 (6)	30 (7)	44 (11)	41 (12)	26 (12)	26 (12)	23 (12)
Russian Federation	48 (2)	35 (2)	41 (2)	35 (2)	21 (2)	36 (2)	42 (3)
Rwanda	0 (0)	1 (1)	1 (0)	1 (0)	2 (1)	3 (1)	3 (1)
Saudi Arabia	16 (13)	30 (16)	54 (17)	77 (16)	67 (15)	80 (14)	80 (13)
Sudan	42 (13)	56 (13)	83 (14)	111 (16)	131 (15)	164 (15)	193 (16)
Senegal	17 (15)	25 (16)	46 (22)	65 (24)	83 (26)	106 (25)	119 (25)
Solomon Islands	0 (1)	0 (2)	0 (3)	0 (1)	0 (2)	0 (3)	0 (3)

Sierra Leone	0 (0)	0 (1)	1 (1)	2 (2)	5 (4)	10 (6)	12 (7)
El Salvador	3 (3)	5 (4)	7 (5)	8 (5)	9 (6)	7 (6)	6 (6)
Somalia	1 (1)	1 (1)	5 (3)	7 (3)	8 (4)	10 (3)	12 (4)
Serbia	5 (5)	5 (5)	10 (8)	9 (9)	8 (10)	7 (10)	7 (10)
South Sudan	1 (2)	3 (2)	5 (3)	8 (4)	13 (5)	11 (4)	13 (4)
Sao Tome and Principe	0 (7)	0 (7)	0 (7)	0 (7)	0 (7)	0 (0)	0 (0)
Suriname	2 (17)	3 (23)	2 (25)	3 (25)	3 (25)	3 (27)	2 (21)
Slovakia	3 (3)	3 (3)	7 (7)	7 (8)	5 (8)	5 (8)	5 (8)
Slovenia	0 (0)	0 (1)	1 (2)	1 (3)	0 (3)	1 (3)	1 (3)
Sweden	2 (2)	1 (1)	1 (1)	1 (1)	1 (1)	1 (2)	2 (2)
Swaziland	0 (4)	1 (4)	2 (8)	3 (9)	2 (9)	3 (9)	3 (9)
Syria	11 (7)	24 (11)	47 (15)	85 (22)	97 (23)	104 (22)	100 (23)
Chad	8 (8)	11 (9)	18 (10)	23 (9)	33 (10)	40 (9)	42 (8)
Togo	1 (1)	1 (2)	2 (2)	3 (2)	4 (2)	5 (3)	5 (3)
Thailand	21 (2)	29 (2)	32 (3)	19 (2)	15 (2)	12 (2)	11 (2)
Tajikistan	1 (1)	1 (1)	2 (2)	6 (3)	7 (4)	7 (4)	7 (4)
Turkmenistan	2 (4)	3 (4)	4 (4)	4 (4)	4 (4)	5 (4)	6 (4)
Timor-Leste	0 (0)	0 (1)	0 (1)	1 (3)	1 (3)	1 (3)	1 (3)
Trinidad and Tobago	1 (4)	1 (6)	3 (15)	4 (20)	3 (23)	4 (24)	3 (24)
Tunisia	4 (3)	6 (4)	12 (7)	13 (7)	10 (7)	11 (7)	14 (8)
Turkey	24 (3)	84 (8)	131 (11)	180 (15)	184 (16)	187 (17)	180 (17)
Tanzania	1 (0)	5 (1)	18 (3)	28 (3)	38 (4)	52 (4)	74 (4)
Uganda	3 (1)	6 (2)	11 (2)	15 (2)	21 (2)	29 (2)	36 (2)
Ukraine	20 (3)	17 (3)	24 (3)	24 (4)	16 (4)	18 (4)	17 (4)
United States	171 (4)	235 (7)	231 (7)	249 (7)	241 (6)	314 (8)	315 (8)
Uzbekistan	3 (1)	5 (1)	10 (2)	31 (5)	27 (5)	27 (5)	26 (5)
Saint Vincent and the Grenadines	0 (0)	0 (0)	0 (0)	0 (0)	0 (2)	0 (0)	0 (0)
Venezuela	20 (8)	22 (7)	41 (11)	51 (11)	55 (11)	55 (11)	53 (10)

United States Virgin Islands	0 (7)	0 (7)	0 (5)	0 (5)	0 (5)	0 (0)	0 (0)
Vietnam	8 (1)	10 (1)	14 (1)	18 (1)	14 (1)	13 (1)	12 (1)
Vanuatu	0 (0)	0 (0)	0 (0)	0 (6)	0 (6)	0 (7)	0 (7)
Samoa	0 (4)	0 (5)	0 (5)	0 (5)	0 (2)	0 (0)	0 (0)
Yemen	20 (12)	25 (11)	37 (11)	49 (10)	52 (9)	58 (8)	67 (8)
South Africa	23 (4)	37 (5)	61 (7)	79 (8)	79 (8)	78 (8)	77 (8)
Zambia	6 (5)	17 (11)	29 (13)	40 (14)	54 (15)	70 (15)	80 (14)
Zimbabwe	15 (10)	26 (13)	41 (14)	49 (14)	50 (14)	57 (14)	45 (12)

Table 8: **Absolute population (in thousands) of cohorts living unprecedented exposure to droughts and $CF_{droughts}$ (%) per country and birth year in a 2.5°C pathway**

Country	1960	1970	1980	1990	2000	2010	2020
Afghanistan	13 (4)	23 (5)	33 (6)	40 (8)	91 (10)	165 (14)	213 (16)
Angola	2 (1)	3 (1)	3 (1)	6 (2)	8 (2)	24 (4)	37 (6)
Albania	0 (0)	0 (0)	0 (1)	1 (2)	1 (2)	1 (4)	2 (6)
United Arab Emirates	0 (12)	0 (14)	2 (14)	3 (14)	3 (15)	4 (15)	5 (16)
Argentina	4 (1)	6 (1)	9 (1)	10 (1)	14 (2)	19 (3)	21 (3)
Armenia	11 (18)	13 (24)	16 (26)	17 (22)	9 (22)	11 (24)	11 (27)
Australia	3 (1)	6 (2)	8 (4)	14 (6)	18 (8)	24 (9)	28 (9)
Austria	6 (5)	6 (5)	5 (5)	5 (6)	5 (5)	7 (9)	8 (10)
Azerbaijan	8 (7)	9 (8)	10 (9)	15 (10)	13 (12)	16 (16)	18 (16)
Burundi	0 (0)	0 (0)	0 (0)	1 (0)	1 (1)	8 (3)	10 (3)
Belgium	0 (0)	0 (0)	0 (0)	0 (0)	0 (0)	1 (0)	3 (2)
Benin	2 (4)	3 (4)	2 (2)	5 (3)	9 (4)	14 (6)	20 (6)
Burkina Faso	9 (6)	12 (6)	22 (8)	36 (10)	53 (12)	91 (15)	116 (17)
Bangladesh	30 (2)	23 (1)	27 (1)	49 (2)	53 (2)	52 (2)	51 (2)
Bulgaria	4 (3)	5 (4)	9 (7)	12 (11)	8 (12)	10 (14)	15 (23)
Bahamas	0 (11)	0 (11)	0 (6)	0 (6)	0 (5)	0 (3)	0 (5)
Bosnia and Herzegovina	5 (5)	3 (4)	4 (5)	5 (7)	3 (7)	3 (8)	3 (10)
Belarus	2 (1)	2 (1)	4 (2)	4 (2)	2 (3)	3 (3)	7 (7)
Belize	0 (0)	0 (0)	0 (1)	0 (2)	0 (2)	0 (2)	0 (5)
Bolivia	4 (4)	8 (6)	12 (7)	18 (9)	24 (11)	31 (13)	32 (14)
Brazil	11 (0)	33 (1)	76 (2)	140 (4)	172 (6)	166 (6)	181 (7)
Brunei Darussalam	0 (0)	0 (0)	0 (0)	0 (0)	0 (0)	0 (0)	0 (0)
Bhutan	0 (0)	0 (0)	0 (1)	1 (3)	1 (4)	1 (5)	1 (7)
Botswana	1 (5)	2 (7)	3 (7)	3 (6)	4 (8)	5 (12)	6 (15)
Central African Republic	0 (1)	0 (1)	1 (1)	1 (1)	1 (1)	2 (2)	4 (3)
Canada	14 (3)	11 (3)	11 (3)	15 (4)	16 (5)	19 (6)	22 (6)
Switzerland	1 (1)	1 (2)	1 (2)	1 (2)	1 (2)	1 (2)	1 (1)
Chile	26 (13)	41 (18)	54 (25)	81 (33)	80 (35)	86 (39)	84 (40)
China	380 (2)	801 (3)	666 (3)	1068 (4)	952 (6)	1147 (7)	1190 (9)
Cote dIvoire	7 (5)	16 (7)	30 (9)	40 (9)	48 (8)	67 (10)	111 (16)
Cameroon	4 (2)	5 (2)	7 (2)	10 (2)	16 (3)	25 (4)	30 (4)

Democratic Republic of the Congo	26 (5)	39 (6)	45 (5)	54 (4)	90 (6)	153 (7)	203 (8)
Republic of Congo	1 (2)	1 (1)	1 (1)	4 (2)	7 (2)	14 (5)	15 (5)
Colombia	3 (1)	3 (0)	2 (0)	4 (0)	6 (1)	7 (1)	10 (1)
Cape Verde	0 (10)	0 (10)	0 (17)	0 (20)	0 (20)	0 (0)	0 (0)
Costa Rica	1 (2)	1 (2)	1 (2)	1 (1)	1 (1)	1 (1)	1 (1)
Cuba	1 (1)	4 (2)	3 (3)	3 (3)	3 (3)	4 (5)	5 (6)
Cyprus	1 (24)	1 (26)	1 (31)	1 (37)	1 (37)	2 (41)	2 (43)
Czech Republic	6 (5)	7 (6)	13 (8)	12 (10)	9 (11)	14 (13)	15 (14)
Germany	24 (2)	28 (2)	17 (2)	15 (2)	13 (2)	26 (4)	28 (4)
Djibouti	0 (12)	0 (10)	0 (7)	0 (3)	0 (4)	0 (5)	1 (5)
Denmark	0 (0)	0 (0)	0 (0)	0 (0)	0 (0)	1 (2)	1 (2)
Dominican Republic	0 (0)	2 (2)	5 (3)	6 (4)	8 (5)	9 (5)	8 (5)
Algeria	28 (7)	52 (10)	90 (13)	133 (18)	130 (22)	185 (25)	198 (25)
Ecuador	1 (0)	1 (0)	1 (0)	1 (0)	2 (1)	2 (1)	3 (1)
Egypt	131 (15)	171 (16)	213 (17)	298 (18)	283 (19)	374 (21)	438 (22)
Eritrea	5 (10)	2 (4)	3 (4)	3 (3)	4 (4)	8 (5)	14 (8)
Spain	43 (8)	64 (12)	74 (14)	58 (17)	60 (20)	88 (23)	76 (24)
Estonia	0 (1)	0 (1)	0 (0)	0 (0)	0 (0)	0 (0)	0 (1)
Ethiopia	8 (1)	10 (1)	17 (1)	27 (1)	54 (2)	75 (3)	113 (4)
Finland	1 (1)	1 (1)	0 (1)	0 (1)	0 (1)	0 (1)	1 (2)
France	14 (2)	17 (2)	15 (2)	23 (3)	24 (4)	35 (5)	39 (5)
United Kingdom	27 (4)	27 (3)	17 (3)	19 (3)	10 (1)	12 (2)	17 (2)
Georgia	12 (14)	14 (16)	14 (17)	15 (17)	11 (20)	14 (24)	12 (23)
Ghana	15 (7)	27 (9)	28 (8)	34 (7)	46 (8)	58 (9)	85 (11)
Guinea	0 (0)	1 (1)	5 (4)	10 (6)	19 (7)	27 (9)	34 (11)
The Gambia	1 (11)	1 (12)	2 (13)	4 (13)	5 (13)	7 (15)	7 (15)
Guinea-Bissau	1 (10)	1 (9)	2 (13)	3 (15)	5 (19)	7 (24)	7 (23)
Equatorial Guinea	0 (0)	0 (0)	0 (0)	0 (1)	0 (1)	0 (0)	0 (0)
Greece	1 (1)	4 (4)	7 (6)	9 (11)	13 (16)	16 (20)	16 (23)
Guatemala	3 (2)	6 (3)	12 (5)	11 (4)	17 (5)	20 (5)	28 (7)

Guyana	2 (12)	3 (15)	4 (23)	4 (26)	5 (30)	4 (29)	4 (29)
Honduras	5 (7)	6 (7)	9 (8)	12 (8)	13 (7)	14 (8)	17 (11)
Croatia	5 (6)	2 (3)	3 (5)	4 (7)	4 (9)	4 (10)	4 (13)
Haiti	0 (0)	1 (1)	4 (2)	7 (3)	7 (3)	8 (3)	6 (3)
Hungary	14 (9)	10 (7)	15 (8)	11 (9)	10 (10)	11 (11)	13 (14)
Indonesia	21 (1)	32 (1)	58 (2)	51 (1)	57 (2)	93 (2)	83 (2)
India	130 (1)	152 (1)	250 (1)	402 (2)	712 (3)	1026 (4)	1082 (5)
Ireland	0 (0)	0 (0)	0 (0)	0 (0)	0 (0)	0 (0)	1 (2)
Iran	91 (12)	113 (12)	158 (12)	238 (13)	200 (16)	230 (19)	237 (19)
Iraq	30 (12)	51 (13)	69 (14)	99 (16)	154 (19)	202 (20)	233 (20)
Israel	8 (21)	10 (24)	15 (27)	20 (30)	30 (35)	41 (37)	48 (37)
Italy	9 (1)	15 (2)	23 (4)	36 (8)	40 (9)	55 (12)	50 (12)
Jordan	2 (5)	10 (17)	17 (22)	28 (26)	38 (29)	48 (33)	49 (33)
Japan	4 (0)	8 (1)	5 (0)	4 (0)	4 (0)	5 (1)	5 (1)
Kazakhstan	3 (1)	2 (1)	2 (1)	2 (0)	1 (1)	5 (1)	13 (4)
Kenya	1 (0)	1 (0)	6 (1)	8 (1)	20 (2)	39 (3)	48 (4)
Kyrgyzstan	2 (2)	2 (3)	2 (2)	1 (1)	4 (3)	6 (5)	6 (4)
Cambodia	1 (0)	0 (0)	3 (2)	11 (3)	10 (3)	10 (3)	7 (2)
Republic of Korea	1 (0)	5 (1)	6 (1)	5 (1)	7 (1)	4 (1)	5 (2)
Kuwait	1 (17)	3 (19)	4 (19)	5 (19)	5 (22)	6 (22)	6 (22)
Lao PDR	0 (1)	1 (1)	2 (2)	2 (2)	3 (2)	4 (3)	3 (3)
Lebanon	6 (10)	8 (13)	11 (16)	19 (31)	21 (33)	14 (32)	20 (35)
Liberia	0 (0)	0 (0)	1 (1)	1 (2)	1 (2)	2 (2)	5 (4)
Libya	4 (10)	7 (10)	13 (13)	20 (18)	20 (19)	27 (25)	38 (32)
Sri Lanka	1 (1)	2 (1)	2 (1)	0 (0)	1 (0)	1 (0)	1 (0)
Lesotho	1 (4)	2 (5)	3 (7)	3 (7)	4 (7)	6 (10)	6 (11)
Lithuania	1 (1)	1 (1)	1 (1)	1 (1)	0 (1)	0 (1)	3 (9)
Latvia	1 (2)	0 (1)	0 (0)	0 (0)	0 (1)	0 (1)	1 (6)
Morocco	43 (13)	88 (21)	127 (27)	160 (31)	150 (33)	199 (38)	201 (39)
Moldova	0 (1)	1 (1)	1 (1)	0 (1)	0 (1)	1 (2)	1 (3)
Madagascar	1 (1)	3 (1)	5 (2)	21 (5)	47 (9)	90 (15)	138 (20)
Mexico	205 (15)	302 (17)	373 (17)	364 (17)	398 (18)	399 (19)	413 (20)
Macedonia	0 (1)	0 (1)	2 (5)	2 (7)	2 (8)	2 (10)	4 (17)
Mali	10 (6)	14 (7)	25 (9)	34 (10)	53 (12)	88 (15)	105 (15)
Myanmar	5 (1)	4 (1)	6 (1)	13 (1)	8 (1)	9 (1)	22 (3)
Montenegro	0 (0)	0 (0)	0 (0)	0 (2)	0 (2)	0 (2)	0 (2)
Mongolia	3 (10)	5 (10)	7 (11)	8 (11)	6 (13)	7 (12)	6 (10)
Mozambique	6 (3)	7 (2)	9 (2)	11 (2)	23 (4)	51 (7)	81 (11)
Mauritania	4 (11)	5 (12)	8 (14)	11 (17)	15 (18)	20 (19)	21 (19)

Malawi	5 (5)	8 (5)	13 (6)	19 (6)	26 (7)	72 (14)	87 (14)
Malaysia	22 (10)	28 (11)	32 (11)	13 (4)	20 (5)	13 (4)	14 (4)
Namibia	1 (5)	1 (5)	2 (6)	2 (6)	3 (6)	4 (7)	4 (7)
Niger	5 (4)	9 (5)	14 (6)	24 (8)	52 (11)	79 (12)	111 (13)
Nigeria	33 (2)	45 (2)	62 (2)	74 (2)	113 (3)	195 (4)	273 (4)
Nicaragua	7 (9)	8 (9)	11 (8)	13 (9)	12 (9)	11 (8)	10 (8)
Netherlands	2 (1)	2 (1)	2 (1)	2 (2)	2 (1)	3 (2)	3 (2)
Norway	2 (3)	1 (2)	0 (0)	0 (0)	0 (0)	1 (1)	2 (3)
Nepal	0 (0)	0 (0)	2 (0)	5 (1)	9 (1)	11 (1)	18 (2)
New Zealand	0 (0)	0 (1)	1 (1)	2 (3)	2 (4)	2 (4)	2 (4)
Oman	2 (16)	3 (16)	6 (17)	8 (16)	6 (14)	6 (13)	7 (13)
Pakistan	48 (3)	69 (4)	111 (4)	203 (6)	259 (7)	314 (7)	321 (7)
Panama	1 (4)	2 (6)	2 (6)	1 (3)	0 (1)	0 (0)	1 (1)
Peru	6 (2)	10 (3)	14 (3)	18 (4)	22 (5)	23 (5)	25 (6)
Philippines	38 (5)	48 (6)	53 (5)	50 (4)	61 (4)	69 (5)	67 (4)
Papua New Guinea	1 (2)	1 (2)	1 (2)	1 (1)	3 (2)	5 (3)	4 (3)
Poland	8 (1)	9 (2)	18 (3)	17 (3)	15 (4)	18 (5)	26 (7)
Puerto Rico	3 (4)	3 (6)	3 (4)	3 (6)	3 (5)	3 (7)	2 (6)
Dem. Rep. Korea	3 (1)	6 (2)	5 (2)	7 (2)	18 (6)	16 (6)	10 (4)
Portugal	10 (7)	14 (11)	21 (16)	22 (25)	23 (27)	22 (29)	20 (29)
Paraguay	2 (4)	3 (3)	3 (4)	4 (3)	4 (3)	8 (7)	11 (10)
Qatar	0 (22)	1 (22)	1 (17)	1 (15)	2 (17)	2 (20)	3 (22)
Romania	23 (6)	24 (6)	37 (9)	33 (9)	21 (10)	25 (11)	29 (15)
Russian Federation	54 (2)	37 (2)	31 (2)	21 (1)	12 (1)	23 (1)	29 (2)
Rwanda	0 (0)	0 (0)	0 (0)	0 (0)	0 (0)	1 (0)	1 (0)
Saudi Arabia	16 (12)	27 (14)	53 (16)	82 (17)	77 (17)	105 (18)	113 (18)
Sudan	41 (13)	57 (13)	75 (13)	99 (14)	130 (15)	173 (16)	201 (16)
Senegal	18 (15)	25 (16)	40 (19)	52 (20)	65 (20)	77 (18)	73 (15)
Solomon Islands	0 (1)	0 (1)	0 (1)	0 (0)	0 (0)	0 (1)	0 (1)
Sierra Leone	0 (0)	0 (0)	0 (0)	1 (1)	3 (2)	8 (5)	14 (8)
El Salvador	3 (3)	4 (3)	6 (4)	5 (3)	4 (3)	4 (3)	5 (5)
Somalia	1 (1)	1 (2)	3 (2)	4 (2)	5 (2)	9 (3)	11 (4)

Serbia	7 (6)	4 (4)	5 (5)	7 (7)	6 (7)	6 (8)	9 (13)
South Sudan	1 (2)	2 (2)	4 (3)	6 (3)	8 (3)	6 (2)	9 (3)
Sao Tome and Principe	0 (5)	0 (5)	0 (5)	0 (5)	0 (5)	0 (0)	0 (0)
Suriname	2 (15)	2 (14)	2 (20)	2 (21)	2 (23)	2 (23)	2 (20)
Slovakia	4 (4)	4 (5)	5 (5)	6 (6)	4 (7)	5 (8)	6 (9)
Slovenia	0 (1)	0 (0)	0 (0)	0 (2)	1 (4)	1 (4)	1 (5)
Sweden	1 (2)	1 (1)	0 (0)	0 (0)	0 (0)	1 (1)	2 (2)
Swaziland	0 (2)	0 (3)	1 (3)	1 (4)	1 (4)	3 (11)	5 (17)
Syria	11 (6)	24 (11)	62 (20)	102 (27)	134 (31)	161 (33)	163 (37)
Chad	7 (7)	11 (8)	16 (9)	18 (7)	27 (8)	43 (10)	53 (11)
Togo	0 (1)	1 (1)	1 (1)	1 (1)	3 (2)	5 (2)	8 (4)
Thailand	22 (2)	32 (3)	36 (3)	13 (1)	10 (1)	9 (1)	8 (1)
Tajikistan	1 (1)	1 (1)	3 (2)	1 (1)	3 (2)	11 (6)	14 (8)
Turkmenistan	2 (4)	3 (4)	5 (6)	6 (5)	6 (6)	9 (7)	11 (8)
Timor-Leste	0 (0)	0 (1)	0 (1)	0 (2)	1 (3)	1 (4)	1 (3)
Trinidad and Tobago	1 (5)	1 (7)	2 (8)	2 (10)	1 (10)	2 (11)	2 (16)
Tunisia	3 (3)	7 (5)	12 (7)	18 (9)	21 (14)	26 (17)	30 (18)
Turkey	28 (3)	74 (7)	134 (11)	216 (18)	259 (22)	298 (26)	308 (28)
Tanzania	1 (0)	2 (0)	6 (1)	10 (1)	21 (2)	57 (4)	93 (5)
Uganda	2 (1)	3 (1)	4 (1)	5 (1)	10 (1)	30 (2)	39 (2)
Ukraine	18 (2)	16 (2)	20 (3)	17 (3)	12 (3)	16 (4)	22 (5)
United States	148 (4)	130 (4)	148 (5)	197 (5)	271 (7)	340 (9)	356 (9)
Uzbekistan	4 (1)	7 (2)	10 (2)	9 (1)	20 (4)	48 (9)	56 (10)
Saint Vincent and the Grenadines	0 (0)	0 (0)	0 (0)	0 (2)	0 (2)	0 (0)	0 (0)
Venezuela	17 (7)	20 (6)	28 (7)	41 (8)	52 (10)	54 (10)	58 (11)
United States Virgin Islands	0 (12)	0 (7)	0 (2)	0 (2)	0 (7)	0 (0)	0 (0)
Vietnam	7 (1)	10 (1)	12 (1)	13 (1)	15 (1)	15 (1)	15 (1)
Samoa	0 (5)	0 (5)	0 (5)	0 (5)	0 (2)	0 (0)	0 (0)
Yemen	19 (12)	25 (11)	41 (13)	68 (14)	77 (13)	90 (13)	111 (13)

South Africa	14 (3)	23 (3)	33 (4)	41 (4)	43 (5)	80 (8)	89 (9)
Zambia	6 (5)	12 (7)	22 (10)	29 (10)	43 (11)	80 (17)	103 (18)
Zimbabwe	12 (8)	19 (9)	33 (11)	39 (11)	40 (11)	60 (15)	67 (18)

Table 9: **Absolute population (in thousands) of cohorts living unprecedented exposure to droughts and $\mathbf{CF_{droughts}}$ (%) per country and birth year in a 3.5°C pathway**

Country	1960	1970	1980	1990	2000	2010	2020
Afghanistan	18 (5)	28 (6)	42 (8)	49 (9)	115 (12)	173 (15)	228 (17)
Angola	2 (1)	2 (1)	3 (1)	6 (2)	10 (2)	22 (3)	26 (4)
Albania	0 (0)	0 (0)	0 (0)	1 (1)	1 (3)	3 (7)	2 (6)
United Arab Emirates	0 (14)	0 (14)	2 (14)	2 (13)	2 (12)	4 (14)	5 (15)
Argentina	4 (1)	5 (1)	8 (1)	9 (1)	11 (2)	13 (2)	19 (3)
Armenia	7 (12)	10 (17)	12 (19)	15 (19)	6 (17)	8 (19)	11 (25)
Australia	1 (1)	4 (2)	9 (4)	19 (8)	23 (10)	31 (12)	35 (12)
Austria	4 (3)	4 (3)	3 (3)	7 (8)	10 (12)	13 (16)	13 (16)
Azerbaijan	3 (3)	4 (3)	5 (5)	9 (6)	8 (7)	7 (8)	10 (9)
Burundi	0 (0)	0 (0)	0 (0)	3 (1)	3 (2)	5 (2)	6 (2)
Belgium	0 (0)	0 (0)	0 (0)	0 (0)	0 (0)	1 (1)	1 (1)
Benin	2 (2)	2 (2)	2 (2)	5 (3)	8 (4)	9 (4)	12 (4)
Burkina Faso	6 (4)	7 (3)	15 (5)	24 (7)	45 (10)	76 (13)	93 (13)
Bangladesh	29 (2)	20 (1)	25 (1)	31 (1)	35 (1)	32 (1)	30 (1)
Bulgaria	2 (2)	3 (2)	5 (4)	11 (10)	9 (14)	14 (19)	15 (23)
Bahamas	0 (8)	0 (8)	0 (2)	0 (1)	0 (3)	0 (2)	0 (2)
Bosnia and Herzegovina	2 (3)	1 (2)	2 (2)	2 (3)	2 (5)	2 (5)	2 (6)
Belarus	1 (0)	1 (0)	1 (1)	1 (1)	1 (1)	1 (1)	1 (1)
Belize	0 (1)	0 (0)	0 (1)	0 (1)	0 (3)	0 (6)	0 (6)
Bolivia	4 (4)	8 (6)	11 (6)	16 (8)	17 (8)	31 (13)	38 (16)
Brazil	14 (1)	22 (1)	47 (2)	72 (2)	88 (3)	124 (5)	148 (6)
Brunei Darussalam	0 (0)	0 (0)	0 (0)	0 (0)	0 (0)	0 (0)	0 (0)
Bhutan	0 (0)	0 (0)	0 (0)	0 (2)	1 (5)	2 (8)	2 (12)
Botswana	1 (5)	2 (7)	3 (9)	4 (10)	5 (11)	8 (18)	12 (26)
Central African Republic	0 (0)	0 (0)	0 (0)	1 (1)	1 (1)	2 (2)	3 (3)
Canada	12 (3)	10 (3)	10 (3)	11 (3)	11 (3)	12 (4)	14 (4)
Switzerland	0 (0)	0 (0)	0 (0)	1 (1)	3 (5)	4 (6)	4 (6)
Chile	16 (8)	31 (13)	45 (21)	67 (27)	79 (34)	82 (37)	83 (40)
China	539 (3)	890 (3)	684 (4)	1029 (4)	856 (5)	872 (5)	904 (7)
Cote dIvoire	7 (5)	14 (7)	24 (7)	36 (8)	44 (8)	70 (11)	69 (10)
Cameroon	2 (1)	2 (1)	3 (1)	8 (2)	15 (3)	20 (3)	22 (3)

Democratic Republic of the Congo	13 (2)	19 (3)	17 (2)	29 (2)	42 (3)	70 (3)	102 (4)
Republic of Congo	0 (1)	1 (1)	0 (0)	1 (0)	1 (0)	2 (1)	13 (4)
Colombia	4 (1)	5 (1)	8 (1)	7 (1)	11 (1)	13 (2)	14 (2)
Cape Verde	0 (8)	0 (12)	0 (20)	1 (24)	1 (24)	0 (0)	0 (0)
Costa Rica	1 (2)	1 (2)	1 (2)	1 (1)	1 (1)	1 (1)	0 (0)
Cuba	0 (0)	0 (0)	1 (1)	1 (1)	2 (2)	2 (2)	2 (3)
Cyprus	1 (12)	1 (14)	1 (24)	1 (25)	1 (27)	1 (29)	1 (28)
Czech Republic	5 (3)	4 (4)	6 (4)	5 (5)	7 (8)	12 (11)	13 (12)
Germany	17 (1)	19 (2)	14 (2)	21 (2)	19 (2)	35 (5)	39 (5)
Djibouti	0 (15)	0 (12)	0 (12)	0 (6)	0 (6)	1 (7)	1 (8)
Denmark	0 (0)	0 (0)	0 (0)	0 (0)	1 (1)	1 (1)	1 (1)
Dominican Republic	0 (0)	3 (2)	5 (3)	5 (3)	16 (9)	17 (10)	15 (9)
Algeria	19 (5)	34 (7)	70 (11)	132 (17)	143 (24)	232 (31)	279 (36)
Ecuador	1 (0)	1 (0)	2 (1)	2 (1)	10 (4)	12 (4)	12 (4)
Egypt	139 (16)	176 (17)	226 (18)	343 (21)	323 (22)	420 (24)	528 (27)
Eritrea	4 (7)	1 (2)	2 (2)	2 (2)	3 (3)	8 (5)	11 (6)
Spain	24 (5)	31 (6)	51 (10)	49 (14)	63 (20)	91 (24)	80 (25)
Ethiopia	5 (1)	9 (1)	15 (1)	26 (1)	48 (2)	81 (3)	94 (3)
Finland	0 (1)	0 (0)	0 (0)	0 (1)	0 (1)	1 (2)	1 (2)
France	8 (1)	9 (1)	6 (1)	10 (1)	11 (2)	20 (3)	20 (3)
United Kingdom	29 (4)	28 (3)	14 (2)	28 (4)	23 (4)	44 (6)	45 (6)
Georgia	8 (9)	8 (9)	10 (12)	12 (14)	8 (14)	8 (15)	10 (19)
Ghana	11 (5)	18 (6)	20 (5)	28 (6)	45 (8)	63 (9)	55 (7)
Guinea	1 (1)	1 (1)	4 (3)	8 (4)	20 (8)	34 (12)	35 (11)
The Gambia	1 (18)	2 (20)	4 (21)	6 (21)	9 (24)	11 (23)	12 (25)
Guinea-Bissau	2 (16)	2 (16)	3 (18)	5 (21)	6 (25)	8 (30)	11 (36)
Equatorial Guinea	0 (0)	0 (0)	0 (0)	0 (0)	0 (0)	0 (2)	0 (2)
Greece	1 (0)	2 (2)	6 (6)	8 (9)	9 (12)	16 (20)	16 (23)
Guatemala	4 (3)	8 (4)	12 (5)	13 (4)	24 (7)	25 (7)	35 (9)
Guyana	3 (15)	5 (25)	7 (36)	7 (43)	7 (44)	6 (43)	6 (51)

Honduras	7 (11)	10 (11)	13 (11)	18 (12)	20 (12)	21 (12)	21 (13)
Croatia	3 (4)	1 (1)	1 (2)	2 (4)	5 (10)	4 (11)	4 (11)
Haiti	0 (0)	0 (0)	7 (4)	5 (2)	7 (3)	10 (4)	8 (3)
Hungary	7 (4)	6 (4)	9 (5)	7 (5)	8 (8)	12 (12)	11 (12)
Indonesia	13 (0)	19 (1)	31 (1)	18 (0)	23 (1)	31 (1)	38 (1)
India	91 (1)	108 (1)	169 (1)	306 (1)	424 (2)	646 (3)	750 (3)
Iran	104 (14)	125 (13)	188 (14)	291 (16)	229 (19)	256 (21)	325 (26)
Iraq	24 (10)	39 (10)	65 (13)	85 (14)	152 (19)	211 (21)	281 (24)
Israel	5 (13)	7 (17)	13 (22)	22 (34)	33 (38)	50 (45)	71 (54)
Italy	6 (1)	8 (1)	17 (3)	21 (5)	32 (7)	54 (12)	56 (14)
Jordan	1 (5)	7 (12)	16 (21)	30 (29)	47 (36)	55 (39)	62 (42)
Japan	3 (0)	3 (0)	0 (0)	3 (0)	4 (0)	6 (1)	5 (1)
Kazakhstan	2 (1)	2 (1)	2 (1)	2 (1)	1 (1)	2 (1)	6 (2)
Kenya	1 (0)	1 (0)	1 (0)	2 (0)	5 (0)	12 (1)	24 (2)
Kyrgyzstan	3 (4)	4 (4)	4 (4)	3 (3)	6 (6)	10 (7)	7 (5)
Cambodia	0 (0)	0 (0)	2 (1)	4 (1)	4 (1)	7 (2)	9 (3)
Republic of Korea	1 (0)	2 (0)	5 (1)	5 (1)	7 (1)	4 (1)	4 (1)
Kuwait	1 (16)	3 (20)	4 (20)	5 (20)	6 (24)	8 (28)	9 (32)
Lao PDR	0 (1)	1 (1)	2 (2)	3 (2)	4 (3)	5 (3)	7 (5)
Lebanon	3 (5)	6 (9)	9 (13)	15 (25)	17 (26)	16 (36)	24 (42)
Liberia	0 (0)	0 (0)	0 (0)	0 (0)	2 (2)	5 (5)	8 (7)
Libya	4 (9)	7 (9)	16 (16)	25 (23)	27 (27)	30 (27)	36 (30)
Sri Lanka	1 (0)	1 (0)	1 (1)	0 (0)	0 (0)	2 (1)	1 (1)
Lesotho	2 (6)	3 (9)	5 (12)	6 (12)	6 (10)	8 (14)	9 (14)
Lithuania	0 (0)	0 (0)	0 (0)	1 (1)	0 (1)	0 (1)	1 (2)
Latvia	0 (0)	0 (0)	0 (0)	0 (1)	0 (1)	0 (0)	0 (1)
Morocco	31 (9)	62 (15)	91 (19)	128 (25)	150 (33)	203 (39)	225 (43)
Moldova	1 (1)	0 (1)	1 (1)	1 (1)	2 (5)	2 (6)	2 (5)
Madagascar	1 (0)	2 (1)	6 (2)	17 (4)	40 (8)	69 (11)	110 (16)
Mexico	223 (16)	336 (18)	397 (18)	397 (18)	424 (19)	410 (19)	396 (19)
Macedonia	0 (0)	1 (2)	1 (2)	2 (6)	3 (11)	3 (17)	4 (19)
Mali	8 (5)	13 (6)	20 (8)	27 (8)	48 (11)	86 (14)	103 (15)
Myanmar	8 (1)	5 (1)	8 (1)	10 (1)	12 (1)	12 (1)	13 (2)
Montenegro	0 (0)	0 (0)	0 (0)	0 (0)	0 (0)	0 (2)	0 (3)
Mongolia	4 (11)	5 (11)	7 (12)	8 (12)	5 (10)	6 (11)	7 (11)
Mozambique	7 (3)	9 (3)	13 (3)	17 (4)	21 (4)	43 (6)	61 (8)
Mauritania	5 (15)	7 (16)	10 (19)	13 (19)	17 (20)	22 (21)	25 (22)
Malawi	9 (7)	13 (8)	21 (9)	26 (8)	35 (9)	57 (11)	75 (12)
Malaysia	17 (8)	19 (8)	25 (9)	11 (3)	8 (2)	2 (1)	2 (0)

Namibia	1 (6)	2 (7)	3 (8)	3 (8)	4 (8)	5 (8)	6 (9)
Niger	5 (3)	7 (4)	6 (2)	12 (4)	32 (7)	54 (8)	72 (9)
Nigeria	13 (1)	9 (0)	11 (0)	33 (1)	55 (1)	116 (2)	148 (2)
Nicaragua	12 (15)	12 (14)	14 (11)	15 (11)	17 (12)	13 (10)	13 (11)
Netherlands	1 (1)	1 (1)	1 (1)	1 (1)	1 (1)	2 (1)	2 (1)
Norway	3 (5)	2 (4)	0 (0)	0 (1)	1 (2)	1 (2)	1 (2)
Nepal	0 (0)	1 (0)	2 (0)	4 (1)	6 (1)	11 (1)	14 (2)
Oman	3 (19)	4 (21)	8 (22)	8 (16)	6 (13)	7 (13)	8 (15)
Pakistan	57 (4)	86 (4)	146 (6)	230 (6)	265 (7)	343 (8)	423 (9)
Panama	1 (5)	2 (7)	3 (7)	2 (4)	1 (1)	1 (1)	0 (0)
Peru	8 (3)	13 (4)	20 (5)	25 (6)	28 (6)	32 (7)	30 (7)
Philippines	20 (3)	26 (3)	15 (1)	9 (1)	12 (1)	11 (1)	13 (1)
Papua New Guinea	1 (2)	1 (2)	2 (2)	2 (2)	3 (3)	5 (3)	4 (3)
Poland	6 (1)	4 (1)	9 (1)	13 (2)	13 (3)	19 (5)	22 (6)
Puerto Rico	2 (3)	2 (4)	2 (3)	3 (6)	4 (8)	3 (8)	3 (8)
Dem. Rep. Korea	2 (1)	2 (0)	1 (0)	1 (0)	5 (1)	10 (4)	12 (5)
Portugal	5 (3)	7 (5)	12 (9)	13 (15)	19 (23)	20 (26)	19 (27)
Paraguay	2 (2)	2 (2)	3 (3)	4 (3)	5 (4)	5 (4)	8 (7)
Qatar	0 (20)	0 (16)	1 (12)	1 (12)	1 (12)	1 (12)	2 (12)
Romania	18 (5)	21 (5)	21 (5)	23 (7)	20 (9)	27 (13)	26 (14)
Russian Federation	30 (1)	20 (1)	19 (1)	15 (1)	10 (1)	17 (1)	26 (2)
Rwanda	0 (0)	0 (0)	0 (0)	0 (0)	0 (0)	1 (0)	4 (1)
Saudi Arabia	14 (11)	24 (12)	48 (15)	84 (17)	77 (17)	106 (18)	126 (20)
Sudan	26 (8)	36 (8)	54 (9)	83 (12)	126 (15)	179 (17)	214 (17)
Senegal	22 (19)	33 (21)	48 (23)	58 (22)	74 (23)	102 (24)	116 (25)
Solomon Islands	0 (1)	0 (1)	0 (1)	0 (0)	0 (0)	0 (1)	0 (1)
Sierra Leone	0 (0)	0 (0)	0 (0)	1 (1)	2 (1)	12 (7)	13 (7)
El Salvador	5 (5)	5 (4)	7 (5)	7 (5)	7 (5)	6 (5)	5 (5)
Somalia	1 (1)	1 (1)	2 (1)	3 (1)	4 (2)	5 (2)	6 (2)
Serbia	3 (2)	2 (2)	3 (3)	5 (5)	7 (8)	7 (10)	8 (11)
South Sudan	1 (1)	1 (1)	2 (1)	3 (2)	4 (2)	5 (2)	8 (3)
Suriname	2 (14)	2 (17)	2 (27)	2 (24)	2 (24)	3 (28)	4 (42)

Slovakia	3 (3)	2 (2)	2 (2)	3 (3)	5 (8)	7 (12)	7 (12)
Slovenia	0 (1)	0 (0)	0 (1)	0 (2)	1 (7)	2 (8)	2 (10)
Sweden	1 (1)	1 (1)	0 (0)	1 (1)	1 (2)	2 (2)	3 (3)
Swaziland	0 (3)	1 (5)	1 (3)	1 (4)	1 (4)	4 (14)	5 (17)
Syria	8 (5)	23 (10)	54 (17)	96 (25)	124 (29)	148 (31)	159 (36)
Chad	4 (4)	5 (4)	7 (4)	10 (4)	22 (7)	33 (7)	39 (8)
Togo	0 (1)	0 (1)	1 (1)	1 (1)	2 (1)	3 (2)	4 (2)
Thailand	17 (2)	24 (2)	26 (2)	11 (1)	10 (1)	11 (1)	15 (2)
Tajikistan	1 (2)	2 (2)	4 (3)	7 (4)	8 (5)	9 (5)	8 (4)
Turkmenistan	2 (4)	4 (5)	6 (7)	8 (7)	6 (6)	9 (7)	12 (9)
Timor-Leste	0 (0)	0 (1)	0 (1)	1 (3)	2 (7)	3 (8)	4 (10)
Trinidad and Tobago	1 (4)	1 (6)	2 (12)	3 (16)	3 (22)	5 (30)	5 (36)
Tunisia	3 (2)	6 (4)	9 (5)	18 (9)	20 (13)	28 (18)	33 (20)
Turkey	24 (3)	50 (5)	115 (10)	166 (14)	190 (16)	239 (21)	325 (30)
Tanzania	1 (0)	2 (0)	3 (0)	6 (1)	11 (1)	34 (2)	83 (5)
Uganda	1 (1)	2 (0)	2 (0)	4 (1)	10 (1)	14 (1)	22 (1)
Ukraine	14 (2)	10 (2)	12 (2)	12 (2)	10 (2)	14 (3)	15 (4)
United States	102 (3)	94 (3)	115 (4)	182 (5)	237 (6)	287 (7)	319 (8)
Uzbekistan	5 (2)	7 (2)	16 (3)	29 (5)	25 (5)	27 (5)	28 (5)
Saint Vincent and the Grenadines	0 (0)	0 (0)	0 (4)	0 (4)	0 (16)	0 (0)	0 (0)
Venezuela	23 (9)	28 (9)	48 (12)	69 (14)	80 (16)	92 (18)	98 (19)
United States Virgin Islands	0 (16)	0 (12)	0 (12)	0 (8)	0 (8)	0 (0)	0 (0)
Vietnam	3 (0)	5 (0)	8 (1)	11 (1)	9 (1)	11 (1)	18 (1)
Samoa	0 (8)	0 (8)	0 (8)	0 (8)	0 (0)	0 (0)	0 (0)
Yemen	23 (14)	28 (13)	52 (16)	86 (18)	89 (15)	114 (16)	143 (16)
South Africa	22 (4)	36 (5)	54 (6)	64 (7)	66 (7)	106 (11)	127 (13)
Zambia	8 (7)	16 (10)	23 (10)	33 (12)	42 (11)	80 (17)	107 (19)
Zimbabwe	14 (9)	22 (11)	35 (12)	41 (12)	47 (13)	65 (16)	75 (20)

Table 10: **Absolute population (in thousands) of cohorts living unprecedented exposure to crop failures and $\mathbf{CF_{crop\ failures}}$ (%) per country and birth year in a 1.5°C pathway**

Country	1960	1970	1980	1990	2000	2010	2020
Afghanistan	4 (1)	10 (2)	14 (3)	14 (3)	25 (3)	31 (3)	37 (3)
Angola	9 (5)	14 (6)	32 (11)	46 (12)	68 (15)	102 (16)	103 (15)
Albania	5 (9)	5 (8)	12 (19)	19 (27)	15 (29)	10 (29)	14 (40)
United Arab Emirates	0 (11)	0 (17)	2 (19)	4 (19)	4 (19)	5 (20)	6 (20)
Argentina	10 (2)	28 (6)	39 (6)	50 (7)	51 (7)	55 (8)	72 (10)
Armenia	3 (5)	3 (5)	3 (5)	6 (8)	3 (8)	4 (8)	4 (8)
Australia	4 (2)	3 (1)	3 (1)	7 (3)	7 (3)	8 (3)	9 (3)
Austria	4 (3)	6 (5)	3 (3)	3 (3)	3 (4)	3 (4)	4 (5)
Azerbaijan	6 (5)	2 (2)	3 (3)	2 (2)	2 (2)	2 (2)	2 (2)
Burundi	6 (6)	10 (8)	17 (11)	19 (8)	39 (17)	56 (18)	67 (18)
Belgium	0 (0)	0 (0)	0 (0)	0 (0)	0 (0)	2 (1)	13 (8)
Benin	3 (4)	7 (8)	20 (16)	32 (20)	57 (28)	73 (28)	82 (27)
Burkina Faso	12 (8)	33 (16)	67 (25)	109 (31)	143 (31)	206 (35)	237 (34)
Bangladesh	0 (0)	0 (0)	0 (0)	10 (0)	39 (1)	81 (3)	41 (2)
Bulgaria	0 (0)	2 (1)	2 (2)	2 (2)	2 (3)	3 (4)	2 (3)
Bahamas	0 (9)	0 (14)	0 (18)	0 (17)	0 (12)	0 (12)	0 (22)
Bosnia and Herzegovina	7 (8)	5 (7)	7 (10)	8 (11)	4 (9)	4 (12)	5 (16)
Belarus	0 (0)	0 (0)	0 (0)	0 (0)	0 (0)	0 (0)	0 (0)
Belize	0 (10)	0 (11)	0 (15)	1 (18)	1 (18)	1 (18)	1 (14)
Bolivia	2 (2)	5 (4)	13 (7)	21 (10)	39 (17)	45 (19)	44 (19)
Brazil	94 (4)	164 (6)	339 (11)	583 (18)	661 (21)	572 (22)	577 (23)
Bhutan	0 (3)	1 (4)	1 (5)	3 (14)	3 (16)	3 (16)	3 (16)
Botswana	3 (13)	2 (9)	4 (10)	7 (14)	7 (15)	8 (19)	8 (19)
Central African Republic	5 (13)	9 (18)	15 (22)	22 (24)	25 (24)	35 (31)	41 (33)
Canada	8 (2)	20 (6)	26 (8)	27 (7)	27 (8)	31 (9)	36 (10)
Chile	2 (1)	14 (6)	14 (7)	17 (7)	11 (5)	14 (6)	13 (6)
China	1537 (8)	3043 (12)	2366 (12)	3382 (13)	2190 (14)	2210 (14)	2071 (15)
Cote dIvoire	20 (15)	53 (25)	87 (26)	154 (34)	234 (41)	270 (41)	291 (41)
Cameroon	34 (20)	66 (28)	109 (33)	149 (33)	209 (40)	272 (41)	286 (41)

Democratic Republic of the Congo	79 (15)	138 (20)	257 (28)	372 (30)	524 (32)	723 (32)	802 (30)
Republic of Congo	4 (10)	8 (9)	12 (9)	13 (7)	33 (12)	53 (18)	51 (17)
Colombia	26 (5)	42 (6)	60 (8)	78 (10)	89 (11)	101 (13)	105 (14)
Cape Verde	0 (0)	0 (0)	0 (13)	0 (13)	0 (7)	0 (0)	0 (0)
Costa Rica	2 (6)	4 (7)	4 (8)	1 (2)	1 (2)	1 (2)	5 (8)
Cuba	11 (9)	14 (8)	12 (11)	12 (10)	12 (11)	9 (11)	10 (13)
Germany	4 (0)	1 (0)	0 (0)	1 (0)	1 (0)	1 (0)	3 (0)
Djibouti	0 (0)	0 (0)	0 (0)	0 (0)	0 (0)	0 (1)	0 (2)
Denmark	0 (0)	0 (0)	1 (2)	2 (5)	3 (5)	2 (6)	2 (6)
Dominican Republic	4 (4)	5 (4)	6 (4)	7 (4)	8 (5)	8 (5)	9 (5)
Algeria	9 (2)	7 (1)	14 (2)	22 (3)	14 (2)	20 (3)	32 (4)
Ecuador	21 (13)	30 (15)	37 (15)	36 (13)	46 (16)	42 (14)	38 (13)
Egypt	171 (20)	475 (45)	673 (53)	960 (59)	881 (60)	1073 (62)	1192 (61)
Eritrea	2 (4)	2 (3)	3 (4)	5 (4)	5 (5)	16 (10)	18 (10)
Spain	17 (3)	17 (3)	15 (3)	18 (5)	18 (6)	23 (6)	26 (8)
Estonia	0 (2)	0 (0)	0 (0)	0 (0)	0 (0)	0 (0)	0 (0)
Ethiopia	34 (4)	72 (7)	149 (11)	265 (15)	459 (19)	532 (20)	590 (20)
Finland	0 (0)	1 (1)	1 (1)	1 (1)	1 (1)	1 (1)	1 (1)
Fiji	0 (0)	0 (0)	0 (1)	0 (1)	0 (1)	0 (1)	0 (1)
France	9 (1)	11 (1)	11 (2)	13 (2)	11 (2)	36 (5)	41 (6)
Gabon	1 (10)	2 (14)	3 (16)	2 (11)	2 (9)	3 (9)	3 (8)
United Kingdom	2 (0)	1 (0)	9 (1)	12 (2)	11 (2)	20 (3)	19 (3)
Georgia	2 (2)	2 (2)	1 (1)	1 (1)	1 (2)	1 (2)	1 (2)
Ghana	15 (6)	37 (12)	84 (23)	168 (37)	238 (43)	293 (43)	327 (43)
Guinea	9 (10)	21 (17)	32 (23)	57 (30)	87 (33)	115 (39)	134 (43)
The Gambia	1 (18)	2 (22)	4 (24)	8 (28)	11 (29)	13 (28)	11 (22)
Guinea-Bissau	1 (5)	1 (7)	3 (16)	6 (30)	8 (31)	8 (30)	9 (29)
Equatorial Guinea	1 (11)	1 (12)	1 (13)	1 (12)	1 (9)	2 (14)	2 (14)
Greece	3 (3)	3 (3)	5 (5)	5 (6)	6 (7)	6 (8)	6 (9)
Guatemala	22 (14)	42 (22)	79 (31)	111 (38)	127 (36)	136 (36)	166 (42)
Guyana	0 (0)	1 (3)	1 (3)	1 (6)	3 (17)	2 (19)	3 (22)

Honduras	12 (17)	21 (22)	37 (30)	46 (30)	59 (34)	58 (33)	56 (34)
Croatia	2 (2)	2 (4)	4 (6)	5 (9)	4 (8)	4 (10)	7 (19)
Haiti	5 (4)	10 (8)	13 (8)	2 (1)	6 (3)	7 (3)	6 (3)
Hungary	11 (6)	6 (4)	7 (4)	1 (1)	0 (0)	1 (1)	8 (9)
Indonesia	90 (3)	186 (6)	277 (7)	345 (9)	384 (11)	437 (11)	428 (12)
India	835 (6)	1438 (9)	1986 (10)	3186 (13)	4323 (17)	4677 (19)	4369 (19)
Iran	11 (1)	20 (2)	26 (2)	41 (2)	33 (3)	35 (3)	40 (3)
Iraq	26 (10)	119 (30)	173 (35)	238 (39)	331 (41)	391 (39)	460 (40)
Israel	2 (4)	3 (7)	7 (12)	12 (19)	21 (24)	26 (23)	31 (24)
Italy	53 (8)	51 (7)	42 (7)	33 (7)	32 (8)	40 (9)	39 (10)
Jamaica	3 (8)	6 (12)	7 (16)	8 (18)	6 (14)	5 (13)	5 (13)
Jordan	1 (2)	5 (9)	12 (16)	25 (23)	30 (23)	33 (23)	34 (23)
Japan	33 (2)	31 (2)	14 (1)	14 (1)	27 (3)	25 (3)	29 (4)
Kazakhstan	2 (1)	3 (1)	5 (1)	6 (2)	4 (2)	12 (4)	17 (5)
Kenya	18 (6)	51 (12)	123 (19)	191 (22)	262 (25)	329 (26)	462 (34)
Kyrgyzstan	2 (2)	2 (3)	3 (3)	4 (3)	4 (3)	4 (3)	5 (3)
Cambodia	2 (1)	1 (0)	2 (1)	4 (1)	4 (1)	4 (1)	4 (1)
Republic of Korea	0 (0)	0 (0)	0 (0)	2 (0)	2 (0)	2 (1)	4 (1)
Lao PDR	0 (0)	1 (1)	4 (3)	7 (5)	10 (7)	10 (7)	9 (7)
Lebanon	1 (1)	0 (0)	0 (0)	1 (1)	1 (1)	1 (1)	1 (1)
Liberia	0 (0)	1 (2)	2 (4)	3 (5)	6 (8)	9 (10)	14 (12)
Libya	1 (3)	1 (1)	1 (1)	9 (8)	9 (9)	9 (9)	10 (9)
Sri Lanka	0 (0)	0 (0)	0 (0)	0 (0)	0 (0)	0 (0)	1 (1)
Lesotho	5 (18)	7 (20)	10 (24)	11 (21)	12 (22)	10 (18)	12 (20)
Lithuania	0 (1)	0 (0)	0 (0)	1 (1)	1 (2)	1 (2)	1 (2)
Luxembourg	0 (0)	0 (0)	0 (0)	0 (0)	0 (0)	0 (7)	0 (7)
Latvia	0 (0)	0 (1)	0 (1)	1 (3)	1 (4)	1 (5)	1 (5)
Morocco	19 (6)	38 (9)	80 (17)	91 (18)	83 (18)	93 (18)	95 (18)
Moldova	0 (1)	0 (0)	0 (1)	1 (1)	0 (1)	1 (4)	0 (1)
Madagascar	3 (2)	6 (3)	8 (3)	17 (4)	61 (12)	76 (12)	104 (15)
Mexico	320 (23)	597 (33)	914 (43)	951 (44)	1004 (45)	971 (46)	993 (47)
Macedonia	0 (0)	0 (0)	0 (0)	0 (0)	0 (1)	0 (2)	3 (14)
Mali	3 (2)	24 (11)	43 (16)	81 (25)	137 (32)	211 (35)	255 (37)
Myanmar	2 (0)	4 (0)	9 (1)	10 (1)	15 (2)	13 (1)	30 (4)
Montenegro	2 (16)	1 (7)	1 (12)	1 (10)	1 (8)	1 (8)	1 (9)
Mongolia	0 (1)	0 (1)	0 (0)	0 (1)	0 (1)	2 (3)	2 (3)
Mozambique	39 (16)	50 (17)	77 (20)	87 (20)	108 (19)	127 (18)	151 (20)
Mauritania	2 (5)	1 (2)	2 (4)	5 (7)	8 (10)	11 (11)	12 (11)
Malawi	31 (26)	55 (33)	95 (41)	135 (41)	167 (42)	187 (36)	221 (35)

Malaysia	18 (8)	31 (13)	39 (14)	45 (13)	50 (13)	44 (12)	48 (12)
Namibia	0 (1)	1 (4)	1 (4)	2 (4)	2 (4)	2 (4)	3 (4)
New Caledonia	0 (2)	0 (5)	0 (3)	0 (3)	0 (2)	0 (4)	0 (3)
Niger	6 (4)	10 (5)	35 (15)	59 (19)	99 (22)	140 (22)	191 (23)
Nigeria	107 (7)	239 (13)	471 (18)	645 (19)	929 (23)	1502 (28)	1746 (28)
Nicaragua	6 (8)	12 (13)	36 (29)	42 (29)	39 (28)	38 (29)	34 (27)
Netherlands	1 (0)	0 (0)	0 (0)	0 (0)	0 (0)	6 (4)	8 (6)
Norway	0 (0)	0 (0)	0 (0)	0 (0)	0 (0)	0 (1)	0 (1)
Nepal	14 (3)	38 (8)	61 (10)	149 (20)	247 (29)	242 (31)	244 (31)
New Zealand	3 (6)	3 (6)	1 (1)	0 (1)	2 (3)	2 (3)	3 (5)
Oman	0 (1)	0 (1)	1 (4)	3 (5)	3 (8)	4 (7)	4 (8)
Pakistan	354 (24)	520 (27)	848 (33)	1277 (36)	1469 (38)	1668 (39)	1790 (39)
Panama	2 (6)	4 (12)	5 (12)	7 (18)	8 (18)	9 (18)	10 (21)
Peru	15 (5)	30 (8)	50 (12)	79 (17)	89 (20)	85 (20)	81 (19)
Philippines	32 (5)	47 (6)	65 (6)	116 (9)	150 (10)	158 (11)	176 (12)
Papua New Guinea	3 (5)	5 (8)	10 (11)	16 (15)	25 (20)	34 (22)	33 (20)
Poland	0 (0)	1 (0)	2 (0)	2 (0)	1 (0)	1 (0)	4 (1)
Puerto Rico	0 (0)	0 (0)	0 (0)	0 (0)	0 (0)	1 (1)	0 (1)
Dem. Rep. Korea	28 (10)	43 (12)	39 (15)	50 (17)	56 (18)	48 (18)	46 (19)
Portugal	4 (3)	3 (3)	5 (4)	3 (4)	4 (5)	7 (9)	7 (10)
Paraguay	3 (4)	7 (9)	8 (9)	19 (16)	23 (18)	19 (17)	21 (18)
Qatar	0 (0)	0 (7)	0 (7)	1 (7)	1 (13)	1 (13)	2 (13)
Romania	10 (3)	8 (2)	6 (1)	7 (2)	9 (4)	11 (5)	10 (5)
Russian Federation	25 (1)	15 (1)	9 (0)	13 (1)	10 (1)	19 (1)	22 (1)
Rwanda	7 (5)	19 (12)	37 (16)	46 (15)	73 (25)	116 (30)	116 (26)
Saudi Arabia	4 (3)	14 (7)	28 (9)	51 (10)	50 (11)	65 (11)	72 (12)
Sudan	50 (16)	83 (20)	150 (26)	214 (30)	250 (29)	328 (30)	371 (30)
Senegal	14 (12)	23 (15)	45 (21)	63 (24)	80 (25)	105 (25)	117 (25)
Solomon Islands	0 (1)	0 (1)	0 (1)	0 (2)	0 (2)	0 (0)	0 (4)
Sierra Leone	0 (0)	0 (0)	2 (2)	7 (6)	10 (8)	14 (9)	17 (10)
El Salvador	4 (4)	23 (18)	38 (26)	40 (28)	42 (29)	37 (31)	33 (30)

Somalia	1 (1)	2 (2)	10 (6)	14 (7)	21 (9)	30 (11)	34 (11)
Serbia	8 (7)	5 (5)	5 (4)	4 (4)	1 (2)	2 (3)	8 (12)
South Sudan	6 (7)	19 (15)	37 (23)	72 (33)	103 (37)	101 (37)	112 (36)
Sao Tome and Principe	0 (13)	0 (13)	0 (13)	1 (13)	1 (13)	0 (0)	0 (0)
Suriname	0 (0)	0 (0)	0 (1)	0 (0)	0 (1)	0 (1)	0 (1)
Slovakia	2 (2)	1 (1)	1 (1)	0 (0)	0 (0)	0 (0)	0 (0)
Slovenia	1 (5)	1 (3)	0 (2)	0 (1)	0 (1)	1 (6)	4 (15)
Sweden	0 (0)	0 (0)	1 (1)	1 (2)	1 (2)	2 (2)	2 (2)
Swaziland	3 (28)	4 (28)	5 (25)	7 (26)	7 (27)	10 (35)	12 (43)
Syria	1 (0)	1 (0)	3 (1)	28 (7)	32 (7)	36 (7)	39 (9)
Chad	16 (16)	25 (20)	48 (28)	76 (32)	111 (33)	155 (35)	161 (32)
Togo	2 (4)	4 (5)	12 (12)	17 (14)	27 (17)	38 (19)	38 (20)
Thailand	4 (0)	16 (1)	27 (2)	40 (4)	43 (5)	37 (5)	39 (5)
Tajikistan	1 (1)	0 (0)	1 (1)	2 (1)	2 (1)	2 (1)	2 (1)
Turkmenistan	0 (0)	1 (2)	2 (2)	3 (2)	2 (2)	2 (2)	3 (2)
Timor-Leste	0 (0)	0 (0)	0 (1)	0 (1)	0 (1)	0 (1)	2 (6)
Trinidad and Tobago	0 (0)	0 (0)	0 (0)	1 (3)	1 (6)	1 (6)	1 (6)
Tunisia	5 (4)	11 (7)	13 (7)	14 (7)	12 (8)	13 (9)	19 (11)
Turkey	10 (1)	14 (1)	43 (4)	52 (4)	53 (5)	62 (5)	66 (6)
Tanzania	10 (3)	19 (4)	48 (8)	71 (9)	128 (13)	172 (12)	210 (12)
Uganda	18 (7)	29 (8)	37 (7)	71 (10)	95 (9)	123 (9)	153 (9)
Ukraine	9 (1)	39 (6)	43 (6)	40 (6)	26 (6)	30 (7)	33 (8)
Uruguay	0 (0)	0 (0)	0 (0)	1 (1)	1 (1)	1 (1)	1 (1)
United States	69 (2)	140 (4)	174 (5)	213 (6)	259 (7)	333 (8)	389 (10)
Uzbekistan	1 (0)	3 (1)	4 (1)	12 (2)	16 (3)	27 (5)	39 (7)
Venezuela	84 (34)	125 (39)	177 (45)	253 (52)	298 (59)	311 (60)	312 (60)
Vietnam	14 (1)	24 (2)	94 (7)	119 (7)	98 (8)	99 (8)	117 (9)
Vanuatu	0 (0)	0 (4)	0 (7)	0 (7)	0 (9)	0 (13)	0 (13)
Samoa	0 (0)	0 (7)	0 (7)	0 (7)	0 (13)	0 (0)	0 (0)
Yemen	28 (17)	50 (23)	91 (28)	144 (29)	169 (29)	209 (29)	237 (27)
South Africa	61 (11)	98 (14)	158 (18)	200 (20)	188 (20)	209 (21)	203 (21)
Zambia	24 (21)	44 (27)	68 (30)	84 (29)	106 (28)	142 (30)	164 (28)
Zimbabwe	22 (15)	44 (22)	70 (24)	79 (22)	86 (23)	99 (24)	88 (23)

Table 11: **Absolute population (in thousands) of cohorts living unprecedented exposure to crop failures and $CF_{\text{crop failures}}$ (%) per country and birth year in a 2.5°C pathway**

Country	1960	1970	1980	1990	2000	2010	2020
Afghanistan	4 (1)	7 (2)	14 (3)	15 (3)	28 (3)	73 (6)	104 (8)
Angola	9 (5)	14 (6)	27 (10)	44 (12)	65 (14)	125 (20)	163 (24)
Albania	3 (6)	3 (6)	6 (10)	9 (13)	10 (21)	8 (23)	11 (30)
United Arab Emirates	0 (6)	0 (19)	3 (24)	5 (25)	5 (25)	8 (27)	9 (27)
Argentina	9 (2)	11 (2)	17 (2)	16 (2)	18 (3)	32 (4)	38 (5)
Armenia	5 (7)	4 (7)	4 (7)	5 (7)	2 (6)	2 (5)	2 (6)
Australia	4 (2)	3 (1)	4 (2)	2 (1)	9 (4)	13 (5)	16 (6)
Austria	3 (3)	2 (1)	2 (3)	2 (3)	2 (3)	2 (3)	4 (4)
Azerbaijan	6 (5)	2 (1)	1 (1)	1 (1)	0 (0)	0 (0)	0 (0)
Burundi	5 (5)	7 (6)	33 (22)	47 (21)	50 (22)	76 (25)	124 (33)
Belgium	0 (0)	0 (0)	0 (0)	0 (0)	1 (1)	1 (1)	3 (2)
Benin	2 (2)	7 (7)	20 (17)	34 (21)	58 (28)	103 (39)	123 (40)
Burkina Faso	10 (6)	14 (7)	56 (21)	91 (26)	217 (48)	294 (50)	362 (52)
Bangladesh	0 (0)	0 (0)	0 (0)	0 (0)	0 (0)	14 (0)	13 (0)
Bulgaria	1 (1)	0 (0)	0 (0)	0 (0)	0 (0)	0 (0)	0 (0)
Bahamas	0 (6)	0 (6)	0 (17)	0 (26)	0 (27)	0 (30)	0 (30)
Bosnia and Herzegovina	7 (7)	5 (6)	6 (8)	12 (18)	9 (19)	7 (20)	8 (25)
Belarus	0 (0)	0 (0)	0 (0)	0 (0)	0 (0)	0 (0)	0 (0)
Belize	0 (13)	0 (16)	0 (14)	1 (25)	1 (27)	1 (27)	2 (41)
Bolivia	2 (2)	4 (3)	11 (6)	26 (13)	44 (19)	52 (22)	65 (27)
Brazil	65 (3)	81 (3)	185 (6)	327 (10)	394 (13)	393 (15)	421 (17)
Bhutan	0 (2)	0 (3)	1 (4)	4 (18)	5 (24)	4 (19)	4 (21)
Botswana	2 (12)	1 (4)	1 (3)	0 (1)	1 (3)	3 (7)	2 (5)
Central African Republic	5 (12)	8 (15)	14 (20)	20 (22)	26 (25)	41 (35)	57 (46)
Canada	9 (2)	7 (2)	8 (2)	10 (3)	11 (3)	24 (7)	35 (9)
Chile	6 (3)	13 (6)	13 (6)	17 (7)	24 (10)	24 (11)	25 (12)
China	1394 (8)	2985 (11)	2393 (12)	3572 (14)	2279 (14)	2482 (16)	2219 (16)
Cote dIvoire	14 (11)	31 (15)	79 (24)	151 (34)	188 (33)	278 (42)	355 (51)
Cameroon	28 (16)	64 (27)	106 (32)	162 (36)	204 (39)	275 (41)	311 (45)

Democratic Republic of the Congo	68 (13)	103 (15)	157 (17)	255 (21)	408 (25)	724 (32)	927 (35)
Republic of Congo	4 (8)	7 (8)	11 (8)	29 (16)	49 (17)	55 (18)	57 (19)
Colombia	18 (3)	34 (5)	54 (7)	69 (9)	79 (10)	99 (13)	111 (15)
Costa Rica	2 (4)	2 (5)	3 (5)	1 (2)	2 (2)	2 (3)	2 (3)
Cuba	16 (13)	31 (18)	24 (22)	25 (20)	25 (24)	31 (36)	30 (38)
Czech Republic	0 (0)	0 (0)	0 (0)	0 (0)	0 (0)	0 (0)	5 (5)
Germany	3 (0)	0 (0)	0 (0)	0 (0)	0 (0)	0 (0)	3 (0)
Denmark	0 (0)	0 (0)	0 (0)	0 (0)	3 (6)	3 (6)	3 (6)
Dominican Republic	7 (7)	11 (8)	14 (9)	18 (11)	37 (21)	39 (22)	40 (24)
Algeria	11 (3)	12 (2)	30 (4)	33 (4)	25 (4)	32 (4)	34 (4)
Ecuador	20 (12)	20 (10)	33 (13)	39 (14)	43 (15)	54 (18)	70 (24)
Egypt	82 (10)	314 (30)	577 (45)	975 (60)	1050 (72)	1334 (77)	1584 (81)
Eritrea	2 (4)	1 (2)	3 (3)	5 (4)	7 (7)	15 (9)	27 (15)
Spain	22 (4)	20 (4)	17 (3)	15 (4)	21 (7)	32 (8)	31 (10)
Estonia	0 (3)	0 (0)	0 (0)	0 (0)	0 (0)	0 (0)	0 (3)
Ethiopia	36 (5)	82 (8)	133 (10)	325 (18)	637 (26)	796 (30)	952 (33)
Fiji	0 (0)	0 (0)	0 (0)	0 (0)	0 (0)	0 (5)	0 (5)
France	17 (2)	18 (2)	17 (3)	16 (2)	11 (2)	39 (5)	57 (8)
Gabon	1 (10)	2 (13)	3 (18)	4 (17)	4 (18)	8 (26)	11 (33)
United Kingdom	9 (1)	2 (0)	5 (1)	9 (1)	13 (2)	21 (3)	31 (4)
Georgia	2 (2)	1 (2)	1 (2)	2 (2)	2 (3)	2 (3)	2 (3)
Ghana	10 (4)	22 (7)	62 (17)	112 (24)	182 (33)	333 (49)	468 (61)
Guinea	8 (9)	16 (13)	25 (18)	45 (24)	74 (28)	102 (35)	139 (45)
The Gambia	1 (22)	2 (22)	4 (22)	8 (27)	10 (26)	13 (28)	13 (26)
Guinea-Bissau	0 (0)	0 (3)	1 (5)	3 (14)	6 (25)	9 (30)	9 (30)
Equatorial Guinea	1 (9)	1 (9)	1 (13)	1 (12)	2 (11)	3 (19)	3 (21)
Greece	2 (2)	3 (2)	4 (4)	5 (6)	6 (7)	7 (8)	8 (11)
Guatemala	22 (14)	42 (22)	82 (32)	123 (41)	172 (49)	179 (48)	210 (53)
Guyana	0 (1)	0 (1)	0 (0)	0 (1)	0 (3)	0 (3)	1 (5)
Honduras	10 (15)	19 (20)	38 (31)	42 (28)	59 (34)	62 (35)	71 (43)
Croatia	6 (9)	4 (6)	3 (5)	8 (15)	7 (13)	9 (23)	10 (30)

Haiti	7 (6)	8 (6)	19 (11)	21 (10)	36 (16)	41 (18)	63 (28)
Hungary	19 (11)	10 (6)	9 (5)	5 (4)	0 (0)	0 (0)	4 (5)
Indonesia	77 (3)	159 (5)	289 (8)	364 (10)	404 (11)	550 (14)	568 (16)
India	721 (5)	1393 (8)	2372 (12)	3860 (16)	4750 (19)	5402 (22)	5140 (23)
Iran	22 (3)	33 (4)	38 (3)	64 (4)	64 (5)	70 (6)	83 (7)
Iraq	38 (15)	111 (28)	203 (40)	320 (52)	493 (62)	659 (66)	788 (69)
Israel	1 (3)	4 (10)	6 (11)	19 (28)	28 (33)	41 (37)	51 (38)
Italy	46 (7)	46 (6)	39 (7)	30 (7)	43 (10)	59 (13)	57 (14)
Jamaica	3 (7)	8 (15)	8 (18)	11 (26)	11 (27)	10 (26)	10 (27)
Jordan	1 (4)	10 (16)	11 (14)	22 (21)	29 (23)	35 (24)	35 (24)
Japan	29 (2)	27 (2)	8 (1)	2 (0)	1 (0)	1 (0)	1 (0)
Kazakhstan	4 (1)	3 (1)	5 (2)	8 (2)	7 (3)	16 (5)	21 (6)
Kenya	21 (7)	40 (9)	82 (13)	117 (14)	161 (16)	253 (20)	312 (23)
Kyrgyzstan	1 (1)	2 (2)	3 (3)	8 (6)	7 (6)	9 (7)	15 (11)
Cambodia	1 (1)	1 (0)	1 (1)	3 (1)	3 (1)	2 (1)	2 (1)
Lao PDR	0 (0)	0 (0)	0 (0)	1 (1)	4 (3)	4 (3)	4 (3)
Lebanon	1 (2)	0 (0)	0 (0)	0 (0)	0 (0)	0 (0)	0 (0)
Liberia	0 (0)	0 (1)	1 (1)	1 (3)	3 (4)	3 (4)	7 (6)
Libya	1 (2)	1 (1)	1 (1)	5 (4)	4 (4)	9 (8)	18 (15)
Sri Lanka	0 (0)	0 (0)	0 (0)	0 (0)	0 (0)	0 (0)	3 (1)
Lesotho	4 (14)	6 (17)	7 (16)	6 (12)	8 (14)	9 (16)	11 (18)
Lithuania	1 (2)	0 (0)	0 (0)	0 (0)	0 (0)	0 (0)	1 (2)
Morocco	27 (8)	46 (11)	85 (18)	142 (28)	134 (29)	175 (33)	190 (36)
Moldova	0 (1)	0 (0)	0 (0)	0 (0)	0 (0)	0 (0)	0 (0)
Madagascar	1 (1)	3 (1)	9 (3)	22 (6)	46 (9)	80 (13)	100 (14)
Mexico	305 (22)	517 (28)	749 (35)	840 (39)	1007 (45)	1008 (47)	1072 (51)
Mali	2 (1)	11 (5)	35 (13)	64 (20)	135 (32)	248 (41)	313 (45)
Myanmar	1 (0)	1 (0)	5 (0)	7 (1)	8 (1)	10 (1)	12 (2)
Montenegro	1 (8)	1 (12)	2 (20)	1 (16)	1 (16)	1 (19)	1 (21)
Mongolia	0 (1)	0 (1)	0 (0)	0 (0)	0 (1)	1 (1)	1 (1)
Mozambique	38 (16)	51 (17)	75 (19)	83 (19)	118 (20)	160 (22)	251 (33)
Mauritania	1 (4)	1 (2)	2 (4)	6 (9)	13 (16)	16 (16)	20 (18)
Malawi	40 (33)	61 (37)	101 (43)	163 (49)	191 (48)	301 (57)	383 (60)
Malaysia	23 (11)	28 (11)	36 (13)	41 (12)	43 (11)	34 (10)	37 (9)
Namibia	0 (1)	0 (1)	0 (1)	0 (0)	0 (1)	0 (1)	1 (1)
New Caledonia	0 (0)	0 (1)	0 (1)	0 (13)	0 (19)	0 (23)	0 (23)
Niger	6 (4)	12 (7)	50 (21)	85 (27)	153 (34)	235 (36)	324 (39)
Nigeria	101 (7)	211 (11)	463 (17)	750 (23)	1188 (29)	2009 (37)	2630 (42)
Nicaragua	9 (12)	16 (17)	27 (21)	41 (29)	45 (32)	45 (35)	53 (43)
Netherlands	1 (1)	0 (0)	0 (0)	0 (0)	2 (2)	3 (2)	5 (3)

Nepal	16 (4)	22 (5)	41 (7)	163 (22)	281 (33)	316 (40)	345 (44)
New Zealand	3 (5)	3 (5)	1 (2)	1 (2)	3 (6)	4 (6)	5 (8)
Oman	0 (1)	0 (1)	1 (2)	4 (9)	5 (11)	7 (13)	10 (20)
Pakistan	359 (24)	618 (32)	971 (38)	1639 (46)	1983 (51)	2289 (53)	2544 (55)
Panama	2 (5)	4 (12)	8 (21)	10 (23)	14 (32)	16 (34)	18 (38)
Peru	12 (4)	24 (7)	55 (13)	92 (20)	117 (26)	122 (28)	122 (29)
Philippines	24 (3)	31 (4)	61 (6)	72 (6)	125 (9)	181 (13)	217 (14)
Papua New Guinea	2 (4)	5 (8)	8 (10)	14 (14)	21 (16)	33 (21)	37 (23)
Poland	0 (0)	0 (0)	0 (0)	0 (0)	0 (0)	0 (0)	30 (8)
Dem. Rep. Korea	38 (13)	41 (11)	30 (12)	43 (14)	44 (14)	35 (13)	26 (11)
Portugal	5 (3)	4 (3)	5 (4)	3 (3)	3 (3)	3 (4)	3 (4)
Paraguay	5 (7)	6 (8)	12 (13)	25 (21)	31 (24)	31 (27)	42 (36)
Qatar	0 (0)	0 (0)	0 (0)	0 (0)	1 (11)	2 (22)	3 (23)
Romania	10 (3)	5 (1)	4 (1)	3 (1)	2 (1)	5 (2)	6 (3)
Russian Federation	28 (1)	12 (1)	6 (0)	6 (0)	4 (0)	8 (0)	17 (1)
Rwanda	4 (3)	8 (5)	51 (22)	66 (22)	85 (29)	120 (31)	111 (25)
Saudi Arabia	3 (3)	11 (6)	27 (8)	46 (9)	50 (11)	73 (12)	81 (13)
Sudan	41 (13)	73 (17)	121 (21)	212 (30)	311 (36)	440 (41)	532 (43)
Senegal	14 (12)	16 (11)	37 (18)	54 (20)	73 (22)	75 (18)	82 (17)
Sierra Leone	0 (0)	0 (0)	0 (0)	1 (1)	3 (2)	4 (2)	3 (2)
El Salvador	12 (12)	24 (19)	36 (25)	38 (27)	44 (30)	36 (31)	44 (39)
Somalia	0 (0)	1 (1)	2 (1)	4 (2)	9 (4)	32 (11)	40 (13)
Serbia	8 (8)	4 (3)	3 (2)	6 (6)	3 (3)	2 (3)	7 (10)
South Sudan	6 (6)	14 (11)	26 (16)	62 (28)	93 (33)	118 (43)	140 (45)
Sao Tome and Principe	0 (11)	0 (11)	0 (11)	0 (11)	0 (11)	0 (0)	0 (0)
Slovakia	2 (2)	1 (1)	0 (0)	0 (0)	0 (0)	0 (0)	0 (0)
Slovenia	1 (4)	1 (3)	0 (1)	0 (1)	0 (1)	2 (8)	11 (45)
Sweden	0 (0)	0 (0)	1 (1)	2 (3)	3 (4)	4 (5)	5 (6)
Swaziland	2 (19)	1 (10)	3 (13)	2 (8)	3 (11)	9 (32)	11 (37)
Syria	2 (1)	1 (0)	3 (1)	5 (1)	12 (3)	14 (3)	21 (5)

Chad	14 (14)	18 (14)	35 (20)	65 (27)	109 (33)	172 (39)	211 (42)
Togo	1 (2)	2 (2)	5 (6)	13 (10)	25 (16)	65 (33)	64 (33)
Thailand	4 (0)	11 (1)	38 (3)	39 (4)	40 (4)	40 (5)	46 (6)
Tajikistan	1 (2)	0 (0)	1 (1)	2 (1)	4 (2)	4 (2)	4 (2)
Turkmenistan	0 (1)	1 (2)	2 (3)	3 (3)	3 (3)	3 (3)	5 (4)
Timor-Leste	0 (0)	0 (0)	0 (0)	0 (1)	1 (2)	2 (6)	4 (11)
Trinidad and Tobago	0 (0)	0 (0)	0 (0)	0 (0)	0 (0)	0 (0)	1 (6)
Tunisia	5 (4)	11 (7)	6 (4)	10 (5)	11 (7)	14 (9)	16 (9)
Turkey	11 (1)	11 (1)	16 (1)	15 (1)	18 (1)	27 (2)	33 (3)
Tanzania	9 (3)	17 (4)	29 (5)	48 (6)	81 (8)	178 (12)	239 (14)
Uganda	9 (4)	16 (4)	22 (4)	46 (6)	102 (10)	200 (15)	304 (19)
Ukraine	14 (2)	1 (0)	3 (0)	3 (0)	2 (0)	7 (1)	12 (3)
Uruguay	0 (0)	0 (0)	0 (0)	0 (0)	0 (0)	0 (0)	0 (1)
United States	71 (2)	70 (2)	96 (3)	142 (4)	215 (6)	283 (7)	377 (9)
Uzbekistan	2 (1)	6 (2)	8 (2)	21 (3)	28 (5)	49 (9)	74 (13)
Saint Vincent and the Grenadines	0 (0)	0 (0)	0 (0)	0 (11)	0 (22)	0 (0)	0 (0)
Venezuela	81 (33)	122 (38)	176 (45)	271 (56)	312 (62)	338 (65)	345 (67)
Vietnam	11 (1)	19 (1)	40 (3)	66 (4)	62 (5)	78 (6)	72 (6)
Vanuatu	0 (0)	0 (3)	0 (7)	0 (11)	0 (11)	0 (22)	0 (22)
Samoa	0 (0)	0 (0)	0 (0)	0 (11)	0 (0)	0 (0)	0 (0)
Yemen	28 (17)	45 (21)	97 (31)	175 (36)	226 (38)	271 (38)	350 (40)
South Africa	45 (8)	63 (9)	91 (10)	111 (11)	114 (12)	160 (16)	170 (17)
Zambia	23 (20)	44 (27)	66 (30)	80 (28)	102 (27)	143 (30)	182 (31)
Zimbabwe	15 (10)	18 (9)	21 (7)	20 (6)	22 (6)	58 (14)	81 (21)

Table 12: **Absolute population (in thousands) of cohorts living unprecedented exposure to crop failures and $CF_{crop\ failures}$ (%) per country and birth year in a 3.5°C pathway**

Country	1960	1970	1980	1990	2000	2010	2020
Afghanistan	8 (2)	14 (3)	18 (3)	13 (3)	39 (4)	57 (5)	124 (9)
Angola	6 (4)	10 (4)	14 (5)	28 (8)	53 (12)	121 (19)	176 (26)
Albania	3 (6)	5 (8)	10 (16)	22 (31)	16 (31)	15 (42)	16 (45)
United Arab Emirates	0 (17)	0 (18)	2 (20)	5 (28)	5 (28)	6 (21)	9 (30)
Argentina	8 (2)	10 (2)	12 (2)	14 (2)	100 (14)	123 (17)	133 (19)
Armenia	2 (3)	2 (3)	2 (3)	2 (3)	1 (3)	0 (0)	0 (0)
Australia	1 (0)	1 (0)	2 (1)	3 (1)	3 (1)	4 (2)	4 (1)
Austria	0 (0)	0 (0)	7 (8)	7 (8)	7 (8)	7 (9)	11 (13)
Azerbaijan	0 (0)	0 (0)	0 (0)	0 (0)	0 (0)	0 (0)	1 (1)
Burundi	0 (0)	0 (0)	34 (22)	54 (24)	86 (38)	156 (51)	226 (61)
Belgium	0 (0)	0 (0)	0 (0)	0 (0)	0 (0)	0 (0)	0 (0)
Benin	0 (0)	5 (5)	26 (22)	59 (37)	97 (47)	178 (68)	235 (77)
Burkina Faso	5 (3)	8 (4)	88 (33)	179 (51)	293 (64)	427 (72)	580 (83)
Bahamas	0 (18)	0 (19)	0 (29)	0 (30)	0 (30)	0 (33)	0 (33)
Bosnia and Herzegovina	0 (0)	0 (0)	0 (0)	0 (0)	0 (0)	3 (8)	1 (4)
Belize	0 (8)	1 (38)	1 (36)	1 (29)	2 (45)	3 (66)	4 (73)
Bolivia	1 (1)	2 (1)	7 (4)	18 (9)	37 (16)	55 (23)	76 (32)
Brazil	26 (1)	47 (2)	179 (6)	273 (9)	402 (13)	458 (18)	676 (27)
Bhutan	0 (2)	0 (2)	1 (6)	1 (6)	1 (6)	1 (6)	1 (7)
Botswana	4 (20)	2 (8)	4 (9)	2 (5)	2 (4)	8 (18)	8 (19)
Central African Republic	4 (10)	7 (13)	12 (17)	24 (27)	31 (29)	58 (50)	81 (65)
Canada	7 (2)	7 (2)	8 (2)	18 (5)	17 (5)	41 (12)	43 (11)
Chile	1 (0)	1 (0)	1 (0)	3 (1)	3 (1)	3 (1)	2 (1)
China	1375 (8)	2837 (11)	2457 (13)	3668 (14)	2412 (15)	2789 (18)	2597 (19)
Cote dIvoire	24 (18)	39 (19)	132 (40)	262 (59)	394 (69)	598 (90)	667 (95)
Cameroon	22 (13)	59 (25)	107 (32)	150 (33)	196 (37)	252 (38)	359 (51)
Democratic Republic of the Congo	52 (10)	70 (10)	127 (14)	270 (22)	591 (36)	1167 (51)	1572 (59)

Republic of Congo	2 (5)	7 (9)	15 (12)	17 (10)	30 (11)	36 (12)	35 (12)
Colombia	6 (1)	50 (7)	97 (13)	120 (15)	195 (25)	238 (31)	274 (36)
Costa Rica	0 (0)	0 (0)	1 (1)	4 (6)	9 (13)	11 (16)	11 (17)
Cuba	3 (2)	22 (13)	32 (28)	50 (40)	73 (69)	68 (80)	62 (80)
Germany	0 (0)	0 (0)	0 (0)	0 (0)	0 (0)	1 (0)	2 (0)
Denmark	0 (0)	0 (0)	5 (10)	7 (16)	9 (18)	8 (18)	7 (18)
Dominican Republic	0 (0)	3 (2)	4 (2)	7 (4)	29 (17)	54 (31)	52 (31)
Algeria	5 (1)	7 (1)	12 (2)	13 (2)	30 (5)	74 (10)	115 (15)
Ecuador	24 (15)	25 (12)	39 (16)	50 (18)	58 (20)	63 (21)	81 (28)
Egypt	14 (2)	398 (38)	835 (65)	1160 (71)	1146 (78)	1565 (90)	1867 (95)
Eritrea	0 (1)	1 (2)	2 (2)	5 (4)	6 (6)	15 (9)	39 (22)
Spain	17 (3)	2 (0)	7 (1)	11 (3)	15 (5)	37 (10)	45 (14)
Ethiopia	22 (3)	58 (6)	152 (11)	401 (22)	895 (37)	1212 (46)	1519 (52)
France	1 (0)	1 (0)	1 (0)	3 (0)	5 (1)	10 (1)	23 (3)
Gabon	1 (7)	1 (7)	2 (10)	2 (10)	3 (15)	8 (26)	13 (42)
United Kingdom	2 (0)	2 (0)	2 (0)	22 (3)	26 (4)	32 (5)	43 (6)
Georgia	0 (0)	0 (0)	0 (1)	1 (2)	1 (2)	1 (2)	2 (4)
Ghana	18 (8)	33 (11)	93 (26)	266 (58)	386 (69)	651 (96)	761 (100)
Guinea	8 (9)	15 (12)	32 (23)	49 (26)	113 (43)	174 (60)	214 (69)
The Gambia	4 (67)	7 (67)	11 (67)	21 (73)	28 (73)	34 (73)	36 (73)
Guinea-Bissau	0 (0)	0 (1)	1 (6)	4 (20)	16 (64)	24 (87)	28 (87)
Equatorial Guinea	0 (6)	1 (7)	1 (8)	1 (7)	2 (12)	2 (18)	3 (18)
Greece	3 (3)	3 (3)	4 (4)	5 (6)	5 (6)	7 (9)	11 (16)
Guatemala	31 (20)	47 (24)	101 (40)	194 (65)	252 (71)	280 (75)	317 (80)
Guyana	0 (1)	0 (1)	0 (1)	0 (2)	1 (7)	1 (7)	2 (12)
Honduras	24 (35)	45 (48)	74 (60)	108 (70)	123 (70)	131 (75)	128 (78)
Croatia	0 (0)	0 (0)	0 (0)	0 (0)	1 (2)	4 (9)	4 (10)
Haiti	5 (4)	1 (1)	79 (47)	35 (16)	36 (16)	78 (34)	120 (55)
Indonesia	58 (2)	198 (6)	265 (7)	471 (12)	575 (16)	806 (21)	947 (27)
India	214 (2)	340 (2)	745 (4)	1734 (7)	2576 (10)	4008 (16)	4696 (21)
Ireland	0 (0)	0 (0)	1 (0)	0 (0)	0 (0)	0 (0)	0 (0)
Iran	17 (2)	15 (2)	15 (1)	55 (3)	48 (4)	64 (5)	115 (9)
Iraq	4 (1)	55 (14)	92 (18)	226 (37)	452 (57)	703 (71)	855 (74)
Israel	1 (3)	1 (2)	1 (2)	8 (12)	30 (34)	56 (50)	88 (67)
Italy	47 (7)	52 (7)	52 (9)	40 (9)	68 (16)	110 (24)	152 (37)
Jamaica	9 (20)	11 (23)	12 (27)	11 (26)	11 (26)	10 (26)	10 (26)

Jordan	0 (0)	0 (0)	10 (13)	21 (20)	25 (19)	30 (21)	30 (21)
Japan	84 (6)	82 (5)	20 (1)	6 (1)	4 (0)	2 (0)	51 (7)
Kazakhstan	0 (0)	0 (0)	1 (0)	7 (2)	8 (4)	17 (5)	32 (10)
Kenya	29 (9)	31 (7)	50 (8)	62 (7)	58 (6)	138 (11)	185 (14)
Kyrgyzstan	2 (3)	3 (3)	5 (5)	7 (6)	16 (15)	18 (13)	24 (17)
Cambodia	0 (0)	0 (0)	0 (0)	0 (0)	2 (0)	5 (2)	9 (3)
Lao PDR	0 (0)	0 (0)	0 (0)	0 (0)	2 (2)	10 (7)	12 (9)
Lebanon	0 (0)	0 (0)	0 (0)	0 (0)	0 (0)	0 (0)	7 (12)
Liberia	0 (0)	1 (1)	0 (1)	1 (2)	2 (2)	5 (5)	9 (8)
Libya	2 (5)	1 (1)	1 (1)	2 (2)	4 (4)	11 (10)	17 (14)
Lesotho	10 (38)	14 (43)	23 (53)	17 (34)	16 (30)	32 (56)	36 (59)
Lithuania	0 (0)	0 (0)	0 (0)	0 (0)	0 (1)	0 (0)	0 (0)
Morocco	11 (3)	9 (2)	10 (2)	9 (2)	36 (8)	108 (20)	130 (25)
Madagascar	1 (1)	2 (1)	4 (1)	6 (2)	15 (3)	25 (4)	32 (5)
Mexico	354 (26)	619 (34)	774 (36)	925 (43)	1081 (48)	1081 (51)	1129 (53)
Mali	5 (3)	14 (7)	43 (16)	137 (42)	229 (53)	376 (63)	450 (65)
Myanmar	1 (0)	1 (0)	1 (0)	2 (0)	5 (0)	5 (1)	7 (1)
Mongolia	1 (2)	1 (1)	0 (0)	0 (0)	0 (0)	0 (0)	0 (1)
Mozambique	77 (32)	97 (33)	152 (39)	175 (40)	228 (39)	210 (29)	279 (37)
Mauritania	4 (12)	3 (6)	12 (22)	21 (32)	33 (40)	45 (43)	48 (43)
Malawi	68 (57)	102 (62)	165 (71)	212 (64)	261 (65)	277 (53)	412 (65)
Malaysia	31 (14)	38 (15)	45 (16)	85 (24)	89 (23)	78 (22)	92 (23)
Namibia	0 (2)	0 (2)	1 (2)	1 (1)	1 (1)	1 (1)	2 (3)
New Caledonia	0 (0)	0 (0)	0 (0)	0 (13)	0 (20)	0 (23)	0 (23)
Niger	7 (5)	5 (3)	5 (2)	20 (6)	35 (8)	136 (21)	206 (25)
Nigeria	91 (6)	94 (5)	258 (10)	576 (17)	1046 (26)	1593 (30)	2955 (47)
Nicaragua	24 (32)	34 (38)	89 (72)	108 (76)	109 (79)	105 (81)	99 (81)
Norway	0 (0)	0 (0)	0 (0)	0 (0)	0 (0)	0 (1)	0 (1)
Nepal	0 (0)	0 (0)	6 (1)	76 (10)	188 (22)	220 (28)	326 (41)
New Zealand	0 (0)	0 (0)	0 (0)	0 (0)	0 (0)	6 (10)	7 (13)
Oman	0 (0)	0 (0)	1 (2)	6 (12)	10 (22)	15 (30)	18 (34)
Pakistan	164 (11)	291 (15)	651 (25)	1168 (33)	1508 (39)	1769 (41)	2016 (43)
Panama	0 (0)	5 (13)	9 (23)	11 (26)	19 (42)	27 (58)	28 (58)
Peru	16 (6)	31 (8)	60 (14)	85 (19)	119 (26)	123 (28)	134 (32)
Philippines	62 (9)	79 (9)	116 (11)	183 (14)	224 (16)	231 (16)	293 (20)
Papua New Guinea	3 (5)	8 (12)	14 (16)	18 (18)	26 (20)	43 (27)	58 (36)
Puerto Rico	0 (0)	0 (0)	5 (8)	0 (0)	0 (0)	0 (0)	0 (0)

Dem. Rep. Korea	5 (2)	20 (5)	26 (10)	31 (10)	32 (10)	31 (12)	24 (10)
Portugal	4 (3)	4 (3)	6 (4)	3 (3)	4 (4)	4 (5)	3 (5)
Paraguay	0 (0)	0 (0)	9 (10)	38 (32)	46 (36)	69 (61)	85 (73)
Romania	2 (0)	3 (1)	3 (1)	3 (1)	2 (1)	2 (1)	2 (1)
Russian Federation	13 (1)	7 (0)	7 (0)	9 (0)	7 (1)	11 (1)	13 (1)
Rwanda	0 (0)	14 (9)	34 (15)	67 (22)	70 (24)	138 (36)	180 (41)
Saudi Arabia	2 (2)	11 (6)	35 (11)	53 (11)	52 (11)	79 (14)	98 (16)
Sudan	9 (3)	24 (6)	70 (12)	157 (22)	268 (31)	387 (36)	479 (39)
Senegal	37 (31)	43 (27)	95 (45)	121 (45)	129 (40)	177 (42)	180 (38)
Sierra Leone	0 (0)	0 (0)	1 (1)	2 (2)	2 (2)	22 (13)	27 (15)
El Salvador	22 (23)	48 (38)	97 (67)	130 (91)	141 (97)	114 (97)	110 (99)
Somalia	1 (1)	1 (1)	2 (1)	20 (10)	26 (11)	12 (4)	46 (15)
Serbia	0 (0)	0 (0)	0 (0)	0 (0)	0 (0)	1 (2)	0 (0)
South Sudan	4 (4)	11 (9)	20 (12)	80 (36)	119 (43)	142 (52)	191 (61)
Slovakia	0 (0)	0 (0)	0 (0)	0 (0)	0 (0)	0 (0)	9 (14)
Slovenia	0 (1)	0 (1)	0 (0)	0 (1)	0 (1)	4 (14)	4 (16)
Sweden	0 (0)	0 (0)	1 (1)	1 (1)	1 (1)	2 (2)	3 (4)
Swaziland	5 (49)	3 (22)	6 (29)	2 (7)	2 (7)	14 (47)	18 (61)
Chad	8 (8)	8 (6)	23 (13)	46 (19)	69 (21)	126 (29)	189 (38)
Togo	2 (4)	4 (5)	10 (10)	20 (16)	30 (20)	141 (72)	170 (87)
Thailand	5 (1)	32 (3)	53 (4)	78 (8)	118 (13)	250 (32)	273 (38)
Tajikistan	0 (1)	1 (1)	1 (1)	3 (2)	4 (2)	4 (2)	4 (2)
Turkmenistan	0 (0)	0 (0)	0 (0)	0 (0)	0 (0)	0 (0)	3 (2)
Timor-Leste	0 (0)	0 (0)	0 (0)	0 (0)	2 (6)	3 (9)	15 (38)
Tunisia	10 (8)	16 (11)	20 (11)	10 (5)	9 (6)	7 (5)	10 (6)
Turkey	4 (0)	1 (0)	1 (0)	0 (0)	5 (0)	8 (1)	37 (3)
Tanzania	6 (2)	10 (2)	15 (2)	30 (4)	38 (4)	84 (6)	134 (8)
Uganda	24 (9)	38 (10)	46 (9)	141 (20)	203 (20)	285 (22)	493 (31)
Uruguay	0 (0)	0 (0)	0 (0)	0 (0)	1 (3)	0 (0)	1 (2)
United States	45 (1)	36 (1)	60 (2)	167 (4)	218 (6)	365 (9)	380 (9)
Uzbekistan	1 (0)	2 (0)	4 (1)	9 (1)	12 (2)	30 (6)	72 (13)

Saint Vincent and the Grenadines	0 (0)	0 (0)	1 (33)	1 (33)	1 (33)	0 (0)	0 (0)
Venezuela	168 (69)	249 (77)	308 (78)	421 (86)	443 (88)	463 (89)	463 (90)
Vietnam	12 (1)	18 (1)	37 (3)	65 (4)	90 (7)	313 (24)	383 (30)
Vanuatu	0 (0)	0 (8)	0 (22)	0 (22)	0 (0)	0 (0)	0 (0)
Samoa	0 (0)	0 (0)	0 (0)	0 (33)	0 (33)	0 (0)	0 (0)
Yemen	46 (28)	71 (32)	141 (44)	268 (55)	360 (61)	486 (69)	608 (70)
South Africa	118 (21)	165 (24)	218 (25)	257 (26)	285 (30)	380 (38)	391 (40)
Zambia	30 (26)	43 (27)	55 (25)	78 (27)	108 (29)	166 (35)	237 (41)
Zimbabwe	37 (25)	39 (19)	48 (17)	42 (12)	40 (11)	71 (17)	97 (26)

Table 13: **Absolute population (in thousands) of cohorts living unprecedented exposure to wildfires and $CF_{wildfires}$ (%) per country and birth year in a 1.5°C pathway**

Country	1960	1970	1980	1990	2000	2010	2020
Afghanistan	19 (6)	50 (12)	76 (14)	86 (16)	168 (18)	217 (19)	259 (19)
Angola	10 (5)	21 (10)	35 (12)	49 (13)	67 (15)	103 (16)	115 (17)
Albania	2 (3)	2 (3)	4 (6)	7 (10)	5 (10)	5 (13)	5 (15)
United Arab Emirates	0 (0)	0 (15)	3 (26)	5 (30)	8 (44)	13 (44)	15 (46)
Argentina	18 (4)	20 (4)	30 (5)	36 (5)	43 (6)	53 (7)	54 (8)
Armenia	2 (2)	2 (4)	4 (6)	5 (7)	3 (7)	4 (9)	6 (14)
Australia	11 (5)	15 (6)	16 (7)	21 (9)	24 (10)	27 (10)	31 (10)
Austria	0 (0)	3 (3)	6 (7)	7 (8)	9 (11)	11 (14)	13 (16)
Azerbaijan	3 (3)	5 (4)	7 (6)	10 (7)	8 (8)	9 (9)	10 (10)
Burundi	6 (6)	10 (8)	13 (8)	14 (6)	18 (8)	28 (9)	37 (10)
Belgium	0 (0)	0 (0)	1 (1)	6 (4)	11 (8)	12 (8)	14 (9)
Benin	0 (0)	1 (1)	2 (2)	4 (3)	5 (3)	7 (3)	10 (3)
Burkina Faso	0 (0)	2 (1)	4 (1)	5 (1)	8 (2)	12 (2)	17 (2)
Bangladesh	13 (1)	22 (1)	42 (2)	68 (2)	117 (4)	146 (5)	140 (5)
Bulgaria	1 (1)	3 (2)	6 (4)	7 (7)	5 (8)	7 (10)	6 (9)
Bahamas	0 (0)	0 (12)	0 (13)	0 (13)	0 (13)	0 (15)	0 (15)
Bosnia and Herzegovina	2 (2)	4 (5)	7 (9)	7 (11)	6 (13)	5 (15)	4 (13)
Belarus	2 (1)	4 (2)	6 (4)	9 (6)	8 (9)	12 (12)	13 (12)
Belize	0 (0)	0 (0)	0 (0)	0 (0)	0 (1)	0 (1)	0 (1)
Bolivia	4 (3)	10 (7)	17 (10)	25 (12)	31 (14)	36 (15)	38 (16)
Brazil	44 (2)	77 (3)	104 (3)	128 (4)	140 (5)	133 (5)	142 (6)
Brunei Darussalam	0 (6)	0 (6)	0 (6)	0 (3)	0 (3)	0 (3)	0 (3)
Bhutan	0 (0)	0 (0)	0 (1)	1 (3)	2 (11)	3 (16)	3 (19)
Botswana	0 (0)	0 (1)	1 (3)	1 (2)	1 (2)	4 (9)	6 (13)
Central African Republic	1 (2)	5 (9)	9 (12)	6 (6)	5 (5)	11 (10)	17 (13)
Canada	8 (2)	13 (4)	21 (6)	31 (9)	45 (13)	50 (14)	54 (14)
Switzerland	0 (0)	2 (3)	2 (4)	3 (5)	4 (6)	4 (7)	7 (10)
Chile	10 (5)	20 (9)	22 (10)	34 (14)	34 (15)	34 (16)	33 (16)
China	119 (1)	607 (2)	625 (3)	878 (3)	643 (4)	726 (5)	675 (5)
Cote dIvoire	4 (3)	7 (4)	12 (4)	15 (3)	16 (3)	27 (4)	40 (6)
Cameroon	1 (1)	6 (2)	14 (4)	17 (4)	15 (3)	31 (5)	36 (5)

Democratic Republic of the Congo	10 (2)	22 (3)	42 (5)	74 (6)	124 (8)	227 (10)	301 (11)
Republic of Congo	1 (2)	1 (1)	2 (1)	2 (1)	9 (3)	19 (6)	21 (7)
Colombia	10 (2)	21 (3)	23 (3)	24 (3)	25 (3)	25 (3)	24 (3)
Comoros	0 (0)	0 (0)	0 (0)	0 (0)	0 (0)	0 (0)	0 (0)
Cape Verde	0 (0)	0 (0)	0 (3)	0 (3)	0 (3)	0 (0)	0 (0)
Costa Rica	2 (4)	2 (5)	3 (5)	2 (4)	3 (4)	2 (3)	1 (2)
Cuba	3 (2)	3 (2)	3 (2)	3 (2)	3 (3)	2 (2)	2 (2)
Cyprus	0 (6)	0 (6)	0 (6)	0 (7)	0 (5)	0 (5)	0 (5)
Czech Republic	1 (1)	3 (2)	5 (3)	6 (5)	6 (8)	12 (12)	14 (14)
Germany	3 (0)	14 (1)	18 (2)	42 (5)	55 (7)	61 (9)	71 (10)
Djibouti	0 (1)	0 (1)	0 (4)	0 (4)	0 (3)	0 (3)	0 (3)
Denmark	0 (0)	0 (0)	0 (1)	2 (3)	3 (5)	3 (7)	4 (9)
Dominican Republic	0 (0)	0 (0)	1 (1)	0 (0)	0 (0)	1 (0)	1 (1)
Algeria	3 (1)	42 (9)	60 (9)	88 (12)	76 (13)	101 (14)	112 (14)
Ecuador	1 (0)	2 (1)	5 (2)	9 (3)	16 (5)	19 (6)	20 (7)
Egypt	0 (0)	245 (23)	299 (23)	383 (24)	344 (24)	411 (24)	468 (24)
Eritrea	1 (2)	1 (2)	2 (2)	2 (2)	2 (2)	4 (3)	5 (3)
Spain	10 (2)	22 (4)	36 (7)	33 (9)	33 (11)	47 (12)	44 (14)
Estonia	0 (0)	0 (0)	0 (0)	0 (2)	0 (3)	1 (5)	1 (7)
Ethiopia	16 (2)	20 (2)	29 (2)	57 (3)	110 (5)	223 (8)	287 (10)
Finland	0 (0)	0 (0)	2 (3)	4 (8)	7 (13)	8 (15)	10 (17)
Fiji	0 (0)	0 (0)	0 (0)	0 (0)	0 (0)	0 (1)	0 (3)
France	9 (1)	14 (2)	19 (3)	32 (4)	37 (5)	55 (8)	65 (9)
Gabon	0 (3)	1 (6)	1 (7)	2 (7)	2 (7)	3 (8)	3 (10)
United Kingdom	15 (2)	42 (5)	50 (8)	77 (11)	84 (13)	102 (15)	120 (16)
Georgia	1 (1)	2 (3)	4 (4)	6 (7)	5 (9)	7 (12)	6 (12)
Ghana	1 (0)	1 (0)	1 (0)	1 (0)	1 (0)	2 (0)	4 (1)
Guinea	1 (1)	4 (3)	11 (8)	17 (9)	20 (8)	25 (9)	36 (11)
The Gambia	0 (0)	0 (0)	0 (0)	0 (0)	0 (0)	0 (0)	1 (2)
Guinea-Bissau	0 (0)	0 (1)	0 (2)	1 (2)	1 (4)	1 (4)	2 (6)

Equatorial Guinea	0 (2)	0 (3)	0 (7)	1 (9)	2 (12)	1 (8)	1 (8)
Greece	2 (2)	3 (3)	6 (5)	7 (9)	8 (10)	10 (12)	8 (12)
Guatemala	0 (0)	0 (0)	0 (0)	0 (0)	0 (0)	1 (0)	1 (0)
Guyana	0 (1)	0 (1)	0 (1)	0 (2)	0 (2)	0 (3)	0 (3)
Hong Kong	5 (6)	4 (6)	6 (9)	10 (15)	8 (15)	0 (0)	0 (0)
Honduras	0 (0)	0 (0)	1 (0)	1 (1)	1 (1)	2 (1)	2 (1)
Croatia	1 (2)	3 (4)	5 (8)	6 (10)	6 (11)	5 (13)	5 (14)
Haiti	0 (0)	0 (0)	0 (0)	1 (0)	4 (2)	4 (2)	4 (2)
Hungary	1 (0)	3 (2)	5 (3)	5 (4)	6 (6)	8 (8)	8 (8)
Indonesia	47 (2)	97 (3)	126 (3)	130 (3)	180 (5)	217 (6)	198 (6)
India	105 (1)	214 (1)	294 (1)	406 (2)	634 (3)	729 (3)	682 (3)
Iran	26 (3)	43 (5)	25 (2)	114 (6)	91 (7)	103 (8)	112 (9)
Iraq	2 (1)	62 (16)	78 (16)	98 (16)	130 (16)	161 (16)	186 (16)
Iceland	0 (0)	0 (0)	0 (0)	0 (0)	0 (0)	0 (1)	0 (1)
Israel	0 (0)	0 (0)	0 (1)	1 (2)	3 (3)	3 (3)	4 (3)
Italy	11 (2)	39 (5)	49 (8)	48 (11)	51 (12)	61 (13)	60 (15)
Jamaica	0 (0)	0 (0)	3 (6)	2 (6)	2 (6)	2 (6)	2 (6)
Jordan	1 (5)	5 (8)	7 (9)	8 (7)	10 (7)	11 (8)	11 (8)
Japan	4 (0)	6 (0)	21 (1)	18 (2)	26 (3)	28 (3)	32 (4)
Kazakhstan	30 (10)	40 (12)	53 (15)	58 (15)	32 (14)	52 (16)	61 (18)
Kenya	6 (2)	13 (3)	21 (3)	20 (2)	27 (3)	68 (5)	97 (7)
Kyrgyzstan	7 (10)	11 (13)	14 (14)	19 (15)	16 (15)	20 (15)	26 (18)
Cambodia	3 (1)	0 (0)	7 (4)	23 (6)	29 (9)	32 (9)	37 (11)
Republic of Korea	1 (0)	9 (1)	23 (3)	17 (3)	35 (7)	23 (7)	20 (6)
Kuwait	0 (0)	3 (24)	5 (24)	6 (24)	6 (24)	13 (46)	14 (46)
Lao PDR	9 (13)	12 (14)	15 (14)	19 (14)	22 (15)	21 (15)	21 (16)
Lebanon	0 (0)	0 (0)	0 (0)	0 (0)	1 (1)	0 (1)	0 (0)
Liberia	1 (2)	3 (8)	5 (9)	5 (9)	7 (10)	13 (14)	20 (17)
Libya	0 (1)	5 (7)	8 (8)	11 (10)	11 (11)	13 (12)	15 (13)
Sri Lanka	4 (2)	12 (5)	14 (5)	12 (5)	13 (6)	20 (7)	18 (7)
Lesotho	2 (6)	4 (13)	9 (20)	7 (15)	5 (9)	10 (18)	12 (20)
Lithuania	0 (0)	0 (0)	0 (0)	0 (1)	0 (1)	0 (1)	0 (1)
Latvia	0 (0)	0 (0)	0 (0)	1 (2)	1 (3)	1 (4)	1 (5)
Morocco	2 (1)	8 (2)	18 (4)	28 (5)	28 (6)	34 (7)	34 (7)
Moldova	0 (0)	0 (0)	1 (1)	2 (3)	2 (4)	2 (6)	2 (6)
Madagascar	15 (9)	34 (15)	55 (18)	73 (19)	103 (20)	124 (20)	142 (20)
Mexico	64 (5)	119 (7)	191 (9)	178 (8)	199 (9)	200 (9)	204 (10)

Macedonia	0 (1)	1 (4)	2 (8)	3 (10)	2 (10)	2 (12)	2 (11)
Mali	2 (1)	22 (10)	37 (14)	57 (17)	72 (17)	98 (16)	122 (17)
Myanmar	18 (3)	34 (4)	72 (7)	69 (7)	80 (8)	73 (8)	68 (9)
Montenegro	0 (0)	0 (1)	0 (4)	0 (5)	0 (6)	1 (9)	1 (12)
Mongolia	2 (7)	6 (12)	7 (13)	10 (14)	7 (14)	8 (14)	10 (16)
Mozambique	15 (6)	22 (7)	32 (8)	35 (8)	49 (8)	85 (12)	122 (16)
Mauritania	0 (1)	3 (7)	5 (8)	5 (8)	6 (8)	8 (8)	9 (8)
Malawi	5 (4)	9 (5)	20 (9)	16 (5)	21 (5)	64 (12)	87 (14)
Malaysia	1 (1)	2 (1)	3 (1)	1 (0)	2 (0)	1 (0)	2 (0)
Namibia	0 (0)	1 (4)	2 (6)	1 (2)	1 (2)	3 (6)	5 (9)
New Caledonia	0 (0)	0 (8)	0 (8)	0 (9)	0 (9)	0 (8)	0 (8)
Niger	0 (0)	1 (1)	5 (2)	8 (2)	14 (3)	22 (3)	32 (4)
Nigeria	91 (6)	70 (4)	84 (3)	79 (2)	88 (2)	122 (2)	195 (3)
Nicaragua	0 (0)	0 (0)	1 (1)	1 (1)	1 (1)	1 (1)	1 (1)
Netherlands	0 (0)	1 (0)	1 (1)	5 (4)	11 (7)	11 (8)	14 (9)
Norway	0 (0)	0 (0)	0 (0)	0 (1)	2 (4)	3 (6)	4 (7)
Nepal	11 (3)	31 (7)	52 (9)	83 (11)	115 (14)	109 (14)	107 (14)
New Zealand	3 (5)	3 (5)	2 (5)	4 (7)	4 (8)	6 (10)	7 (13)
Oman	0 (0)	4 (18)	10 (27)	16 (31)	14 (33)	16 (32)	17 (32)
Pakistan	4 (0)	112 (6)	161 (6)	242 (7)	264 (7)	293 (7)	316 (7)
Panama	1 (3)	1 (4)	1 (1)	0 (1)	0 (1)	0 (1)	1 (1)
Peru	5 (2)	14 (4)	22 (5)	26 (6)	28 (6)	29 (7)	29 (7)
Philippines	7 (1)	9 (1)	20 (2)	19 (1)	44 (3)	55 (4)	71 (5)
Papua New Guinea	0 (1)	2 (3)	5 (6)	6 (6)	8 (7)	11 (7)	11 (7)
Poland	3 (0)	5 (1)	7 (1)	16 (3)	23 (6)	27 (7)	30 (8)
Puerto Rico	0 (0)	4 (7)	8 (13)	8 (15)	7 (15)	6 (15)	5 (15)
Dem. Rep. Korea	1 (0)	21 (6)	26 (10)	30 (10)	32 (10)	30 (11)	27 (11)
Portugal	4 (3)	7 (5)	11 (9)	9 (11)	10 (12)	13 (17)	13 (19)
Paraguay	5 (8)	7 (9)	9 (10)	12 (11)	13 (10)	13 (11)	12 (10)
Qatar	0 (0)	1 (24)	1 (24)	2 (24)	4 (46)	5 (47)	6 (47)
Romania	3 (1)	11 (3)	22 (5)	28 (8)	20 (9)	23 (11)	19 (10)
Russian Federation	33 (1)	30 (2)	47 (2)	59 (3)	38 (3)	78 (5)	95 (6)
Rwanda	4 (3)	5 (3)	7 (3)	8 (2)	7 (2)	13 (3)	15 (3)
Saudi Arabia	1 (1)	44 (23)	77 (24)	115 (24)	110 (24)	139 (24)	146 (24)

Sudan	1 (0)	13 (3)	36 (6)	48 (7)	62 (7)	77 (7)	94 (8)
Senegal	0 (0)	0 (0)	1 (1)	4 (2)	7 (2)	17 (4)	23 (5)
Solomon Islands	0 (3)	0 (5)	0 (6)	0 (5)	0 (6)	0 (6)	0 (6)
Sierra Leone	1 (1)	1 (1)	1 (1)	1 (1)	2 (2)	3 (2)	5 (3)
Somalia	2 (2)	3 (4)	6 (3)	10 (5)	11 (4)	16 (5)	19 (6)
Serbia	0 (0)	1 (1)	3 (3)	6 (6)	7 (8)	7 (9)	6 (9)
South Sudan	2 (2)	6 (5)	11 (7)	22 (10)	33 (12)	35 (13)	44 (14)
Suriname	0 (1)	0 (1)	0 (3)	1 (5)	0 (5)	0 (5)	0 (5)
Slovakia	0 (0)	1 (1)	2 (2)	4 (5)	4 (7)	6 (9)	5 (9)
Slovenia	0 (1)	1 (3)	2 (7)	2 (12)	3 (17)	6 (23)	7 (28)
Sweden	0 (0)	1 (1)	3 (3)	7 (7)	8 (10)	9 (12)	12 (13)
Swaziland	1 (11)	3 (20)	5 (24)	6 (22)	4 (16)	6 (20)	7 (23)
Syria	6 (4)	13 (6)	22 (7)	28 (7)	32 (7)	35 (7)	34 (8)
Chad	1 (1)	3 (3)	7 (4)	9 (4)	13 (4)	20 (5)	27 (5)
Thailand	5 (1)	16 (1)	32 (3)	35 (3)	36 (4)	34 (4)	37 (5)
Tajikistan	2 (2)	4 (4)	8 (6)	19 (10)	21 (12)	25 (14)	29 (17)
Turkmenistan	1 (2)	4 (5)	6 (6)	9 (8)	9 (9)	13 (11)	16 (11)
Timor-Leste	0 (0)	0 (0)	0 (0)	0 (2)	1 (4)	2 (5)	3 (7)
Trinidad and Tobago	0 (0)	0 (1)	1 (3)	1 (3)	0 (3)	0 (3)	1 (4)
Tunisia	0 (0)	2 (1)	4 (2)	5 (3)	4 (3)	5 (3)	8 (5)
Turkey	23 (3)	65 (6)	122 (10)	151 (13)	178 (15)	188 (17)	188 (17)
Tanzania	20 (6)	39 (9)	59 (10)	69 (9)	90 (9)	178 (12)	251 (15)
Uganda	2 (1)	5 (1)	7 (1)	8 (1)	11 (1)	25 (2)	40 (2)
Ukraine	3 (0)	5 (1)	11 (1)	15 (2)	14 (3)	20 (4)	19 (4)
Uruguay	1 (2)	1 (2)	1 (2)	2 (4)	3 (6)	4 (8)	4 (9)
United States	158 (4)	220 (6)	301 (9)	368 (10)	422 (11)	478 (12)	487 (12)
Uzbekistan	18 (6)	26 (7)	41 (9)	64 (10)	64 (12)	69 (13)	77 (14)
Saint Vincent and the Grenadines	0 (0)	0 (0)	0 (0)	0 (0)	0 (6)	0 (0)	0 (0)
Venezuela	1 (0)	3 (1)	7 (2)	7 (2)	13 (3)	18 (4)	18 (4)
United States Virgin Islands	0 (0)	0 (0)	0 (3)	0 (6)	0 (6)	0 (0)	0 (0)

Vietnam	44 (4)	38 (3)	38 (3)	29 (2)	25 (2)	25 (2)	30 (2)
Vanuatu	0 (0)	0 (0)	0 (7)	0 (7)	0 (7)	0 (15)	0 (15)
Samoa	0 (0)	0 (0)	0 (0)	0 (15)	0 (15)	0 (0)	0 (0)
Yemen	0 (0)	14 (6)	25 (8)	45 (9)	59 (10)	71 (10)	84 (10)
South Africa	22 (4)	49 (7)	92 (10)	103 (11)	98 (10)	138 (14)	155 (16)
Zambia	16 (14)	29 (18)	43 (20)	46 (16)	54 (14)	92 (19)	117 (20)
Zimbabwe	14 (10)	31 (15)	49 (17)	44 (13)	42 (11)	67 (16)	68 (18)

Table 14: **Absolute population (in thousands) of cohorts living unprecedented exposure to wildfires and $CF_{wildfires}$ (%) per country and birth year in a 2.5°C pathway**

Country	1960	1970	1980	1990	2000	2010	2020
Afghanistan	16 (5)	52 (12)	87 (16)	97 (19)	188 (20)	255 (22)	303 (23)
Angola	10 (5)	22 (10)	36 (12)	51 (14)	69 (15)	107 (17)	118 (18)
Albania	1 (3)	2 (4)	5 (8)	8 (12)	7 (15)	8 (22)	9 (25)
United Arab Emirates	0 (0)	0 (14)	3 (26)	6 (30)	9 (45)	13 (45)	15 (47)
Argentina	23 (5)	24 (5)	36 (5)	42 (6)	59 (9)	70 (10)	75 (11)
Armenia	1 (2)	4 (6)	5 (8)	6 (8)	6 (15)	9 (22)	9 (22)
Australia	12 (6)	16 (7)	19 (9)	24 (10)	27 (11)	33 (13)	40 (14)
Austria	0 (0)	1 (1)	3 (4)	6 (7)	9 (10)	13 (16)	17 (20)
Azerbaijan	4 (3)	7 (6)	10 (9)	17 (12)	14 (14)	16 (16)	18 (17)
Burundi	6 (6)	9 (7)	13 (9)	19 (8)	20 (9)	35 (11)	42 (11)
Belgium	0 (0)	0 (0)	0 (0)	5 (3)	9 (7)	13 (9)	14 (9)
Benin	0 (0)	1 (1)	2 (2)	3 (2)	4 (2)	5 (2)	7 (2)
Burkina Faso	0 (0)	1 (1)	3 (1)	5 (1)	8 (2)	13 (2)	16 (2)
Bangladesh	15 (1)	27 (1)	44 (2)	82 (3)	106 (4)	109 (4)	108 (4)
Bulgaria	1 (1)	4 (3)	8 (6)	8 (8)	6 (9)	10 (14)	10 (15)
Bahamas	0 (0)	0 (13)	0 (14)	0 (14)	0 (14)	0 (16)	0 (16)
Bosnia and Herzegovina	3 (3)	3 (4)	6 (8)	10 (15)	8 (17)	7 (20)	7 (22)
Belarus	1 (0)	5 (3)	9 (6)	12 (7)	8 (9)	11 (10)	13 (13)
Belize	0 (0)	0 (0)	0 (0)	0 (0)	0 (0)	0 (0)	0 (0)
Bolivia	4 (3)	11 (8)	21 (12)	29 (14)	39 (17)	46 (20)	51 (21)
Brazil	57 (3)	94 (4)	124 (4)	154 (5)	171 (6)	162 (6)	181 (7)
Brunei Darussalam	0 (8)	0 (8)	0 (8)	0 (8)	0 (8)	1 (8)	1 (8)
Bhutan	0 (0)	0 (0)	0 (1)	1 (7)	3 (13)	4 (21)	5 (26)
Botswana	0 (0)	0 (2)	1 (3)	1 (2)	1 (2)	3 (7)	8 (17)
Central African Republic	1 (2)	5 (9)	9 (13)	5 (5)	4 (4)	12 (10)	21 (17)
Canada	8 (2)	13 (4)	30 (9)	44 (12)	48 (14)	62 (18)	73 (19)
Switzerland	1 (2)	2 (3)	2 (4)	3 (5)	4 (6)	6 (10)	8 (12)
Chile	12 (6)	24 (10)	27 (12)	39 (16)	45 (19)	47 (21)	48 (23)
China	126 (1)	577 (2)	585 (3)	924 (4)	728 (5)	955 (6)	960 (7)
Cote dIvoire	4 (3)	7 (4)	12 (4)	14 (3)	14 (2)	21 (3)	26 (4)
Cameroon	1 (1)	5 (2)	12 (4)	10 (2)	12 (2)	22 (3)	35 (5)

Democratic Republic of the Congo	9 (2)	21 (3)	39 (4)	70 (6)	116 (7)	213 (9)	297 (11)
Republic of Congo	1 (1)	1 (1)	1 (1)	1 (1)	11 (4)	25 (8)	37 (12)
Colombia	22 (4)	28 (4)	31 (4)	28 (3)	20 (3)	19 (3)	29 (4)
Cape Verde	0 (0)	0 (4)	0 (4)	0 (4)	0 (4)	0 (0)	0 (0)
Costa Rica	2 (5)	3 (6)	3 (6)	4 (6)	3 (4)	1 (1)	1 (1)
Cuba	3 (3)	4 (2)	2 (2)	2 (2)	2 (1)	2 (2)	2 (3)
Cyprus	0 (8)	0 (8)	0 (8)	0 (8)	0 (8)	1 (14)	1 (18)
Czech Republic	1 (1)	2 (2)	5 (3)	5 (4)	5 (7)	15 (14)	17 (16)
Germany	4 (0)	14 (1)	16 (2)	36 (4)	46 (6)	57 (8)	64 (9)
Djibouti	0 (0)	0 (2)	0 (3)	0 (3)	0 (3)	0 (3)	0 (2)
Denmark	0 (0)	0 (0)	0 (1)	1 (2)	2 (4)	3 (7)	3 (8)
Dominican Republic	0 (0)	0 (0)	2 (1)	1 (0)	1 (1)	1 (1)	3 (1)
Algeria	3 (1)	47 (10)	65 (10)	97 (13)	82 (14)	113 (15)	125 (16)
Ecuador	1 (1)	2 (1)	5 (2)	7 (3)	14 (5)	21 (7)	21 (7)
Egypt	0 (0)	250 (24)	304 (24)	391 (24)	351 (24)	419 (24)	477 (24)
Eritrea	1 (2)	2 (3)	1 (2)	2 (2)	3 (2)	5 (3)	7 (4)
Spain	18 (3)	34 (6)	54 (10)	46 (13)	48 (16)	70 (18)	64 (20)
Estonia	0 (0)	0 (1)	0 (1)	0 (1)	0 (1)	1 (6)	1 (9)
Ethiopia	17 (2)	24 (2)	33 (2)	72 (4)	160 (7)	306 (12)	405 (14)
Finland	0 (0)	1 (1)	3 (5)	5 (9)	6 (12)	9 (16)	10 (17)
Fiji	0 (0)	0 (0)	0 (0)	0 (0)	0 (0)	0 (2)	0 (3)
France	11 (1)	17 (2)	24 (4)	47 (7)	56 (8)	79 (11)	86 (12)
Gabon	0 (3)	1 (5)	1 (6)	1 (5)	1 (4)	2 (5)	1 (5)
United Kingdom	5 (1)	34 (4)	48 (8)	66 (9)	78 (12)	106 (15)	133 (18)
Georgia	1 (2)	5 (5)	6 (7)	11 (12)	9 (15)	10 (18)	10 (19)
Ghana	1 (0)	1 (0)	1 (0)	1 (0)	0 (0)	1 (0)	3 (0)
Guinea	1 (1)	3 (3)	10 (7)	13 (7)	19 (7)	23 (8)	35 (11)
Guinea-Bissau	0 (0)	0 (1)	0 (1)	0 (1)	0 (1)	0 (1)	1 (2)
Equatorial Guinea	0 (1)	0 (4)	1 (9)	1 (7)	1 (7)	0 (3)	0 (3)
Greece	3 (2)	4 (4)	8 (7)	9 (11)	11 (15)	13 (16)	11 (16)
Guyana	0 (1)	0 (1)	0 (1)	0 (1)	0 (0)	0 (1)	0 (1)

Hong Kong	7 (8)	6 (8)	9 (12)	11 (16)	13 (24)	0 (0)	0 (0)
Honduras	0 (0)	0 (0)	1 (1)	1 (1)	1 (1)	1 (1)	1 (1)
Croatia	3 (5)	3 (5)	4 (7)	6 (11)	6 (13)	6 (16)	6 (16)
Haiti	0 (0)	0 (0)	2 (1)	3 (2)	4 (2)	4 (2)	4 (2)
Hungary	1 (1)	2 (2)	4 (2)	4 (3)	5 (5)	6 (6)	7 (8)
Indonesia	58 (2)	116 (4)	150 (4)	127 (3)	156 (4)	197 (5)	191 (5)
India	118 (1)	207 (1)	316 (2)	433 (2)	546 (2)	635 (3)	647 (3)
Ireland	0 (0)	0 (0)	0 (0)	0 (0)	0 (0)	0 (0)	0 (1)
Iran	30 (4)	49 (5)	31 (2)	139 (8)	113 (9)	136 (11)	159 (13)
Iraq	3 (1)	64 (16)	83 (17)	106 (17)	142 (18)	180 (18)	209 (18)
Iceland	0 (0)	0 (0)	0 (0)	0 (1)	0 (1)	0 (1)	0 (1)
Israel	0 (0)	0 (1)	1 (1)	1 (1)	2 (2)	2 (2)	2 (2)
Italy	15 (2)	36 (5)	53 (9)	58 (13)	67 (16)	82 (18)	76 (19)
Jamaica	0 (0)	0 (0)	3 (7)	3 (6)	3 (6)	2 (6)	3 (7)
Jordan	2 (6)	5 (9)	7 (9)	10 (9)	13 (10)	15 (10)	15 (10)
Japan	6 (0)	9 (1)	18 (1)	13 (1)	11 (1)	11 (1)	22 (3)
Kazakhstan	32 (10)	44 (14)	59 (17)	68 (18)	41 (18)	68 (21)	74 (22)
Kenya	6 (2)	15 (3)	25 (4)	29 (3)	44 (4)	94 (7)	143 (11)
Kyrgyzstan	8 (10)	12 (14)	15 (15)	21 (16)	19 (17)	26 (19)	28 (20)
Cambodia	3 (1)	0 (0)	8 (4)	25 (7)	27 (8)	31 (9)	33 (10)
Republic of Korea	0 (0)	2 (0)	24 (3)	39 (7)	57 (11)	50 (15)	52 (17)
Kuwait	0 (0)	3 (24)	5 (24)	6 (24)	6 (24)	13 (47)	14 (47)
Lao PDR	9 (13)	12 (14)	16 (15)	20 (15)	23 (16)	23 (17)	23 (17)
Lebanon	0 (0)	0 (0)	0 (1)	1 (1)	1 (2)	1 (3)	2 (3)
Liberia	0 (1)	3 (8)	5 (9)	6 (10)	7 (10)	12 (13)	17 (15)
Libya	1 (1)	5 (8)	9 (9)	11 (10)	12 (12)	15 (14)	18 (15)
Sri Lanka	2 (1)	17 (6)	23 (8)	16 (6)	14 (6)	20 (7)	20 (8)
Lesotho	2 (7)	4 (12)	9 (22)	9 (19)	7 (13)	14 (23)	14 (24)
Lithuania	0 (0)	0 (0)	0 (0)	0 (1)	1 (2)	1 (2)	1 (2)
Latvia	0 (0)	0 (1)	0 (2)	1 (3)	1 (5)	1 (6)	2 (8)
Morocco	3 (1)	13 (3)	27 (6)	39 (8)	40 (9)	54 (10)	59 (11)
Moldova	0 (0)	0 (0)	0 (0)	0 (0)	1 (1)	1 (3)	3 (8)
Madagascar	15 (9)	36 (16)	58 (19)	78 (21)	108 (21)	133 (22)	154 (22)
Mexico	76 (6)	145 (8)	202 (9)	231 (11)	252 (11)	251 (12)	267 (13)
Macedonia	0 (1)	1 (3)	3 (9)	4 (14)	3 (14)	3 (14)	3 (15)
Mali	2 (1)	23 (11)	38 (14)	60 (18)	76 (18)	105 (18)	129 (19)
Myanmar	19 (3)	36 (4)	70 (7)	81 (8)	84 (9)	91 (10)	90 (11)
Montenegro	0 (0)	0 (1)	0 (3)	0 (3)	0 (7)	1 (14)	1 (15)

Mongolia	2 (6)	6 (13)	9 (16)	14 (20)	10 (21)	13 (22)	15 (23)
Mozambique	15 (6)	24 (8)	34 (9)	39 (9)	57 (10)	89 (12)	126 (17)
Mauritania	0 (1)	3 (7)	5 (8)	5 (8)	7 (8)	8 (8)	9 (8)
Malawi	5 (4)	9 (5)	19 (8)	14 (4)	22 (5)	74 (14)	116 (18)
Malaysia	2 (1)	3 (1)	5 (2)	5 (1)	3 (1)	4 (1)	4 (1)
Namibia	0 (1)	1 (4)	2 (6)	1 (2)	1 (3)	4 (7)	6 (10)
New Caledonia	0 (0)	0 (8)	0 (10)	0 (9)	0 (9)	0 (8)	0 (8)
Niger	0 (0)	1 (1)	5 (2)	9 (3)	15 (3)	23 (4)	33 (4)
Nigeria	107 (7)	77 (4)	81 (3)	79 (2)	91 (2)	123 (2)	180 (3)
Netherlands	0 (0)	3 (2)	4 (3)	9 (6)	12 (7)	12 (8)	13 (9)
Norway	0 (0)	0 (0)	0 (0)	0 (1)	1 (3)	2 (5)	5 (10)
Nepal	13 (3)	33 (7)	61 (10)	92 (12)	121 (14)	126 (16)	135 (17)
New Zealand	3 (6)	3 (5)	3 (7)	4 (8)	4 (8)	5 (8)	6 (10)
Oman	0 (0)	4 (19)	10 (27)	16 (31)	15 (33)	16 (33)	18 (34)
Pakistan	5 (0)	115 (6)	169 (7)	270 (8)	314 (8)	359 (8)	398 (9)
Panama	1 (5)	2 (5)	1 (3)	0 (1)	0 (0)	0 (0)	0 (1)
Peru	5 (2)	15 (4)	24 (6)	31 (7)	34 (8)	35 (8)	37 (9)
Philippines	9 (1)	13 (2)	29 (3)	12 (1)	19 (1)	16 (1)	21 (1)
Papua New Guinea	0 (1)	3 (5)	6 (7)	6 (6)	9 (7)	10 (6)	10 (6)
Poland	4 (1)	6 (1)	12 (2)	17 (3)	21 (5)	28 (7)	33 (9)
Puerto Rico	0 (0)	3 (6)	8 (14)	8 (16)	8 (16)	7 (16)	6 (16)
Dem. Rep. Korea	2 (1)	23 (6)	23 (9)	31 (10)	40 (13)	36 (14)	36 (15)
Portugal	5 (4)	9 (7)	15 (12)	12 (14)	14 (17)	15 (20)	16 (23)
Paraguay	6 (10)	9 (12)	10 (11)	16 (14)	18 (14)	17 (15)	18 (15)
Qatar	0 (4)	1 (28)	2 (28)	2 (28)	4 (48)	5 (48)	7 (48)
Romania	2 (1)	8 (2)	21 (5)	28 (8)	22 (10)	28 (13)	27 (14)
Russian Federation	33 (1)	37 (2)	55 (3)	70 (3)	47 (4)	93 (6)	125 (8)
Rwanda	5 (4)	6 (4)	10 (4)	10 (3)	11 (4)	19 (5)	23 (5)
Saudi Arabia	1 (1)	45 (23)	78 (24)	118 (24)	113 (25)	143 (24)	151 (24)
Sudan	1 (0)	13 (3)	36 (6)	49 (7)	61 (7)	76 (7)	90 (7)
Senegal	0 (0)	1 (0)	1 (1)	5 (2)	7 (2)	10 (2)	12 (3)
Solomon Islands	0 (2)	0 (3)	0 (2)	0 (3)	0 (4)	0 (5)	0 (3)

Sierra Leone	1 (1)	1 (1)	1 (1)	1 (1)	2 (1)	4 (2)	5 (3)
Somalia	2 (3)	4 (5)	7 (4)	10 (5)	13 (6)	21 (7)	25 (8)
Serbia	0 (0)	1 (1)	4 (4)	5 (5)	6 (7)	6 (8)	7 (10)
South Sudan	2 (2)	6 (5)	13 (8)	26 (12)	36 (13)	39 (14)	48 (15)
Suriname	0 (1)	0 (2)	0 (1)	0 (1)	0 (0)	0 (0)	0 (0)
Slovakia	0 (0)	1 (1)	3 (2)	3 (3)	3 (6)	5 (8)	6 (10)
Slovenia	0 (2)	1 (3)	1 (5)	3 (14)	3 (20)	7 (29)	8 (32)
Sweden	0 (0)	2 (2)	3 (4)	5 (5)	7 (9)	9 (11)	12 (13)
Swaziland	2 (15)	3 (24)	5 (24)	7 (24)	6 (22)	7 (24)	7 (24)
Syria	7 (4)	16 (7)	26 (8)	35 (9)	39 (9)	45 (9)	42 (10)
Chad	1 (1)	3 (3)	7 (4)	10 (4)	12 (4)	20 (5)	28 (5)
Thailand	6 (1)	17 (1)	30 (3)	25 (2)	27 (3)	28 (3)	30 (4)
Tajikistan	2 (3)	5 (5)	12 (9)	24 (13)	27 (15)	31 (17)	32 (18)
Turkmenistan	1 (3)	4 (5)	7 (8)	11 (9)	9 (9)	14 (12)	18 (13)
Timor-Leste	0 (0)	0 (0)	0 (0)	0 (1)	1 (2)	1 (3)	1 (3)
Trinidad and Tobago	0 (0)	0 (0)	0 (2)	0 (0)	0 (0)	0 (0)	0 (0)
Tunisia	0 (0)	3 (2)	5 (2)	6 (3)	6 (4)	8 (5)	10 (6)
Turkey	24 (3)	66 (6)	140 (12)	180 (15)	204 (17)	225 (20)	228 (21)
Tanzania	20 (6)	40 (9)	63 (10)	75 (9)	110 (11)	202 (14)	299 (17)
Uganda	3 (1)	6 (2)	7 (1)	8 (1)	13 (1)	36 (3)	53 (3)
Ukraine	2 (0)	6 (1)	10 (1)	14 (2)	13 (3)	20 (4)	23 (5)
Uruguay	1 (2)	1 (3)	2 (3)	4 (8)	5 (10)	6 (12)	7 (15)
United States	173 (4)	226 (7)	318 (10)	418 (11)	502 (13)	566 (14)	590 (15)
Uzbekistan	20 (7)	30 (8)	48 (10)	74 (12)	76 (14)	86 (16)	92 (17)
Venezuela	1 (0)	5 (2)	5 (1)	4 (1)	5 (1)	2 (0)	6 (1)
United States Virgin Islands	0 (0)	0 (4)	0 (4)	0 (8)	0 (8)	0 (0)	0 (0)
Vietnam	57 (5)	61 (5)	52 (4)	36 (2)	29 (2)	30 (2)	32 (2)
Vanuatu	0 (0)	0 (0)	0 (5)	0 (7)	0 (10)	0 (16)	0 (16)
Samoa	0 (0)	0 (0)	0 (0)	0 (16)	0 (16)	0 (0)	0 (0)
Yemen	0 (0)	14 (6)	31 (10)	52 (11)	65 (11)	83 (12)	111 (13)
South Africa	26 (5)	55 (8)	102 (12)	120 (12)	121 (13)	174 (18)	201 (21)
Zambia	17 (15)	29 (18)	46 (21)	51 (18)	67 (18)	105 (22)	134 (23)
Zimbabwe	16 (11)	31 (15)	51 (18)	50 (14)	51 (14)	83 (20)	88 (23)

Table 15: **Absolute population (in thousands) of cohorts living unprecedented exposure to wildfires and $CF_{wildfires}$ (%) per country and birth year in a 3.5°C pathway**

Country	1960	1970	1980	1990	2000	2010	2020
Afghanistan	15 (4)	52 (12)	88 (16)	101 (19)	202 (22)	275 (24)	351 (26)
Angola	14 (8)	26 (12)	43 (15)	62 (17)	85 (19)	126 (20)	139 (21)
Albania	2 (4)	3 (6)	8 (13)	11 (16)	11 (22)	10 (27)	10 (29)
United Arab Emirates	0 (0)	0 (17)	3 (29)	6 (34)	10 (49)	14 (50)	17 (52)
Argentina	18 (4)	21 (4)	32 (5)	39 (6)	52 (7)	64 (9)	71 (10)
Armenia	0 (0)	3 (6)	4 (7)	9 (12)	8 (20)	10 (24)	11 (25)
Australia	13 (6)	16 (7)	22 (10)	25 (11)	30 (13)	36 (14)	44 (15)
Austria	1 (1)	1 (1)	4 (5)	10 (11)	16 (19)	21 (26)	22 (27)
Azerbaijan	2 (2)	6 (5)	10 (9)	16 (11)	14 (14)	15 (15)	20 (18)
Burundi	9 (9)	14 (11)	17 (11)	25 (11)	27 (12)	43 (14)	54 (15)
Belgium	0 (0)	0 (0)	0 (0)	12 (9)	18 (14)	21 (14)	24 (16)
Benin	0 (0)	1 (2)	2 (2)	3 (2)	4 (2)	5 (2)	9 (3)
Burkina Faso	0 (0)	1 (1)	3 (1)	6 (2)	9 (2)	14 (2)	16 (2)
Bangladesh	5 (0)	5 (0)	14 (1)	20 (1)	56 (2)	79 (3)	70 (3)
Bulgaria	1 (1)	3 (2)	7 (5)	7 (6)	6 (10)	9 (13)	9 (13)
Bahamas	0 (0)	0 (11)	0 (12)	0 (12)	0 (12)	0 (13)	0 (13)
Bosnia and Herzegovina	2 (2)	4 (4)	8 (11)	11 (15)	10 (21)	8 (24)	9 (26)
Belarus	3 (1)	3 (2)	5 (4)	12 (7)	8 (9)	11 (10)	17 (16)
Belize	0 (0)	0 (0)	0 (0)	0 (0)	0 (0)	0 (0)	0 (0)
Bolivia	4 (4)	11 (8)	20 (11)	33 (17)	43 (19)	52 (22)	55 (23)
Brazil	55 (2)	87 (3)	115 (4)	139 (4)	156 (5)	154 (6)	195 (8)
Brunei Darussalam	0 (7)	0 (7)	0 (7)	0 (7)	0 (7)	0 (7)	1 (7)
Bhutan	0 (0)	0 (0)	0 (0)	1 (5)	3 (16)	5 (23)	4 (23)
Botswana	0 (0)	0 (1)	1 (3)	1 (1)	1 (3)	5 (12)	8 (18)
Central African Republic	1 (3)	4 (8)	10 (14)	5 (6)	5 (5)	12 (10)	23 (18)
Canada	9 (2)	15 (4)	36 (11)	53 (15)	61 (18)	73 (21)	88 (23)
Switzerland	0 (1)	4 (5)	5 (8)	8 (13)	11 (17)	12 (19)	18 (25)
Chile	11 (6)	24 (10)	28 (13)	42 (17)	47 (20)	50 (23)	52 (25)
China	153 (1)	637 (2)	628 (3)	983 (4)	815 (5)	1119 (7)	1290 (9)
Cote dIvoire	4 (3)	9 (4)	14 (4)	15 (3)	15 (3)	24 (4)	26 (4)
Cameroon	2 (1)	6 (3)	17 (5)	15 (3)	20 (4)	38 (6)	53 (8)

Democratic Republic of the Congo	10 (2)	20 (3)	37 (4)	63 (5)	108 (7)	205 (9)	285 (11)
Republic of Congo	0 (1)	1 (1)	1 (1)	1 (1)	18 (6)	23 (8)	41 (14)
Colombia	14 (2)	25 (4)	26 (4)	25 (3)	23 (3)	23 (3)	23 (3)
Cape Verde	0 (0)	0 (0)	0 (0)	0 (7)	0 (7)	0 (0)	0 (0)
Costa Rica	2 (6)	3 (7)	4 (7)	5 (7)	2 (3)	2 (3)	2 (3)
Cuba	3 (3)	4 (2)	2 (2)	2 (1)	1 (1)	1 (1)	1 (1)
Cyprus	0 (7)	0 (7)	0 (7)	0 (12)	0 (12)	0 (12)	1 (12)
Czech Republic	1 (1)	1 (1)	3 (2)	3 (3)	9 (11)	18 (17)	20 (20)
Germany	6 (1)	13 (1)	17 (2)	57 (6)	78 (10)	74 (10)	82 (11)
Djibouti	0 (0)	0 (2)	0 (2)	0 (2)	0 (2)	0 (3)	0 (3)
Denmark	0 (0)	0 (0)	0 (0)	1 (2)	3 (6)	4 (10)	4 (11)
Dominican Republic	0 (0)	0 (0)	0 (0)	0 (0)	2 (1)	2 (1)	2 (1)
Algeria	3 (1)	49 (10)	64 (10)	100 (13)	86 (15)	117 (16)	136 (17)
Ecuador	1 (1)	2 (1)	5 (2)	5 (2)	9 (3)	10 (3)	15 (5)
Egypt	0 (0)	278 (26)	338 (27)	434 (27)	390 (27)	466 (27)	530 (27)
Eritrea	1 (2)	1 (2)	2 (2)	2 (2)	2 (2)	6 (4)	6 (3)
Spain	14 (3)	29 (5)	60 (11)	52 (15)	54 (18)	78 (20)	73 (23)
Estonia	0 (0)	0 (1)	0 (2)	0 (2)	0 (2)	1 (5)	2 (14)
Ethiopia	16 (2)	21 (2)	32 (2)	73 (4)	181 (7)	352 (13)	481 (17)
Finland	0 (0)	1 (1)	3 (5)	5 (9)	9 (17)	10 (18)	12 (21)
Fiji	0 (0)	0 (0)	0 (0)	0 (0)	0 (0)	0 (1)	0 (2)
France	16 (2)	27 (4)	49 (7)	81 (12)	95 (14)	118 (16)	128 (18)
Gabon	0 (5)	1 (6)	1 (8)	1 (5)	1 (4)	2 (5)	3 (10)
United Kingdom	4 (1)	45 (5)	60 (10)	104 (15)	129 (20)	167 (24)	188 (25)
Georgia	1 (1)	4 (4)	7 (8)	14 (15)	9 (17)	11 (19)	12 (22)
Ghana	1 (0)	1 (0)	1 (0)	0 (0)	1 (0)	1 (0)	1 (0)
Guinea	2 (2)	5 (4)	11 (8)	15 (8)	21 (8)	30 (10)	40 (13)
Guinea-Bissau	0 (0)	0 (1)	0 (1)	0 (1)	1 (3)	1 (3)	1 (3)
Equatorial Guinea	0 (0)	0 (4)	1 (9)	1 (7)	1 (6)	0 (3)	1 (5)
Greece	3 (2)	5 (4)	10 (9)	10 (12)	12 (16)	13 (17)	12 (18)
Guyana	0 (1)	0 (1)	0 (1)	0 (1)	0 (1)	0 (2)	0 (1)

Hong Kong	6 (7)	5 (7)	5 (7)	9 (13)	14 (27)	0 (0)	0 (0)
Honduras	0 (0)	0 (0)	1 (1)	1 (0)	1 (0)	1 (0)	1 (0)
Croatia	3 (4)	3 (4)	4 (6)	5 (9)	6 (13)	6 (15)	6 (17)
Haiti	0 (0)	0 (0)	0 (0)	0 (0)	0 (0)	0 (0)	1 (0)
Hungary	0 (0)	1 (1)	3 (1)	4 (3)	5 (4)	8 (8)	10 (11)
Indonesia	67 (3)	133 (4)	165 (4)	110 (3)	138 (4)	180 (5)	184 (5)
India	93 (1)	170 (1)	268 (1)	337 (1)	408 (2)	444 (2)	475 (2)
Ireland	0 (0)	0 (0)	0 (0)	0 (0)	0 (0)	0 (1)	1 (1)
Iran	29 (4)	47 (5)	22 (2)	142 (8)	120 (10)	162 (13)	181 (14)
Iraq	2 (1)	71 (18)	90 (18)	115 (19)	152 (19)	187 (19)	221 (19)
Iceland	0 (0)	0 (0)	0 (1)	0 (0)	0 (1)	0 (1)	0 (1)
Israel	0 (0)	0 (1)	1 (1)	1 (1)	1 (2)	2 (2)	6 (5)
Italy	13 (2)	31 (4)	52 (9)	59 (13)	70 (17)	87 (19)	89 (22)
Jamaica	0 (0)	0 (0)	3 (6)	2 (5)	2 (5)	2 (6)	2 (6)
Jordan	2 (5)	5 (8)	6 (8)	10 (9)	12 (9)	14 (9)	14 (9)
Japan	7 (1)	9 (1)	14 (1)	8 (1)	14 (1)	11 (1)	18 (2)
Kazakhstan	34 (11)	47 (14)	66 (19)	74 (19)	46 (20)	77 (23)	84 (25)
Kenya	6 (2)	15 (3)	27 (4)	29 (3)	54 (5)	120 (9)	166 (12)
Kyrgyzstan	7 (10)	11 (13)	17 (17)	23 (17)	22 (20)	27 (21)	31 (22)
Cambodia	1 (1)	0 (0)	7 (4)	24 (6)	23 (7)	27 (8)	31 (9)
Republic of Korea	0 (0)	10 (1)	16 (2)	53 (10)	87 (17)	69 (21)	73 (23)
Kuwait	0 (0)	4 (27)	6 (27)	7 (27)	6 (27)	15 (53)	16 (53)
Lao PDR	10 (15)	14 (17)	17 (16)	22 (16)	27 (18)	25 (18)	26 (20)
Lebanon	0 (0)	1 (1)	1 (1)	1 (2)	1 (2)	2 (5)	4 (7)
Liberia	1 (2)	4 (9)	7 (12)	7 (12)	9 (13)	15 (16)	23 (20)
Libya	0 (1)	6 (8)	11 (11)	12 (11)	13 (13)	15 (13)	16 (13)
Sri Lanka	2 (1)	18 (7)	19 (7)	16 (6)	15 (6)	30 (10)	22 (9)
Lesotho	2 (7)	5 (16)	11 (27)	12 (24)	10 (19)	15 (26)	16 (27)
Lithuania	0 (0)	0 (0)	0 (0)	1 (1)	0 (1)	0 (1)	1 (2)
Latvia	0 (0)	0 (0)	0 (2)	1 (4)	1 (4)	1 (6)	2 (11)
Morocco	1 (0)	10 (2)	20 (4)	32 (6)	35 (8)	50 (9)	55 (11)
Moldova	0 (0)	0 (0)	2 (2)	1 (2)	3 (6)	4 (10)	4 (12)
Madagascar	17 (10)	39 (17)	66 (22)	90 (24)	125 (24)	150 (24)	173 (25)
Mexico	66 (5)	128 (7)	175 (8)	215 (10)	253 (11)	259 (12)	285 (13)
Macedonia	0 (1)	1 (3)	2 (8)	3 (12)	3 (12)	3 (15)	3 (15)
Mali	2 (1)	25 (12)	42 (16)	66 (20)	82 (19)	109 (18)	130 (19)
Myanmar	21 (3)	39 (5)	69 (7)	84 (9)	86 (9)	90 (10)	88 (11)
Montenegro	0 (0)	0 (1)	0 (4)	0 (6)	1 (9)	1 (20)	2 (27)

Mongolia	2 (6)	6 (13)	11 (20)	15 (21)	11 (23)	15 (26)	18 (27)
Mozambique	15 (6)	23 (8)	39 (10)	43 (10)	60 (10)	95 (13)	136 (18)
Mauritania	0 (1)	3 (7)	5 (9)	5 (8)	6 (8)	8 (8)	9 (8)
Malawi	5 (4)	9 (6)	21 (9)	14 (4)	20 (5)	76 (14)	121 (19)
Malaysia	2 (1)	3 (1)	4 (2)	5 (1)	3 (1)	5 (1)	6 (1)
Namibia	0 (1)	1 (4)	2 (5)	0 (1)	1 (2)	4 (7)	6 (10)
New Caledonia	0 (0)	0 (7)	0 (9)	0 (9)	0 (9)	0 (7)	0 (7)
Niger	0 (0)	1 (1)	6 (3)	10 (3)	16 (4)	26 (4)	37 (4)
Nigeria	107 (7)	82 (4)	89 (3)	81 (2)	82 (2)	148 (3)	177 (3)
Nicaragua	0 (0)	0 (0)	1 (1)	1 (1)	1 (1)	1 (1)	1 (1)
Netherlands	0 (0)	2 (1)	5 (4)	15 (10)	19 (12)	20 (13)	22 (15)
Norway	0 (0)	0 (0)	0 (0)	2 (3)	3 (5)	5 (10)	7 (14)
Nepal	11 (3)	27 (6)	50 (8)	88 (12)	117 (14)	118 (15)	122 (15)
New Zealand	4 (7)	4 (8)	4 (9)	5 (11)	5 (10)	7 (12)	8 (14)
Oman	0 (0)	4 (20)	11 (31)	18 (35)	16 (37)	18 (36)	19 (36)
Pakistan	3 (0)	124 (6)	180 (7)	269 (8)	307 (8)	352 (8)	469 (10)
Panama	1 (4)	2 (5)	2 (5)	1 (3)	1 (3)	0 (1)	0 (1)
Peru	4 (1)	16 (4)	26 (6)	34 (7)	36 (8)	36 (8)	35 (9)
Philippines	7 (1)	8 (1)	11 (1)	15 (1)	15 (1)	27 (2)	29 (2)
Papua New Guinea	1 (1)	4 (5)	6 (7)	7 (7)	9 (7)	12 (7)	9 (6)
Poland	5 (1)	4 (1)	6 (1)	11 (2)	20 (5)	30 (8)	39 (10)
Puerto Rico	0 (0)	3 (5)	7 (12)	7 (13)	7 (13)	5 (13)	5 (13)
Dem. Rep. Korea	2 (1)	37 (10)	34 (13)	42 (14)	49 (16)	51 (19)	53 (22)
Portugal	5 (4)	10 (7)	16 (12)	15 (17)	18 (22)	18 (24)	19 (27)
Paraguay	5 (8)	8 (10)	10 (11)	14 (12)	15 (12)	15 (13)	15 (13)
Qatar	0 (7)	1 (34)	2 (34)	3 (34)	5 (53)	6 (54)	7 (54)
Romania	3 (1)	9 (2)	22 (5)	25 (7)	27 (12)	32 (14)	31 (16)
Russian Federation	25 (1)	25 (1)	42 (2)	70 (3)	51 (4)	103 (7)	159 (10)
Rwanda	4 (3)	5 (3)	9 (4)	10 (3)	7 (2)	17 (4)	22 (5)
Saudi Arabia	1 (0)	49 (26)	86 (27)	130 (27)	125 (27)	158 (27)	167 (27)
Sudan	1 (0)	16 (4)	40 (7)	54 (8)	69 (8)	82 (8)	97 (8)
Senegal	0 (0)	1 (0)	1 (1)	1 (0)	8 (2)	11 (3)	22 (5)
Solomon Islands	0 (3)	0 (4)	0 (5)	0 (2)	0 (5)	0 (8)	0 (8)

Sierra Leone	1 (1)	1 (2)	2 (2)	2 (2)	3 (2)	4 (3)	5 (3)
Somalia	2 (2)	4 (4)	6 (4)	10 (5)	14 (6)	18 (6)	28 (9)
Serbia	0 (0)	1 (1)	3 (3)	5 (5)	5 (6)	7 (9)	8 (11)
South Sudan	2 (3)	7 (6)	14 (8)	26 (12)	39 (14)	40 (15)	44 (14)
Suriname	0 (1)	0 (1)	0 (1)	0 (1)	0 (1)	0 (1)	0 (0)
Slovakia	0 (0)	0 (1)	2 (2)	3 (3)	4 (7)	6 (11)	8 (13)
Slovenia	1 (2)	1 (2)	2 (6)	4 (19)	4 (27)	10 (39)	11 (46)
Sweden	0 (0)	1 (1)	2 (3)	9 (9)	10 (13)	13 (16)	17 (19)
Swaziland	1 (11)	4 (27)	5 (27)	7 (27)	7 (25)	8 (27)	8 (27)
Syria	7 (4)	14 (6)	22 (7)	30 (8)	34 (8)	39 (8)	38 (9)
Chad	1 (1)	4 (3)	7 (4)	11 (5)	15 (5)	21 (5)	26 (5)
Thailand	7 (1)	14 (1)	27 (2)	23 (2)	25 (3)	27 (3)	36 (5)
Tajikistan	2 (3)	5 (5)	11 (8)	23 (13)	28 (16)	31 (18)	41 (23)
Turkmenistan	2 (3)	6 (8)	9 (10)	13 (11)	12 (12)	17 (14)	22 (15)
Timor-Leste	0 (0)	0 (0)	0 (0)	1 (4)	1 (4)	2 (5)	1 (3)
Tunisia	1 (0)	3 (2)	3 (2)	6 (3)	6 (4)	8 (5)	10 (6)
Turkey	26 (3)	67 (7)	150 (13)	200 (17)	223 (19)	248 (22)	277 (26)
Tanzania	24 (7)	46 (10)	72 (12)	84 (11)	114 (11)	229 (16)	313 (18)
Uganda	3 (1)	6 (2)	8 (1)	8 (1)	12 (1)	29 (2)	45 (3)
Ukraine	3 (0)	4 (1)	14 (2)	18 (3)	15 (4)	24 (5)	27 (6)
Uruguay	1 (2)	1 (2)	2 (3)	4 (8)	5 (9)	6 (13)	4 (10)
United States	199 (5)	255 (7)	346 (11)	475 (13)	568 (15)	666 (17)	715 (18)
Uzbekistan	21 (7)	31 (8)	53 (11)	84 (13)	90 (16)	103 (19)	116 (21)
Venezuela	2 (1)	7 (2)	6 (2)	7 (1)	2 (0)	8 (2)	2 (0)
United States Virgin Islands	0 (0)	0 (0)	0 (7)	0 (13)	0 (13)	0 (0)	0 (0)
Vietnam	52 (5)	51 (4)	51 (4)	32 (2)	30 (2)	32 (2)	43 (3)
Vanuatu	0 (0)	0 (0)	0 (5)	0 (9)	0 (12)	0 (13)	0 (13)
Samoa	0 (0)	0 (0)	0 (0)	0 (13)	0 (13)	0 (0)	0 (0)
Yemen	0 (0)	15 (7)	31 (10)	55 (11)	67 (11)	80 (11)	98 (11)
South Africa	25 (4)	56 (8)	107 (12)	127 (13)	141 (15)	210 (21)	231 (24)
Zambia	18 (15)	32 (20)	51 (23)	58 (20)	74 (20)	117 (25)	149 (26)
Zimbabwe	16 (11)	33 (16)	57 (20)	56 (16)	57 (15)	99 (24)	100 (26)

Table 16: **Absolute population (in thousands) of cohorts living unprecedented exposure to tropical cyclones and $CF_{\text{tropical cyclones}}$ (%) per country and birth year in a 1.5°C pathway**

Country	1960	1970	1980	1990	2000	2010	2020
Afghanistan	0 (0)	0 (0)	0 (0)	0 (0)	0 (0)	0 (0)	0 (0)
Angola	2 (1)	2 (1)	5 (2)	7 (2)	8 (2)	12 (2)	13 (2)
United Arab Emirates	0 (3)	0 (3)	0 (3)	1 (3)	1 (3)	1 (4)	0 (0)
Australia	25 (12)	19 (8)	18 (8)	25 (11)	26 (11)	31 (12)	33 (11)
Benin	1 (2)	2 (2)	2 (2)	3 (2)	4 (2)	0 (0)	0 (0)
Bangladesh	26 (1)	126 (6)	189 (7)	475 (16)	577 (19)	499 (18)	474 (18)
Bahamas	0 (0)	0 (0)	0 (2)	0 (10)	0 (10)	0 (12)	0 (12)
Belize	0 (0)	0 (0)	0 (0)	0 (11)	0 (10)	0 (10)	1 (10)
Brazil	17 (1)	26 (1)	32 (1)	44 (1)	54 (2)	45 (2)	47 (2)
Bhutan	0 (0)	0 (0)	0 (0)	0 (0)	2 (9)	2 (9)	2 (9)
Botswana	2 (8)	2 (8)	3 (8)	3 (7)	0 (0)	0 (0)	0 (0)
Canada	8 (2)	4 (1)	4 (1)	4 (1)	4 (1)	3 (1)	1 (0)
China	581 (3)	1306 (5)	1186 (6)	1643 (6)	843 (5)	945 (6)	870 (6)
Cote dIvoire	3 (2)	11 (5)	20 (6)	25 (6)	26 (5)	24 (4)	27 (4)
Colombia	12 (2)	15 (2)	16 (2)	21 (3)	20 (3)	19 (3)	13 (2)
Cape Verde	0 (0)	0 (0)	0 (12)	1 (25)	1 (25)	0 (0)	0 (0)
Costa Rica	1 (1)	1 (2)	1 (2)	1 (2)	1 (2)	1 (2)	1 (2)
Cuba	1 (1)	7 (4)	9 (8)	14 (11)	13 (12)	11 (13)	10 (13)
Dominican Republic	0 (0)	0 (0)	0 (0)	7 (4)	11 (6)	16 (9)	16 (9)
Ethiopia	2 (0)	2 (0)	2 (0)	3 (0)	6 (0)	6 (0)	5 (0)
Fiji	0 (6)	0 (6)	1 (10)	1 (23)	2 (44)	4 (41)	3 (40)
France	1 (0)	1 (0)	1 (0)	1 (0)	2 (0)	1 (0)	0 (0)
Gabon	1 (6)	1 (6)	1 (4)	1 (4)	1 (4)	1 (2)	0 (0)
United Kingdom	2 (0)	2 (0)	1 (0)	1 (0)	0 (0)	0 (0)	0 (0)
Ghana	25 (11)	33 (11)	40 (11)	60 (13)	47 (8)	63 (9)	73 (10)
Guinea	2 (3)	3 (3)	7 (5)	7 (4)	14 (5)	15 (5)	21 (7)
The Gambia	0 (0)	0 (0)	2 (9)	3 (10)	10 (27)	14 (29)	14 (29)
Guinea-Bissau	0 (0)	0 (0)	0 (1)	1 (3)	5 (19)	6 (22)	7 (22)
Guatemala	11 (7)	26 (13)	54 (21)	60 (20)	79 (22)	90 (24)	93 (23)
Hong Kong	23 (25)	19 (25)	27 (38)	17 (25)	27 (50)	0 (0)	0 (0)

Honduras	3 (5)	8 (9)	18 (15)	34 (22)	42 (24)	40 (23)	35 (21)
Haiti	0 (0)	0 (0)	0 (0)	0 (0)	22 (10)	22 (10)	21 (10)
Indonesia	39 (1)	68 (2)	303 (8)	453 (12)	478 (13)	632 (17)	605 (17)
India	779 (6)	1332 (8)	1711 (9)	2170 (9)	2198 (9)	2308 (9)	1986 (9)
Ireland	4 (3)	4 (3)	3 (2)	2 (2)	0 (0)	0 (0)	0 (0)
Japan	10 (1)	5 (0)	28 (2)	69 (6)	72 (7)	82 (9)	78 (10)
Kenya	2 (0)	2 (0)	0 (0)	0 (0)	1 (0)	6 (1)	7 (1)
Cambodia	0 (0)	0 (0)	21 (11)	65 (17)	74 (23)	88 (26)	90 (27)
Republic of Korea	0 (0)	0 (0)	25 (4)	44 (8)	47 (9)	36 (11)	46 (15)
Lao PDR	3 (4)	5 (5)	8 (8)	18 (13)	21 (15)	26 (19)	32 (24)
Liberia	2 (6)	3 (6)	3 (6)	4 (6)	2 (3)	2 (2)	3 (2)
Sri Lanka	0 (0)	0 (0)	3 (1)	13 (5)	16 (7)	22 (8)	29 (11)
Morocco	2 (0)	2 (1)	3 (1)	3 (1)	3 (1)	3 (1)	0 (0)
Madagascar	3 (1)	3 (1)	4 (1)	13 (3)	44 (9)	93 (15)	117 (17)
Mexico	237 (17)	337 (18)	467 (22)	510 (24)	522 (23)	474 (22)	406 (19)
Mali	0 (0)	0 (0)	0 (0)	1 (0)	5 (1)	7 (1)	7 (1)
Myanmar	23 (3)	31 (4)	60 (6)	80 (8)	133 (14)	175 (19)	171 (22)
Mozambique	17 (7)	21 (7)	32 (8)	27 (6)	27 (5)	33 (5)	56 (7)
Mauritania	0 (1)	1 (3)	2 (3)	2 (3)	2 (3)	3 (3)	3 (2)
Malawi	4 (3)	12 (7)	16 (7)	24 (7)	30 (7)	31 (6)	38 (6)
Malaysia	15 (7)	18 (7)	29 (10)	33 (9)	42 (11)	45 (13)	65 (16)
New Caledonia	0 (4)	0 (16)	0 (19)	0 (15)	0 (16)	0 (7)	0 (12)
Nigeria	27 (2)	14 (1)	19 (1)	24 (1)	30 (1)	0 (0)	0 (0)
Nicaragua	7 (9)	12 (13)	12 (10)	29 (20)	32 (23)	26 (20)	25 (20)
Nepal	20 (5)	24 (5)	31 (5)	17 (2)	18 (2)	11 (1)	10 (1)
New Zealand	7 (13)	10 (18)	10 (22)	9 (18)	9 (18)	10 (18)	10 (17)
Oman	0 (3)	1 (3)	1 (3)	2 (3)	1 (3)	1 (1)	1 (1)
Pakistan	46 (3)	61 (3)	80 (3)	113 (3)	122 (3)	134 (3)	31 (1)
Panama	3 (10)	5 (14)	6 (14)	6 (15)	6 (14)	2 (5)	2 (3)
Peru	0 (0)	0 (0)	0 (0)	0 (0)	0 (0)	2 (1)	2 (1)
Philippines	4 (1)	33 (4)	131 (13)	264 (21)	366 (25)	397 (28)	523 (35)
Papua New Guinea	0 (1)	2 (3)	6 (7)	17 (16)	24 (19)	32 (20)	37 (23)
Puerto Rico	0 (0)	0 (0)	0 (0)	12 (22)	12 (25)	10 (25)	7 (20)

Dem. Rep. Korea	2 (1)	2 (1)	6 (2)	7 (2)	7 (2)	7 (3)	17 (7)
Russian Federation	1 (0)	1 (0)	2 (0)	3 (0)	1 (0)	1 (0)	2 (0)
Senegal	3 (2)	5 (3)	20 (10)	28 (11)	23 (7)	44 (11)	28 (6)
Solomon Islands	0 (2)	0 (3)	1 (13)	1 (28)	2 (37)	3 (46)	3 (59)
Sierra Leone	12 (18)	17 (21)	20 (20)	12 (10)	2 (2)	1 (0)	1 (0)
El Salvador	0 (0)	19 (15)	25 (17)	52 (36)	67 (46)	53 (45)	49 (44)
Somalia	1 (1)	1 (1)	1 (1)	3 (1)	8 (3)	12 (4)	16 (5)
Suriname	0 (2)	0 (2)	0 (0)	0 (0)	0 (0)	0 (0)	0 (0)
Swaziland	0 (5)	1 (5)	1 (4)	1 (4)	1 (4)	1 (4)	0 (0)
Thailand	17 (2)	53 (4)	84 (7)	86 (8)	94 (10)	107 (13)	107 (15)
Timor-Leste	0 (0)	0 (0)	0 (0)	3 (11)	9 (26)	9 (26)	11 (29)
Trinidad and Tobago	0 (0)	0 (0)	3 (12)	6 (32)	3 (25)	5 (32)	4 (31)
Tanzania	0 (0)	0 (0)	0 (0)	0 (0)	1 (0)	9 (1)	12 (1)
United States	186 (5)	181 (5)	211 (7)	216 (6)	251 (7)	272 (7)	271 (7)
Saint Vincent and the Grenadines	1 (25)	0 (12)	1 (25)	1 (25)	1 (25)	0 (0)	0 (0)
Venezuela	3 (1)	3 (1)	4 (1)	8 (2)	8 (2)	8 (2)	8 (2)
United States Virgin Islands	0 (0)	0 (12)	0 (12)	0 (12)	0 (25)	0 (0)	0 (0)
Vietnam	64 (6)	80 (6)	130 (9)	188 (11)	200 (15)	225 (17)	186 (15)
Vanuatu	0 (0)	0 (8)	0 (25)	0 (30)	0 (18)	0 (38)	0 (25)
Samoa	0 (0)	0 (0)	0 (25)	0 (38)	0 (38)	0 (0)	0 (0)
Yemen	10 (6)	13 (6)	18 (6)	28 (6)	33 (6)	14 (2)	2 (0)
South Africa	13 (2)	17 (2)	22 (2)	24 (2)	11 (1)	11 (1)	8 (1)
Zambia	0 (0)	0 (0)	0 (0)	0 (0)	0 (0)	1 (0)	1 (0)
Zimbabwe	3 (2)	4 (2)	12 (4)	15 (4)	14 (4)	14 (3)	13 (3)

Table 17: **Absolute population (in thousands) of cohorts living unprecedented exposure to tropical cyclones and $CF_{tropical\ cyclones}$ (%) per country and birth year in a 2.5°C pathway**

Country	1960	1970	1980	1990	2000	2010	2020
Afghanistan	0 (0)	0 (0)	0 (0)	0 (0)	0 (0)	0 (0)	0 (0)
Angola	1 (1)	2 (1)	7 (2)	11 (3)	12 (3)	27 (4)	27 (4)
United Arab Emirates	0 (3)	0 (4)	0 (4)	1 (4)	1 (5)	1 (5)	0 (0)
Australia	18 (9)	19 (8)	26 (12)	33 (14)	34 (14)	49 (19)	54 (18)
Benin	2 (2)	2 (2)	9 (7)	11 (7)	15 (7)	18 (7)	21 (7)
Bangladesh	16 (1)	103 (5)	194 (8)	430 (15)	640 (21)	725 (26)	668 (26)
Bahamas	0 (3)	0 (3)	0 (3)	0 (3)	0 (8)	0 (13)	0 (21)
Belize	0 (0)	0 (0)	0 (0)	0 (0)	0 (2)	1 (13)	1 (16)
Brazil	22 (1)	36 (1)	33 (1)	55 (2)	52 (2)	53 (2)	88 (3)
Brunei Darussalam	0 (0)	0 (0)	0 (0)	1 (16)	1 (16)	1 (16)	1 (16)
Bhutan	0 (0)	0 (0)	0 (1)	0 (1)	0 (1)	0 (1)	0 (1)
Botswana	1 (7)	2 (8)	3 (7)	3 (7)	0 (1)	0 (1)	0 (1)
Canada	5 (1)	3 (1)	3 (1)	4 (1)	3 (1)	2 (1)	2 (0)
China	657 (4)	1259 (5)	1269 (6)	2309 (9)	1318 (8)	1431 (9)	1354 (10)
Cote dIvoire	0 (0)	6 (3)	12 (4)	4 (1)	31 (5)	45 (7)	47 (7)
Democratic Republic of the Congo	0 (0)	0 (0)	1 (0)	2 (0)	2 (0)	13 (1)	15 (1)
Republic of Congo	0 (0)	0 (0)	0 (0)	0 (0)	1 (0)	1 (0)	1 (0)
Colombia	15 (3)	23 (3)	25 (3)	27 (3)	26 (3)	25 (3)	20 (3)
Cape Verde	0 (0)	0 (0)	0 (0)	0 (17)	0 (17)	0 (0)	0 (0)
Costa Rica	0 (1)	0 (1)	0 (1)	1 (2)	1 (2)	1 (1)	6 (9)
Cuba	4 (3)	9 (5)	7 (6)	12 (9)	21 (19)	22 (26)	20 (26)
Djibouti	0 (0)	0 (0)	0 (1)	0 (1)	0 (1)	0 (1)	0 (0)
Dominican Republic	0 (0)	0 (0)	1 (1)	6 (3)	6 (3)	29 (16)	39 (23)
Eritrea	0 (0)	0 (0)	0 (0)	0 (0)	0 (0)	0 (0)	1 (0)
Ethiopia	1 (0)	2 (0)	2 (0)	4 (0)	5 (0)	7 (0)	7 (0)
Fiji	0 (0)	0 (0)	0 (0)	0 (0)	0 (0)	1 (15)	2 (21)

France	1 (0)	1 (0)	1 (0)	1 (0)	1 (0)	1 (0)	0 (0)
Gabon	0 (4)	1 (4)	1 (3)	1 (3)	1 (4)	1 (2)	1 (2)
United Kingdom	2 (0)	2 (0)	1 (0)	1 (0)	0 (0)	0 (0)	0 (0)
Ghana	19 (9)	36 (12)	51 (14)	59 (13)	40 (7)	50 (7)	84 (11)
Guinea	2 (2)	4 (3)	11 (8)	12 (7)	22 (8)	31 (11)	38 (12)
The Gambia	0 (0)	0 (0)	2 (9)	0 (0)	4 (11)	5 (11)	6 (11)
Guinea-Bissau	0 (0)	0 (1)	1 (8)	2 (8)	3 (10)	3 (11)	5 (17)
Guatemala	8 (5)	16 (8)	31 (12)	73 (25)	93 (26)	96 (26)	128 (32)
Guyana	0 (0)	0 (0)	0 (0)	2 (13)	2 (13)	2 (13)	3 (23)
Hong Kong	0 (0)	0 (0)	12 (17)	12 (17)	18 (33)	0 (0)	0 (0)
Honduras	3 (4)	6 (6)	14 (12)	27 (17)	31 (18)	30 (17)	32 (19)
Haiti	0 (0)	0 (0)	0 (0)	1 (1)	11 (5)	41 (17)	63 (29)
Indonesia	37 (1)	109 (3)	317 (8)	413 (11)	475 (13)	631 (16)	504 (14)
India	812 (6)	1213 (7)	1998 (10)	2889 (12)	3038 (12)	3227 (13)	3130 (14)
Ireland	4 (3)	4 (2)	3 (2)	2 (2)	0 (0)	0 (0)	0 (0)
Jamaica	0 (0)	1 (2)	0 (0)	0 (0)	1 (2)	1 (2)	3 (8)
Japan	17 (1)	3 (0)	12 (1)	43 (4)	55 (6)	83 (9)	129 (17)
Kenya	2 (1)	2 (0)	0 (0)	0 (0)	0 (0)	0 (0)	3 (0)
Cambodia	0 (0)	0 (0)	18 (10)	63 (17)	104 (32)	127 (37)	123 (36)
Republic of Korea	0 (0)	0 (0)	1 (0)	13 (2)	24 (5)	10 (3)	15 (5)
Lao PDR	4 (5)	6 (7)	8 (7)	18 (13)	21 (15)	28 (20)	33 (25)
Liberia	1 (5)	2 (6)	3 (5)	3 (5)	4 (6)	11 (12)	12 (11)
Libya	0 (0)	2 (2)	3 (3)	3 (3)	3 (3)	3 (3)	3 (3)
Sri Lanka	0 (0)	3 (1)	3 (1)	13 (5)	34 (14)	42 (15)	52 (20)
Madagascar	3 (2)	4 (2)	3 (1)	6 (2)	23 (5)	37 (6)	57 (8)
Mexico	189 (14)	289 (16)	459 (21)	503 (23)	611 (27)	599 (28)	586 (28)
Mali	0 (0)	0 (0)	4 (1)	4 (1)	6 (1)	9 (1)	11 (2)
Myanmar	24 (4)	31 (4)	56 (6)	78 (8)	139 (15)	134 (15)	132 (17)
Mozambique	15 (6)	20 (7)	29 (7)	28 (7)	28 (5)	38 (5)	61 (8)
Mauritania	0 (0)	1 (3)	2 (3)	2 (3)	3 (3)	4 (4)	4 (4)
Malawi	5 (4)	15 (9)	22 (9)	33 (10)	49 (12)	48 (9)	94 (15)
Malaysia	13 (6)	23 (9)	25 (9)	36 (10)	45 (12)	46 (13)	51 (13)
New Caledonia	0 (4)	0 (7)	0 (15)	0 (24)	0 (26)	0 (33)	0 (30)
Nigeria	29 (2)	18 (1)	48 (2)	75 (2)	95 (2)	82 (2)	96 (2)
Nicaragua	3 (4)	5 (6)	11 (9)	15 (11)	20 (15)	23 (18)	27 (22)

Nepal	19 (5)	23 (5)	41 (7)	22 (3)	26 (3)	24 (3)	24 (3)
New Zealand	7 (14)	8 (15)	8 (16)	10 (19)	8 (14)	8 (14)	9 (16)
Oman	0 (3)	1 (6)	2 (6)	3 (6)	3 (6)	3 (5)	3 (5)
Pakistan	9 (1)	13 (1)	20 (1)	29 (1)	31 (1)	33 (1)	11 (0)
Panama	2 (8)	9 (24)	11 (28)	14 (34)	18 (39)	20 (43)	20 (42)
Philippines	10 (1)	8 (1)	55 (5)	198 (16)	427 (30)	598 (42)	646 (43)
Papua New Guinea	0 (1)	1 (2)	4 (5)	10 (9)	13 (10)	29 (19)	28 (17)
Puerto Rico	0 (0)	0 (0)	0 (0)	0 (0)	6 (13)	14 (33)	12 (33)
Dem. Rep. Korea	2 (1)	3 (1)	2 (1)	8 (3)	13 (4)	30 (11)	19 (8)
Russian Federation	1 (0)	1 (0)	1 (0)	2 (0)	2 (0)	3 (0)	2 (0)
Senegal	0 (0)	2 (1)	6 (3)	13 (5)	20 (6)	24 (6)	67 (14)
Solomon Islands	0 (0)	0 (8)	0 (8)	0 (6)	1 (18)	2 (31)	3 (47)
Sierra Leone	11 (16)	14 (18)	17 (18)	8 (7)	1 (1)	24 (14)	27 (15)
El Salvador	0 (0)	7 (6)	0 (0)	10 (7)	23 (16)	22 (18)	12 (11)
Somalia	1 (1)	0 (1)	7 (4)	9 (5)	12 (5)	31 (11)	44 (14)
Suriname	0 (2)	0 (2)	0 (0)	0 (0)	0 (0)	1 (14)	1 (14)
Swaziland	0 (3)	0 (3)	2 (12)	3 (12)	3 (11)	3 (11)	2 (8)
Togo	0 (0)	0 (0)	7 (7)	9 (7)	11 (7)	31 (16)	31 (16)
Thailand	19 (2)	34 (3)	38 (3)	46 (5)	87 (10)	113 (14)	107 (15)
Timor-Leste	0 (0)	0 (0)	0 (0)	0 (0)	11 (33)	15 (44)	15 (37)
Trinidad and Tobago	0 (0)	0 (0)	2 (9)	0 (0)	0 (0)	1 (9)	1 (9)
Tanzania	0 (0)	0 (0)	3 (0)	5 (1)	7 (1)	11 (1)	15 (1)
United States	165 (4)	198 (6)	226 (7)	289 (8)	353 (9)	442 (11)	505 (13)
Saint Vincent and the Grenadines	0 (17)	1 (17)	1 (33)	0 (17)	0 (17)	0 (0)	0 (0)
Venezuela	15 (6)	21 (6)	27 (7)	33 (7)	35 (7)	37 (7)	10 (2)
Vietnam	60 (5)	50 (4)	146 (10)	224 (14)	237 (18)	279 (22)	260 (20)

Vanuatu	0 (0)	0 (0)	0 (13)	0 (17)	0 (17)	0 (17)	0 (17)
Samoa	0 (0)	0 (0)	0 (0)	0 (17)	0 (33)	0 (0)	0 (0)
Yemen	13 (8)	17 (8)	26 (8)	61 (12)	97 (16)	89 (13)	107 (12)
South Africa	11 (2)	14 (2)	34 (4)	38 (4)	25 (3)	25 (3)	23 (2)
Zimbabwe	2 (2)	10 (5)	13 (4)	16 (5)	15 (4)	46 (11)	45 (12)

Table 18: **Absolute population (in thousands) of cohorts living unprecedented exposure to tropical cyclones and $CF_{\text{tropical cyclones}}$ (%) per country and birth year in a 3.5°C pathway**

Country	1960	1970	1980	1990	2000	2010	2020
Afghanistan	0 (0)	0 (0)	0 (0)	0 (0)	0 (0)	0 (0)	0 (0)
Angola	1 (1)	2 (1)	6 (2)	9 (2)	11 (2)	17 (3)	19 (3)
United Arab Emirates	0 (0)	0 (0)	0 (0)	0 (0)	1 (7)	2 (7)	2 (7)
Australia	14 (7)	19 (8)	27 (13)	35 (15)	37 (16)	41 (16)	46 (16)
Benin	2 (4)	3 (4)	5 (4)	8 (5)	10 (5)	3 (1)	4 (1)
Bangladesh	0 (0)	0 (0)	22 (1)	233 (8)	433 (14)	446 (16)	500 (19)
Bahamas	0 (0)	0 (0)	0 (5)	0 (5)	0 (0)	0 (0)	0 (0)
Brazil	16 (1)	19 (1)	11 (0)	18 (1)	45 (1)	43 (2)	54 (2)
Brunei Darussalam	0 (0)	0 (0)	0 (0)	1 (24)	1 (24)	2 (24)	2 (24)
Botswana	1 (5)	1 (5)	2 (5)	2 (5)	0 (0)	0 (0)	0 (0)
Canada	7 (2)	4 (1)	5 (1)	5 (1)	4 (1)	4 (1)	5 (1)
China	831 (5)	1739 (7)	1653 (8)	2612 (10)	1527 (10)	1867 (12)	1665 (12)
Cote dIvoire	0 (0)	1 (1)	2 (1)	10 (2)	13 (2)	15 (2)	19 (3)
Democratic Republic of the Congo	0 (0)	0 (0)	2 (0)	2 (0)	7 (0)	9 (0)	58 (2)
Republic of Congo	0 (0)	0 (0)	0 (0)	0 (0)	2 (1)	3 (1)	3 (1)
Colombia	22 (4)	26 (4)	28 (4)	33 (4)	32 (4)	32 (4)	30 (4)
Costa Rica	1 (1)	1 (1)	2 (3)	2 (3)	2 (3)	1 (1)	1 (1)
Cuba	0 (0)	8 (5)	7 (6)	19 (15)	18 (17)	16 (18)	16 (21)
Djibouti	0 (0)	0 (0)	0 (1)	0 (1)	0 (1)	0 (1)	0 (0)
Dominican Republic	0 (0)	0 (0)	1 (1)	18 (11)	20 (12)	28 (16)	41 (24)
Eritrea	0 (0)	0 (0)	0 (1)	1 (0)	0 (0)	1 (0)	1 (0)
Ethiopia	3 (0)	3 (0)	4 (0)	5 (0)	7 (0)	15 (1)	13 (0)
Fiji	0 (0)	0 (0)	0 (0)	0 (0)	0 (0)	0 (2)	0 (2)
France	2 (0)	2 (0)	1 (0)	1 (0)	2 (0)	1 (0)	0 (0)
Gabon	1 (6)	1 (6)	1 (5)	1 (5)	1 (5)	1 (3)	1 (3)
United Kingdom	2 (0)	2 (0)	2 (0)	7 (1)	5 (1)	7 (1)	9 (1)

Ghana	14 (6)	19 (6)	23 (6)	31 (7)	9 (2)	74 (11)	94 (12)
Guinea	1 (1)	5 (4)	7 (5)	10 (5)	13 (5)	23 (8)	26 (8)
The Gambia	0 (0)	0 (0)	0 (0)	0 (0)	0 (0)	8 (17)	10 (20)
Guinea-Bissau	0 (0)	0 (0)	0 (2)	1 (6)	2 (7)	5 (16)	8 (25)
Guatemala	1 (1)	5 (3)	40 (16)	43 (15)	61 (17)	87 (23)	158 (40)
Guyana	0 (0)	0 (0)	0 (0)	0 (1)	2 (15)	2 (15)	2 (15)
Hong Kong	0 (0)	19 (25)	18 (25)	17 (25)	14 (25)	0 (0)	0 (0)
Honduras	1 (1)	2 (2)	6 (5)	8 (5)	27 (15)	64 (36)	57 (35)
Haiti	0 (0)	0 (0)	0 (0)	13 (6)	41 (18)	42 (18)	40 (18)
Indonesia	49 (2)	223 (7)	532 (14)	455 (12)	562 (16)	673 (18)	646 (18)
India	453 (3)	761 (5)	1673 (8)	2345 (10)	3308 (13)	3814 (16)	4229 (19)
Ireland	6 (4)	5 (3)	4 (3)	3 (4)	2 (3)	1 (2)	1 (2)
Jamaica	0 (0)	0 (0)	0 (0)	0 (0)	0 (0)	1 (3)	1 (3)
Japan	6 (0)	3 (0)	3 (0)	34 (3)	47 (5)	123 (14)	162 (21)
Kenya	2 (1)	2 (1)	0 (0)	0 (0)	1 (0)	2 (0)	2 (0)
Cambodia	0 (0)	0 (0)	31 (17)	107 (28)	125 (38)	168 (49)	221 (65)
Republic of Korea	0 (0)	0 (0)	19 (3)	20 (4)	37 (7)	23 (7)	43 (14)
Lao PDR	3 (5)	6 (7)	8 (8)	13 (9)	20 (14)	28 (20)	30 (23)
Liberia	1 (3)	1 (3)	2 (3)	2 (4)	4 (5)	2 (2)	8 (7)
Sri Lanka	0 (0)	2 (1)	0 (0)	1 (0)	4 (2)	50 (17)	102 (40)
Morocco	0 (0)	0 (0)	0 (0)	0 (0)	8 (2)	10 (2)	10 (2)
Madagascar	3 (2)	2 (1)	1 (0)	6 (2)	12 (2)	31 (5)	49 (7)
Mexico	188 (14)	289 (16)	389 (18)	429 (20)	473 (21)	462 (22)	445 (21)
Myanmar	24 (4)	37 (4)	60 (6)	82 (9)	109 (11)	157 (17)	207 (26)
Mozambique	11 (5)	15 (5)	26 (7)	30 (7)	48 (8)	58 (8)	68 (9)
Mauritania	0 (0)	1 (3)	2 (4)	3 (4)	3 (4)	4 (4)	4 (4)
Malawi	4 (3)	8 (5)	15 (6)	22 (7)	29 (7)	42 (8)	54 (8)
Malaysia	19 (9)	25 (10)	34 (12)	48 (14)	51 (13)	48 (14)	72 (18)
New Caledonia	0 (3)	0 (7)	0 (13)	0 (16)	0 (22)	0 (25)	0 (17)
Nigeria	43 (3)	27 (1)	55 (2)	69 (2)	88 (2)	50 (1)	192 (3)
Nicaragua	5 (6)	6 (6)	12 (10)	18 (13)	49 (35)	56 (43)	54 (44)
Nepal	11 (3)	13 (3)	18 (3)	22 (3)	55 (6)	51 (7)	51 (7)
New Zealand	4 (9)	5 (9)	5 (10)	7 (13)	8 (15)	10 (18)	9 (15)
Oman	0 (0)	0 (0)	0 (0)	0 (0)	0 (1)	0 (1)	1 (1)
Pakistan	12 (1)	17 (1)	23 (1)	35 (1)	45 (1)	48 (1)	56 (1)

Panama	7 (24)	10 (28)	15 (39)	19 (45)	22 (48)	22 (45)	22 (46)
Philippines	11 (2)	19 (2)	107 (10)	244 (19)	538 (37)	738 (52)	983 (66)
Papua New Guinea	0 (1)	2 (2)	5 (6)	11 (11)	18 (14)	25 (16)	23 (15)
Dem. Rep. Korea	1 (0)	1 (0)	1 (0)	15 (5)	30 (10)	27 (10)	24 (10)
Russian Federation	1 (0)	1 (0)	1 (0)	2 (0)	1 (0)	1 (0)	2 (0)
Saudi Arabia	0 (0)	0 (0)	0 (0)	0 (0)	12 (3)	15 (3)	16 (3)
Senegal	0 (0)	0 (0)	3 (2)	8 (3)	8 (3)	65 (16)	77 (16)
Solomon Islands	0 (0)	0 (4)	0 (3)	0 (9)	1 (22)	3 (48)	4 (63)
Sierra Leone	8 (12)	11 (14)	14 (14)	6 (5)	3 (2)	2 (2)	4 (2)
El Salvador	0 (0)	0 (0)	0 (0)	0 (0)	22 (15)	45 (39)	46 (41)
Somalia	1 (1)	1 (1)	3 (2)	3 (1)	5 (2)	47 (17)	59 (19)
South Sudan	0 (0)	0 (0)	0 (0)	0 (0)	0 (0)	0 (0)	1 (0)
Suriname	0 (1)	0 (1)	0 (0)	0 (0)	0 (0)	0 (0)	0 (0)
Swaziland	1 (5)	1 (5)	1 (5)	1 (4)	1 (4)	1 (4)	0 (0)
Togo	0 (0)	0 (0)	0 (0)	16 (13)	20 (13)	46 (23)	46 (23)
Thailand	23 (2)	37 (3)	39 (3)	34 (3)	64 (7)	103 (13)	153 (22)
Timor-Leste	0 (0)	0 (0)	0 (0)	6 (23)	17 (50)	17 (50)	28 (70)
Tanzania	0 (0)	0 (0)	3 (1)	5 (1)	7 (1)	21 (1)	32 (2)
United States	220 (5)	313 (9)	371 (12)	413 (11)	448 (12)	515 (13)	513 (13)
Saint Vincent and the Grenadines	1 (25)	1 (25)	1 (25)	1 (25)	1 (25)	0 (0)	0 (0)
Venezuela	3 (1)	4 (1)	5 (1)	68 (14)	72 (14)	74 (14)	74 (14)
Vietnam	60 (5)	106 (8)	202 (14)	275 (17)	302 (23)	396 (31)	472 (37)
Vanuatu	0 (0)	0 (11)	0 (25)	0 (23)	0 (25)	0 (25)	0 (25)
Samoa	0 (0)	0 (0)	0 (0)	0 (25)	0 (25)	0 (0)	0 (0)
Yemen	8 (5)	11 (5)	39 (12)	61 (12)	88 (15)	121 (17)	149 (17)
South Africa	10 (2)	15 (2)	21 (2)	24 (2)	18 (2)	21 (2)	18 (2)
Zimbabwe	1 (1)	2 (1)	27 (9)	33 (9)	42 (11)	49 (12)	45 (12)

Table 19: **Ensemble members per extreme event category and GMT pathway**

GMT pathway	Wildfires	Crop failures	Droughts	River floods	Heatwaves	Tropical cyclones
1.5°C	34	15	56	56	8	8
1.6°C	34	15	56	56	8	8
1.7°C	34	15	56	56	8	8
1.8°C	29	12	48	48	7	7
1.9°C	29	12	48	48	7	7
2.0°C	29	12	48	48	7	7
2.1°C	29	12	48	48	7	7
2.2°C	25	9	41	41	6	6
2.3°C	25	9	41	41	6	6
2.4°C	25	9	41	41	6	6
2.5°C	25	9	41	41	6	6
2.6°C	20	6	33	33	5	5
2.7°C	20	6	33	33	5	5
2.8°C	15	3	25	25	4	4
2.9°C	15	3	25	25	4	4
3.0°C	15	3	25	25	4	4
3.1°C	15	3	25	25	4	4
3.2°C	15	3	25	25	4	4
3.3°C	15	3	25	25	4	4
3.4°C	15	3	25	25	4	4
3.5°C	15	3	25	25	4	4

Table 20: **The thresholds and models used for defining extreme events. In cases where models are not listed as global climate models (for all extremes other than heatwaves), global climate models provide boundary conditions to the listed models.**

Event	Models	Thresholds in exposure definition
**Wildfire**	Global vegetation models: CARAIB, LPJ-GUESS, LPJmL, ORCHIDEE, VISIT	-
**Crop failure**	Global gridded crop models: GEPIC, LPJmL, PEPIC	2.5% of local pre-industrial reference yield
**Drought**	Global hydrological models: CLM4.5, H08, LPJmL, JULES-W1, MPI-HM, ORCHIDEE, PCR-GLOBWB, WaterGAP2	2.5% of local pre-industrial baseline soil moisture
**River Flood**	Global hydrological models: CLM4.5, H08, LPJmL, JULES-W1, MPI-HM, ORCHIDEE, PCR-GLOBWB, WaterGAP2	100-year return level in local pre-industrial discharge
**Heatwave**	Global climate models: GFDL-ESM2M, HadGEM2-ES, IPSL-CM5A-LR, MIROC5	HWMId of that year exceeds the 99th percentile of the pre-industrial control HWMId distribution in that grid cell [1]. This incorporates daily maximum temperature (hereby "t_{dmax}") comparisons across time scales of pre-industrial control data by (i) assessing hot periods of at least 3 consecutive days as having each day exceed the 90% of pre-industrial, 31-day centered windows of t_{dmax} and (ii) computing the HWMId of these hot periods against the 25% and 75% of pre-industrial, annual maximum t_{dmax}
**Tropical cyclone**	Dynamical tropical cyclone model [2] and wind-field model [3]	1 minute of 64 kt winds

Supplementary Figures

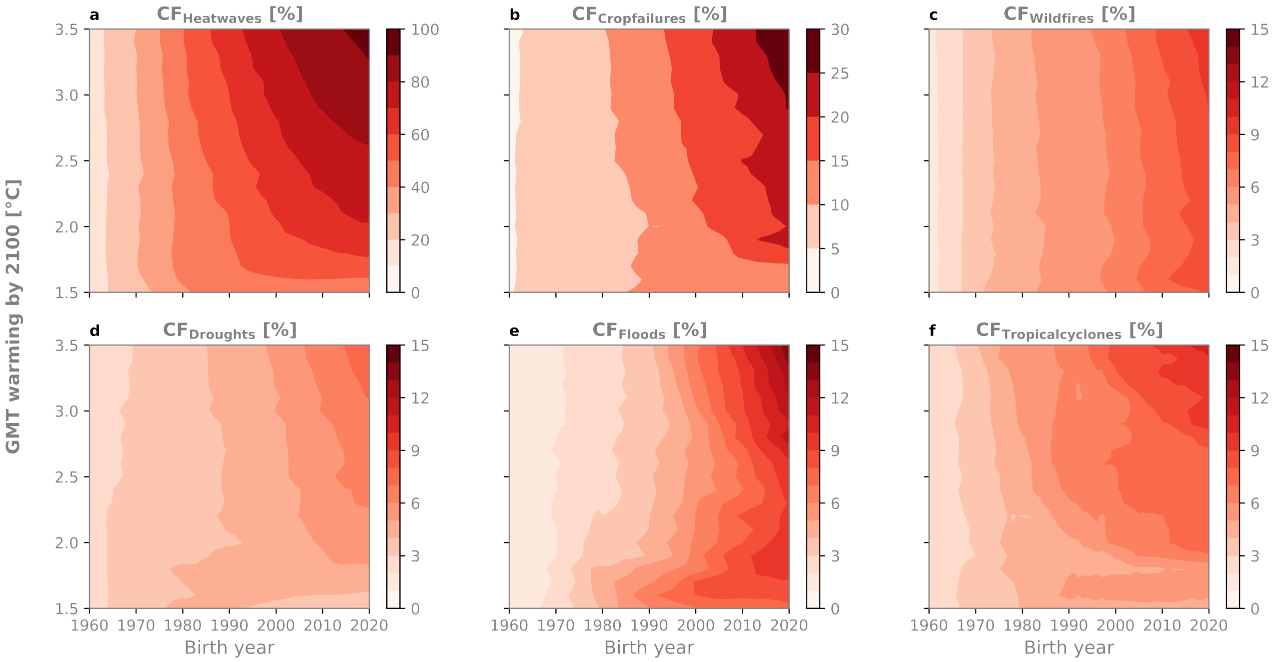

Supplementary Figure 1 | Heatmaps of multi-model mean CF for all extreme events when ensembles are limited to simulations valid for GMT pathways. CF shown across all birth years (1960 to 2020) and GMT pathways (1.5 to 3.5 °C pathways) for wild fires ($CF_{wildfires}$; **a**), crop failures ($CF_{cropfailures}$; **b**), droughts ($CF_{droughts}$; **c**), river floods (CF_{floods}; **d**), heatwaves ($CF_{heatwaves}$; **e**) and tropical cyclones ($CF_{tropicalcyclones}$; **f**). Each extreme event panel has its own colorbar range in percents.

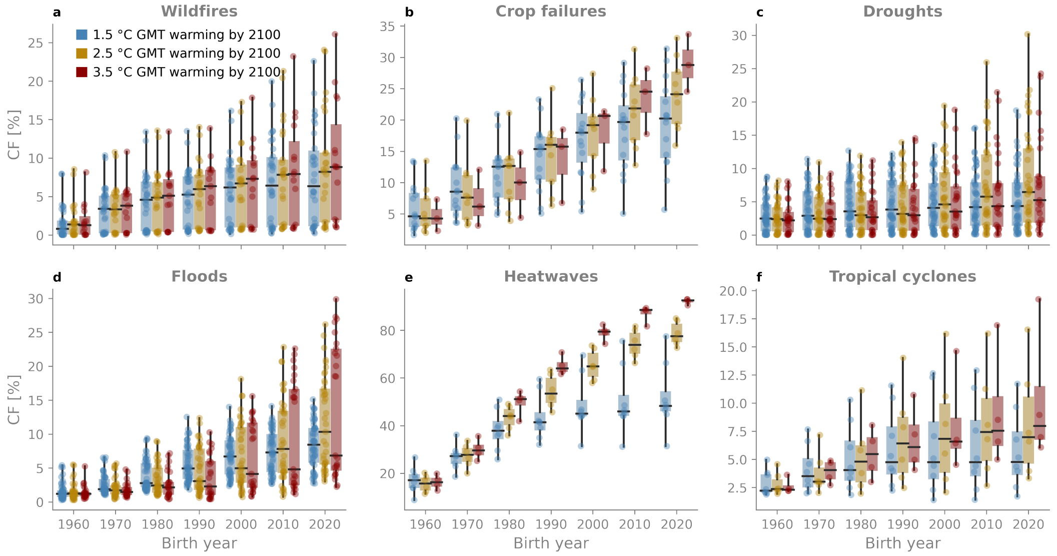

Supplementary Figure 2 | Birth cohort fractions experiencing unprecedented levels of exposure to extremes in 1.5, 2.5 and 3.5 °C pathways. Blue, gold and red box plots show CF for 1.5, 2.5 and 3.5 °C pathways and birth cohorts between 1960 and 2020 for wildfires (**a**), crop failures (**b**), droughts (**c**), river floods (**d**), heatwaves (**e**) and tropical cyclones (**f**).

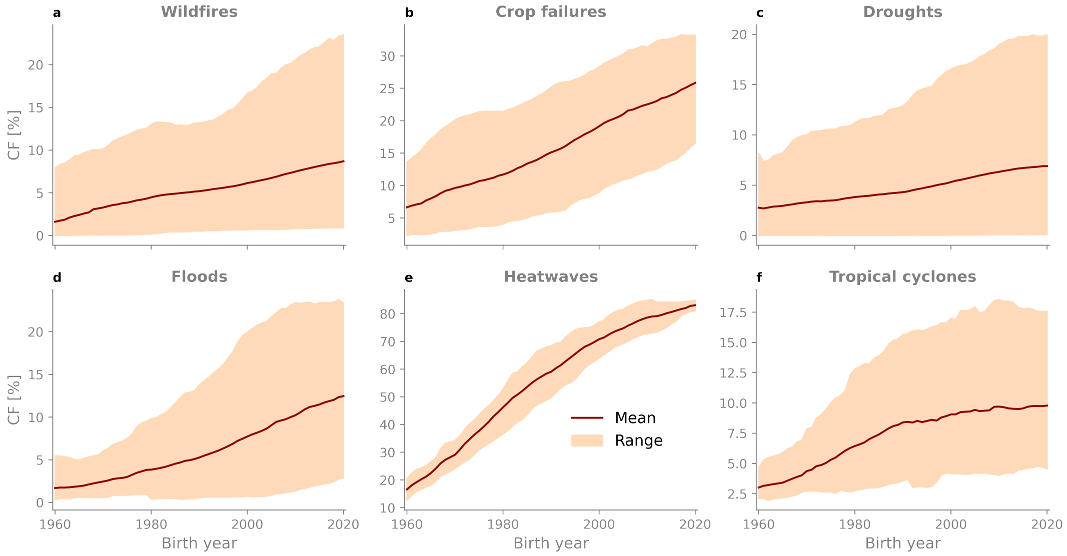

Supplementary Figure 3 | Birth cohort fractions experiencing unprecedented levels of exposure to extremes in 2.7 °C pathways. Multi-model mean time series and range across projections for CF in 2.7 °C pathway and birth cohorts between 1960 and 2020 for wildfires (**a**), crop failures (**b**), droughts (**c**), river floods (**d**), heatwaves (**e**) and tropical cyclones (**f**).

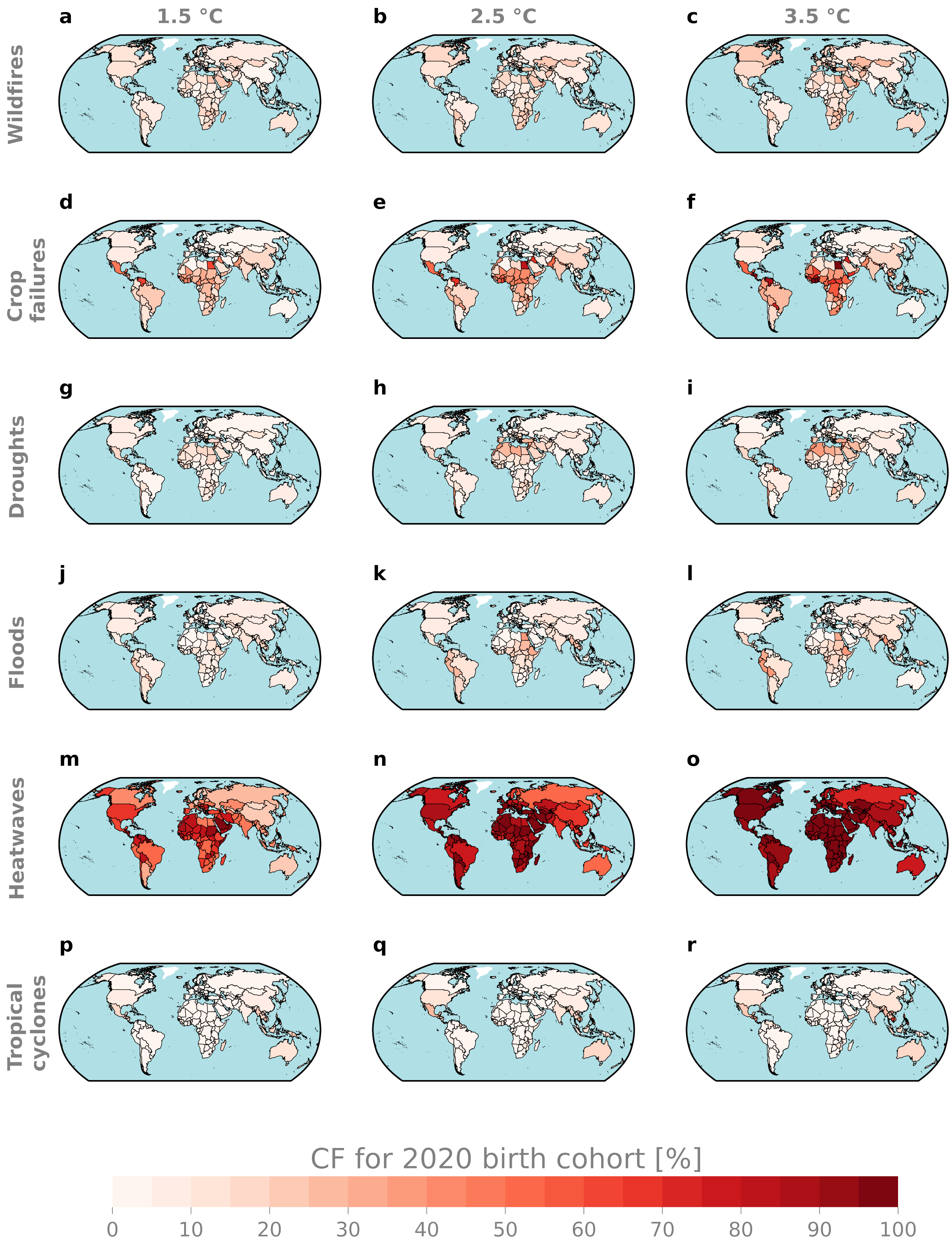

Supplementary Figure 4 | Maps of country level CF of the 2020 birth cohort for 1.5, 2.5 and 3.5 °C pathways for all extreme events. CF is shown for wildfires (**a,g,m**), crop failures (**b,h,n**), droughts (**c,i,o**), river floods (**d,j,p**), heatwaves (**e,k,q**) and tropical cyclones (**f,l,r**)

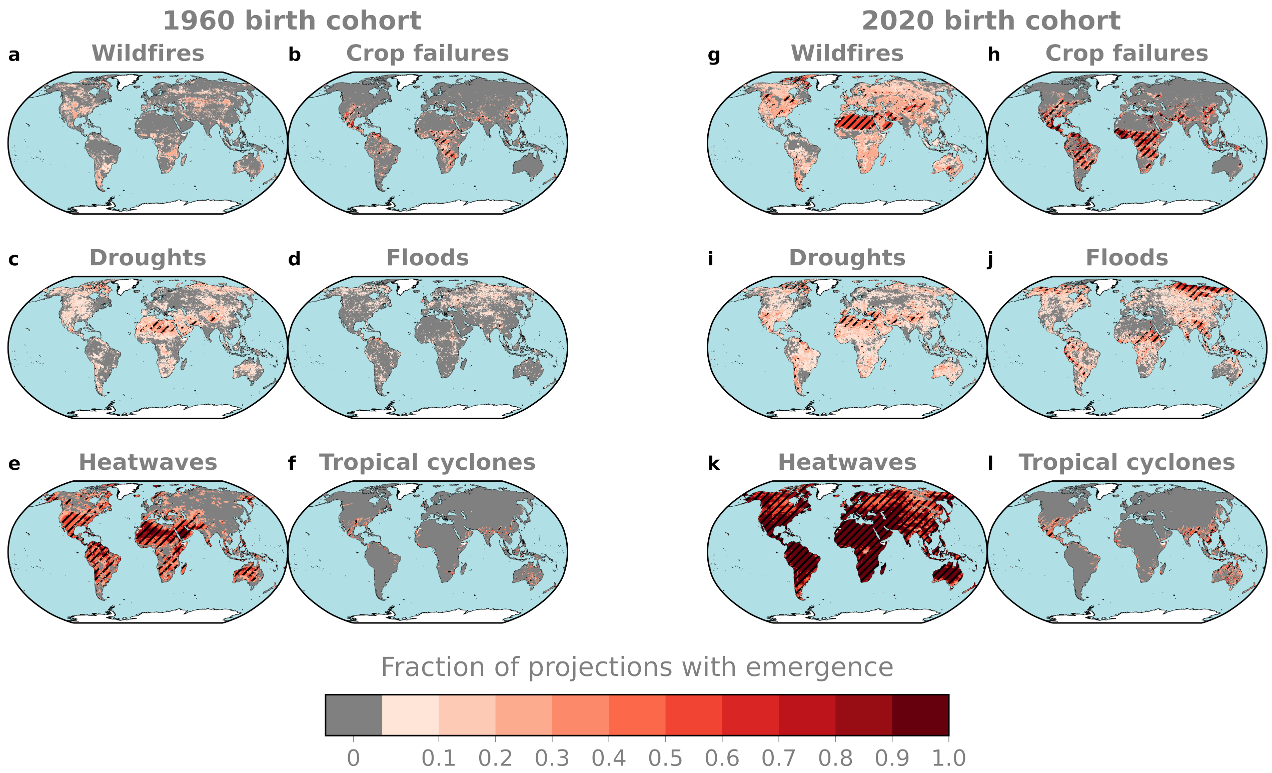

Supplementary Figure 5 | Locations of emergence shown as the fraction of projections reaching unprecedented exposure. Fractions of projections with emergence for each extreme event for the 1960 birth cohort (**a-f**) and the 2020 birth cohort (**h-m**) in a 2.7 °C pathway, in line with the Climate Action Tracker current pledges estimate. Black hatching marks regions where emergence into unprecedented exposure occurs for ≥25% of the event category's ensemble of projections.

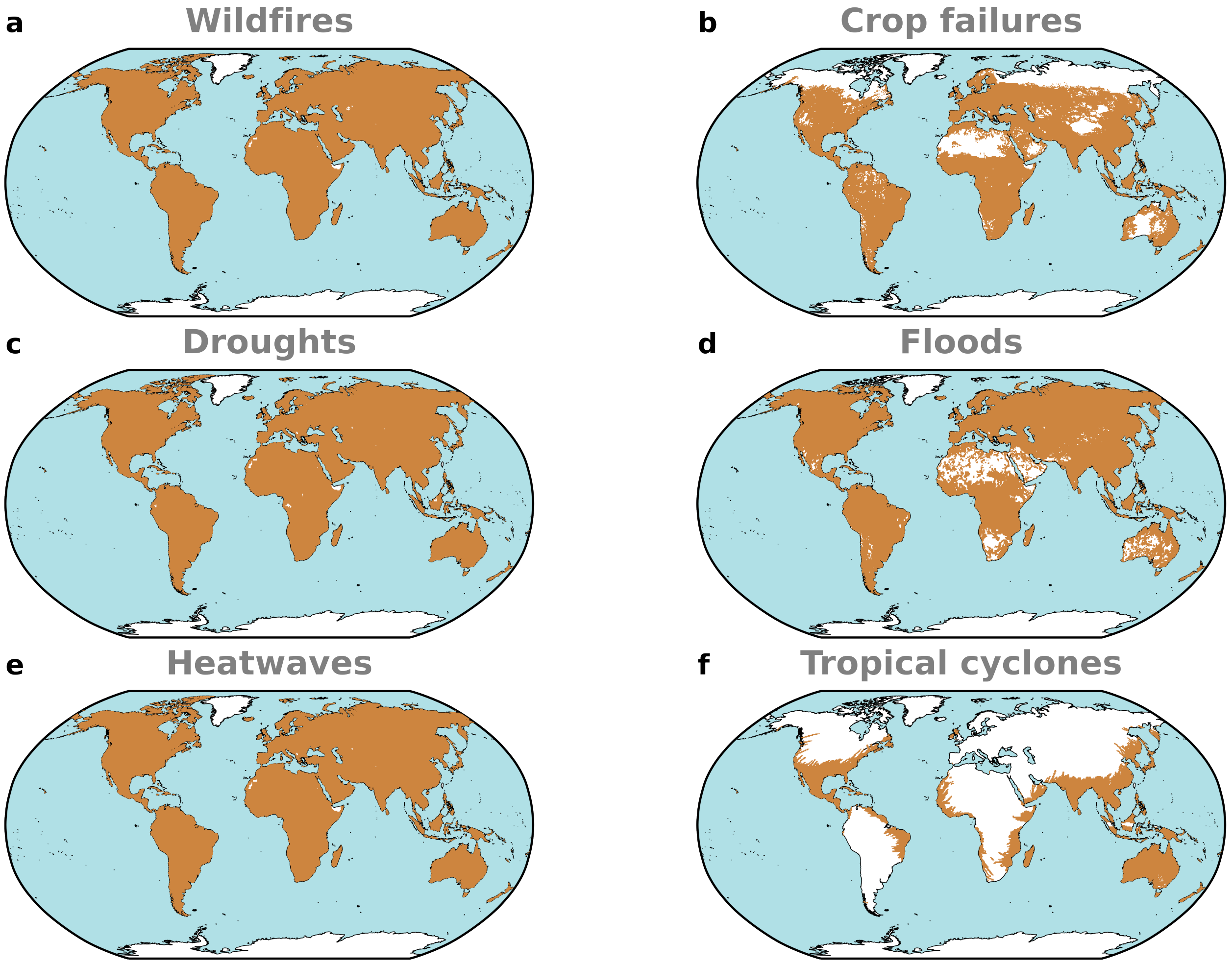

Supplementary Figure 6 | Locations where exposure to extreme events occurs. Grid cells (brown) where exposure occurs for each extreme event in any projections in our ensemble at any time for wildfires (**a**), crop failures (**b**), droughts (**c**), river floods (**d**), heatwaves (**e**) and tropical cyclones (**f**).

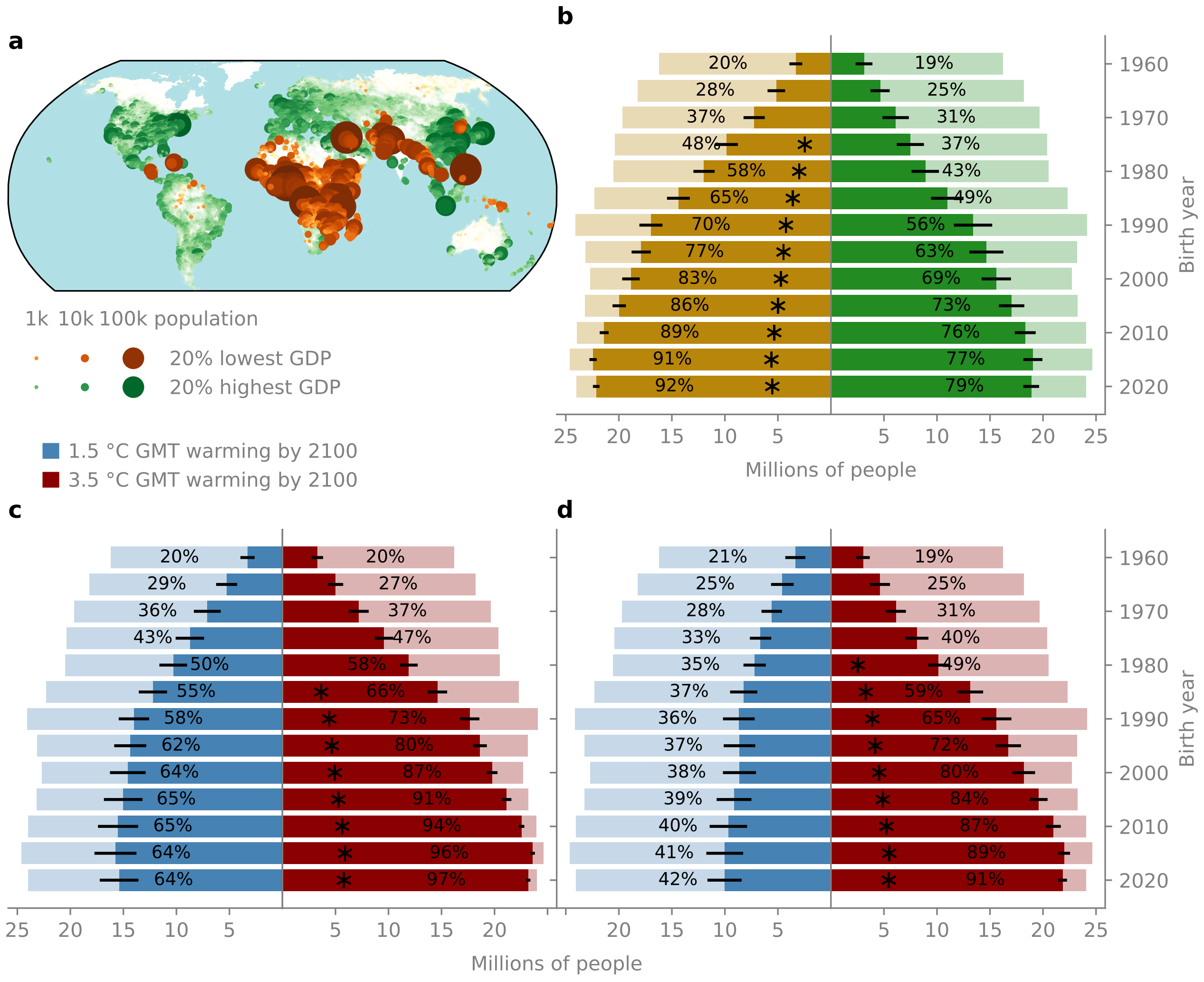

Supplementary Figure 7 | The poorest experience significantly more unprecedented exposure to heatwaves. Geographic distribution of 2020 birth cohort belonging to the 20% lowest (**a**; brown markers) and 20% highest (**a**; green markers) in lifetime mean gross domestic product (GDP). Grid cell marker sizes and colors are scaled by their population. The birth cohort membership of these two groups projected to experience ULE to heatwaves under the current policies pathway of 2.7 °C warming by 2100 for every 5th birth year (**b**). Error bars show the standard deviation in projections. Asterisks imply that a given birth cohort and low- or high-vulnerability group has significantly more members with ULE to heatwaves than the alternative vulnerability group of the same birth cohort (at the 5% level). The low- (**c**) and high-GDP (**d**) group's membership that is projected to experience ULE under 1.5 and 3.5 °C pathways. Light colored bars show total cohort sizes per birth year and vulnerability class.

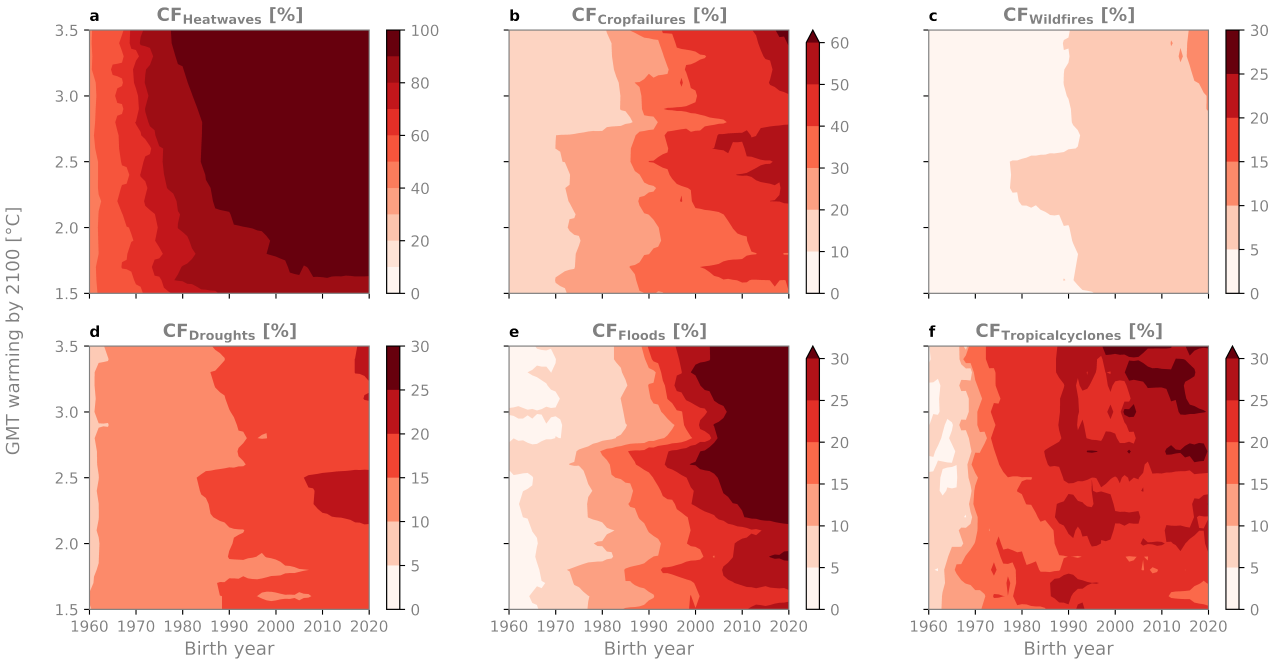

Supplementary Figure 8 | Heatmaps of multi-model mean CF for all extreme events using country average exposure. CF shown across all birth years (1960 to 2020) and GMT trajectories (1.5 to 3.5 °C pathways) for wild fires ($CF_{wildfires}$; **a**), crop failures ($CF_{cropfailures}$; **b**), droughts ($CF_{droughts}$; **c**), river floods (CF_{floods}; **d**), heatwaves ($CF_{heatwaves}$; **e**) and tropical cyclones ($CF_{tropicalcyclones}$; **f**). Each extreme event panel has its own colorbar range in percents.

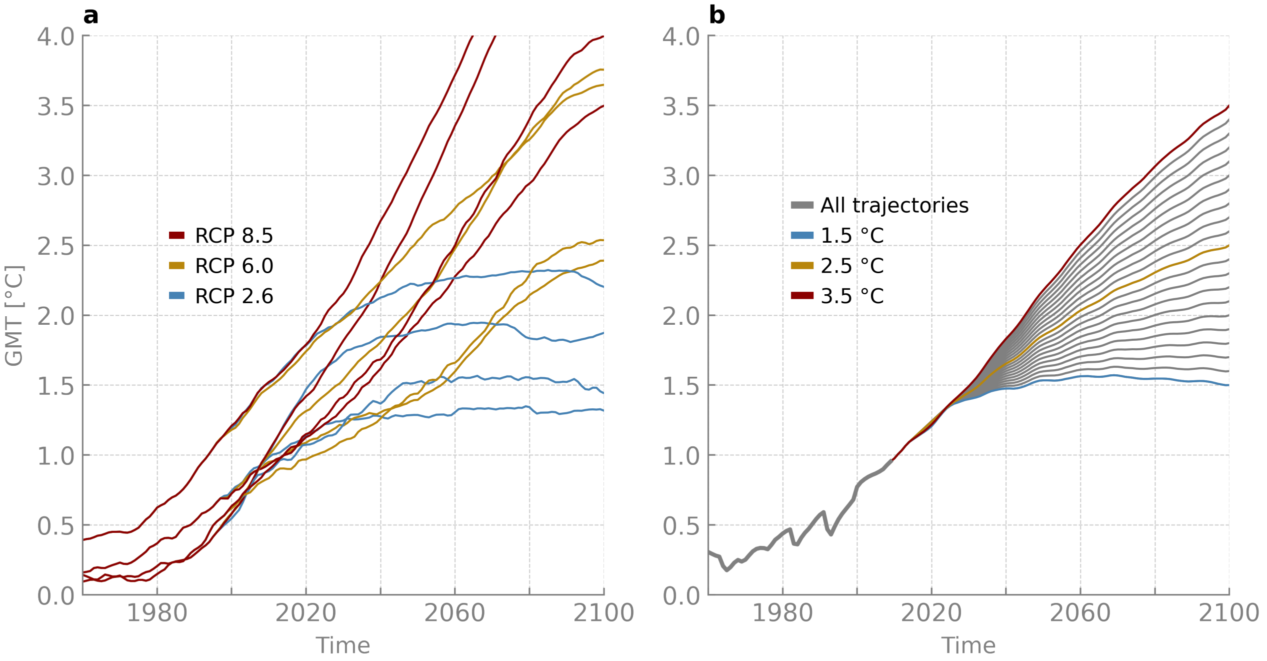

Supplementary Figure 9 | GMT pathways of RCP forcings and AR6 time series' used in mapping exposure to climate extremes. GMT time series of RCPs 2.6, 6.0 and 8.5 smoothed with a 21-year rolling mean (**a**) and pathways generated for mapping exposure projections to specific warming targets (**b**).

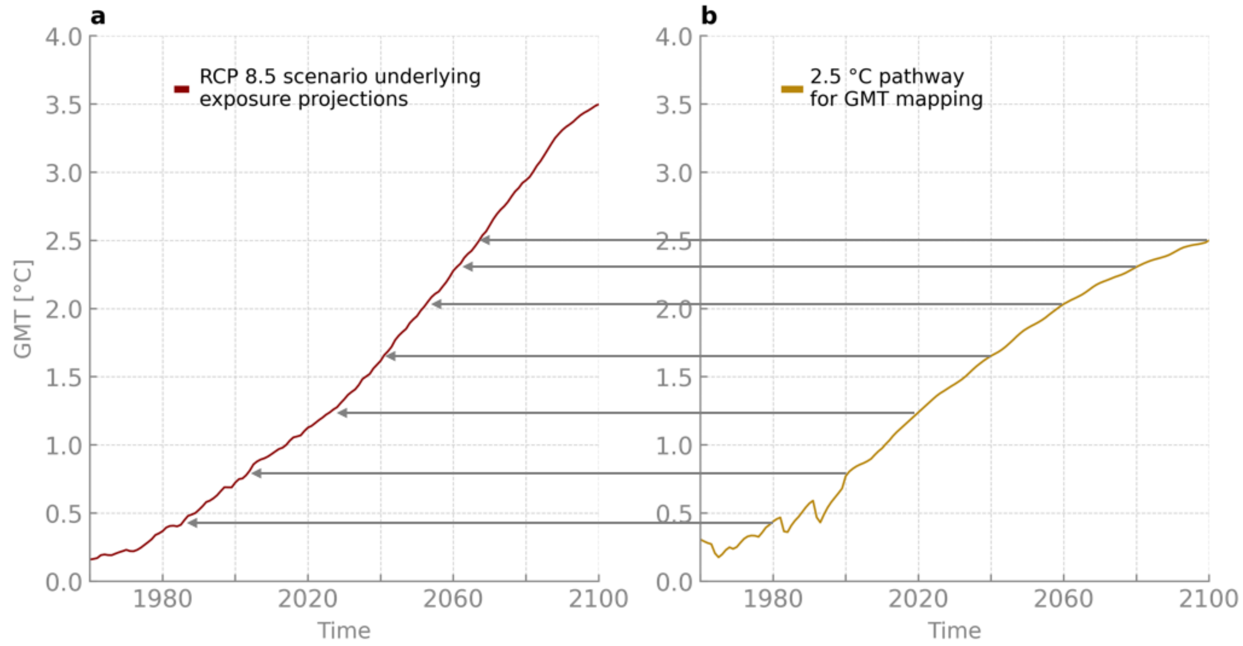

Supplementary Figure 10 | Mapping extreme event exposures from radiative forcing scenarios to GMT pathways. Per year (GMT anomaly) of the GMT pathway considered for the lifetime exposure analysis (**b**; 2.5 °C), we find the closest GMT anomaly from the original exposure projections to select an annual map of exposed area (**a**; RCP 8.5).

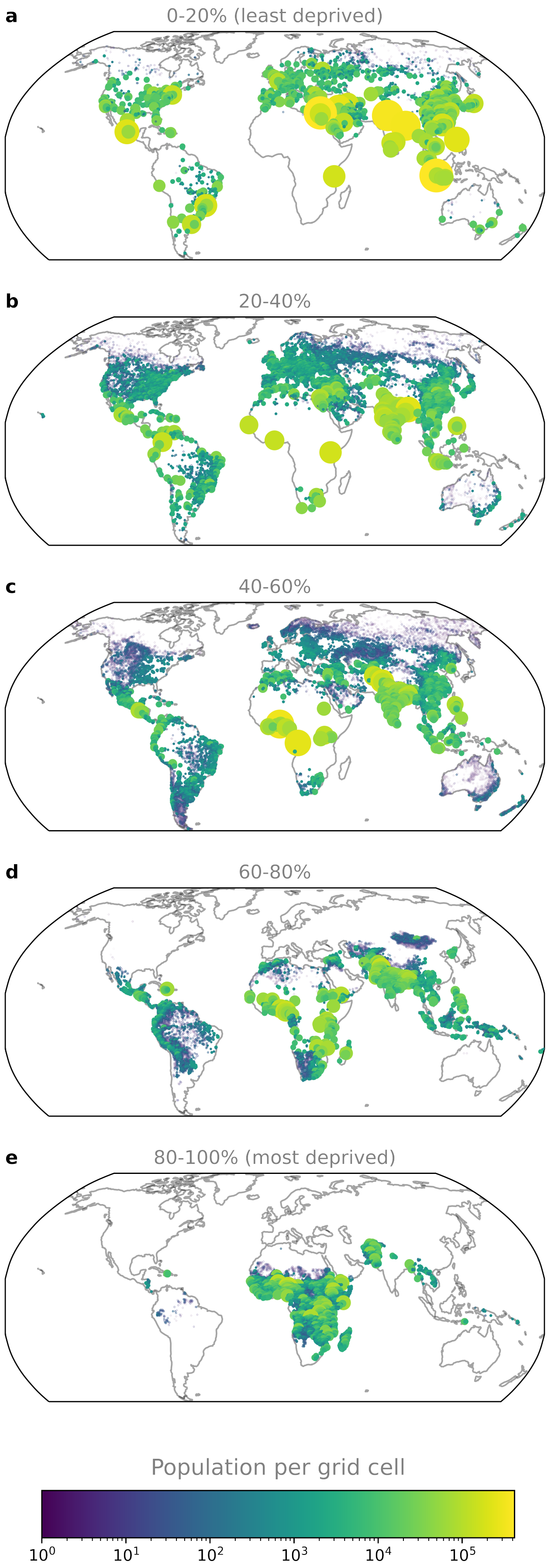

Supplementary Figure 11 | Geographic distribution of GRDI quantile ranges. The lowest ranking in the GRDI depravity index globally for the 2020 birth cohort (0-20% or least deprived; **a**) to the highest ranking (80-100% or most deprived; **e**), as binned by population. Populations of each grid cell are communicated by color and marker size.

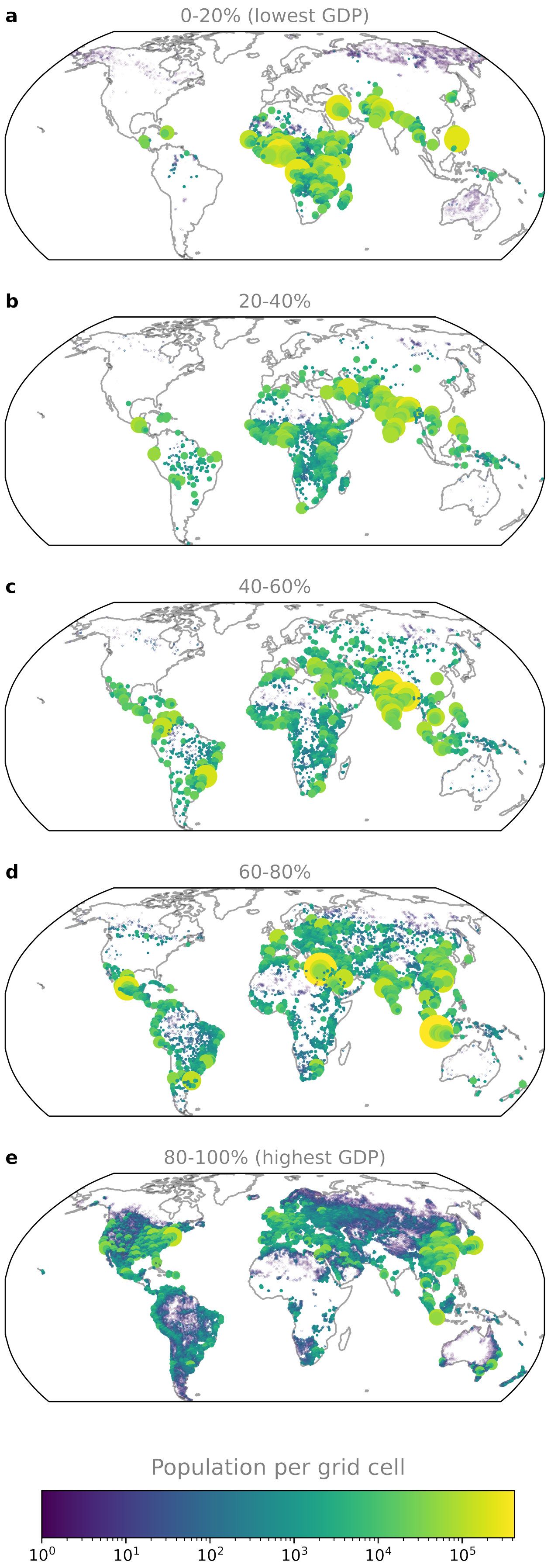

Supplementary Figure 12 | Geographic distribution of lifetime mean GDP quantile ranges. The lowest ranking in lifetime mean GDP globally for the 2020 birth cohort (0-20%; **a**) to the highest ranking (80-100%; **e**), as binned by population. Populations of each grid cell are communicated by color and marker size.

1 Supplementary Notes

1.1 Supplementary Note 1: Sensitivity of results to ensemble sampling

Our multi-model mean projections of CF for crop failures, droughts, river floods and tropical cyclones exhibit discontinuities across GMT pathways (see Fig. 3b-e). These are artifacts originating from our ensemble sampling procedure. We map years of simulations forced by RCP scenarios to AR6 GMT pathways according to GMT warming levels in order to project lifetime exposure along specific warming scenarios (Supplementary Fig. 8). Candidate simulations are only used when a threshold criteria is met which determines the maximum possible difference in GMT anomaly between matched years in this mapping procedure (see Methods). Therefore, the number of available simulations for GMT mapping reduces incrementally as the GMT warming pathway increases between 1.5 and 3.5 °C by 2100 (see Supplementary Table 19).

Our ensemble of exposure projections consists of impact models (such as global hydrological models for river floods) forced by four GCM atmospheres under different RCPs. Therefore, the above constraints on our ensemble size means that we can lose the influence of relatively wet or dry atmospheres forcing the impact models as GMT pathways increase. Crop failures, droughts, river floods and tropical cyclones all reduce in CF before the 2.9 °C pathway (Fig. 3b-e), implying that at this GMT transition we remove relatively wet GCM atmospheric data. To test this, we reproduced the ensemble mean CF for all extremes using only those projections that are viable under the above criteria for all GMT pathways in our analysis (Supplementary Fig. 1). In doing so, we lose the jumps before the 2.9 °C pathway. In this constrained estimate, droughts reach a maximum at the end points of the analysis (the 3.5 °C pathway for the 2020 birth cohort; Supplementary Fig. 1c) instead of at an lower GMT pathway (see Fig. 3). Otherwise, end point projections for CF remain mostly consistent between both approaches. Despite the discontinuities in the estimates using the full ensemble, we feel it is necessary to publish those findings in the main text to capture as much model uncertainty as possible in our final result. Naturally, we expect that a greater ensemble size in this analysis would smooth out these artifacts, which would be the case once ISIMIP3b data is published and likewise used to project exposure.

1.2 Supplementary Note 2: Uncertainty in projections of hydroclimate extremes

CF for extremes other than heatwaves have relatively larger uncertainties. This is visible in the large envelopes of their CF projections across birth years for given GMT pathways (Supplementary Fig. 2,3). To quantify how this affects the significance of the change in CF between 1960 and 2020 birth cohorts relative to the variance in our ensemble estimates of CF, we performed

paired-sample t-tests for each extreme in a 2.7 °C pathway. For each extreme, the two samples are the CF projections for 1960 and 2020 birth cohorts, and our null hypothesis is that CF is not different across 1960 and 2020 birth cohorts at a 95% significance level. We opt for the paired test because CF for 1960 and 2020 birth cohorts per projection are not independent given that they source the same underlying simulation in GMT mapping. Projections of CF for 1960 and 2020 birth cohorts for all extremes are significantly different in this test ($p_{\text{wildfires}} = 1.00\text{x}10^{-5}$; $p_{\text{cropfailures}} = 3.49\text{x}10^{-5}$; $p_{\text{droughts}} = 3.65\text{x}10^{-6}$; $p_{\text{floods}} = 2.47\text{x}10^{-12}$; $p_{\text{heatwaves}} = 2.70\text{x}10^{-6}$; $p_{\text{tropicalcyclones}} = 0.014$). Additionally, for each extreme, we compare the coefficient of variation, $CV = \sigma/\mu$, against the ratio of the change in CF between 1960 and 2020 birth cohorts and the overall mean CF, $\frac{\mu_{2020}-\mu_{1960}}{\mu}$. If $\mu_{2020} - \mu_{1960}$ is proportionally larger than σ, this provides evidence of significant changes in the multi-model mean. For all extremes other than droughts ($\frac{\mu_{2020}-\mu_{1960}}{\mu} = 0.91$ and $CV = 0.96$), $\frac{\mu_{2020}-\mu_{1960}}{\mu}$ is greater than CV.

The extremes in this analysis with large uncertainty in projections of CF are dependent on an accurately represented water cycle. Yet, water cycle systems contain some of the most challenging processes to represent in climate and impact modelling [4]. Uncertainties in simulating the water cycle stem from representing Earth's climate, terrestrial processes, human management factors and their interactions. For climate, this includes challenges such as extreme precipitation [5], cloud formation [6] and feedbacks with internal climate variability across systems [7]. Terrestrial modelling challenges include land-atmosphere interactions like local surface energy fluxes [8] and evapotranspiration [9], landscape features and hydrological processes like infiltration, soil moisture and river routing [10]. Representing human management touches many systems, such as forestry and wildfire management, crop management and irrigation, river canalization, water use and urbanization. Feedbacks across these categories are widespread and include the effects of warming on seasonal snow melt and runoff [11], the effects of drought on water availability for municipal and irrigation use [12–14] and biogeophysical land use impacts on local climates [15, 16].

Locally, some of the emergence of lifetime exposure into unprecedented levels needs to be interpreted with caution. Specifically, drought and wildfire occurrences in the Sahara desert (Supplementary Fig. 4,5c,g,i). However, this potentially unrealistic behaviour is limited to small sub-regions and has little influence on CF at larger scale.

For a literature review and evaluation of past applications of the models projecting these extreme event categories, we refer to Section 2 of Lange et al. [17].

1.3 Supplementary Note 3: Country scale CF for hydroclimate variables

Our methodology for quantifying CF uses lifetime exposure estimates at the grid scale, yet country-average exposure can also be used here. In the previous study using this dataset [1], exposures projected at the grid scale from ISIMIP2b datasets were first aggregated for each country before summing exposures across life expectancy estimates. This was calculated as a population weighted mean using absolute population estimates at the grid scale (see Methods), thereby accounting for the spatial distribution of a country's annual population in its annual, country-average exposure. The resulting definition of mean exposure for a country takes on some ambiguity, representing either the fraction of a country's population that was exposed to an extreme or the average number of extreme event exposures experienced by a person in a country.

Country-average lifetime exposure can be used to estimate CF, but this methodological approach has drawbacks relative to the grid scale analysis used in our study. This occurs in tallying demographics based on country level emergence. For a given birth cohort, when comparing lifetime exposure under different GMT pathways to extreme pre-industrial lifetime exposure at a country level, we lose the ability to project emergence in a spatially explicit way. In other words, when a birth cohort's exposure emerges into unprecedented levels, its country-wide membership is tallied into the global pool of people of that global birth cohort living unprecedented exposure. The country level analysis therefore imparts large fluctuations on the time series of global CF across birth cohorts or GMT pathways. Here, ensemble variance in exposure projections can mean the difference between entire birth cohorts from countries like India, China or the United States being tallied into global pools of people living unprecedented lives. This yields larger and noisier estimates of CF for each extreme than in the grid scale approach (Supplementary Fig. 7). The country level approach also increases the sensitivity of results to ensemble sampling (see Supplementary Note 1), especially for less spatially ubiquitous event categories like river floods and tropical cyclones (Supplementary Fig. 8d,f). In contrast, for the grid scale approach used in this study, emergence within a country advances regionally across birth years and GMT pathways. For the grid scale approach, the sums of birth cohort members living in locations of emergence are less chaotic across birth cohorts or GMT pathways.

Supplementary References

[1] Thiery, W. *et al.* Intergenerational inequities in exposure to climate extremes. *Science* **374**, 158–160 (2021).

[2] Emanuel, K. Downscaling CMIP5 climate models shows increased tropical cyclone activity over the 21st century. *Proceedings of the National Academy of Sciences* **110**, 12219–12224 (2013).

[3] Holland, G. A revised hurricane pressure-wind model. *Monthly Weather Review* **136**, 3432–3445 (2008).

[4] Marotzke, J. *et al.* Climate research must sharpen its view. *Nature Climate Change* **7**, 89–91 (2017). URL `https://doi.org/10.1038/nclimate3206`.

[5] Kim, S., Eghdamirad, S., Sharma, A. & Kim, J. H. Quantification of Uncertainty in Projections of Extreme Daily Precipitation. *Earth and Space Science* **7**, e2019EA001052 (2020). URL `https://doi.org/10.1029/2019EA001052https://agupubs.onlinelibrary.wiley.com/doi/10.1029/2019EA001052`.

[6] Miao, H., Wang, X., Liu, Y. & Wu, G. A Regime-Based Investigation Into the Errors of CMIP6 Simulated Cloud Radiative Effects Using Satellite Observations. *Geophysical Research Letters* **48** (2021).

[7] Hegerl, G. C. *et al.* Challenges in Quantifying Changes in the Global Water Cycle. *Bulletin of the American Meteorological Society* **96**, 1097–1115 (2015). URL `https://journals.ametsoc.org/view/journals/bams/96/7/bams-d-13-00212.1.xmlhttps://journals.ametsoc.org/doi/10.1175/BAMS-D-13-00212.1`.

[8] Grant, L. *et al.* Biogeophysical Effects of Land-Use and Land-Cover Change Not Detectable in Warmest Month. *Journal of Climate* **36**, 1845–1861 (2023).

[9] Long, D., Longuevergne, L. & Scanlon, B. R. Uncertainty in evapotranspiration from land surface modeling, remote sensing, and GRACE satellites. *Water Resources Research* **50**, 1131–1151 (2014). URL `https://doi.org/10.1002/2013WR014581http://doi.wiley.com/10.1002/2013WR014581`.

[10] Gao, H. *et al.* Landscape heterogeneity and hydrological processes: a review of landscape-based hydrological models. *Landscape Ecology* **33**, 1461–1480 (2018). URL `https://doi.org/10.1007/s10980-018-0690-4`.

[11] Barnett, T. P. *et al.* Human-Induced Changes in the Hydrology of the Western United States. *Science* **319**, 1080–1083 (2008). URL `https://doi.org/10.1126/science.1152538`.

[12] Cook, B. I., Mankin, J. S. & Anchukaitis, K. J. Climate Change and Drought: From Past to Future. *Current Climate Change Reports* **4**, 164–179 (2018). URL https://doi.org/10.1007/s40641-018-0093-2.

[13] Thiery, W. *et al.* Present-day irrigation mitigates heat extremes. *Journal of Geophysical Research* **122** (2017).

[14] Thiery, W. *et al.* Warming of hot extremes alleviated by expanding irrigation. *Nature Communications* **11**, 1–15 (2020).

[15] Lejeune, Q., Davin, E. L., Gudmundsson, L., Winckler, J. & Seneviratne, S. I. Historical deforestation locally increased the intensity of hot days in northern mid-latitudes. *Nature Climate Change* **8**, 386–390 (2018).

[16] De Hertog, S. J. *et al.* The biogeophysical effects of idealized land cover and land management changes in Earth System Models. *Earth System Dynamics Discussions* 1–53 (2022).

[17] Lange, S. *et al.* Projecting Exposure to Extreme Climate Impact Events Across Six Event Categories and Three Spatial Scales. *Earth's Future* **8**, 1–22 (2020).
